# TPOT-NN: augmenting tree-based automated machine learning with neural network estimators

**DOI:** 10.1007/s10710-021-09401-z

**Published:** 2021-03-02

**Authors:** Joseph D. Romano, Trang T. Le, Weixuan Fu, Jason H. Moore

**Affiliations:** 1 Institute for Biomedical Informatics, University of Pennsylvania, Philadelphia, PA 19104, USA; 2 Center of Excellence in Environmental Toxicology, University of Pennsylvania, Philadelphia, PA 19104, USA

**Keywords:** Automated machine learning, Genetic programming, Evolutionary algorithms, Artificial neural networks, Pareto optimization

## Abstract

Automated machine learning (AutoML) and artificial neural networks (ANNs) have revolutionized the field of artificial intelligence by yielding incredibly high-performing models to solve a myriad of inductive learning tasks. In spite of their successes, little guidance exists on when to use one versus the other. Furthermore, relatively few tools exist that allow the integration of both AutoML and ANNs in the same analysis to yield results combining both of their strengths. Here, we present TPOT-NN—a new extension to the tree-based AutoML software TPOT—and use it to explore the behavior of automated machine learning augmented with neural network estimators (AutoML+NN), particularly when compared to non-NN AutoML in the context of simple binary classification on a number of public benchmark datasets. Our observations suggest that TPOT-NN is an effective tool that achieves greater classification accuracy than standard tree-based AutoML on some datasets, with no loss in accuracy on others. We also provide preliminary guidelines for performing AutoML+NN analyses, and recommend possible future directions for AutoML+NN methods research, especially in the context of TPOT.

## 1 Introduction

Automated machine learning (AutoML) and artificial neural networks (NNs, or ANNs) comprise two paradigms for building highly performing models that dramatically outperform other classes of models in a variety of scenarios, including on classification and regression tasks. In spite of their successes, there remains substantial debate—and little quantitative evidence—on the practical advantages of the two approaches and how to determine which will perform best on specific real-world problems, resulting in NNs (especially in the context of deep learning) and AutoML often being seen as competing methods. Luckily, there has been growing interest in combining AutoML and NNs to further improve their performance by capitalizing on their collective advantages. For example, Cartesian genetic programming is a branch of genetic programming (GP) that encodes programs as a 2-dimensional grid of graph nodes [23]—since nodes in a neural network can easily be represented as nodes in a graph, NNs have emerged as a natural and effective implementation of Cartesian GP [13, 17]. Several other notable examples of NNs used in an AutoML context also exist, including Auto-Net [15] and Amazon’s AutoGluon [10], but none are both widely available and allow discovery of pipelines that mix neural networks with other non-NN estimators within the same ML pipeline.

We sought to explore these issues by expanding the AutoML tool TPOT (described below in Sect. 2.2.1) to utilize NN estimators in its classification pipelines. We refer to this extension to TPOT as TPOT-NN. We then evaluated TPOT-NN by comparing its performance on 6 well-characterized binary classification problems to non-NN TPOT (which we refer to as ‘base’ TPOT) and to NN classifiers alone (via NN-based pipelines learned by restricting TPOT to only use NN estimators stacked in GP-optimized configurations), finding that tree-based AutoML+NN attains better classification performance in certain contexts than either of these baseline approaches, albeit at the cost of increased training time. Finally, we explore the architectures of AutoML+NN pipelines learned by TPOT-NN to gain a better understanding of how this performance increase is manifested, and use the results to suggest future directions for AutoML+NN research. We provide TPOT-NN as a built-in feature of TPOT (beginning with release v0.11.4), which is freely provided online as an open-source tool for the scientific community.

In summary, this study provides the following:

- TPOT-NN is a new expansion to the tree-based AutoML software TPOT that incorporates neural network estimators into its learned ML pipelines.
- TPOT-NN performs at least as well as—and sometimes significantly better than—non-NN TPOT.
- Currently, the primary tradeoff in adding NN estimators to TPOT is increased model training time.
- The architectures learned by TPOT-NN sometimes have interesting structures that imitate portions of larger, more complex deep learning architectures, a phenomenon that merits further investigation.
- This initial work on TPOT-NN lays the foundation for future studies that focus on more complex, irregularly structured data types, as well as deeper architectures.

## 2 Background

### 2.1 Artificial neural networks

Artificial neural networks are—as the name implies—artificial approximations of biological networks, comprised of individual neurons that propagate a signal to other neurons as a function of one or more inputs and a set of tunable hyperparameters. They are often used in the context of deep learning—a class of ML that uses multiple ANN layers stacked in a ‘deep’ configuration (tens or even hundreds of layers deep), which allows models to approximate highly complex and nonlinear objective functions, unlike more traditional ‘shallow’ models such as random forest, support vector machines, or linear regression [19, 30]. Logistic regression is one of the simplest nontrivial models that can be expressed in terms of an ANN, defined as

y=ϕ(Wx+b)

where x is an input vector, y is an output vector, W and b are weight matrices and bias vectors, respectively, and ϕ. is a nonlinear transformation known as an *activation function*. Here, $\phi \left(z_{i}\right)=\frac{\exp\left(z_{i}\right)}{\sum_{j}\exp\left(z_{j}\right)}$ is the softmax function—a multivariate generalization of the standard logistic function. In this study, we only consider binary classification experiments, meaning that the softmax function is identical to the logistic function $\phi (x)=\frac{\exp(x)}{\exp(x)+1}$.

The simplest ANN architecture with multiple stacked layers is the Multilayer Perceptron (MLP), given (with 1 hidden layer) by

$$h_{1}=\phi_{1}\left(W_{1}x+b_{1}\right)\;y=\phi_{2}\left(W_{2}h_{1}+b_{2}\right)$$

In both of these models, the inductive learning task is to find the values of $W_{\left(\cdot \right)}$ and $b_{\left(\cdot \right)}$ that minimize the output of a separately-defined loss function L on a set of testing data, usually via convex optimization over a set of training data.

LR and MLP are particularly important building blocks of larger NN architectures for several reasons. The first and most obvious of these is their mathematical simplicity. Of equal or greater importance is the fact that adding a single hidden layer to an ANN (i.e., moving from LR to MLP) is enough to turn a linear model into a nonlinear model. Since most traditional ML models are limited in their ability to approximate nonlinear or otherwise complex objective functions, they often perform suboptimally in comparison to neural networks, particularly on datasets where entities with similar characteristics are not cleanly separated by linear decision boundaries. Generally, increased depth or width of an ANN model increases estimation capacity. It follows that, given sufficient depth and/or width along with an appropriate learning algorithm, even structurally simple feed-forward neural networks with a finite number of neurons (such as MLPs) can approximate virtually any continuous function on compact subsets of Euclidean space [20]. Furthermore, sufficiently large NN architectures exhibit the striking phenomenon that both bias and variance can decrease as model complexity increases [3, 24], which works in opposition to the bias-variance tradeoff that plagues other ML models.

Nonetheless, the successes of DL and ANNs have been tempered by a number of important criticisms. Compared to shallow models, it is substantially more complex to parameterize a deep ANN due to the explosion of free parameters that results from increased depth of the network or width of individual layers [32]. Furthermore, ANN models are notoriously challenging to interpret since the features in a network’s intermediate layers are a combination of features from all previous layers, which effectively obscures the intuitive meaning of individual feature weights in most nontrivial cases [21, 22].

It is also worth noting that deep ANN model architectures can reach immense sizes. For example, the popular image classification network ResNet performed best in an early study when constructed with 110 convolutional layers, containing over 1.7 billion tunable parameters [14]. However, for standard binary classification on simple datasets, smaller NN architectures of relatively shallow depth can still substantially outperform non-NN learners, both in terms of error and training time [1, 6]. For the purpose of establishing a baseline comparison between AutoML and NNs, we restrict our analyses in this study to this latter case of applications—future work will involve considering substantially deeper TPOT-NN models.

### 2.2 Automated machine learning

One of the most challenging aspects of designing an ML system is identifying the best feature transformations, model architectures, and hyperparameterizations for the task at hand. For example, count data may benefit from a square-root transformation prior to being used as input to an ML estimator. Similarly, a support-vector machine (SVM) model might predict susceptibility to a certain complex genetic disease more accurately than a gradient boosting model trained on the same dataset. Also, different choices of hyperparameters within that SVM model for kernel function k and soft margin width C can lead to vastly different performances. Traditionally, these architecture considerations need to be made with the help of prior experience, brute-force search, or experimenter intuition, all of which can complicate the overall process of building the ML system and can hinder the performance of the final learned pipeline.

AutoML provides methods for automatically handling these choices given a universe of possible architecture configurations. A number of different AutoML techniques can be used to find the best architecture for a given task, but one of the most popular is based on *genetic programming* (GP). Broadly, this type of AutoML constructs trees of mathematical functions that are optimized with respect to a fitness metric such as classification accuracy [2]. Each generation of trees is constructed via random mutations to the tree’s structure or the operations performed at each node in the tree. Repeating this process for a number of training generations produces an optimal tree. Like in natural evolution, increasingly more fit architectures are propagated forward to future generations while less fit architectures “die out”.

#### 2.2.1 TPOT

TPOT (Tree-based Pipeline Automation Tool) is a Python-based AutoML tool that uses genetic programming to discover optimal ML pipelines for either regression or classification on a given (labeled) dataset [18, 25, 27]. Briefly, TPOT performs GP on trees, where nodes are comprised of *operators*, each of which falls into one of four operator types: Preprocessors, decomposition functions, feature selectors, or estimators (i.e., classifiers and regressors). Identical copies of the input data enter the tree at leaf nodes, and predictions are output at the root node. Certain operators—such as feature set selectors or model selectors—can accept input from multiple previous operators, comprising ‘branching points’ in the tree. Each operator has a number of free parameters that are optimized during the training process. TPOT maintains a balance between high performance and low model complexity. As a result, the pipelines learned by TPOT consist of a relatively small number of operators (e.g., in the single-digits) that can still meet or exceed the performance of competing state-of-the-art ML approaches. TPOT’s original fitting process is roughly outlined in Algorithm 1. Recent additions to the TPOT codebase have added Pareto optimization via the NSGA-II algorithm [8], and have migrated the genetic programming steps to use the well-maintained implementation provided by DEAP [7].

An important component of TPOT is its implementation of StackingEstimator components, which allow estimators (i.e., classification or regression models) to propagate their outputs as ‘synthetic features’ to subsequent operators. The stacking estimator takes an earlier operator as its only argument, and feeds all output values from that operator into the subsequent operator as an input vector of synthetic features. This means that TPOT can both (a.) chain estimators in sequence and (b.) concatenate the outputs of multiple operators into a single synthetic feature matrix for another operator, yielding a ‘branching’ pattern of operators. Any number of raw outputs from a single estimator can be passed as inputs to subsequent operators, a useful property that can be leveraged for constructing nonlinear multi-layer NN architectures where adjacent layers can be arbitrarily wide.

**Algorithm 1:** Learning an AutoML pipeline in TPOT. Subroutines are omitted for brevity. Recent versions of TPOT have added Pareto optimization and several other improvements.

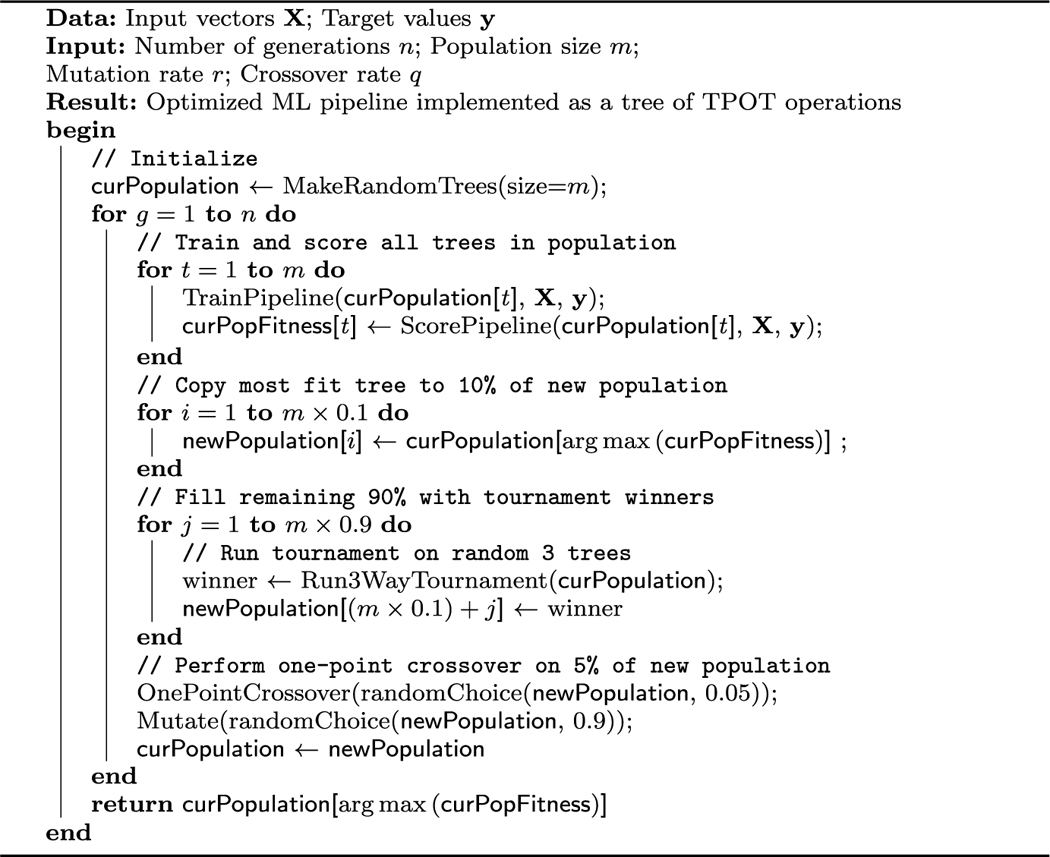

With the exception of our new NN estimators (which are implemented in PyTorch, as described below), all operators are implemented in either Scikit-learn [28] or XGBoost [5], both of which are popular open-source Python-based machine learning libraries. TPOT natively performs cross validation and generates Python scripts that implement the learned pipelines to allow reuse in subsequent analyses. For a more detailed description and evaluations of TPOT, please see [18, 25, 27].

## 3 Methods

**Fig. 1:**
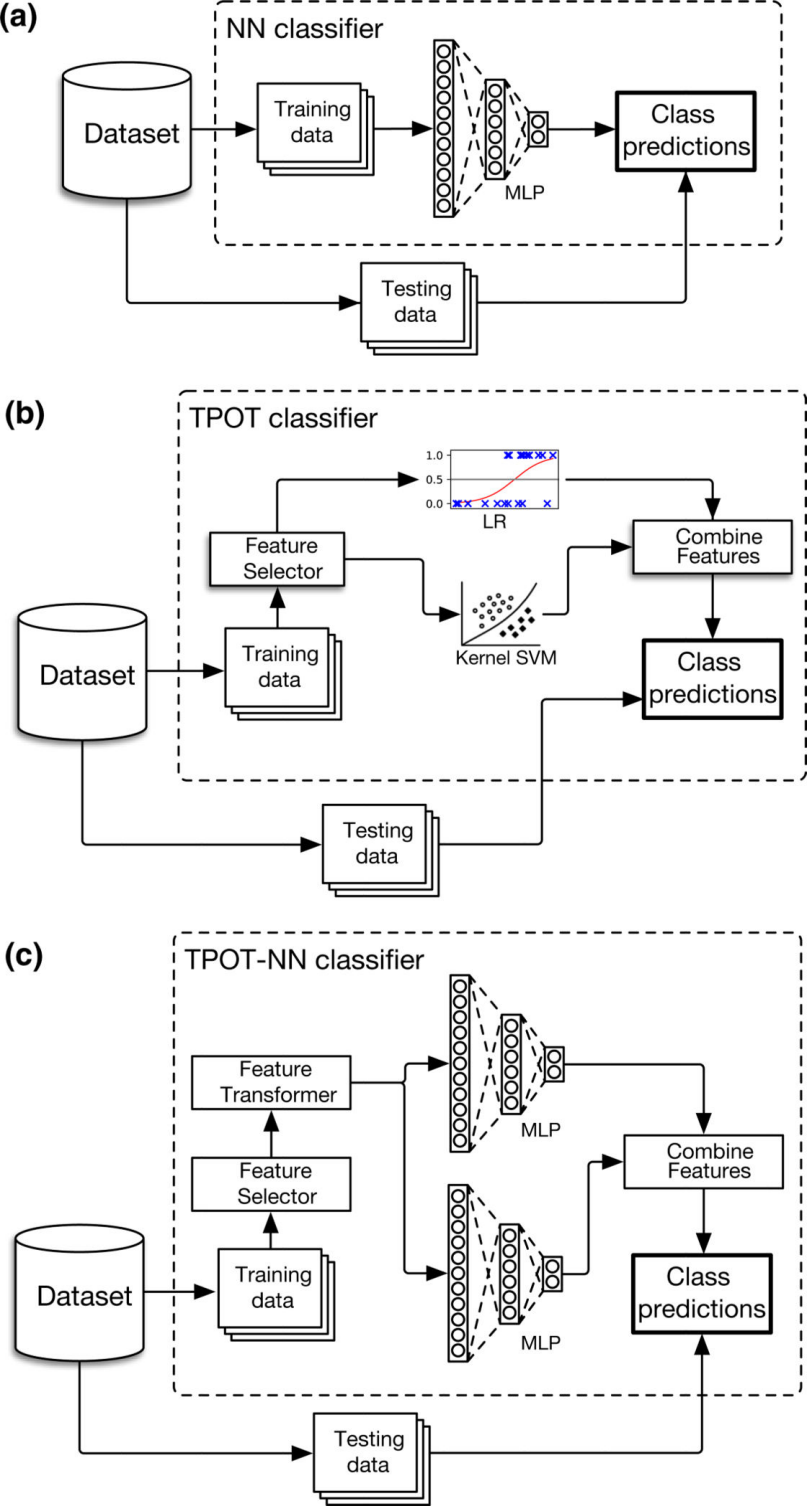
Example pipelines for model configurations used in this study. **a** Stacked NN strategy with no AutoML. **b** Example of a standard (no neural networks) TPOT pipeline containing a logistic regression classifier and a kernel SVM classifier. **c** Example of a TPOT-NN pipeline containing two multilayer perceptron estimators

We designed TPOT-NN as a new feature implemented within the existing TPOT software. Briefly, operators in TPOT are either implemented natively or imported from external Python packages (mainly from Scikit Learn and XGBoost). Each operator takes zero or more tunable hyperparameters that TPOT can optimize during the learning process (an example is given above, in Sect. 2.2) (Fig. 1).

Since a non-NN LR model (implemented as part of Scikit-Learn) is included in standard TPOT, we are able to directly compare the two LR implementations to validate that the PyTorch models are compatible with the TPOT framework, and to quantify the performance variation due to differences in the internal implementations of equivalent models.

### 3.1 TPOT-NN

Our initial release of TPOT-NN adds two new classification estimators that are available to TPOT while learning pipelines: a logistic regression classifier (LR), and a multilayer perceptron classifier (MLP)—both of which are implemented as artificial neural network models constructed in PyTorch. TPOT uses GP to optimize several metaparameters of these LR and MLP estimators that are commonly tuned either manually or via a brute-force architecture search, including the dimensionality of intermediate network layers, the specific convex optimization algorithm used in training, learning rate, and several others. The dimensionality of intermediate layers in TPOT-NN estimators is optimized by GP, but the available options in a given experiment are based on the number of features in the input dataset—although this places a limit on the estimation capacity of individual layers, it prevents TPOT from building massive networks that would be intractable for learning in the context of TPOT’s GP algorithm. LR and MLP are considered two of the simplest neural network architectures, and are therefore suitable for this initial analysis of how neural network estimators behave when integrated into TPOT. Since MLPs add depth to LR’s shallow architecture, this allows us to control for two sources of variation:

1. Changes in TPOT’s performance due solely to the inclusion of ANN estimators (e.g., baseline TPOT vs. TPOT-NN’s LR estimator).
2. Changes in performance due to the adoption of multi-layer ANN estimators within the TPOT pipelines (e.g., TPOT-NN’s MLP estimator vs. all other TPOT configurations).

TPOT users can control the set of available operators—as well as the trainable parameters and the values they can assume—by providing a ‘configuration dictionary’ (a default configuration dictionary is used if the user does not provide one). In the experiments described below, we use this functionality to selectively restrict TPOT’s estimators to characterize these two sources of variation. Briefly, we sought to determine performance differences between 4 experimental scenarios (both in terms of final classifier pipeline performance and in the time efficiency of learning the final pipeline), as described in Table 2.

Although TPOT-NN currently only adds estimators written in PyTorch, we designed TPOT-NN’s architecture to be easily extended to estimators implemented in other neural computing frameworks. Since TPOT requires that all estimators implement an identical public interface (compatible with the scikit-learn API), we wrapped the PyTorch models in classes that implement the necessary methods. Users interested in contributing additional NN estimators can find detailed documentation for how to do so on the main TPOT website.

### 3.2 Dataset descriptions

We evaluated TPOT-NN on its ability to classify data from 6 diverse, well-studied publicly available datasets with binary targets. All 6 of the datasets have been used in previous evaluations of TPOT, and are easily accessible from both the UCI Machine Learning Repository [9] and from the Penn Machine Learning Benchmarks (PMLB) dataset repository [26]. The dataset names—along with summary characteristics—are listed in Table 1. We used the PMLB Python package to retrieve the datasets and their associated target variables. Hill_Valley_with_noise and Hill_Valley_without_noise both contain synthetic data; the others contain real (non-synthetic) data. The number of data points, data types for each feature (i.e., binary, integer, or floating-point decimal), number of features, and number of target classes in each dataset are variable.

spambase [9] contains features extracted from email text, including frequencies of 48 informative words, 6 character frequencies, and several others, where the target is to predict whether an email is ‘spam’ or ‘not spam’. The ionosphere dataset [33] contains continuous measurements where features are radar measurements of the atmosphere at different frequencies, and the classification task is to differentiate ‘good’ radar readings from ‘bad’ radar readings, where ‘bad’ readings are ones that pass through the atmosphere without returning useful information. Notably, ionosphere was specifically curated for use with neural network classifiers. breast-cancer-wisconsin [34] contains descriptive features (texture, area, concavity, etc.) of cell nuclei aspirated from human breast tissue masses, with the prediction target being ‘malignant’ versus ‘benign.’ mushroom [16] is comprised of visible characteristics (gill color, cap shape, etc.) of mushrooms, as well as several features describing growing habitat, with the target being differentiation of edible versus poisonous mushroom specimens. Finally the two Hill_Valley datasets [9] are synthetic representations of ‘hills’ and ‘valleys’ (with and without added noise) consisting of 100 ordered coordinates on a two-dimensional graph, where the task is to distinguish hills from valleys.

For further details on the 6 datasets, we have included dataset profiling reports (using the Pandas Profiling package for Python), which are included in Supplemental Materials. These reports detail additional characteristics—such as feature descriptions, case/control ratios, correlation structures, prevalence of missing values and zeros, and others—as well as sample rows from the datasets.

### 3.3 Experimental setup

We performed 720 TPOT experiments in total, corresponding to 30 repetitions for each of the 6 datasets (described previously, and in Table 1) on each of the the 4 configurations listed in Table 2. We used an 80%/20% train/test split on the datasets and scored pipelines based on classification accuracy with 5-fold cross-validation. Across all experiments, we allowed TPOT to train to completion by terminating training after 35 generations with no improvement to the Pareto front of the best pipelines, and each generation contained 100 individual pipeline trees. Of the 4 configurations, tpot-base and tpot-all are meant to highlight performance differences that occur when NN estimators are added to TPOT (tpot-all includes the new NN estimators). The tpot-lr and tpot-mlp configurations limit TPOT to only use PyTorchLRClassifier and PyTorchMLPClassifier estimators (respectively, in addition to the feature selectors and feature transformers available in all configurations), and are meant to explore the performance capabilities of those estimators alone, as well as whether TPOT is capable of automatically constructing deeper configurations of smaller NN building blocks.

### 3.4 Hardware and high-performance computing environment

All experiments were run on a high-performance computing (HPC) cluster at the University of Pennsylvania. Each experiment was run on a compute node with 48 available CPU cores and 256 GB of RAM. Job scheduling was managed using IBM’s Platform Load Sharing Facility (LSF). All experiments involving PyTorch neural network estimators were run on nodes equipped with N VIDIA® TITAN GPUs, and using a version of PyTorch compiled with support for CUDA GPU acceleration.

## 4 Results

We evaluated the pipelines’ performance based on two metrics: classification accuracy of the trained pipeline and the elapsed time to train the pipeline.

**Fig. 2:**
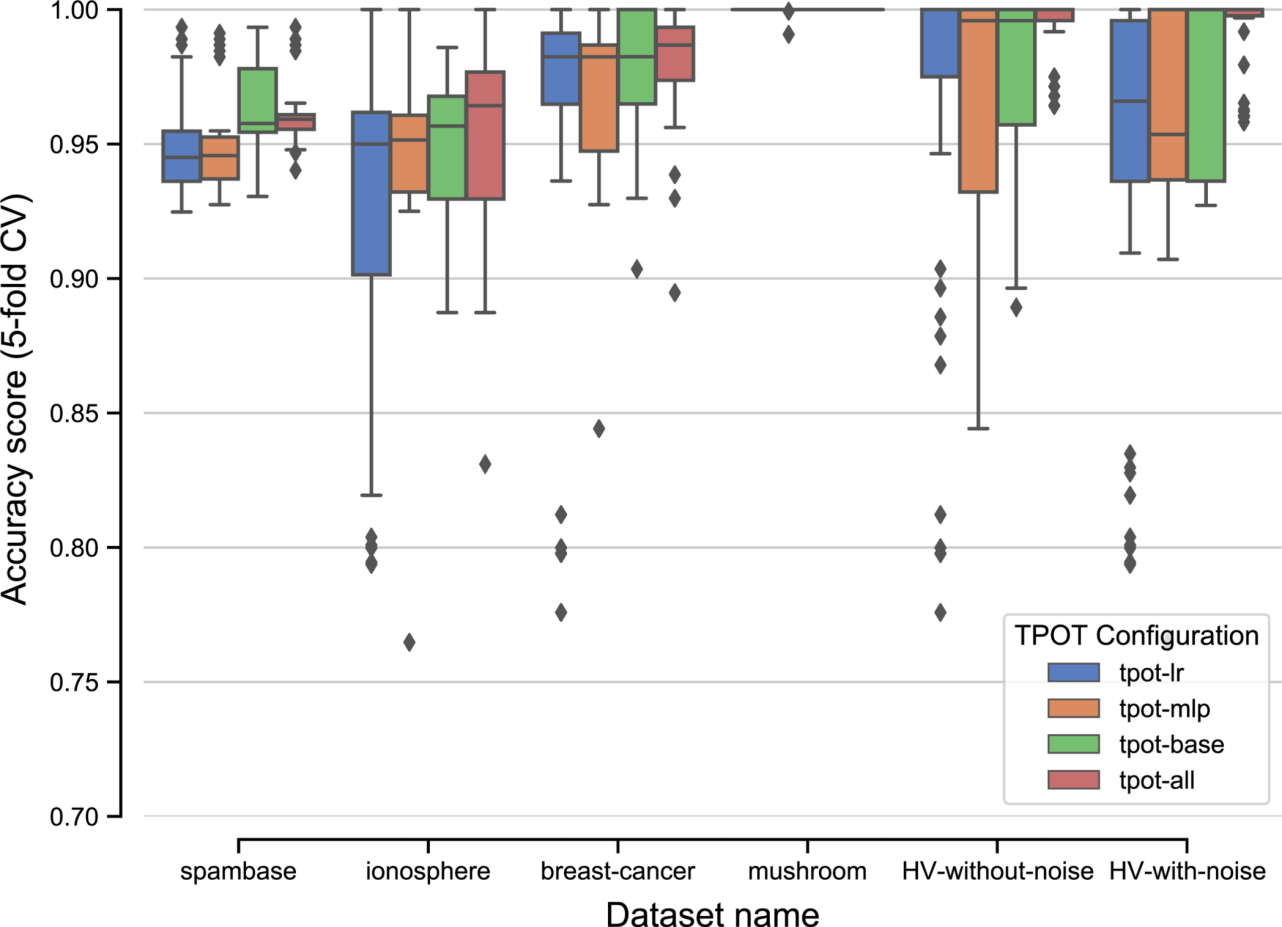
Distributions of accuracy scores for TPOT deployed in various configurations on 6 well-studied public datasets. Each distribution consists of 30 experiments using the same initial TPOT configuration on the same dataset

The results of running TPOT in the 4 configurations (described in Sect. 3.3) on the 6 previously described datasets are shown in Fig. 2 and Table 3. Along with mean classification accuracy, we also report 2-tailed *p*-values for two separate hypothesis tests performed on “base” TPOT (tpot-base) and TPOT-NN (tpot-all) experiments: the independent samples *t*-test on means, used to determine whether there is a significant difference in classification accuracy; and Levene’s test, used to determine whether there is a significant difference in variance of the accuracy scores [4].

### 4.1 Base TPOT vs. TPOT with PyTorch neural network estimators

TPOT-NN yields significantly greater classification accuracy on two of the datasets: HV-without-noise and HV-with-noise. Notably, TPOT-NN performed at least as well as base TPOT on all 6 datasets, in spite of the improvement not being statistically significant on 4 of those datasets. These observations are consistent with the principles of GP-based AutoML—namely, that adding new operators should not result in decreased performance, since evolution will select for the best pipelines using the available set of operators, ignoring those that would yield worse performance.

Interestingly, we also observe that TPOT-NN significantly reduces variance in classification accuracy in 3 of the datasets (HV-without-noise, HV-with-noise, and ionosphere), which suggests that when TPOT is used with the new PyTorch neural network estimators, the learned pipelines may perform more consistently than otherwise, even in the absence of significantly improved accuracy.

In our experiments, TPOT yielded perfect classification accuracy across all 4 configurations when applied to the mushroom dataset, suggesting that the two target classes (whether a mushroom is poisonous or edible) can be easily distinguished by many types of ML pipelines.

### 4.2 Single- vs. multi-layer NN estimators in TPOT-NN

In addition to exploring the effect of adding NN models to TPOT (as described in the previous section), we also explored the effect of introducing multi-layer versus single-layer NN estimators in a GP context. The two corresponding TPOT configurations—tpot-lr and tpot-mlp—yielded pipelines consisting of PyTorch LR estimators and MLP estimators (respectively), plus TPOT’s feature selectors and feature transformers (in other words, removing all non-NN estimators from TPOT’s operator pool). Since TPOT pipelines can contain multiple estimators, these pipelines often consist of multiple NN estimators arranged in parallel and/or serial, which theoretically allows them to become arbitrarily deep and arbitrarily wide, depending on TPOT’s evolutionary algorithm. However, the model simplicity constraint introduced via Pareto optimization encourages learning simpler (shallower) models in the absence of significant performance differences, so it is unlikely for TPOT-NN to learn pipelines with very high depth.

The results in Fig. 2 and Table 3 show that tpot-lr and tpot-mlp perform nearly identically across most datasets, with no statistically significant differences in mean accuracy. However, the average number of PytorchLRClassifier estimators in a tpot-lr pipeline is noticeably greater than the average number of PytorchMLPClassifier estimators in a tpot-mlp pipeline (mean 3.04 vs. 2.49; 2-tailed *t*-test *p*= 0.01 ). This can be interpreted as TPOT automatically using multiple LR estimators to achieve a certain depth, which equivalently is accomplished using a smaller number of MLP estimators. In other words, TPOT automatically replicates the well-known observation that greater estimation capacity can be achieved by increasing either the width or depth of a given neural network model.

### 4.3 Time efficiency of TPOT-NN

**Fig. 3:**
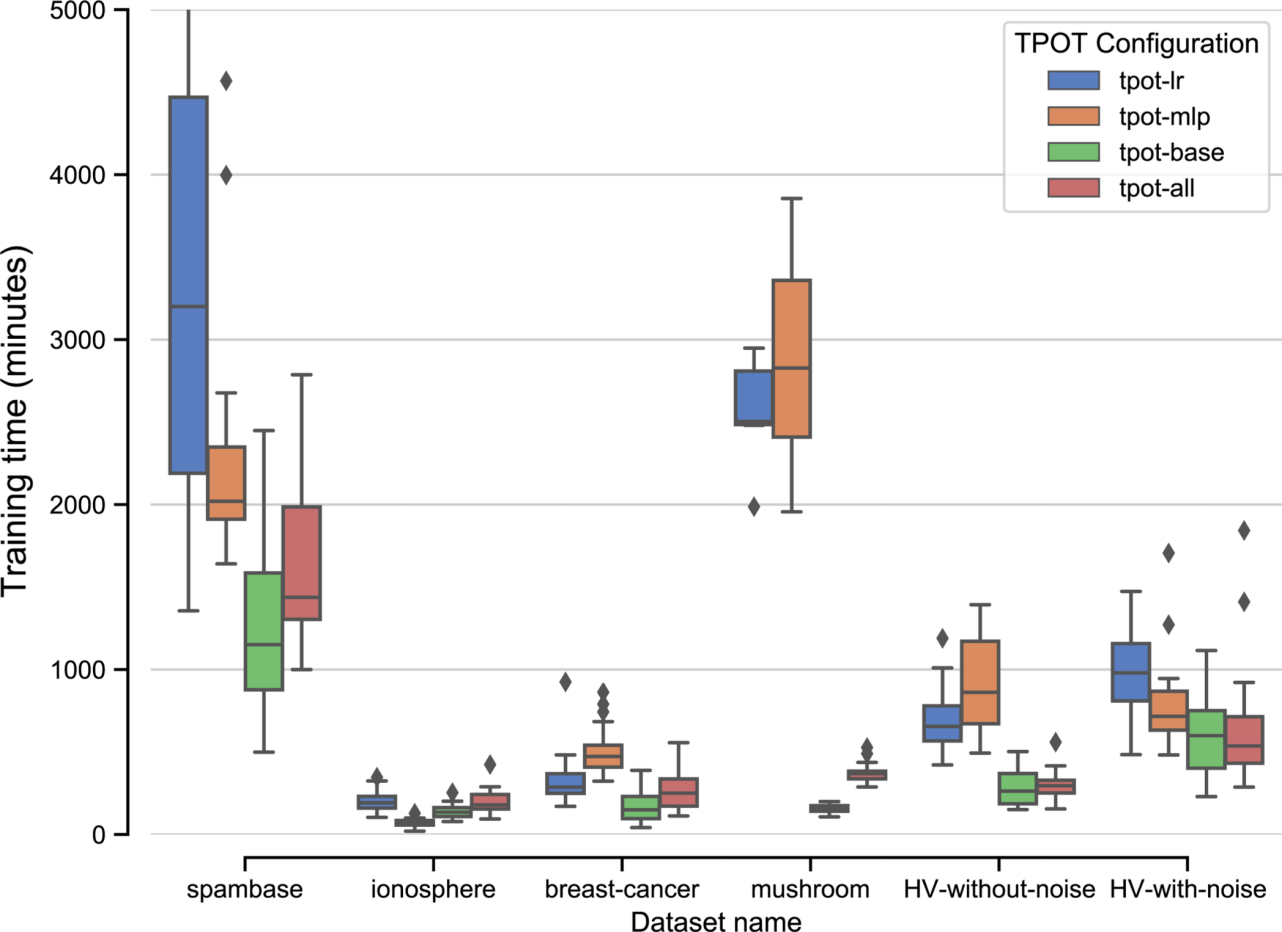
CPU clock time distributions for training TPOT on each of the 6 evaluation datasets. In most cases, TPOT configurations containing PyTorch neural network estimators require longer to train than “base” TPOT configurations, with the overall effect scaling proportionally with the size of the dataset

Neural network models—in general—are known to require substantially more computational power to train than traditional, shallow ML models, largely due to the number of tunable free parameters and the complexity of optimizing over gradients that span multiple layers. As expected, TPOT pipelines that include NN estimators correspondingly take longer to train than pipelines learned by base TPOT. Fig. 3 and Table 4 show the training time distributions for the previously described TPOT/TPOT-NN experiments. For most datasets, TPOT pipelines that use NN models exclusively (tpot-lr and tpot-mlp) require noticeably more time to train than tpot-all pipelines that contain a mixture of NN and non-NN estimators, which in turn take longer to train than tpot-base pipelines that only contain non-NN estimators.

This pattern seems to disappear when TPOT is trained on the ionosphere dataset which, at only 351 samples, is the smallest dataset tested in our experiments. Anecdotally, this is likely attributable to the fact that the increased time to train NN models ends up being eclipsed by other computational tasks when the dataset is small, such as TPOT having to select from a larger pool of potential operators, as well as the computational overhead inherent to creating and evaluating Scikit-Learn and XGBoost estimators.

### 4.4 Structural topologies of pipelines learned by GP

TPOT assembles pipelines that consist of multiple operators—possibly including multiple classifiers or regressors in addition to feature selectors and feature transformers—to achieve better performance than individual machine learning estimators [25]. Since the estimation capacity of simple feedforward neural networks monotonically increases with added network depth, we sought to determine whether TPOT will automatically construct deeper architectures by stacking several NN estimators in the absence of *a priori* instruction to do so. Although we would expect a naïve AutoML system to do this in most cases, TPOT’s multi-objective optimization balances model performance against model simplicity, and therefore adding more NN estimators to a pipeline needs to be ‘justified’ by a substantial boost in classification performance.

As described earlier, TPOT’s StackingEstimator concatenates the outputs of earlier operators together as a set of synthetic features for subsequent operators. Currently, TPOT-NN’s LR and MLP estimators are designed such that they will output the raw values of their final fully-connected layers when occurring in the middle of a pipeline (i.e., when wrapped in a stacking estimator), but instead feed the raw values through an output layer yielding a class prediction when the estimator is the final step in the pipeline.

The one major challenge/caveat exposed by this design is that adjacent NN estimators are “decoupled” during optimization: Since they are implemented as separate PyTorch networks, gradients cannot be propagated across the entire architecture, leading to reduced computational efficiency.

When we force TPOT-NN to build pipelines comprised only of feature selectors, feature transformers, and logistic regression estimators, it did indeed construct pipelines consisting of stacked arrangements of logistic layers that bear a resemblance to segments of well-known DL models. The following Python code is the output of one of these, selected at random from the pool of LR-only TPOT-NN pipelines (hyperparameters have been removed for readability):


# Average CV score on the training set was: 0.9406477266781772
      exported_pipeline = make_pipeline(
        make_union(
          StackingEstimator(estimator=make_pipeline(
            StackingEstimator(estimator=PytorchLRClassifier(...)), # LR1
            StackingEstimator(estimator=PytorchLRClassifier(...)), # LR2
            PytorchLRClassifier(...) # LR3
          )),
          FunctionTransformer(copy) # Identity (skip connection)
        ),
        PytorchLRClassifier(...) # LR4
      )


**Fig. 4:**
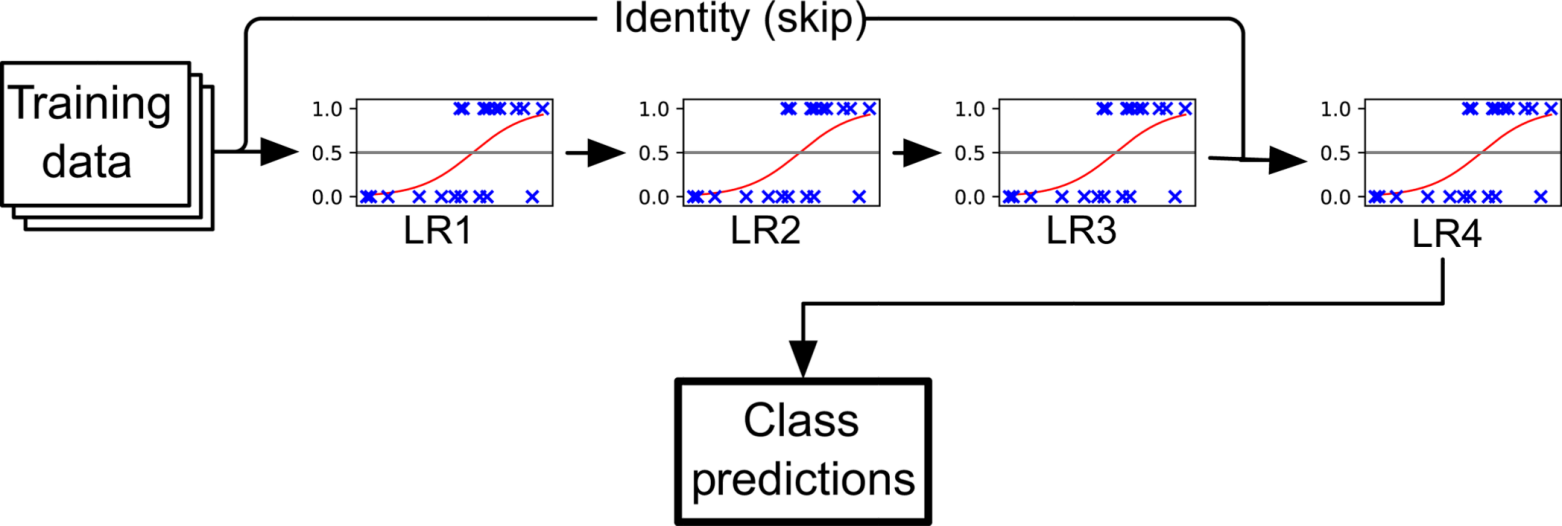
Randomly selected pipeline learned when restricting TPOT’s pool of estimators to logistic regression classifiers only. Some redundant components, such as make_pipeline function calls, are omitted. Notably, the structure of this pipeline resembles one of the key components of the popular ResNet architecture, which suggests that other motifs learned by TPOT-NN may be possible to expand into deeper architectures

This pipeline is replicated graphically in Fig. 4. Notably, this pipeline strongly resembles a key building block of the world record-holding ResNet image recognition architecture [14]. Specifically, the “skip connection” segment (implemented in TPOT by splicing a second copy of the training data into a second leaf node in the pipeline) behaves similarly to “shortcut connections” found in ResNet, which essentially allow signals in the input data to be directly utilized in later neural network layers of the model. The mathematical properties of these skip connections have been known at least since 1989, when they were known as the cascade-correlation architecture [11], underscoring their influence on the larger field of AI. This suggests that AutoML could be used as a tool for identifying new submodules for larger NN-based models. We discuss this possibility further in Sect. 5.4.

## 5 Discussion

### 5.1 Compatibility of neural network estimators with TPOT

The two NN estimators we have currently designed for TPOT (PyTorchLRClassifier and PyTorchMLPClassifier) integrate without issue into the TPOT workflow. Due to the frequently increased training time of TPOT-NN pipelines versus base TPOT pipelines, the NN estimators are not enabled by default, but can be enabled by passing the parameter config_dict=‘TPOT NN’ when initializing TPOT.

Overall, TPOT has not been tested extensively on many specialized classification tasks (such as image classification, text classification, and others), which comprise an important topic that we have prioritized for future exploration. However, the inclusion of NN estimators is an important step in bridging this gap—neural networks implemented in PyTorch and other neural computing libraries have proven to be incredibly flexible for a vast number of applications, which opens many exciting opportunities for expanding TPOT and other AutoML tools.

This, however, points to one of the major challenges in developing NN estimators for TPOT: While most shallow estimators can be included in TPOT simply by referring to their modules in Scikit-Learn or XGBoost, TPOT-NN estimators need to be implemented ‘from scratch’ in PyTorch (or another neural computing library). Once these are written and incorporated into TPOT’s codebase, an appropriate set of tunable metaparameters also need to be defined. For most non-NN estimators, this is as simple as enumerating the possible arguments provided by their source libraries, but for NN estimators it can include complex dynamic characteristics that are highly responsive to the underlying dataset, like the number of hidden layers, layer width, multiple activation functions, and dropout rates, among many others.

Nevertheless, the fact that TPOT is supported by contributions from the open-source community—as well as the continued development of more streamlined neural computing interfaces (such as Keras)—suggests that these barriers will prove less challenging to handle in the future. The examples we show in this study illustrate the early potential of TPOT-NN and demonstrate how it behaves in comparison to base TPOT.

### 5.2 TPOT-NN significantly improves classification accuracy and reduces variance, but only for some datasets

A major criticism of neural networks and deep learning is that it has often been unfairly touted as a “magic bullet” that is ideal for solving most problems in AI. Recent research does a good job acknowledging that this is more nuanced than originally thought [31], and that shallow learners actually outperform deep learning in many cases, in spite of deep models in theory being universal approximators. We observed that the TPOT-NN performs substantially better than non-NN TPOT on 2 of the 6 datasets we tested—HV-with-noise and HV-without-noise—and we have not found a situation when it performs worse in terms of classification accuracy when compared to base TPOT.

Of possibly equal importance, we observed that repeated experiments using TPOT-NN yield more consistent results (lower variance in classification accuracy). This effect was statistically significant in 3 of 6 datasets (HV-with-noise, HV-without-noise, and ionosphere), but the observed variance measurements were smaller using TPOT-NN versus base TPOT in all 6 datasets. This observation suggests that TPOT-NN (and similar tools, in the future) could be used to improve the reproducibility of ML analyses. As this result was unexpected, we intend to explore this phenomenon more comprehensively in future studies.

This highlights one of the chief strengths of AutoML, and one of the major motivations for developing TPOT-NN: Neural network models clearly are advantageous for certain classification tasks performed on certain datasets, but simpler shallow models might work better on other datasets, including smaller datasets like those tested in this study. Further still, TPOT pipelines that incorporate both NN and non-NN estimators with different optimization objectives have the potential to outperform simpler pipelines containing only one estimator, especially when datasets contain complex sets of features made up of different data types. Finally, the inclusion of feature transformer and feature selector operators in TPOT adds model introspection capabilities to experiments that use ANNs.

### 5.3 Assessing the tradeoff between model performance and training efficiency

The amount of time needed to train a pipeline is an important pragmatic consideration in real-world applications of ML. This certainly extends to the case of AutoML: The parameters we use for TPOT include somewhere between 50 and 100 training generations with a population size of 100 in each generation, meaning that we evaluate several thousand candidate pipelines—each of which consists of a variable number of independently optimizable operators—for every experiment (of which there were 720 in the present study). As shown in Table 4, we generally expect a non-NN pipeline to train in the range of several hours to slightly over 1 day, depending on the dataset.

Our relatively simple NN estimators sit at the lower end (complexity-wise) of components used to build DL architectures, and likewise are among the simplest to train. Regardless, using either the LR or MLP PyTorch estimators in a TPOT experiment can cause the average training time to increase significantly—in our experiments, the average training time on the mushroom dataset increased by 18-fold when comparing the tpot-base to tpot-mlp configurations, and the datasets we used are smaller than those used in most DL applications, which can have millions of datapoints comprised of thousands of features each [35]. Users will have to determine, on an individual basis and dependent on the use case, whether the potential accuracy increase of at most several percentage points is worth the additional time and computational investment inherent to ANNs.

Nonetheless, our results make it clear that it is unlikely for a TPOT-NN pipeline to perform *worse* than a (non-NN) TPOT pipeline. In ‘mission critical’ settings where training time is not a major concern, TPOT-NN can be expected to perform *at least* as well as standard TPOT. Furthermore, the surprising observation that TPOT-NN seems to yield pipelines with less variance in their classification accuracy suggests that TPOT-NN’s new estimators may make the results of experiments more reliable and reproducible. However, this claim needs to be tested on additional datasets and explored further before any definitive conclusions can be made.

### 5.4 AutoML as a tool to discover novel neural network architectures

Based on the results we describe in Sect. 4.4, AutoML (and TPOT-NN, in particular) may be useful for discovering new neural network “motifs” to be composed into larger networks. For example, by repeating the internal architecture shown in Fig. 4 to a final depth of 152 hidden layers, converting the fully connected layers to convolutional layers, and adjusting the number of nodes in those layers, the result is highly similar to the version of ResNet that won first place in 5 categories at two major image recognition competitions in 2015 [14]. In the near future, we plan to investigate whether this phenomenon could be scaled into a larger, fully data-driven approach for generating modular neural network motifs that can be composed into models effective for a myriad of learning tasks.

However, there are two main challenges that need to be addressed before TPOT can automatically learn models of substantially greater depth. First, new strategies for efficiently learning pipelines using large training datasets need to be implemented. As it currently stands, TPOT pipelines become computationally infeasible to learn in a reasonable amount of time (and with reasonable computational resources) when datasets reach tens of thousands of samples, which is substantially smaller than many of the popular datasets used to train highly performant DL models. Second, TPOT penalizes larger pipelines in favor of smaller (and more interpretable) pipelines. Since increasing depth would result in larger pipelines, TPOT-NN would need to compensate for this penalty somehow. Both of these challenges are currently on the roadmap of tasks to address for TPOT in the near future.

### 5.5 Future work on integrating AutoML and ANNs

Since one of our primary goals in this work was to provide a baseline for future development of neural network models in the context of AutoML, the two PyTorch models we have currently built (logistic regression and multilayer perceptron) are structurally simple. Future work on TPOT-NN will allow expansion of its functionality to improve the capabilities of the existing models as well as incorporate other, more complex architectures, such as convolutional neural networks, recurrent neural networks, and other applications of deep learning. Additionally, we will be adding support for NN-based regression.

In implementing these neural network estimators, we also intend to evaluate and improve TPOT for use with other, more complex types datatypes, including images, text, graph data, and others, which have all played important roles in the success of ANNs and modern applications of AI. However, by first evaluating TPOT-NN on simple binary classification datasets made up of regularly structured, pre-extracted features, we have layed a strong foundation for future development in many exciting directions.

## 6 Conclusions

AutoML and ANNs are immensely useful tools for approaching a wide variety of inductive learning tasks, and it is clear that both hold strengths and weaknesses for specific use cases. Rather than viewing them as *competing* methods, we instead propose that the two can work synergistically: For at least the cases we explored in this study (binary classification on 6 well-characterized datasets with regularly structured inputs), the addition of simple NN blocks into the pool of available estimators improves classification accuracy in comparison to non-NN AutoML. Anecdotally, our observations also suggest that the objective nature of learning pipelines containing NN estimators via GP may allow for the discovery of new NN ‘motifs’ that can be expanded into deep learning pipelines that achieve very high performance. Since TPOT-NN’s learned pipelines often explicitly include feature selection and feature transformation operators, they also provide a feasible mechanism for improving interpretability of models that contain NN components arranged in a deep configuration.

Currently, use of these NN estimators increases the training time for TPOT pipelines, which may limit their usefulness in some situations. As we continue to improve TPOT in subsequent releases, we plan to explore various strategies for improving the training time, such as techniques for performing parallel GPU optimization of adjacent PyTorch neural network models within the same pipeline. Nonetheless, our results suggest a multitude of novel directions for methodological research in machine learning and artificial intelligence. TPOT-NN serves as both an early case study as well as a platform to facilitate AutoML+NN research in a reproducible, transparent manner that is open to the scientific community.

## Supplementary Material

Supplementary Material 1

## Figures and Tables

**Table 1:** 6 datasets used to evaluate TPOT-NN

Dataset name	*n*	Number of features	Data type	Real/synthetic
spambase	4601	57	Mixed	Real
ionosphere	351	34	Mixed	Real
breast-cancer-wisconsin	569	30	Float	Real
mushroom	8124	22	Mixed	Real
Hill_Valley_with_noise	1212	100	Float	Synthetic
Hill_Valley_without_noise	1212	100	Float	Synthetic

Names are identical to corresponding labels used to denote the dataset in the PMLB Python library

**Table 2:** Configurations tested for evaluating TPOT-NN

Configuration	Description of estimators	TPOT-NN?
tpot-lr	TPOT restricted to only use TPOT-NN’s LR estimator for classification	Yes
tpot-mlp	TPOT restricted to only use TPOT-NN’s MLP estimator for classification	Yes
tpot-base	Baseline TPOT (no TPOT-NN estimators)	No
tpot-all	TPOT with all estimators (including TPOT-NN)	Yes

By comparing performance of TPOT in each of the 4 configurations, we can determine the effects of adding PyTorch neural network estimators to TPOT, as well as the effect of adding multi-layer (i.e., MLP) neural network estimators

**Table 3:** Characteristics of TPOT deployed in various configurations on 6 public datasets

Dataset	Accuracy by TPOT configuration				2-tailed *p*-value	
		
	tpot-lr	tpot-mlp	tpot-base	tpot-all	*t*-test	Levene
spambase	0.961	0.955	0.964	0.960	0.40	0.43
ionosphere	0.921	0.828	0.948	0.947	0.91	**0.04**
breast-cancer	0.959	0.909	0.979	0.975	0.54	0.85
mushroom	1.000	1.000	1.000	1.000	–	–
HV-without-noise	0.976	0.977	0.977	0.994	**< 0.01**	**< 0.01**
HV-with-noise	0.968	0.970	0.976	0.989	**0.05**	**0.05**

Values for hypothesis tests are *p*-values, shown in bold font when statistically significant (*α* = 0.05)

**Table 4:** Training time statistics for 6 datasets across 4 TPOT configurations

Dataset	Mean training time (minutes)				*t*-test
		
	tpot-lr	tpot-mlp	tpot-base	tpot-all	(base v. all)
spambase	3473	2339	1296	1634	**0.03**
ionosphere	201	166	139	197	**< 0.01**
breast-cancer	324	507	174	272	**0.01**
mushroom	2555	2872	157	372	**< 0.01**
HV-without-noise	690	876	287	295	0.78
HV-with-noise	988	816	589	652	0.48

2-sided *p*-values are given for the difference in means between tpot-base and tpot-all, with statistically significant results highlighted in bold (*α* = 0.05)
