# Supplementary material for: TPOT-NN: augmenting tree-based automated machine learning with neural network estimators
Source: Genet Program Evolvable Mach. Author manuscript; Available in PMC 2025 Aug 6. (PMC12327408; doi:10.1007/s10710-021-09401-z)
Supplement: Supplementary Material 1 [file NIHMS2035489-supplement-Supplementary_Material_1.zip › New folder/breast_cancer_wisconsin.html]

 
 
          breast_cancer_wisconsin            Toggle navigation         breast_cancer_wisconsin      Overview    Variables    Interactions    Correlations    Missing values    Sample            Overview       Overview    Reproduction    Warnings  9        Dataset statistics     Number of variables  20    Number of observations  569    Missing cells  0    Missing cells (%)  0.0%    Duplicate rows  0    Duplicate rows (%)  0.0%    Total size in memory  89.0 KiB    Average record size in memory  160.2 B       Variable types     NUM  19    BOOL  1        Reproduction     Analysis started  2020-08-25 01:09:51.782056    Analysis finished  2020-08-25 01:10:47.052389    Duration  55.27 seconds    Version   pandas-profiling v2.8.0     Command line   pandas_profiling --config_file config.yaml [YOUR_FILE.csv]     Download configuration   config.yaml        Warnings       2   is highly correlated with  25  and  2 other fields    High correlation       25   is highly correlated with  2  and  2 other fields    High correlation       15   is highly correlated with  14    High correlation       14   is highly correlated with  15    High correlation       24   is highly correlated with  25  and  2 other fields    High correlation       4   is highly correlated with  25  and  2 other fields    High correlation       29   has 13 (2.3%) zeros    Zeros       19   has 13 (2.3%) zeros    Zeros       18   has 13 (2.3%) zeros    Zeros             Variables          29   Real number (ℝ ≥0 )   ZEROS          Distinct count  492    Unique (%)  86.5%    Missing  0    Missing (%)  0.0%    Infinite  0    Infinite (%)  0.0%          Mean  0.11460622319859401    Minimum  0.0    Maximum  0.29100000000000004    Zeros  13    Zeros (%)  2.3%    Memory size  4.6 KiB              2020-08-25T01:10:47.100023  image/svg+xml    Matplotlib v3.3.1, https://matplotlib.org/                                                                                                                                                           Toggle details       Statistics    Histogram(s)    Common values    Extreme values       Quantile statistics     Minimum  0    5-th percentile  0.024286    Q1  0.06493    median  0.09993    Q3  0.1614    95-th percentile  0.23692    Maximum  0.291    Range  0.291    Interquartile range (IQR)  0.09647       Descriptive statistics     Standard deviation  0.0657323412    Coefficient of variation (CV)  0.5735494929    Kurtosis  -0.5355351225    Mean  0.1146062232    Median Absolute Deviation (MAD)  0.04457    Skewness  0.4926155269    Sum  65.210941    Variance  0.004320740679           Histogram             2020-08-25T01:10:47.214858  image/svg+xml    Matplotlib v3.3.1, https://matplotlib.org/                                                                                                                                                                                                                                                                                                                                              Histogram with fixed size bins  (bins=10)           Value  Count  Frequency (%)         0  13  2.3%         0.1218  3  0.5%         0.1708  3  0.5%         0.02564  3  0.5%         0.04306  3  0.5%         0.1105  3  0.5%         0.1827  3  0.5%         0.06296  3  0.5%         0.07431  3  0.5%         0.05556  3  0.5%         0.02381  2  0.4%         0.05882  2  0.4%         0.0914  2  0.4%         0.1099  2  0.4%         0.02579  2  0.4%         0.1772  2  0.4%         0.03846  2  0.4%         0.2475  2  0.4%         0.0875  2  0.4%         0.0589  2  0.4%         0.06136  2  0.4%         0.1056  2  0.4%         0.01111  2  0.4%         0.1155  2  0.4%         0.1205  2  0.4%         Other values (467)  499  87.7%               Minimum 5 values    Maximum 5 values         Value  Count  Frequency (%)         0  13  2.3%         0.008772  1  0.2%         0.009259  1  0.2%         0.01042  1  0.2%         0.01111  2  0.4%         0.01389  1  0.2%         0.01635  1  0.2%         0.01667  1  0.2%         0.01852  1  0.2%         0.02022  1  0.2%               Value  Count  Frequency (%)         0.291  1  0.2%         0.2903  1  0.2%         0.2867  1  0.2%         0.2756  1  0.2%         0.2733  1  0.2%         0.2701  1  0.2%         0.2688  1  0.2%         0.2685  1  0.2%         0.2654  1  0.2%         0.265  1  0.2%                         17   Real number (ℝ ≥0 )          Distinct count  541    Unique (%)  95.1%    Missing  0    Missing (%)  0.0%    Infinite  0    Infinite (%)  0.0%          Mean  0.025478138840070295    Minimum  0.002252    Maximum  0.1354    Zeros  0    Zeros (%)  0.0%    Memory size  4.6 KiB              2020-08-25T01:10:47.343223  image/svg+xml    Matplotlib v3.3.1, https://matplotlib.org/                                                                                                                                                 Toggle details       Statistics    Histogram(s)    Common values    Extreme values       Quantile statistics     Minimum  0.002252    5-th percentile  0.0078922    Q1  0.01308    median  0.02045    Q3  0.03245    95-th percentile  0.060578    Maximum  0.1354    Range  0.133148    Interquartile range (IQR)  0.01937       Descriptive statistics     Standard deviation  0.01790817933    Coefficient of variation (CV)  0.702884125    Kurtosis  5.106252483    Mean  0.02547813884    Median Absolute Deviation (MAD)  0.00876    Skewness  1.90222071    Sum  14.497061    Variance  0.0003207028868           Histogram             2020-08-25T01:10:47.455968  image/svg+xml    Matplotlib v3.3.1, https://matplotlib.org/                                                                                                                                                                                                                                                                                                                                                                                      Histogram with fixed size bins  (bins=10)           Value  Count  Frequency (%)         0.01104  3  0.5%         0.01812  3  0.5%         0.0231  3  0.5%         0.01646  2  0.4%         0.01395  2  0.4%         0.01382  2  0.4%         0.01174  2  0.4%         0.03053  2  0.4%         0.01371  2  0.4%         0.01587  2  0.4%         0.01819  2  0.4%         0.01427  2  0.4%         0.03179  2  0.4%         0.02772  2  0.4%         0.01443  2  0.4%         0.009169  2  0.4%         0.01503  2  0.4%         0.02219  2  0.4%         0.01246  2  0.4%         0.01877  2  0.4%         0.01203  2  0.4%         0.0118  2  0.4%         0.03055  2  0.4%         0.01727  2  0.4%         0.02431  2  0.4%         Other values (516)  516  90.7%               Minimum 5 values    Maximum 5 values         Value  Count  Frequency (%)         0.002252  1  0.2%         0.003012  1  0.2%         0.00371  1  0.2%         0.003746  1  0.2%         0.00466  1  0.2%         0.004693  1  0.2%         0.004711  1  0.2%         0.004883  1  0.2%         0.004899  1  0.2%         0.00493  1  0.2%               Value  Count  Frequency (%)         0.1354  1  0.2%         0.1064  1  0.2%         0.1006  1  0.2%         0.09806  1  0.2%         0.09586  1  0.2%         0.09368  1  0.2%         0.08808  1  0.2%         0.08668  1  0.2%         0.08606  1  0.2%         0.08555  1  0.2%                         25   Real number (ℝ ≥0 )    HIGH CORRELATION           Distinct count  544    Unique (%)  95.6%    Missing  0    Missing (%)  0.0%    Infinite  0    Infinite (%)  0.0%          Mean  880.5831282952548    Minimum  185.2    Maximum  4254.0    Zeros  0    Zeros (%)  0.0%    Memory size  4.6 KiB              2020-08-25T01:10:47.585604  image/svg+xml    Matplotlib v3.3.1, https://matplotlib.org/                                                                                                                                         Toggle details       Statistics    Histogram(s)    Common values    Extreme values       Quantile statistics     Minimum  185.2    5-th percentile  331.06    Q1  515.3    median  686.5    Q3  1084    95-th percentile  2009.6    Maximum  4254    Range  4068.8    Interquartile range (IQR)  568.7       Descriptive statistics     Standard deviation  569.3569927    Coefficient of variation (CV)  0.6465681369    Kurtosis  4.396394829    Mean  880.5831283    Median Absolute Deviation (MAD)  215.6    Skewness  1.859373272    Sum  501051.8    Variance  324167.3851           Histogram             2020-08-25T01:10:47.691614  image/svg+xml    Matplotlib v3.3.1, https://matplotlib.org/                                                                                                                                                                                                                                                                                                                                      Histogram with fixed size bins  (bins=10)           Value  Count  Frequency (%)         733.5  2  0.4%         808.9  2  0.4%         1261  2  0.4%         547.4  2  0.4%         402.8  2  0.4%         1623  2  0.4%         749.9  2  0.4%         1210  2  0.4%         458  2  0.4%         1269  2  0.4%         1603  2  0.4%         698.8  2  0.4%         439.6  2  0.4%         830.5  2  0.4%         284.4  2  0.4%         706  2  0.4%         708.8  2  0.4%         472.4  2  0.4%         546.7  2  0.4%         725.9  2  0.4%         1437  2  0.4%         826.4  2  0.4%         489.5  2  0.4%         1750  2  0.4%         624.1  2  0.4%         Other values (519)  519  91.2%               Minimum 5 values    Maximum 5 values         Value  Count  Frequency (%)         185.2  1  0.2%         223.6  1  0.2%         240.1  1  0.2%         242.2  1  0.2%         248  1  0.2%         249.8  1  0.2%         259.2  1  0.2%         268.6  1  0.2%         270  1  0.2%         273.9  1  0.2%               Value  Count  Frequency (%)         4254  1  0.2%         3432  1  0.2%         3234  1  0.2%         3216  1  0.2%         3143  1  0.2%         2944  1  0.2%         2906  1  0.2%         2782  1  0.2%         2642  1  0.2%         2615  1  0.2%                         19   Real number (ℝ ≥0 )   ZEROS          Distinct count  507    Unique (%)  89.1%    Missing  0    Missing (%)  0.0%    Infinite  0    Infinite (%)  0.0%          Mean  0.011796137082601054    Minimum  0.0    Maximum  0.05279    Zeros  13    Zeros (%)  2.3%    Memory size  4.6 KiB              2020-08-25T01:10:47.811136  image/svg+xml    Matplotlib v3.3.1, https://matplotlib.org/                                                                                                                                                 Toggle details       Statistics    Histogram(s)    Common values    Extreme values       Quantile statistics     Minimum  0    5-th percentile  0.0038308    Q1  0.007638    median  0.01093    Q3  0.01471    95-th percentile  0.022884    Maximum  0.05279    Range  0.05279    Interquartile range (IQR)  0.007072       Descriptive statistics     Standard deviation  0.006170285174    Coefficient of variation (CV)  0.5230767607    Kurtosis  5.126301943    Mean  0.01179613708    Median Absolute Deviation (MAD)  0.003485    Skewness  1.444678145    Sum  6.712002    Variance  3.807241913e-05           Histogram             2020-08-25T01:10:47.912880  image/svg+xml    Matplotlib v3.3.1, https://matplotlib.org/                                                                                                                                                                                                                                                                                                      Histogram with fixed size bins  (bins=10)           Value  Count  Frequency (%)         0  13  2.3%         0.01499  3  0.5%         0.0111  3  0.5%         0.01167  3  0.5%         0.009222  2  0.4%         0.01161  2  0.4%         0.01712  2  0.4%         0.01037  2  0.4%         0.01421  2  0.4%         0.01841  2  0.4%         0.01155  2  0.4%         0.01184  2  0.4%         0.01471  2  0.4%         0.01043  2  0.4%         0.0158  2  0.4%         0.01164  2  0.4%         0.01519  2  0.4%         0.00836  2  0.4%         0.01241  2  0.4%         0.012  2  0.4%         0.01269  2  0.4%         0.005495  2  0.4%         0.01004  2  0.4%         0.01843  2  0.4%         0.009199  2  0.4%         Other values (482)  505  88.8%               Minimum 5 values    Maximum 5 values         Value  Count  Frequency (%)         0  13  2.3%         0.001852  1  0.2%         0.002386  1  0.2%         0.002404  1  0.2%         0.002924  1  0.2%         0.002941  1  0.2%         0.003125  1  0.2%         0.003242  1  0.2%         0.003333  1  0.2%         0.00339  1  0.2%               Value  Count  Frequency (%)         0.05279  1  0.2%         0.0409  1  0.2%         0.03927  1  0.2%         0.03487  1  0.2%         0.03441  1  0.2%         0.03322  1  0.2%         0.03024  1  0.2%         0.02919  1  0.2%         0.02853  1  0.2%         0.02801  1  0.2%                         10   Real number (ℝ ≥0 )          Distinct count  432    Unique (%)  75.9%    Missing  0    Missing (%)  0.0%    Infinite  0    Infinite (%)  0.0%          Mean  0.18116186291739894    Minimum  0.106    Maximum  0.304    Zeros  0    Zeros (%)  0.0%    Memory size  4.6 KiB              2020-08-25T01:10:48.025003  image/svg+xml    Matplotlib v3.3.1, https://matplotlib.org/                                                                                                                                             Toggle details       Statistics    Histogram(s)    Common values    Extreme values       Quantile statistics     Minimum  0.106    5-th percentile  0.1415    Q1  0.1619    median  0.1792    Q3  0.1957    95-th percentile  0.23072    Maximum  0.304    Range  0.198    Interquartile range (IQR)  0.0338       Descriptive statistics     Standard deviation  0.02741428134    Coefficient of variation (CV)  0.1513247926    Kurtosis  1.287932992    Mean  0.1811618629    Median Absolute Deviation (MAD)  0.0171    Skewness  0.7256089734    Sum  103.0811    Variance  0.0007515428212           Histogram             2020-08-25T01:10:48.144387  image/svg+xml    Matplotlib v3.3.1, https://matplotlib.org/                                                                                                                                                                                                                                                                                                                                                                                                                    Histogram with fixed size bins  (bins=10)           Value  Count  Frequency (%)         0.1717  4  0.7%         0.1714  4  0.7%         0.1601  4  0.7%         0.1769  4  0.7%         0.1893  4  0.7%         0.1516  3  0.5%         0.193  3  0.5%         0.1779  3  0.5%         0.1925  3  0.5%         0.1943  3  0.5%         0.172  3  0.5%         0.1487  3  0.5%         0.1861  3  0.5%         0.1953  3  0.5%         0.1619  3  0.5%         0.1669  3  0.5%         0.159  3  0.5%         0.1506  3  0.5%         0.1667  3  0.5%         0.1809  3  0.5%         0.1885  3  0.5%         0.1739  3  0.5%         0.1467  3  0.5%         0.1966  3  0.5%         0.1735  3  0.5%         Other values (407)  489  85.9%               Minimum 5 values    Maximum 5 values         Value  Count  Frequency (%)         0.106  1  0.2%         0.1167  1  0.2%         0.1203  1  0.2%         0.1215  1  0.2%         0.122  1  0.2%         0.1274  1  0.2%         0.1305  1  0.2%         0.1308  1  0.2%         0.1337  1  0.2%         0.1339  1  0.2%               Value  Count  Frequency (%)         0.304  1  0.2%         0.2906  1  0.2%         0.2743  1  0.2%         0.2678  1  0.2%         0.2655  1  0.2%         0.2597  1  0.2%         0.2595  1  0.2%         0.2569  1  0.2%         0.2556  1  0.2%         0.2548  1  0.2%                         11   Real number (ℝ ≥0 )          Distinct count  499    Unique (%)  87.7%    Missing  0    Missing (%)  0.0%    Infinite  0    Infinite (%)  0.0%          Mean  0.06279760984182776    Minimum  0.04996    Maximum  0.09744    Zeros  0    Zeros (%)  0.0%    Memory size  4.6 KiB              2020-08-25T01:10:48.271605  image/svg+xml    Matplotlib v3.3.1, https://matplotlib.org/                                                                                                                                 Toggle details       Statistics    Histogram(s)    Common values    Extreme values       Quantile statistics     Minimum  0.04996    5-th percentile  0.053926    Q1  0.0577    median  0.06154    Q3  0.06612    95-th percentile  0.07609    Maximum  0.09744    Range  0.04748    Interquartile range (IQR)  0.00842       Descriptive statistics     Standard deviation  0.007060362795    Coefficient of variation (CV)  0.1124304382    Kurtosis  3.00589212    Mean  0.06279760984    Median Absolute Deviation (MAD)  0.00422    Skewness  1.304488813    Sum  35.73184    Variance  4.98487228e-05           Histogram             2020-08-25T01:10:48.373922  image/svg+xml    Matplotlib v3.3.1, https://matplotlib.org/                                                                                                                                                                                                                                                                                                                              Histogram with fixed size bins  (bins=10)           Value  Count  Frequency (%)         0.05667  3  0.5%         0.06113  3  0.5%         0.05907  3  0.5%         0.05913  3  0.5%         0.06782  3  0.5%         0.0614  2  0.4%         0.05883  2  0.4%         0.05708  2  0.4%         0.06697  2  0.4%         0.0613  2  0.4%         0.06412  2  0.4%         0.05859  2  0.4%         0.05976  2  0.4%         0.05916  2  0.4%         0.0578  2  0.4%         0.05955  2  0.4%         0.06317  2  0.4%         0.07005  2  0.4%         0.06048  2  0.4%         0.06612  2  0.4%         0.05884  2  0.4%         0.05898  2  0.4%         0.05912  2  0.4%         0.06129  2  0.4%         0.05581  2  0.4%         Other values (474)  514  90.3%               Minimum 5 values    Maximum 5 values         Value  Count  Frequency (%)         0.04996  1  0.2%         0.05024  1  0.2%         0.05025  1  0.2%         0.05044  1  0.2%         0.05054  1  0.2%         0.05096  1  0.2%         0.05176  1  0.2%         0.05177  1  0.2%         0.05185  1  0.2%         0.05223  1  0.2%               Value  Count  Frequency (%)         0.09744  1  0.2%         0.09575  1  0.2%         0.09502  1  0.2%         0.09296  1  0.2%         0.0898  1  0.2%         0.08743  1  0.2%         0.0845  1  0.2%         0.08261  1  0.2%         0.08243  1  0.2%         0.08142  1  0.2%                         30   Real number (ℝ ≥0 )          Distinct count  500    Unique (%)  87.9%    Missing  0    Missing (%)  0.0%    Infinite  0    Infinite (%)  0.0%          Mean  0.2900755711775044    Minimum  0.1565    Maximum  0.6638    Zeros  0    Zeros (%)  0.0%    Memory size  4.6 KiB              2020-08-25T01:10:48.489109  image/svg+xml    Matplotlib v3.3.1, https://matplotlib.org/                                                                                                                                             Toggle details       Statistics    Histogram(s)    Common values    Extreme values       Quantile statistics     Minimum  0.1565    5-th percentile  0.2127    Q1  0.2504    median  0.2822    Q3  0.3179    95-th percentile  0.40616    Maximum  0.6638    Range  0.5073    Interquartile range (IQR)  0.0675       Descriptive statistics     Standard deviation  0.06186746754    Coefficient of variation (CV)  0.2132805161    Kurtosis  4.444559518    Mean  0.2900755712    Median Absolute Deviation (MAD)  0.0342    Skewness  1.433927765    Sum  165.053    Variance  0.00382758354           Histogram             2020-08-25T01:10:48.588700  image/svg+xml    Matplotlib v3.3.1, https://matplotlib.org/                                                                                                                                                                                                                                                                                Histogram with fixed size bins  (bins=10)           Value  Count  Frequency (%)         0.2972  3  0.5%         0.2369  3  0.5%         0.2383  3  0.5%         0.2226  3  0.5%         0.3196  3  0.5%         0.3109  3  0.5%         0.3113  2  0.4%         0.2557  2  0.4%         0.2884  2  0.4%         0.2744  2  0.4%         0.271  2  0.4%         0.3055  2  0.4%         0.2826  2  0.4%         0.2955  2  0.4%         0.2556  2  0.4%         0.3379  2  0.4%         0.2651  2  0.4%         0.3105  2  0.4%         0.2227  2  0.4%         0.2458  2  0.4%         0.259  2  0.4%         0.3187  2  0.4%         0.3151  2  0.4%         0.251  2  0.4%         0.2576  2  0.4%         Other values (475)  513  90.2%               Minimum 5 values    Maximum 5 values         Value  Count  Frequency (%)         0.1565  1  0.2%         0.1566  1  0.2%         0.1603  1  0.2%         0.1648  1  0.2%         0.1652  1  0.2%         0.1712  1  0.2%         0.1783  2  0.4%         0.1811  1  0.2%         0.1859  1  0.2%         0.189  1  0.2%               Value  Count  Frequency (%)         0.6638  1  0.2%         0.5774  1  0.2%         0.5558  1  0.2%         0.544  1  0.2%         0.5166  1  0.2%         0.4882  1  0.2%         0.4863  1  0.2%         0.4824  1  0.2%         0.4761  1  0.2%         0.4753  1  0.2%                         26   Real number (ℝ ≥0 )          Distinct count  411    Unique (%)  72.2%    Missing  0    Missing (%)  0.0%    Infinite  0    Infinite (%)  0.0%          Mean  0.13236859402460457    Minimum  0.07117000000000001    Maximum  0.2226    Zeros  0    Zeros (%)  0.0%    Memory size  4.6 KiB              2020-08-25T01:10:48.693682  image/svg+xml    Matplotlib v3.3.1, https://matplotlib.org/                                                                                                                                                   Toggle details       Statistics    Histogram(s)    Common values    Extreme values       Quantile statistics     Minimum  0.07117    5-th percentile  0.095734    Q1  0.1166    median  0.1313    Q3  0.146    95-th percentile  0.17184    Maximum  0.2226    Range  0.15143    Interquartile range (IQR)  0.0294       Descriptive statistics     Standard deviation  0.0228324294    Coefficient of variation (CV)  0.172491289    Kurtosis  0.5178251903    Mean  0.132368594    Median Absolute Deviation (MAD)  0.0147    Skewness  0.4154259963    Sum  75.31773    Variance  0.0005213198325           Histogram             2020-08-25T01:10:48.810512  image/svg+xml    Matplotlib v3.3.1, https://matplotlib.org/                                                                                                                                                                                                                                                                                                                                                              Histogram with fixed size bins  (bins=10)           Value  Count  Frequency (%)         0.1223  4  0.7%         0.1256  4  0.7%         0.1216  4  0.7%         0.1275  4  0.7%         0.1312  4  0.7%         0.1347  4  0.7%         0.1401  4  0.7%         0.1415  4  0.7%         0.1234  4  0.7%         0.1218  3  0.5%         0.1289  3  0.5%         0.1249  3  0.5%         0.1072  3  0.5%         0.1142  3  0.5%         0.1368  3  0.5%         0.1491  3  0.5%         0.1166  3  0.5%         0.1118  3  0.5%         0.1402  3  0.5%         0.1298  3  0.5%         0.146  3  0.5%         0.1419  3  0.5%         0.1297  3  0.5%         0.1199  3  0.5%         0.1162  3  0.5%         Other values (386)  485  85.2%               Minimum 5 values    Maximum 5 values         Value  Count  Frequency (%)         0.07117  1  0.2%         0.08125  1  0.2%         0.08409  1  0.2%         0.08484  1  0.2%         0.08567  1  0.2%         0.08774  1  0.2%         0.08799  1  0.2%         0.08822  1  0.2%         0.08864  1  0.2%         0.08949  1  0.2%               Value  Count  Frequency (%)         0.2226  1  0.2%         0.2184  1  0.2%         0.2098  1  0.2%         0.2006  1  0.2%         0.1909  1  0.2%         0.1902  1  0.2%         0.1883  1  0.2%         0.1878  1  0.2%         0.1873  1  0.2%         0.1862  1  0.2%                         14   Real number (ℝ ≥0 )    HIGH CORRELATION           Distinct count  533    Unique (%)  93.7%    Missing  0    Missing (%)  0.0%    Infinite  0    Infinite (%)  0.0%          Mean  2.866059226713533    Minimum  0.757    Maximum  21.98    Zeros  0    Zeros (%)  0.0%    Memory size  4.6 KiB              2020-08-25T01:10:48.934659  image/svg+xml    Matplotlib v3.3.1, https://matplotlib.org/                                                                                                                                 Toggle details       Statistics    Histogram(s)    Common values    Extreme values       Quantile statistics     Minimum  0.757    5-th percentile  1.1328    Q1  1.606    median  2.287    Q3  3.357    95-th percentile  7.0416    Maximum  21.98    Range  21.223    Interquartile range (IQR)  1.751       Descriptive statistics     Standard deviation  2.021854554    Coefficient of variation (CV)  0.7054475829    Kurtosis  21.40190493    Mean  2.866059227    Median Absolute Deviation (MAD)  0.77    Skewness  3.443615202    Sum  1630.7877    Variance  4.087895838           Histogram             2020-08-25T01:10:49.039876  image/svg+xml    Matplotlib v3.3.1, https://matplotlib.org/                                                                                                                                                                                                                                                                                              Histogram with fixed size bins  (bins=10)           Value  Count  Frequency (%)         1.778  4  0.7%         2.155  2  0.4%         1.429  2  0.4%         1.535  2  0.4%         3.564  2  0.4%         2.406  2  0.4%         1.143  2  0.4%         1.101  2  0.4%         1.959  2  0.4%         1.566  2  0.4%         1.667  2  0.4%         2.363  2  0.4%         3.767  2  0.4%         1.994  2  0.4%         2.569  2  0.4%         2.765  2  0.4%         2.225  2  0.4%         2.873  2  0.4%         2.41  2  0.4%         2.183  2  0.4%         2.747  2  0.4%         1.955  2  0.4%         2.097  2  0.4%         1.597  2  0.4%         1.445  2  0.4%         Other values (508)  517  90.9%               Minimum 5 values    Maximum 5 values         Value  Count  Frequency (%)         0.757  1  0.2%         0.7714  1  0.2%         0.8439  1  0.2%         0.8484  1  0.2%         0.873  1  0.2%         0.9219  1  0.2%         0.968  1  0.2%         0.9812  1  0.2%         0.9857  1  0.2%         0.9887  1  0.2%               Value  Count  Frequency (%)         21.98  1  0.2%         18.65  1  0.2%         11.07  1  0.2%         10.12  1  0.2%         10.05  1  0.2%         9.807  1  0.2%         9.635  1  0.2%         9.424  1  0.2%         8.867  1  0.2%         8.83  1  0.2%                         2   Real number (ℝ ≥0 )    HIGH CORRELATION           Distinct count  456    Unique (%)  80.1%    Missing  0    Missing (%)  0.0%    Infinite  0    Infinite (%)  0.0%          Mean  14.127291739894552    Minimum  6.981    Maximum  28.11    Zeros  0    Zeros (%)  0.0%    Memory size  4.6 KiB              2020-08-25T01:10:49.147636  image/svg+xml    Matplotlib v3.3.1, https://matplotlib.org/                                                                                                                       Toggle details       Statistics    Histogram(s)    Common values    Extreme values       Quantile statistics     Minimum  6.981    5-th percentile  9.5292    Q1  11.7    median  13.37    Q3  15.78    95-th percentile  20.576    Maximum  28.11    Range  21.129    Interquartile range (IQR)  4.08       Descriptive statistics     Standard deviation  3.524048826    Coefficient of variation (CV)  0.2494497099    Kurtosis  0.8455216229    Mean  14.12729174    Median Absolute Deviation (MAD)  1.9    Skewness  0.9423795717    Sum  8038.429    Variance  12.41892013           Histogram             2020-08-25T01:10:49.245512  image/svg+xml    Matplotlib v3.3.1, https://matplotlib.org/                                                                                                                                                                                                                                                                                  Histogram with fixed size bins  (bins=10)           Value  Count  Frequency (%)         12.34  4  0.7%         12.77  3  0.5%         11.71  3  0.5%         13  3  0.5%         12.46  3  0.5%         13.17  3  0.5%         11.06  3  0.5%         12.18  3  0.5%         11.89  3  0.5%         11.6  3  0.5%         10.26  3  0.5%         13.85  3  0.5%         13.05  3  0.5%         15.46  3  0.5%         12.89  3  0.5%         14.99  2  0.4%         19.4  2  0.4%         12.05  2  0.4%         13.77  2  0.4%         13.2  2  0.4%         12.54  2  0.4%         14.87  2  0.4%         13.87  2  0.4%         13.28  2  0.4%         11.93  2  0.4%         Other values (431)  503  88.4%               Minimum 5 values    Maximum 5 values         Value  Count  Frequency (%)         6.981  1  0.2%         7.691  1  0.2%         7.729  1  0.2%         7.76  1  0.2%         8.196  1  0.2%         8.219  1  0.2%         8.571  1  0.2%         8.597  1  0.2%         8.598  1  0.2%         8.618  1  0.2%               Value  Count  Frequency (%)         28.11  1  0.2%         27.42  1  0.2%         27.22  1  0.2%         25.73  1  0.2%         25.22  1  0.2%         24.63  1  0.2%         24.25  1  0.2%         23.51  1  0.2%         23.29  1  0.2%         23.27  1  0.2%                         6   Real number (ℝ ≥0 )          Distinct count  474    Unique (%)  83.3%    Missing  0    Missing (%)  0.0%    Infinite  0    Infinite (%)  0.0%          Mean  0.09636028119507908    Minimum  0.05263    Maximum  0.1634    Zeros  0    Zeros (%)  0.0%    Memory size  4.6 KiB              2020-08-25T01:10:49.353428  image/svg+xml    Matplotlib v3.3.1, https://matplotlib.org/                                                                                                                                               Toggle details       Statistics    Histogram(s)    Common values    Extreme values       Quantile statistics     Minimum  0.05263    5-th percentile  0.075042    Q1  0.08637    median  0.09587    Q3  0.1053    95-th percentile  0.11878    Maximum  0.1634    Range  0.11077    Interquartile range (IQR)  0.01893       Descriptive statistics     Standard deviation  0.01406412814    Coefficient of variation (CV)  0.1459535813    Kurtosis  0.8559749304    Mean  0.0963602812    Median Absolute Deviation (MAD)  0.0095    Skewness  0.4563237648    Sum  54.829    Variance  0.0001977997003           Histogram             2020-08-25T01:10:49.461315  image/svg+xml    Matplotlib v3.3.1, https://matplotlib.org/                                                                                                                                                                                                                                                                                                                                              Histogram with fixed size bins  (bins=10)           Value  Count  Frequency (%)         0.1007  5  0.9%         0.115  4  0.7%         0.1054  4  0.7%         0.1075  4  0.7%         0.1024  3  0.5%         0.09462  3  0.5%         0.1089  3  0.5%         0.1158  3  0.5%         0.1063  3  0.5%         0.09831  3  0.5%         0.1082  3  0.5%         0.1066  3  0.5%         0.1044  3  0.5%         0.1096  3  0.5%         0.1099  3  0.5%         0.1141  3  0.5%         0.1049  3  0.5%         0.1037  3  0.5%         0.08511  3  0.5%         0.117  3  0.5%         0.103  2  0.4%         0.09968  2  0.4%         0.1036  2  0.4%         0.1006  2  0.4%         0.08192  2  0.4%         Other values (449)  494  86.8%               Minimum 5 values    Maximum 5 values         Value  Count  Frequency (%)         0.05263  1  0.2%         0.06251  1  0.2%         0.06429  1  0.2%         0.06576  1  0.2%         0.06613  1  0.2%         0.06828  1  0.2%         0.06883  1  0.2%         0.06935  1  0.2%         0.0695  1  0.2%         0.06955  1  0.2%               Value  Count  Frequency (%)         0.1634  1  0.2%         0.1447  1  0.2%         0.1425  1  0.2%         0.1398  1  0.2%         0.1371  1  0.2%         0.1335  1  0.2%         0.1326  1  0.2%         0.1323  1  0.2%         0.1291  1  0.2%         0.1286  1  0.2%                         18   Real number (ℝ ≥0 )   ZEROS          Distinct count  533    Unique (%)  93.7%    Missing  0    Missing (%)  0.0%    Infinite  0    Infinite (%)  0.0%          Mean  0.03189371634446397    Minimum  0.0    Maximum  0.396    Zeros  13    Zeros (%)  2.3%    Memory size  4.6 KiB              2020-08-25T01:10:49.580550  image/svg+xml    Matplotlib v3.3.1, https://matplotlib.org/                                                                                                                                           Toggle details       Statistics    Histogram(s)    Common values    Extreme values       Quantile statistics     Minimum  0    5-th percentile  0.0032526    Q1  0.01509    median  0.02589    Q3  0.04205    95-th percentile  0.078936    Maximum  0.396    Range  0.396    Interquartile range (IQR)  0.02696       Descriptive statistics     Standard deviation  0.03018606032    Coefficient of variation (CV)  0.9464579166    Kurtosis  48.8613953    Mean  0.03189371634    Median Absolute Deviation (MAD)  0.01248    Skewness  5.110463049    Sum  18.1475246    Variance  0.0009111982378           Histogram             2020-08-25T01:10:49.697916  image/svg+xml    Matplotlib v3.3.1, https://matplotlib.org/                                                                                                                                                                                                                                                                                                                                                                                                Histogram with fixed size bins  (bins=10)           Value  Count  Frequency (%)         0  13  2.3%         0.03576  2  0.4%         0.04344  2  0.4%         0.018  2  0.4%         0.02071  2  0.4%         0.02664  2  0.4%         0.03872  2  0.4%         0.02185  2  0.4%         0.01698  2  0.4%         0.05371  2  0.4%         0.02945  2  0.4%         0.01311  2  0.4%         0.0151  2  0.4%         0.01514  2  0.4%         0.02443  2  0.4%         0.01079  2  0.4%         0.02332  2  0.4%         0.01652  2  0.4%         0.02117  2  0.4%         0.01865  2  0.4%         0.01412  2  0.4%         0.02681  2  0.4%         0.01376  2  0.4%         0.02  2  0.4%         0.03452  2  0.4%         Other values (508)  508  89.3%               Minimum 5 values    Maximum 5 values         Value  Count  Frequency (%)         0  13  2.3%         0.000692  1  0.2%         0.0007929  1  0.2%         0.0009737  1  0.2%         0.001128  1  0.2%         0.001184  1  0.2%         0.001487  1  0.2%         0.001595  1  0.2%         0.001597  1  0.2%         0.001835  1  0.2%               Value  Count  Frequency (%)         0.396  1  0.2%         0.3038  1  0.2%         0.1535  1  0.2%         0.1438  1  0.2%         0.1435  1  0.2%         0.1278  1  0.2%         0.1197  1  0.2%         0.1166  1  0.2%         0.1114  1  0.2%         0.1091  1  0.2%                         7   Real number (ℝ ≥0 )          Distinct count  537    Unique (%)  94.4%    Missing  0    Missing (%)  0.0%    Infinite  0    Infinite (%)  0.0%          Mean  0.10434098418277679    Minimum  0.01938    Maximum  0.3454    Zeros  0    Zeros (%)  0.0%    Memory size  4.6 KiB              2020-08-25T01:10:49.827523  image/svg+xml    Matplotlib v3.3.1, https://matplotlib.org/                                                                                                                                             Toggle details       Statistics    Histogram(s)    Common values    Extreme values       Quantile statistics     Minimum  0.01938    5-th percentile  0.04066    Q1  0.06492    median  0.09263    Q3  0.1304    95-th percentile  0.2087    Maximum  0.3454    Range  0.32602    Interquartile range (IQR)  0.06548       Descriptive statistics     Standard deviation  0.05281275793    Coefficient of variation (CV)  0.5061554512    Kurtosis  1.650130467    Mean  0.1043409842    Median Absolute Deviation (MAD)  0.03263    Skewness  1.190123031    Sum  59.37002    Variance  0.0027891874           Histogram             2020-08-25T01:10:50.135606  image/svg+xml    Matplotlib v3.3.1, https://matplotlib.org/                                                                                                                                                                                                                                                                                                                                                  Histogram with fixed size bins  (bins=10)           Value  Count  Frequency (%)         0.1206  3  0.5%         0.1147  3  0.5%         0.1117  2  0.4%         0.1223  2  0.4%         0.1267  2  0.4%         0.07698  2  0.4%         0.05743  2  0.4%         0.03834  2  0.4%         0.1154  2  0.4%         0.1313  2  0.4%         0.17  2  0.4%         0.1305  2  0.4%         0.1339  2  0.4%         0.1141  2  0.4%         0.1599  2  0.4%         0.1516  2  0.4%         0.1483  2  0.4%         0.1021  2  0.4%         0.2087  2  0.4%         0.09509  2  0.4%         0.07722  2  0.4%         0.1289  2  0.4%         0.05794  2  0.4%         0.1283  2  0.4%         0.1306  2  0.4%         Other values (512)  517  90.9%               Minimum 5 values    Maximum 5 values         Value  Count  Frequency (%)         0.01938  1  0.2%         0.02344  1  0.2%         0.0265  1  0.2%         0.02675  1  0.2%         0.03116  1  0.2%         0.03212  1  0.2%         0.03393  1  0.2%         0.03398  1  0.2%         0.03454  1  0.2%         0.03515  1  0.2%               Value  Count  Frequency (%)         0.3454  1  0.2%         0.3114  1  0.2%         0.2867  1  0.2%         0.2839  1  0.2%         0.2832  1  0.2%         0.2776  1  0.2%         0.277  1  0.2%         0.2768  1  0.2%         0.2665  1  0.2%         0.2576  1  0.2%                         15   Real number (ℝ ≥0 )    HIGH CORRELATION           Distinct count  528    Unique (%)  92.8%    Missing  0    Missing (%)  0.0%    Infinite  0    Infinite (%)  0.0%          Mean  40.337079086116    Minimum  6.8020000000000005    Maximum  542.2    Zeros  0    Zeros (%)  0.0%    Memory size  4.6 KiB              2020-08-25T01:10:50.254627  image/svg+xml    Matplotlib v3.3.1, https://matplotlib.org/                                                                                                                                     Toggle details       Statistics    Histogram(s)    Common values    Extreme values       Quantile statistics     Minimum  6.802    5-th percentile  11.36    Q1  17.85    median  24.53    Q3  45.19    95-th percentile  115.8    Maximum  542.2    Range  535.398    Interquartile range (IQR)  27.34       Descriptive statistics     Standard deviation  45.49100552    Coefficient of variation (CV)  1.127771434    Kurtosis  49.20907651    Mean  40.33707909    Median Absolute Deviation (MAD)  9.19    Skewness  5.447186285    Sum  22951.798    Variance  2069.431583           Histogram             2020-08-25T01:10:50.352205  image/svg+xml    Matplotlib v3.3.1, https://matplotlib.org/                                                                                                                                                                                                                                                                                      Histogram with fixed size bins  (bins=10)           Value  Count  Frequency (%)         17.67  3  0.5%         16.64  3  0.5%         16.97  3  0.5%         18.54  3  0.5%         74.08  2  0.4%         19.53  2  0.4%         20.56  2  0.4%         16.39  2  0.4%         20.2  2  0.4%         17.74  2  0.4%         22.79  2  0.4%         23.92  2  0.4%         14.34  2  0.4%         20.95  2  0.4%         19.87  2  0.4%         23.13  2  0.4%         17.86  2  0.4%         20.74  2  0.4%         23.12  2  0.4%         12.67  2  0.4%         33.01  2  0.4%         20.67  2  0.4%         18.15  2  0.4%         104.9  2  0.4%         34.37  2  0.4%         Other values (503)  515  90.5%               Minimum 5 values    Maximum 5 values         Value  Count  Frequency (%)         6.802  1  0.2%         7.228  1  0.2%         7.254  1  0.2%         7.326  1  0.2%         8.205  1  0.2%         8.322  1  0.2%         8.605  1  0.2%         8.955  1  0.2%         8.966  1  0.2%         9.006  1  0.2%               Value  Count  Frequency (%)         542.2  1  0.2%         525.6  1  0.2%         233  1  0.2%         224.1  1  0.2%         199.7  1  0.2%         180.2  1  0.2%         176.5  1  0.2%         170  1  0.2%         164.1  1  0.2%         158.7  1  0.2%                         13   Real number (ℝ ≥0 )          Distinct count  519    Unique (%)  91.2%    Missing  0    Missing (%)  0.0%    Infinite  0    Infinite (%)  0.0%          Mean  1.2168534270650264    Minimum  0.3602    Maximum  4.885    Zeros  0    Zeros (%)  0.0%    Memory size  4.6 KiB              2020-08-25T01:10:50.459054  image/svg+xml    Matplotlib v3.3.1, https://matplotlib.org/                                                                                                                 Toggle details       Statistics    Histogram(s)    Common values    Extreme values       Quantile statistics     Minimum  0.3602    5-th percentile  0.54014    Q1  0.8339    median  1.108    Q3  1.474    95-th percentile  2.212    Maximum  4.885    Range  4.5248    Interquartile range (IQR)  0.6401       Descriptive statistics     Standard deviation  0.5516483926    Coefficient of variation (CV)  0.4533400493    Kurtosis  5.349168692    Mean  1.216853427    Median Absolute Deviation (MAD)  0.3153    Skewness  1.646443809    Sum  692.3896    Variance  0.3043159491           Histogram             2020-08-25T01:10:50.555565  image/svg+xml    Matplotlib v3.3.1, https://matplotlib.org/                                                                                                                                                                                                                                                        Histogram with fixed size bins  (bins=10)           Value  Count  Frequency (%)         0.8561  3  0.5%         1.268  3  0.5%         1.35  3  0.5%         1.15  3  0.5%         1.563  2  0.4%         1.627  2  0.4%         0.9429  2  0.4%         1.39  2  0.4%         1.169  2  0.4%         1.059  2  0.4%         1.216  2  0.4%         1.033  2  0.4%         1.166  2  0.4%         1.023  2  0.4%         1.027  2  0.4%         1.199  2  0.4%         1.095  2  0.4%         0.7339  2  0.4%         1.363  2  0.4%         1.046  2  0.4%         0.8225  2  0.4%         1.001  2  0.4%         1.045  2  0.4%         1.016  2  0.4%         1.214  2  0.4%         Other values (494)  515  90.5%               Minimum 5 values    Maximum 5 values         Value  Count  Frequency (%)         0.3602  1  0.2%         0.3621  1  0.2%         0.3628  1  0.2%         0.3871  1  0.2%         0.3981  1  0.2%         0.4064  1  0.2%         0.4125  1  0.2%         0.4334  1  0.2%         0.4336  1  0.2%         0.4402  1  0.2%               Value  Count  Frequency (%)         4.885  1  0.2%         3.896  1  0.2%         3.647  1  0.2%         3.568  1  0.2%         3.12  1  0.2%         2.927  1  0.2%         2.91  1  0.2%         2.904  1  0.2%         2.878  1  0.2%         2.836  1  0.2%                         24   Real number (ℝ ≥0 )    HIGH CORRELATION           Distinct count  514    Unique (%)  90.3%    Missing  0    Missing (%)  0.0%    Infinite  0    Infinite (%)  0.0%          Mean  107.26121265377857    Minimum  50.41    Maximum  251.2    Zeros  0    Zeros (%)  0.0%    Memory size  4.6 KiB              2020-08-25T01:10:50.659803  image/svg+xml    Matplotlib v3.3.1, https://matplotlib.org/                                                                                                                           Toggle details       Statistics    Histogram(s)    Common values    Extreme values       Quantile statistics     Minimum  50.41    5-th percentile  67.856    Q1  84.11    median  97.66    Q3  125.4    95-th percentile  171.64    Maximum  251.2    Range  200.79    Interquartile range (IQR)  41.29       Descriptive statistics     Standard deviation  33.60254227    Coefficient of variation (CV)  0.3132776652    Kurtosis  1.070149667    Mean  107.2612127    Median Absolute Deviation (MAD)  16.87    Skewness  1.128163871    Sum  61031.63    Variance  1129.130847           Histogram             2020-08-25T01:10:50.769410  image/svg+xml    Matplotlib v3.3.1, https://matplotlib.org/                                                                                                                                                                                                                                                                                                                                                  Histogram with fixed size bins  (bins=10)           Value  Count  Frequency (%)         101.7  3  0.5%         105.9  3  0.5%         117.7  3  0.5%         104.5  2  0.4%         152.4  2  0.4%         79.93  2  0.4%         79.73  2  0.4%         184.6  2  0.4%         85.07  2  0.4%         113.7  2  0.4%         76.51  2  0.4%         127.1  2  0.4%         85.56  2  0.4%         100.9  2  0.4%         171.1  2  0.4%         84.48  2  0.4%         170.3  2  0.4%         87.36  2  0.4%         152.2  2  0.4%         92.04  2  0.4%         158.8  2  0.4%         108.1  2  0.4%         95.29  2  0.4%         159.8  2  0.4%         135.1  2  0.4%         Other values (489)  516  90.7%               Minimum 5 values    Maximum 5 values         Value  Count  Frequency (%)         50.41  1  0.2%         54.49  1  0.2%         56.65  1  0.2%         57.17  1  0.2%         57.26  1  0.2%         58.08  1  0.2%         58.36  1  0.2%         59.16  1  0.2%         59.9  1  0.2%         60.9  1  0.2%               Value  Count  Frequency (%)         251.2  1  0.2%         229.3  1  0.2%         220.8  1  0.2%         214  1  0.2%         211.7  1  0.2%         211.5  1  0.2%         206.8  1  0.2%         206  1  0.2%         205.7  1  0.2%         202.4  1  0.2%                         3   Real number (ℝ ≥0 )          Distinct count  479    Unique (%)  84.2%    Missing  0    Missing (%)  0.0%    Infinite  0    Infinite (%)  0.0%          Mean  19.289648506151142    Minimum  9.71    Maximum  39.28    Zeros  0    Zeros (%)  0.0%    Memory size  4.6 KiB              2020-08-25T01:10:50.892652  image/svg+xml    Matplotlib v3.3.1, https://matplotlib.org/                                                                                                                       Toggle details       Statistics    Histogram(s)    Common values    Extreme values       Quantile statistics     Minimum  9.71    5-th percentile  13.088    Q1  16.17    median  18.84    Q3  21.8    95-th percentile  27.15    Maximum  39.28    Range  29.57    Interquartile range (IQR)  5.63       Descriptive statistics     Standard deviation  4.301035768    Coefficient of variation (CV)  0.2229711841    Kurtosis  0.7583189724    Mean  19.28964851    Median Absolute Deviation (MAD)  2.81    Skewness  0.6504495421    Sum  10975.81    Variance  18.49890868           Histogram             2020-08-25T01:10:51.000901  image/svg+xml    Matplotlib v3.3.1, https://matplotlib.org/                                                                                                                                                                                                                                                                                                                                      Histogram with fixed size bins  (bins=10)           Value  Count  Frequency (%)         14.93  3  0.5%         15.7  3  0.5%         18.9  3  0.5%         16.84  3  0.5%         17.46  3  0.5%         18.22  3  0.5%         20.52  3  0.5%         16.85  3  0.5%         19.83  3  0.5%         18.89  2  0.4%         13.98  2  0.4%         20.22  2  0.4%         18.18  2  0.4%         18.61  2  0.4%         21.25  2  0.4%         27.15  2  0.4%         21.84  2  0.4%         14.96  2  0.4%         16.58  2  0.4%         20.76  2  0.4%         21.53  2  0.4%         15.51  2  0.4%         21.46  2  0.4%         21.59  2  0.4%         13.9  2  0.4%         Other values (454)  510  89.6%               Minimum 5 values    Maximum 5 values         Value  Count  Frequency (%)         9.71  1  0.2%         10.38  1  0.2%         10.72  1  0.2%         10.82  1  0.2%         10.89  1  0.2%         10.91  1  0.2%         10.94  1  0.2%         11.28  1  0.2%         11.79  1  0.2%         11.89  1  0.2%               Value  Count  Frequency (%)         39.28  1  0.2%         33.81  1  0.2%         33.56  1  0.2%         32.47  1  0.2%         31.12  1  0.2%         30.72  1  0.2%         30.62  1  0.2%         29.97  1  0.2%         29.81  1  0.2%         29.43  1  0.2%                         4   Real number (ℝ ≥0 )    HIGH CORRELATION           Distinct count  522    Unique (%)  91.7%    Missing  0    Missing (%)  0.0%    Infinite  0    Infinite (%)  0.0%          Mean  91.96903339191564    Minimum  43.79    Maximum  188.5    Zeros  0    Zeros (%)  0.0%    Memory size  4.6 KiB              2020-08-25T01:10:51.123509  image/svg+xml    Matplotlib v3.3.1, https://matplotlib.org/                                                                                                                                     Toggle details       Statistics    Histogram(s)    Common values    Extreme values       Quantile statistics     Minimum  43.79    5-th percentile  60.496    Q1  75.17    median  86.24    Q3  104.1    95-th percentile  135.82    Maximum  188.5    Range  144.71    Interquartile range (IQR)  28.93       Descriptive statistics     Standard deviation  24.29898104    Coefficient of variation (CV)  0.2642082899    Kurtosis  0.9722135477    Mean  91.96903339    Median Absolute Deviation (MAD)  12.71    Skewness  0.9906504254    Sum  52330.38    Variance  590.4404795           Histogram             2020-08-25T01:10:51.235777  image/svg+xml    Matplotlib v3.3.1, https://matplotlib.org/                                                                                                                                                                                                                                                                                                                                                Histogram with fixed size bins  (bins=10)           Value  Count  Frequency (%)         82.61  3  0.5%         134.7  3  0.5%         87.76  3  0.5%         130  2  0.4%         58.79  2  0.4%         133.8  2  0.4%         85.98  2  0.4%         113.4  2  0.4%         81.35  2  0.4%         84.08  2  0.4%         73.34  2  0.4%         107.1  2  0.4%         130.7  2  0.4%         88.37  2  0.4%         117.4  2  0.4%         88.73  2  0.4%         78.83  2  0.4%         78.29  2  0.4%         79.19  2  0.4%         102.4  2  0.4%         132.4  2  0.4%         71.49  2  0.4%         82.69  2  0.4%         132.9  2  0.4%         87.21  2  0.4%         Other values (497)  516  90.7%               Minimum 5 values    Maximum 5 values         Value  Count  Frequency (%)         43.79  1  0.2%         47.92  1  0.2%         47.98  1  0.2%         48.34  1  0.2%         51.71  1  0.2%         53.27  1  0.2%         54.09  1  0.2%         54.34  1  0.2%         54.42  1  0.2%         54.53  1  0.2%               Value  Count  Frequency (%)         188.5  1  0.2%         186.9  1  0.2%         182.1  1  0.2%         174.2  1  0.2%         171.5  1  0.2%         166.2  1  0.2%         165.5  1  0.2%         158.9  1  0.2%         155.1  1  0.2%         153.5  1  0.2%                         27   Real number (ℝ ≥0 )          Distinct count  529    Unique (%)  93.0%    Missing  0    Missing (%)  0.0%    Infinite  0    Infinite (%)  0.0%          Mean  0.25426504393673116    Minimum  0.02729    Maximum  1.058    Zeros  0    Zeros (%)  0.0%    Memory size  4.6 KiB              2020-08-25T01:10:51.358377  image/svg+xml    Matplotlib v3.3.1, https://matplotlib.org/                                                                                                                                           Toggle details       Statistics    Histogram(s)    Common values    Extreme values       Quantile statistics     Minimum  0.02729    5-th percentile  0.071196    Q1  0.1472    median  0.2119    Q3  0.3391    95-th percentile  0.56412    Maximum  1.058    Range  1.03071    Interquartile range (IQR)  0.1919       Descriptive statistics     Standard deviation  0.1573364889    Coefficient of variation (CV)  0.6187893014    Kurtosis  3.039288172    Mean  0.2542650439    Median Absolute Deviation (MAD)  0.0871    Skewness  1.4735549    Sum  144.67681    Variance  0.02475477074           Histogram             2020-08-25T01:10:51.475788  image/svg+xml    Matplotlib v3.3.1, https://matplotlib.org/                                                                                                                                                                                                                                                                                                                                              Histogram with fixed size bins  (bins=10)           Value  Count  Frequency (%)         0.1486  3  0.5%         0.3416  3  0.5%         0.2264  2  0.4%         0.1517  2  0.4%         0.165  2  0.4%         0.07348  2  0.4%         0.3583  2  0.4%         0.1676  2  0.4%         0.3735  2  0.4%         0.4061  2  0.4%         0.2698  2  0.4%         0.171  2  0.4%         0.1049  2  0.4%         0.4706  2  0.4%         0.217  2  0.4%         0.3055  2  0.4%         0.448  2  0.4%         0.1808  2  0.4%         0.1352  2  0.4%         0.1879  2  0.4%         0.1202  2  0.4%         0.1773  2  0.4%         0.1346  2  0.4%         0.255  2  0.4%         0.1843  2  0.4%         Other values (504)  517  90.9%               Minimum 5 values    Maximum 5 values         Value  Count  Frequency (%)         0.02729  1  0.2%         0.03432  1  0.2%         0.04327  1  0.2%         0.04619  1  0.2%         0.04712  1  0.2%         0.04953  1  0.2%         0.05036  1  0.2%         0.05131  1  0.2%         0.05213  1  0.2%         0.05232  1  0.2%               Value  Count  Frequency (%)         1.058  1  0.2%         0.9379  1  0.2%         0.9327  1  0.2%         0.8681  1  0.2%         0.8663  1  0.2%         0.7917  1  0.2%         0.7725  1  0.2%         0.7584  1  0.2%         0.7444  1  0.2%         0.7394  1  0.2%                         target   Boolean          Distinct count  2    Unique (%)  0.4%    Missing  0    Missing (%)  0.0%    Memory size  4.6 KiB           0    357      1    212          Toggle details       Frequency Table         Value  Count  Frequency (%)         0  357  62.7%         1  212  37.3%                    Interactions       29   17   25   19   10   11   30   26   14   2   6   18   7   15   13   24   3   4   27          29   17   25   19   10   11   30   26   14   2   6   18   7   15   13   24   3   4   27              2020-08-25T01:09:52.948480  image/svg+xml    Matplotlib v3.3.1, https://matplotlib.org/                                                                                                                                                                                                                                                                                                                                                                                                                                                                                                                                                                                                                                                                                                                                                                                                                                                                                                                                                                                                                                                                                                                                                                                                                                                                                                                                                                                                                                                                                                  2020-08-25T01:09:53.089715  image/svg+xml    Matplotlib v3.3.1, https://matplotlib.org/                                                                                                                                                                                                                                                                                                                                                                                                                                                                                                                                                                                                                                                                                                                                                                                                                                                                                                                                                                                                                                                                                                                                                                                                                                                                                                                                                                                                                                                                                                                                2020-08-25T01:09:53.232380  image/svg+xml    Matplotlib v3.3.1, https://matplotlib.org/                                                                                                                                                                                                                                                                                                                                                                                                                                                                                                                                                                                                                                                                                                                                                                                                                                                                                                                                                                                                                                                                                                                                                                                                                                                                                                                                                                                                                                                                                                                          2020-08-25T01:09:53.376572  image/svg+xml    Matplotlib v3.3.1, https://matplotlib.org/                                                                                                                                                                                                                                                                                                                                                                                                                                                                                                                                                                                                                                                                                                                                                                                                                                                                                                                                                                                                                                                                                                                                                                                                                                                                                                                                                                                                                                                                                        2020-08-25T01:09:53.515208  image/svg+xml    Matplotlib v3.3.1, https://matplotlib.org/                                                                                                                                                                                                                                                                                                                                                                                                                                                                                                                                                                                                                                                                                                                                                                                                                                                                                                                                                                                                                                                                                                                                                                                                                                                                                                                                                                                                                                                                                                                                                    2020-08-25T01:09:53.663058  image/svg+xml    Matplotlib v3.3.1, https://matplotlib.org/                                                                                                                                                                                                                                                                                                                                                                                                                                                                                                                                                                                                                                                                                                                                                                                                                                                                                                                                                                                                                                                                                                                                                                                                                                                                                                                                                                                                                                                                                  2020-08-25T01:09:53.803077  image/svg+xml    Matplotlib v3.3.1, https://matplotlib.org/                                                                                                                                                                                                                                                                                                                                                                                                                                                                                                                                                                                                                                                                                                                                                                                                                                                                                                                                                                                                                                                                                                                                                                                                                                                                                                                                                                                                                                                                    2020-08-25T01:09:53.939672  image/svg+xml    Matplotlib v3.3.1, https://matplotlib.org/                                                                                                                                                                                                                                                                                                                                                                                                                                                                                                                                                                                                                                                                                                                                                                                                                                                                                                                                                                                                                                                                                                                                                                                                                                                                                                                                                                                                                                                                                                                            2020-08-25T01:09:54.090229  image/svg+xml    Matplotlib v3.3.1, https://matplotlib.org/                                                                                                                                                                                                                                                                                                                                                                                                                                                                                                                                                                                                                                                                                                                                                                                                                                                                                                                                                                                                                                                                                                                                                                                                                                                                                                                                                                                                                                                  2020-08-25T01:09:54.224219  image/svg+xml    Matplotlib v3.3.1, https://matplotlib.org/                                                                                                                                                                                                                                                                                                                                                                                                                                                                                                                                                                                                                                                                                                                                                                                                                                                                                                                                                                                                                                                                                                                                                                                                                                                                                                                                                                                                                                      2020-08-25T01:09:54.354799  image/svg+xml    Matplotlib v3.3.1, https://matplotlib.org/                                                                                                                                                                                                                                                                                                                                                                                                                                                                                                                                                                                                                                                                                                                                                                                                                                                                                                                                                                                                                                                                                                                                                                                                                                                                                                                                                                                                                                                                                              2020-08-25T01:09:54.495357  image/svg+xml    Matplotlib v3.3.1, https://matplotlib.org/                                                                                                                                                                                                                                                                                                                                                                                                                                                                                                                                                                                                                                                                                                                                                                                                                                                                                                                                                                                                                                                                                                                                                                                                                                                                                                                                                                                                                                                                                                                                      2020-08-25T01:09:54.647120  image/svg+xml    Matplotlib v3.3.1, https://matplotlib.org/                                                                                                                                                                                                                                                                                                                                                                                                                                                                                                                                                                                                                                                                                                                                                                                                                                                                                                                                                                                                                                                                                                                                                                                                                                                                                                                                                                                                                                                                                                    2020-08-25T01:09:54.788111  image/svg+xml    Matplotlib v3.3.1, https://matplotlib.org/                                                                                                                                                                                                                                                                                                                                                                                                                                                                                                                                                                                                                                                                                                                                                                                                                                                                                                                                                                                                                                                                                                                                                                                                                                                                                                                                                                                                                                                                        2020-08-25T01:09:54.923809  image/svg+xml    Matplotlib v3.3.1, https://matplotlib.org/                                                                                                                                                                                                                                                                                                                                                                                                                                                                                                                                                                                                                                                                                                                                                                                                                                                                                                                                                                                                                                                                                                                                                                                                                                                                                                                                                                                                                                            2020-08-25T01:09:55.056845  image/svg+xml    Matplotlib v3.3.1, https://matplotlib.org/                                                                                                                                                                                                                                                                                                                                                                                                                                                                                                                                                                                                                                                                                                                                                                                                                                                                                                                                                                                                                                                                                                                                                                                                                                                                                                                                                                                                                                                                                                                2020-08-25T01:09:55.205251  image/svg+xml    Matplotlib v3.3.1, https://matplotlib.org/                                                                                                                                                                                                                                                                                                                                                                                                                                                                                                                                                                                                                                                                                                                                                                                                                                                                                                                                                                                                                                                                                                                                                                                                                                                                                                                                                                                                                                                                        2020-08-25T01:09:55.345117  image/svg+xml    Matplotlib v3.3.1, https://matplotlib.org/                                                                                                                                                                                                                                                                                                                                                                                                                                                                                                                                                                                                                                                                                                                                                                                                                                                                                                                                                                                                                                                                                                                                                                                                                                                                                                                                                                                                                                                                                                    2020-08-25T01:09:55.509563  image/svg+xml    Matplotlib v3.3.1, https://matplotlib.org/                                                                                                                                                                                                                                                                                                                                                                                                                                                                                                                                                                                                                                                                                                                                                                                                                                                                                                                                                                                                                                                                                                                                                                                                                                                                                                                                                                                                                                                                                       29   17   25   19   10   11   30   26   14   2   6   18   7   15   13   24   3   4   27              2020-08-25T01:09:55.656679  image/svg+xml    Matplotlib v3.3.1, https://matplotlib.org/                                                                                                                                                                                                                                                                                                                                                                                                                                                                                                                                                                                                                                                                                                                                                                                                                                                                                                                                                                                                                                                                                                                                                                                                                                                                                                                                                                                                                                                                                                                                2020-08-25T01:09:55.808458  image/svg+xml    Matplotlib v3.3.1, https://matplotlib.org/                                                                                                                                                                                                                                                                                                                                                                                                                                                                                                                                                                                                                                                                                                                                                                                                                                                                                                                                                                                                                                                                                                                                                                                                                                                                                                                                                                                                                                                                                                                                  2020-08-25T01:09:55.963297  image/svg+xml    Matplotlib v3.3.1, https://matplotlib.org/                                                                                                                                                                                                                                                                                                                                                                                                                                                                                                                                                                                                                                                                                                                                                                                                                                                                                                                                                                                                                                                                                                                                                                                                                                                                                                                                                                                                                                                                                                                                                2020-08-25T01:09:56.174293  image/svg+xml    Matplotlib v3.3.1, https://matplotlib.org/                                                                                                                                                                                                                                                                                                                                                                                                                                                                                                                                                                                                                                                                                                                                                                                                                                                                                                                                                                                                                                                                                                                                                                                                                                                                                                                                                                                                                                                                                                                  2020-08-25T01:09:56.362855  image/svg+xml    Matplotlib v3.3.1, https://matplotlib.org/                                                                                                                                                                                                                                                                                                                                                                                                                                                                                                                                                                                                                                                                                                                                                                                                                                                                                                                                                                                                                                                                                                                                                                                                                                                                                                                                                                                                                                                                                                                                                                          2020-08-25T01:09:56.521731  image/svg+xml    Matplotlib v3.3.1, https://matplotlib.org/                                                                                                                                                                                                                                                                                                                                                                                                                                                                                                                                                                                                                                                                                                                                                                                                                                                                                                                                                                                                                                                                                                                                                                                                                                                                                                                                                                                                                                                                                                2020-08-25T01:09:56.664084  image/svg+xml    Matplotlib v3.3.1, https://matplotlib.org/                                                                                                                                                                                                                                                                                                                                                                                                                                                                                                                                                                                                                                                                                                                                                                                                                                                                                                                                                                                                                                                                                                                                                                                                                                                                                                                                                                                                                                                                                      2020-08-25T01:09:56.804826  image/svg+xml    Matplotlib v3.3.1, https://matplotlib.org/                                                                                                                                                                                                                                                                                                                                                                                                                                                                                                                                                                                                                                                                                                                                                                                                                                                                                                                                                                                                                                                                                                                                                                                                                                                                                                                                                                                                                                                                                                                                  2020-08-25T01:09:56.958280  image/svg+xml    Matplotlib v3.3.1, https://matplotlib.org/                                                                                                                                                                                                                                                                                                                                                                                                                                                                                                                                                                                                                                                                                                                                                                                                                                                                                                                                                                                                                                                                                                                                                                                                                                                                                                                                                                                                                                                                    2020-08-25T01:09:57.293919  image/svg+xml    Matplotlib v3.3.1, https://matplotlib.org/                                                                                                                                                                                                                                                                                                                                                                                                                                                                                                                                                                                                                                                                                                                                                                                                                                                                                                                                                                                                                                                                                                                                                                                                                                                                                                                                                                                                                                                            2020-08-25T01:09:57.435183  image/svg+xml    Matplotlib v3.3.1, https://matplotlib.org/                                                                                                                                                                                                                                                                                                                                                                                                                                                                                                                                                                                                                                                                                                                                                                                                                                                                                                                                                                                                                                                                                                                                                                                                                                                                                                                                                                                                                                                                                                    2020-08-25T01:09:57.581036  image/svg+xml    Matplotlib v3.3.1, https://matplotlib.org/                                                                                                                                                                                                                                                                                                                                                                                                                                                                                                                                                                                                                                                                                                                                                                                                                                                                                                                                                                                                                                                                                                                                                                                                                                                                                                                                                                                                                                                                                                                                                        2020-08-25T01:09:57.747473  image/svg+xml    Matplotlib v3.3.1, https://matplotlib.org/                                                                                                                                                                                                                                                                                                                                                                                                                                                                                                                                                                                                                                                                                                                                                                                                                                                                                                                                                                                                                                                                                                                                                                                                                                                                                                                                                                                                                                                                                                                          2020-08-25T01:09:57.898559  image/svg+xml    Matplotlib v3.3.1, https://matplotlib.org/                                                                                                                                                                                                                                                                                                                                                                                                                                                                                                                                                                                                                                                                                                                                                                                                                                                                                                                                                                                                                                                                                                                                                                                                                                                                                                                                                                                                                                                                                              2020-08-25T01:09:58.043490  image/svg+xml    Matplotlib v3.3.1, https://matplotlib.org/                                                                                                                                                                                                                                                                                                                                                                                                                                                                                                                                                                                                                                                                                                                                                                                                                                                                                                                                                                                                                                                                                                                                                                                                                                                                                                                                                                                                                                                                  2020-08-25T01:09:58.187760  image/svg+xml    Matplotlib v3.3.1, https://matplotlib.org/                                                                                                                                                                                                                                                                                                                                                                                                                                                                                                                                                                                                                                                                                                                                                                                                                                                                                                                                                                                                                                                                                                                                                                                                                                                                                                                                                                                                                                                                                                                              2020-08-25T01:09:58.342581  image/svg+xml    Matplotlib v3.3.1, https://matplotlib.org/                                                                                                                                                                                                                                                                                                                                                                                                                                                                                                                                                                                                                                                                                                                                                                                                                                                                                                                                                                                                                                                                                                                                                                                                                                                                                                                                                                                                                                                                                              2020-08-25T01:09:58.490041  image/svg+xml    Matplotlib v3.3.1, https://matplotlib.org/                                                                                                                                                                                                                                                                                                                                                                                                                                                                                                                                                                                                                                                                                                                                                                                                                                                                                                                                                                                                                                                                                                                                                                                                                                                                                                                                                                                                                                                                                                          2020-08-25T01:09:58.648177  image/svg+xml    Matplotlib v3.3.1, https://matplotlib.org/                                                                                                                                                                                                                                                                                                                                                                                                                                                                                                                                                                                                                                                                                                                                                                                                                                                                                                                                                                                                                                                                                                                                                                                                                                                                                                                                                                                                                                                                                         29   17   25   19   10   11   30   26   14   2   6   18   7   15   13   24   3   4   27              2020-08-25T01:09:58.789725  image/svg+xml    Matplotlib v3.3.1, https://matplotlib.org/                                                                                                                                                                                                                                                                                                                                                                                                                                                                                                                                                                                                                                                                                                                                                                                                                                                                                                                                                                                                                                                                                                                                                                                                                                                                                                                                                                                                                                                                                                                            2020-08-25T01:09:58.942533  image/svg+xml    Matplotlib v3.3.1, https://matplotlib.org/                                                                                                                                                                                                                                                                                                                                                                                                                                                                                                                                                                                                                                                                                                                                                                                                                                                                                                                                                                                                                                                                                                                                                                                                                                                                                                                                                                                                                                                                                                                                                  2020-08-25T01:09:59.102990  image/svg+xml    Matplotlib v3.3.1, https://matplotlib.org/                                                                                                                                                                                                                                                                                                                                                                                                                                                                                                                                                                                                                                                                                                                                                                                                                                                                                                                                                                                                                                                                                                                                                                                                                                                                                                                                                                                                                                                                                                                                        2020-08-25T01:09:59.259591  image/svg+xml    Matplotlib v3.3.1, https://matplotlib.org/                                                                                                                                                                                                                                                                                                                                                                                                                                                                                                                                                                                                                                                                                                                                                                                                                                                                                                                                                                                                                                                                                                                                                                                                                                                                                                                                                                                                                                                                                                              2020-08-25T01:09:59.404271  image/svg+xml    Matplotlib v3.3.1, https://matplotlib.org/                                                                                                                                                                                                                                                                                                                                                                                                                                                                                                                                                                                                                                                                                                                                                                                                                                                                                                                                                                                                                                                                                                                                                                                                                                                                                                                                                                                                                                                                                                                                                                          2020-08-25T01:09:59.569163  image/svg+xml    Matplotlib v3.3.1, https://matplotlib.org/                                                                                                                                                                                                                                                                                                                                                                                                                                                                                                                                                                                                                                                                                                                                                                                                                                                                                                                                                                                                                                                                                                                                                                                                                                                                                                                                                                                                                                                                                                            2020-08-25T01:09:59.712291  image/svg+xml    Matplotlib v3.3.1, https://matplotlib.org/                                                                                                                                                                                                                                                                                                                                                                                                                                                                                                                                                                                                                                                                                                                                                                                                                                                                                                                                                                                                                                                                                                                                                                                                                                                                                                                                                                                                                                                                                      2020-08-25T01:09:59.854681  image/svg+xml    Matplotlib v3.3.1, https://matplotlib.org/                                                                                                                                                                                                                                                                                                                                                                                                                                                                                                                                                                                                                                                                                                                                                                                                                                                                                                                                                                                                                                                                                                                                                                                                                                                                                                                                                                                                                                                                                                                                            2020-08-25T01:10:00.010264  image/svg+xml    Matplotlib v3.3.1, https://matplotlib.org/                                                                                                                                                                                                                                                                                                                                                                                                                                                                                                                                                                                                                                                                                                                                                                                                                                                                                                                                                                                                                                                                                                                                                                                                                                                                                                                                                                                                                                                                2020-08-25T01:10:00.154666  image/svg+xml    Matplotlib v3.3.1, https://matplotlib.org/                                                                                                                                                                                                                                                                                                                                                                                                                                                                                                                                                                                                                                                                                                                                                                                                                                                                                                                                                                                                                                                                                                                                                                                                                                                                                                                                                                                                                                                        2020-08-25T01:10:00.291073  image/svg+xml    Matplotlib v3.3.1, https://matplotlib.org/                                                                                                                                                                                                                                                                                                                                                                                                                                                                                                                                                                                                                                                                                                                                                                                                                                                                                                                                                                                                                                                                                                                                                                                                                                                                                                                                                                                                                                                                                                              2020-08-25T01:10:00.437382  image/svg+xml    Matplotlib v3.3.1, https://matplotlib.org/                                                                                                                                                                                                                                                                                                                                                                                                                                                                                                                                                                                                                                                                                                                                                                                                                                                                                                                                                                                                                                                                                                                                                                                                                                                                                                                                                                                                                                                                                                                                                        2020-08-25T01:10:00.597422  image/svg+xml    Matplotlib v3.3.1, https://matplotlib.org/                                                                                                                                                                                                                                                                                                                                                                                                                                                                                                                                                                                                                                                                                                                                                                                                                                                                                                                                                                                                                                                                                                                                                                                                                                                                                                                                                                                                                                                                                                                          2020-08-25T01:10:00.751566  image/svg+xml    Matplotlib v3.3.1, https://matplotlib.org/                                                                                                                                                                                                                                                                                                                                                                                                                                                                                                                                                                                                                                                                                                                                                                                                                                                                                                                                                                                                                                                                                                                                                                                                                                                                                                                                                                                                                                                                                      2020-08-25T01:10:00.898379  image/svg+xml    Matplotlib v3.3.1, https://matplotlib.org/                                                                                                                                                                                                                                                                                                                                                                                                                                                                                                                                                                                                                                                                                                                                                                                                                                                                                                                                                                                                                                                                                                                                                                                                                                                                                                                                                                                                                                                          2020-08-25T01:10:01.038711  image/svg+xml    Matplotlib v3.3.1, https://matplotlib.org/                                                                                                                                                                                                                                                                                                                                                                                                                                                                                                                                                                                                                                                                                                                                                                                                                                                                                                                                                                                                                                                                                                                                                                                                                                                                                                                                                                                                                                                                                                                              2020-08-25T01:10:01.201057  image/svg+xml    Matplotlib v3.3.1, https://matplotlib.org/                                                                                                                                                                                                                                                                                                                                                                                                                                                                                                                                                                                                                                                                                                                                                                                                                                                                                                                                                                                                                                                                                                                                                                                                                                                                                                                                                                                                                                                                                      2020-08-25T01:10:01.356527  image/svg+xml    Matplotlib v3.3.1, https://matplotlib.org/                                                                                                                                                                                                                                                                                                                                                                                                                                                                                                                                                                                                                                                                                                                                                                                                                                                                                                                                                                                                                                                                                                                                                                                                                                                                                                                                                                                                                                                                                                                  2020-08-25T01:10:01.511997  image/svg+xml    Matplotlib v3.3.1, https://matplotlib.org/                                                                                                                                                                                                                                                                                                                                                                                                                                                                                                                                                                                                                                                                                                                                                                                                                                                                                                                                                                                                                                                                                                                                                                                                                                                                                                                                                                                                                                                                                                         29   17   25   19   10   11   30   26   14   2   6   18   7   15   13   24   3   4   27              2020-08-25T01:10:01.842225  image/svg+xml    Matplotlib v3.3.1, https://matplotlib.org/                                                                                                                                                                                                                                                                                                                                                                                                                                                                                                                                                                                                                                                                                                                                                                                                                                                                                                                                                                                                                                                                                                                                                                                                                                                                                                                                                                                                                                                                                        2020-08-25T01:10:01.978590  image/svg+xml    Matplotlib v3.3.1, https://matplotlib.org/                                                                                                                                                                                                                                                                                                                                                                                                                                                                                                                                                                                                                                                                                                                                                                                                                                                                                                                                                                                                                                                                                                                                                                                                                                                                                                                                                                                                                                                                                                                  2020-08-25T01:10:02.121286  image/svg+xml    Matplotlib v3.3.1, https://matplotlib.org/                                                                                                                                                                                                                                                                                                                                                                                                                                                                                                                                                                                                                                                                                                                                                                                                                                                                                                                                                                                                                                                                                                                                                                                                                                                                                                                                                                                                                                                                                                            2020-08-25T01:10:02.270990  image/svg+xml    Matplotlib v3.3.1, https://matplotlib.org/                                                                                                                                                                                                                                                                                                                                                                                                                                                                                                                                                                                                                                                                                                                                                                                                                                                                                                                                                                                                                                                                                                                                                                                                                                                                                                                                                                                                                                                                          2020-08-25T01:10:02.405873  image/svg+xml    Matplotlib v3.3.1, https://matplotlib.org/                                                                                                                                                                                                                                                                                                                                                                                                                                                                                                                                                                                                                                                                                                                                                                                                                                                                                                                                                                                                                                                                                                                                                                                                                                                                                                                                                                                                                                                                                                                                          2020-08-25T01:10:02.560752  image/svg+xml    Matplotlib v3.3.1, https://matplotlib.org/                                                                                                                                                                                                                                                                                                                                                                                                                                                                                                                                                                                                                                                                                                                                                                                                                                                                                                                                                                                                                                                                                                                                                                                                                                                                                                                                                                                                                                                                        2020-08-25T01:10:02.694399  image/svg+xml    Matplotlib v3.3.1, https://matplotlib.org/                                                                                                                                                                                                                                                                                                                                                                                                                                                                                                                                                                                                                                                                                                                                                                                                                                                                                                                                                                                                                                                                                                                                                                                                                                                                                                                                                                                                                                                      2020-08-25T01:10:02.827908  image/svg+xml    Matplotlib v3.3.1, https://matplotlib.org/                                                                                                                                                                                                                                                                                                                                                                                                                                                                                                                                                                                                                                                                                                                                                                                                                                                                                                                                                                                                                                                                                                                                                                                                                                                                                                                                                                                                                                                                                                              2020-08-25T01:10:02.969513  image/svg+xml    Matplotlib v3.3.1, https://matplotlib.org/                                                                                                                                                                                                                                                                                                                                                                                                                                                                                                                                                                                                                                                                                                                                                                                                                                                                                                                                                                                                                                                                                                                                                                                                                                                                                                                                                                                                                                    2020-08-25T01:10:03.101408  image/svg+xml    Matplotlib v3.3.1, https://matplotlib.org/                                                                                                                                                                                                                                                                                                                                                                                                                                                                                                                                                                                                                                                                                                                                                                                                                                                                                                                                                                                                                                                                                                                                                                                                                                                                                                                                                                                                                            2020-08-25T01:10:03.227646  image/svg+xml    Matplotlib v3.3.1, https://matplotlib.org/                                                                                                                                                                                                                                                                                                                                                                                                                                                                                                                                                                                                                                                                                                                                                                                                                                                                                                                                                                                                                                                                                                                                                                                                                                                                                                                                                                                                                                                                                2020-08-25T01:10:03.361852  image/svg+xml    Matplotlib v3.3.1, https://matplotlib.org/                                                                                                                                                                                                                                                                                                                                                                                                                                                                                                                                                                                                                                                                                                                                                                                                                                                                                                                                                                                                                                                                                                                                                                                                                                                                                                                                                                                                                                                                                                                        2020-08-25T01:10:03.506633  image/svg+xml    Matplotlib v3.3.1, https://matplotlib.org/                                                                                                                                                                                                                                                                                                                                                                                                                                                                                                                                                                                                                                                                                                                                                                                                                                                                                                                                                                                                                                                                                                                                                                                                                                                                                                                                                                                                                                                                                          2020-08-25T01:10:03.647225  image/svg+xml    Matplotlib v3.3.1, https://matplotlib.org/                                                                                                                                                                                                                                                                                                                                                                                                                                                                                                                                                                                                                                                                                                                                                                                                                                                                                                                                                                                                                                                                                                                                                                                                                                                                                                                                                                                                                                                          2020-08-25T01:10:03.788182  image/svg+xml    Matplotlib v3.3.1, https://matplotlib.org/                                                                                                                                                                                                                                                                                                                                                                                                                                                                                                                                                                                                                                                                                                                                                                                                                                                                                                                                                                                                                                                                                                                                                                                                                                                                                                                                                                                                                              2020-08-25T01:10:03.926276  image/svg+xml    Matplotlib v3.3.1, https://matplotlib.org/                                                                                                                                                                                                                                                                                                                                                                                                                                                                                                                                                                                                                                                                                                                                                                                                                                                                                                                                                                                                                                                                                                                                                                                                                                                                                                                                                                                                                                                                                                  2020-08-25T01:10:04.072546  image/svg+xml    Matplotlib v3.3.1, https://matplotlib.org/                                                                                                                                                                                                                                                                                                                                                                                                                                                                                                                                                                                                                                                                                                                                                                                                                                                                                                                                                                                                                                                                                                                                                                                                                                                                                                                                                                                                                                                          2020-08-25T01:10:04.208399  image/svg+xml    Matplotlib v3.3.1, https://matplotlib.org/                                                                                                                                                                                                                                                                                                                                                                                                                                                                                                                                                                                                                                                                                                                                                                                                                                                                                                                                                                                                                                                                                                                                                                                                                                                                                                                                                                                                                                                                                      2020-08-25T01:10:04.347317  image/svg+xml    Matplotlib v3.3.1, https://matplotlib.org/                                                                                                                                                                                                                                                                                                                                                                                                                                                                                                                                                                                                                                                                                                                                                                                                                                                                                                                                                                                                                                                                                                                                                                                                                                                                                                                                                                                                                                                                         29   17   25   19   10   11   30   26   14   2   6   18   7   15   13   24   3   4   27              2020-08-25T01:10:04.482879  image/svg+xml    Matplotlib v3.3.1, https://matplotlib.org/                                                                                                                                                                                                                                                                                                                                                                                                                                                                                                                                                                                                                                                                                                                                                                                                                                                                                                                                                                                                                                                                                                                                                                                                                                                                                                                                                                                                                                                                                                                                                2020-08-25T01:10:04.638905  image/svg+xml    Matplotlib v3.3.1, https://matplotlib.org/                                                                                                                                                                                                                                                                                                                                                                                                                                                                                                                                                                                                                                                                                                                                                                                                                                                                                                                                                                                                                                                                                                                                                                                                                                                                                                                                                                                                                                                                                                                                                                      2020-08-25T01:10:04.802554  image/svg+xml    Matplotlib v3.3.1, https://matplotlib.org/                                                                                                                                                                                                                                                                                                                                                                                                                                                                                                                                                                                                                                                                                                                                                                                                                                                                                                                                                                                                                                                                                                                                                                                                                                                                                                                                                                                                                                                                                                                                                                    2020-08-25T01:10:04.966497  image/svg+xml    Matplotlib v3.3.1, https://matplotlib.org/                                                                                                                                                                                                                                                                                                                                                                                                                                                                                                                                                                                                                                                                                                                                                                                                                                                                                                                                                                                                                                                                                                                                                                                                                                                                                                                                                                                                                                                                                                                                      2020-08-25T01:10:05.115145  image/svg+xml    Matplotlib v3.3.1, https://matplotlib.org/                                                                                                                                                                                                                                                                                                                                                                                                                                                                                                                                                                                                                                                                                                                                                                                                                                                                                                                                                                                                                                                                                                                                                                                                                                                                                                                                                                                                                                                                                                                                                                                          2020-08-25T01:10:05.276994  image/svg+xml    Matplotlib v3.3.1, https://matplotlib.org/                                                                                                                                                                                                                                                                                                                                                                                                                                                                                                                                                                                                                                                                                                                                                                                                                                                                                                                                                                                                                                                                                                                                                                                                                                                                                                                                                                                                                                                                                                                            2020-08-25T01:10:05.425314  image/svg+xml    Matplotlib v3.3.1, https://matplotlib.org/                                                                                                                                                                                                                                                                                                                                                                                                                                                                                                                                                                                                                                                                                                                                                                                                                                                                                                                                                                                                                                                                                                                                                                                                                                                                                                                                                                                                                                                                                                              2020-08-25T01:10:05.572428  image/svg+xml    Matplotlib v3.3.1, https://matplotlib.org/                                                                                                                                                                                                                                                                                                                                                                                                                                                                                                                                                                                                                                                                                                                                                                                                                                                                                                                                                                                                                                                                                                                                                                                                                                                                                                                                                                                                                                                                                                                                                                      2020-08-25T01:10:05.733154  image/svg+xml    Matplotlib v3.3.1, https://matplotlib.org/                                                                                                                                                                                                                                                                                                                                                                                                                                                                                                                                                                                                                                                                                                                                                                                                                                                                                                                                                                                                                                                                                                                                                                                                                                                                                                                                                                                                                                                                                            2020-08-25T01:10:05.882707  image/svg+xml    Matplotlib v3.3.1, https://matplotlib.org/                                                                                                                                                                                                                                                                                                                                                                                                                                                                                                                                                                                                                                                                                                                                                                                                                                                                                                                                                                                                                                                                                                                                                                                                                                                                                                                                                                                                                                                                                2020-08-25T01:10:06.028904  image/svg+xml    Matplotlib v3.3.1, https://matplotlib.org/                                                                                                                                                                                                                                                                                                                                                                                                                                                                                                                                                                                                                                                                                                                                                                                                                                                                                                                                                                                                                                                                                                                                                                                                                                                                                                                                                                                                                                                                                                                                        2020-08-25T01:10:06.360388  image/svg+xml    Matplotlib v3.3.1, https://matplotlib.org/                                                                                                                                                                                                                                                                                                                                                                                                                                                                                                                                                                                                                                                                                                                                                                                                                                                                                                                                                                                                                                                                                                                                                                                                                                                                                                                                                                                                                                                                                                                                                                                2020-08-25T01:10:06.522438  image/svg+xml    Matplotlib v3.3.1, https://matplotlib.org/                                                                                                                                                                                                                                                                                                                                                                                                                                                                                                                                                                                                                                                                                                                                                                                                                                                                                                                                                                                                                                                                                                                                                                                                                                                                                                                                                                                                                                                                                                                                          2020-08-25T01:10:06.677005  image/svg+xml    Matplotlib v3.3.1, https://matplotlib.org/                                                                                                                                                                                                                                                                                                                                                                                                                                                                                                                                                                                                                                                                                                                                                                                                                                                                                                                                                                                                                                                                                                                                                                                                                                                                                                                                                                                                                                                                                                                  2020-08-25T01:10:06.827869  image/svg+xml    Matplotlib v3.3.1, https://matplotlib.org/                                                                                                                                                                                                                                                                                                                                                                                                                                                                                                                                                                                                                                                                                                                                                                                                                                                                                                                                                                                                                                                                                                                                                                                                                                                                                                                                                                                                                                                                                      2020-08-25T01:10:06.973134  image/svg+xml    Matplotlib v3.3.1, https://matplotlib.org/                                                                                                                                                                                                                                                                                                                                                                                                                                                                                                                                                                                                                                                                                                                                                                                                                                                                                                                                                                                                                                                                                                                                                                                                                                                                                                                                                                                                                                                                                                                                                      2020-08-25T01:10:07.134512  image/svg+xml    Matplotlib v3.3.1, https://matplotlib.org/                                                                                                                                                                                                                                                                                                                                                                                                                                                                                                                                                                                                                                                                                                                                                                                                                                                                                                                                                                                                                                                                                                                                                                                                                                                                                                                                                                                                                                                                                                                  2020-08-25T01:10:07.288873  image/svg+xml    Matplotlib v3.3.1, https://matplotlib.org/                                                                                                                                                                                                                                                                                                                                                                                                                                                                                                                                                                                                                                                                                                                                                                                                                                                                                                                                                                                                                                                                                                                                                                                                                                                                                                                                                                                                                                                                                                                                              2020-08-25T01:10:07.442357  image/svg+xml    Matplotlib v3.3.1, https://matplotlib.org/                                                                                                                                                                                                                                                                                                                                                                                                                                                                                                                                                                                                                                                                                                                                                                                                                                                                                                                                                                                                                                                                                                                                                                                                                                                                                                                                                                                                                                                                                                                             29   17   25   19   10   11   30   26   14   2   6   18   7   15   13   24   3   4   27              2020-08-25T01:10:07.599109  image/svg+xml    Matplotlib v3.3.1, https://matplotlib.org/                                                                                                                                                                                                                                                                                                                                                                                                                                                                                                                                                                                                                                                                                                                                                                                                                                                                                                                                                                                                                                                                                                                                                                                                                                                                                                                                                                                                                                                                                2020-08-25T01:10:07.731852  image/svg+xml    Matplotlib v3.3.1, https://matplotlib.org/                                                                                                                                                                                                                                                                                                                                                                                                                                                                                                                                                                                                                                                                                                                                                                                                                                                                                                                                                                                                                                                                                                                                                                                                                                                                                                                                                                                                                                                                                              2020-08-25T01:10:07.875929  image/svg+xml    Matplotlib v3.3.1, https://matplotlib.org/                                                                                                                                                                                                                                                                                                                                                                                                                                                                                                                                                                                                                                                                                                                                                                                                                                                                                                                                                                                                                                                                                                                                                                                                                                                                                                                                                                                                                                                                                                        2020-08-25T01:10:08.015105  image/svg+xml    Matplotlib v3.3.1, https://matplotlib.org/                                                                                                                                                                                                                                                                                                                                                                                                                                                                                                                                                                                                                                                                                                                                                                                                                                                                                                                                                                                                                                                                                                                                                                                                                                                                                                                                                                                                                                                                      2020-08-25T01:10:08.157906  image/svg+xml    Matplotlib v3.3.1, https://matplotlib.org/                                                                                                                                                                                                                                                                                                                                                                                                                                                                                                                                                                                                                                                                                                                                                                                                                                                                                                                                                                                                                                                                                                                                                                                                                                                                                                                                                                                                                                                                                                                              2020-08-25T01:10:08.300905  image/svg+xml    Matplotlib v3.3.1, https://matplotlib.org/                                                                                                                                                                                                                                                                                                                                                                                                                                                                                                                                                                                                                                                                                                                                                                                                                                                                                                                                                                                                                                                                                                                                                                                                                                                                                                                                                                                                                                            2020-08-25T01:10:08.426629  image/svg+xml    Matplotlib v3.3.1, https://matplotlib.org/                                                                                                                                                                                                                                                                                                                                                                                                                                                                                                                                                                                                                                                                                                                                                                                                                                                                                                                                                                                                                                                                                                                                                                                                                                                                                                                                                                                                                                              2020-08-25T01:10:08.551147  image/svg+xml    Matplotlib v3.3.1, https://matplotlib.org/                                                                                                                                                                                                                                                                                                                                                                                                                                                                                                                                                                                                                                                                                                                                                                                                                                                                                                                                                                                                                                                                                                                                                                                                                                                                                                                                                                                                                                                                                              2020-08-25T01:10:08.688620  image/svg+xml    Matplotlib v3.3.1, https://matplotlib.org/                                                                                                                                                                                                                                                                                                                                                                                                                                                                                                                                                                                                                                                                                                                                                                                                                                                                                                                                                                                                                                                                                                                                                                                                                                                                                                                                                                                                                            2020-08-25T01:10:08.820549  image/svg+xml    Matplotlib v3.3.1, https://matplotlib.org/                                                                                                                                                                                                                                                                                                                                                                                                                                                                                                                                                                                                                                                                                                                                                                                                                                                                                                                                                                                                                                                                                                                                                                                                                                                                                                                                                                                                                2020-08-25T01:10:08.940869  image/svg+xml    Matplotlib v3.3.1, https://matplotlib.org/                                                                                                                                                                                                                                                                                                                                                                                                                                                                                                                                                                                                                                                                                                                                                                                                                                                                                                                                                                                                                                                                                                                                                                                                                                                                                                                                                                                                                                                                2020-08-25T01:10:09.068668  image/svg+xml    Matplotlib v3.3.1, https://matplotlib.org/                                                                                                                                                                                                                                                                                                                                                                                                                                                                                                                                                                                                                                                                                                                                                                                                                                                                                                                                                                                                                                                                                                                                                                                                                                                                                                                                                                                                                                                                                                                2020-08-25T01:10:09.208605  image/svg+xml    Matplotlib v3.3.1, https://matplotlib.org/                                                                                                                                                                                                                                                                                                                                                                                                                                                                                                                                                                                                                                                                                                                                                                                                                                                                                                                                                                                                                                                                                                                                                                                                                                                                                                                                                                                                                                                                              2020-08-25T01:10:09.340203  image/svg+xml    Matplotlib v3.3.1, https://matplotlib.org/                                                                                                                                                                                                                                                                                                                                                                                                                                                                                                                                                                                                                                                                                                                                                                                                                                                                                                                                                                                                                                                                                                                                                                                                                                                                                                                                                                                                                                                      2020-08-25T01:10:09.468929  image/svg+xml    Matplotlib v3.3.1, https://matplotlib.org/                                                                                                                                                                                                                                                                                                                                                                                                                                                                                                                                                                                                                                                                                                                                                                                                                                                                                                                                                                                                                                                                                                                                                                                                                                                                                                                                                                                                                          2020-08-25T01:10:09.592520  image/svg+xml    Matplotlib v3.3.1, https://matplotlib.org/                                                                                                                                                                                                                                                                                                                                                                                                                                                                                                                                                                                                                                                                                                                                                                                                                                                                                                                                                                                                                                                                                                                                                                                                                                                                                                                                                                                                                                                                                      2020-08-25T01:10:09.728691  image/svg+xml    Matplotlib v3.3.1, https://matplotlib.org/                                                                                                                                                                                                                                                                                                                                                                                                                                                                                                                                                                                                                                                                                                                                                                                                                                                                                                                                                                                                                                                                                                                                                                                                                                                                                                                                                                                                                                                      2020-08-25T01:10:09.861837  image/svg+xml    Matplotlib v3.3.1, https://matplotlib.org/                                                                                                                                                                                                                                                                                                                                                                                                                                                                                                                                                                                                                                                                                                                                                                                                                                                                                                                                                                                                                                                                                                                                                                                                                                                                                                                                                                                                                                                                      2020-08-25T01:10:09.995550  image/svg+xml    Matplotlib v3.3.1, https://matplotlib.org/                                                                                                                                                                                                                                                                                                                                                                                                                                                                                                                                                                                                                                                                                                                                                                                                                                                                                                                                                                                                                                                                                                                                                                                                                                                                                                                                                                                                                                                     29   17   25   19   10   11   30   26   14   2   6   18   7   15   13   24   3   4   27              2020-08-25T01:10:10.123914  image/svg+xml    Matplotlib v3.3.1, https://matplotlib.org/                                                                                                                                                                                                                                                                                                                                                                                                                                                                                                                                                                                                                                                                                                                                                                                                                                                                                                                                                                                                                                                                                                                                                                                                                                                                                                                                                                                                                                                                  2020-08-25T01:10:10.254428  image/svg+xml    Matplotlib v3.3.1, https://matplotlib.org/                                                                                                                                                                                                                                                                                                                                                                                                                                                                                                                                                                                                                                                                                                                                                                                                                                                                                                                                                                                                                                                                                                                                                                                                                                                                                                                                                                                                                                                                                    2020-08-25T01:10:10.390904  image/svg+xml    Matplotlib v3.3.1, https://matplotlib.org/                                                                                                                                                                                                                                                                                                                                                                                                                                                                                                                                                                                                                                                                                                                                                                                                                                                                                                                                                                                                                                                                                                                                                                                                                                                                                                                                                                                                                                                                                  2020-08-25T01:10:10.529457  image/svg+xml    Matplotlib v3.3.1, https://matplotlib.org/                                                                                                                                                                                                                                                                                                                                                                                                                                                                                                                                                                                                                                                                                                                                                                                                                                                                                                                                                                                                                                                                                                                                                                                                                                                                                                                                                                                                                                                    2020-08-25T01:10:10.837866  image/svg+xml    Matplotlib v3.3.1, https://matplotlib.org/                                                                                                                                                                                                                                                                                                                                                                                                                                                                                                                                                                                                                                                                                                                                                                                                                                                                                                                                                                                                                                                                                                                                                                                                                                                                                                                                                                                                                                                                                                                2020-08-25T01:10:10.980797  image/svg+xml    Matplotlib v3.3.1, https://matplotlib.org/                                                                                                                                                                                                                                                                                                                                                                                                                                                                                                                                                                                                                                                                                                                                                                                                                                                                                                                                                                                                                                                                                                                                                                                                                                                                                                                                                                                                                                              2020-08-25T01:10:11.104656  image/svg+xml    Matplotlib v3.3.1, https://matplotlib.org/                                                                                                                                                                                                                                                                                                                                                                                                                                                                                                                                                                                                                                                                                                                                                                                                                                                                                                                                                                                                                                                                                                                                                                                                                                                                                                                                                                                                                    2020-08-25T01:10:11.229109  image/svg+xml    Matplotlib v3.3.1, https://matplotlib.org/                                                                                                                                                                                                                                                                                                                                                                                                                                                                                                                                                                                                                                                                                                                                                                                                                                                                                                                                                                                                                                                                                                                                                                                                                                                                                                                                                                                                                                                                                2020-08-25T01:10:11.366516  image/svg+xml    Matplotlib v3.3.1, https://matplotlib.org/                                                                                                                                                                                                                                                                                                                                                                                                                                                                                                                                                                                                                                                                                                                                                                                                                                                                                                                                                                                                                                                                                                                                                                                                                                                                                                                                                                                                          2020-08-25T01:10:11.490498  image/svg+xml    Matplotlib v3.3.1, https://matplotlib.org/                                                                                                                                                                                                                                                                                                                                                                                                                                                                                                                                                                                                                                                                                                                                                                                                                                                                                                                                                                                                                                                                                                                                                                                                                                                                                                                                                                                                  2020-08-25T01:10:11.612928  image/svg+xml    Matplotlib v3.3.1, https://matplotlib.org/                                                                                                                                                                                                                                                                                                                                                                                                                                                                                                                                                                                                                                                                                                                                                                                                                                                                                                                                                                                                                                                                                                                                                                                                                                                                                                                                                                                                                                                  2020-08-25T01:10:11.746023  image/svg+xml    Matplotlib v3.3.1, https://matplotlib.org/                                                                                                                                                                                                                                                                                                                                                                                                                                                                                                                                                                                                                                                                                                                                                                                                                                                                                                                                                                                                                                                                                                                                                                                                                                                                                                                                                                                                                                                                                              2020-08-25T01:10:11.908044  image/svg+xml    Matplotlib v3.3.1, https://matplotlib.org/                                                                                                                                                                                                                                                                                                                                                                                                                                                                                                                                                                                                                                                                                                                                                                                                                                                                                                                                                                                                                                                                                                                                                                                                                                                                                                                                                                                                                                                                2020-08-25T01:10:12.041982  image/svg+xml    Matplotlib v3.3.1, https://matplotlib.org/                                                                                                                                                                                                                                                                                                                                                                                                                                                                                                                                                                                                                                                                                                                                                                                                                                                                                                                                                                                                                                                                                                                                                                                                                                                                                                                                                                                                                                2020-08-25T01:10:12.170514  image/svg+xml    Matplotlib v3.3.1, https://matplotlib.org/                                                                                                                                                                                                                                                                                                                                                                                                                                                                                                                                                                                                                                                                                                                                                                                                                                                                                                                                                                                                                                                                                                                                                                                                                                                                                                                                                                                                    2020-08-25T01:10:12.296961  image/svg+xml    Matplotlib v3.3.1, https://matplotlib.org/                                                                                                                                                                                                                                                                                                                                                                                                                                                                                                                                                                                                                                                                                                                                                                                                                                                                                                                                                                                                                                                                                                                                                                                                                                                                                                                                                                                                                                                                        2020-08-25T01:10:12.438148  image/svg+xml    Matplotlib v3.3.1, https://matplotlib.org/                                                                                                                                                                                                                                                                                                                                                                                                                                                                                                                                                                                                                                                                                                                                                                                                                                                                                                                                                                                                                                                                                                                                                                                                                                                                                                                                                                                                                                2020-08-25T01:10:12.570472  image/svg+xml    Matplotlib v3.3.1, https://matplotlib.org/                                                                                                                                                                                                                                                                                                                                                                                                                                                                                                                                                                                                                                                                                                                                                                                                                                                                                                                                                                                                                                                                                                                                                                                                                                                                                                                                                                                                                                                        2020-08-25T01:10:12.706421  image/svg+xml    Matplotlib v3.3.1, https://matplotlib.org/                                                                                                                                                                                                                                                                                                                                                                                                                                                                                                                                                                                                                                                                                                                                                                                                                                                                                                                                                                                                                                                                                                                                                                                                                                                                                                                                                                                                                                           29   17   25   19   10   11   30   26   14   2   6   18   7   15   13   24   3   4   27              2020-08-25T01:10:12.830966  image/svg+xml    Matplotlib v3.3.1, https://matplotlib.org/                                                                                                                                                                                                                                                                                                                                                                                                                                                                                                                                                                                                                                                                                                                                                                                                                                                                                                                                                                                                                                                                                                                                                                                                                                                                                                                                                                                                                                                                                                                          2020-08-25T01:10:12.979324  image/svg+xml    Matplotlib v3.3.1, https://matplotlib.org/                                                                                                                                                                                                                                                                                                                                                                                                                                                                                                                                                                                                                                                                                                                                                                                                                                                                                                                                                                                                                                                                                                                                                                                                                                                                                                                                                                                                                                                                                                                                2020-08-25T01:10:13.125676  image/svg+xml    Matplotlib v3.3.1, https://matplotlib.org/                                                                                                                                                                                                                                                                                                                                                                                                                                                                                                                                                                                                                                                                                                                                                                                                                                                                                                                                                                                                                                                                                                                                                                                                                                                                                                                                                                                                                                                                                                                                          2020-08-25T01:10:13.280138  image/svg+xml    Matplotlib v3.3.1, https://matplotlib.org/                                                                                                                                                                                                                                                                                                                                                                                                                                                                                                                                                                                                                                                                                                                                                                                                                                                                                                                                                                                                                                                                                                                                                                                                                                                                                                                                                                                                                                                                                                            2020-08-25T01:10:13.427464  image/svg+xml    Matplotlib v3.3.1, https://matplotlib.org/                                                                                                                                                                                                                                                                                                                                                                                                                                                                                                                                                                                                                                                                                                                                                                                                                                                                                                                                                                                                                                                                                                                                                                                                                                                                                                                                                                                                                                                                                                                                                                        2020-08-25T01:10:13.580117  image/svg+xml    Matplotlib v3.3.1, https://matplotlib.org/                                                                                                                                                                                                                                                                                                                                                                                                                                                                                                                                                                                                                                                                                                                                                                                                                                                                                                                                                                                                                                                                                                                                                                                                                                                                                                                                                                                                                                                                                              2020-08-25T01:10:13.719396  image/svg+xml    Matplotlib v3.3.1, https://matplotlib.org/                                                                                                                                                                                                                                                                                                                                                                                                                                                                                                                                                                                                                                                                                                                                                                                                                                                                                                                                                                                                                                                                                                                                                                                                                                                                                                                                                                                                                                                                                2020-08-25T01:10:13.870798  image/svg+xml    Matplotlib v3.3.1, https://matplotlib.org/                                                                                                                                                                                                                                                                                                                                                                                                                                                                                                                                                                                                                                                                                                                                                                                                                                                                                                                                                                                                                                                                                                                                                                                                                                                                                                                                                                                                                                                                                                                            2020-08-25T01:10:14.022368  image/svg+xml    Matplotlib v3.3.1, https://matplotlib.org/                                                                                                                                                                                                                                                                                                                                                                                                                                                                                                                                                                                                                                                                                                                                                                                                                                                                                                                                                                                                                                                                                                                                                                                                                                                                                                                                                                                                                                                              2020-08-25T01:10:14.158549  image/svg+xml    Matplotlib v3.3.1, https://matplotlib.org/                                                                                                                                                                                                                                                                                                                                                                                                                                                                                                                                                                                                                                                                                                                                                                                                                                                                                                                                                                                                                                                                                                                                                                                                                                                                                                                                                                                                                                                      2020-08-25T01:10:14.291053  image/svg+xml    Matplotlib v3.3.1, https://matplotlib.org/                                                                                                                                                                                                                                                                                                                                                                                                                                                                                                                                                                                                                                                                                                                                                                                                                                                                                                                                                                                                                                                                                                                                                                                                                                                                                                                                                                                                                                                                                              2020-08-25T01:10:14.432328  image/svg+xml    Matplotlib v3.3.1, https://matplotlib.org/                                                                                                                                                                                                                                                                                                                                                                                                                                                                                                                                                                                                                                                                                                                                                                                                                                                                                                                                                                                                                                                                                                                                                                                                                                                                                                                                                                                                                                                                                                                                                  2020-08-25T01:10:14.588389  image/svg+xml    Matplotlib v3.3.1, https://matplotlib.org/                                                                                                                                                                                                                                                                                                                                                                                                                                                                                                                                                                                                                                                                                                                                                                                                                                                                                                                                                                                                                                                                                                                                                                                                                                                                                                                                                                                                                                                                                                                        2020-08-25T01:10:14.734765  image/svg+xml    Matplotlib v3.3.1, https://matplotlib.org/                                                                                                                                                                                                                                                                                                                                                                                                                                                                                                                                                                                                                                                                                                                                                                                                                                                                                                                                                                                                                                                                                                                                                                                                                                                                                                                                                                                                                                                                                        2020-08-25T01:10:14.881156  image/svg+xml    Matplotlib v3.3.1, https://matplotlib.org/                                                                                                                                                                                                                                                                                                                                                                                                                                                                                                                                                                                                                                                                                                                                                                                                                                                                                                                                                                                                                                                                                                                                                                                                                                                                                                                                                                                                                                                            2020-08-25T01:10:15.021121  image/svg+xml    Matplotlib v3.3.1, https://matplotlib.org/                                                                                                                                                                                                                                                                                                                                                                                                                                                                                                                                                                                                                                                                                                                                                                                                                                                                                                                                                                                                                                                                                                                                                                                                                                                                                                                                                                                                                                                                                                                            2020-08-25T01:10:15.356555  image/svg+xml    Matplotlib v3.3.1, https://matplotlib.org/                                                                                                                                                                                                                                                                                                                                                                                                                                                                                                                                                                                                                                                                                                                                                                                                                                                                                                                                                                                                                                                                                                                                                                                                                                                                                                                                                                                                                                                                                        2020-08-25T01:10:15.503584  image/svg+xml    Matplotlib v3.3.1, https://matplotlib.org/                                                                                                                                                                                                                                                                                                                                                                                                                                                                                                                                                                                                                                                                                                                                                                                                                                                                                                                                                                                                                                                                                                                                                                                                                                                                                                                                                                                                                                                                                                    2020-08-25T01:10:15.653818  image/svg+xml    Matplotlib v3.3.1, https://matplotlib.org/                                                                                                                                                                                                                                                                                                                                                                                                                                                                                                                                                                                                                                                                                                                                                                                                                                                                                                                                                                                                                                                                                                                                                                                                                                                                                                                                                                                                                                                                                       29   17   25   19   10   11   30   26   14   2   6   18   7   15   13   24   3   4   27              2020-08-25T01:10:15.800294  image/svg+xml    Matplotlib v3.3.1, https://matplotlib.org/                                                                                                                                                                                                                                                                                                                                                                                                                                                                                                                                                                                                                                                                                                                                                                                                                                                                                                                                                                                                                                                                                                                                                                                                                                                                                                                                                                                                                                                    2020-08-25T01:10:15.934023  image/svg+xml    Matplotlib v3.3.1, https://matplotlib.org/                                                                                                                                                                                                                                                                                                                                                                                                                                                                                                                                                                                                                                                                                                                                                                                                                                                                                                                                                                                                                                                                                                                                                                                                                                                                                                                                                                                                                                                                      2020-08-25T01:10:16.067049  image/svg+xml    Matplotlib v3.3.1, https://matplotlib.org/                                                                                                                                                                                                                                                                                                                                                                                                                                                                                                                                                                                                                                                                                                                                                                                                                                                                                                                                                                                                                                                                                                                                                                                                                                                                                                                                                                                                                                                                2020-08-25T01:10:16.206321  image/svg+xml    Matplotlib v3.3.1, https://matplotlib.org/                                                                                                                                                                                                                                                                                                                                                                                                                                                                                                                                                                                                                                                                                                                                                                                                                                                                                                                                                                                                                                                                                                                                                                                                                                                                                                                                                                                                                                      2020-08-25T01:10:16.334550  image/svg+xml    Matplotlib v3.3.1, https://matplotlib.org/                                                                                                                                                                                                                                                                                                                                                                                                                                                                                                                                                                                                                                                                                                                                                                                                                                                                                                                                                                                                                                                                                                                                                                                                                                                                                                                                                                                                                                                                                                  2020-08-25T01:10:16.479860  image/svg+xml    Matplotlib v3.3.1, https://matplotlib.org/                                                                                                                                                                                                                                                                                                                                                                                                                                                                                                                                                                                                                                                                                                                                                                                                                                                                                                                                                                                                                                                                                                                                                                                                                                                                                                                                                                                                                                2020-08-25T01:10:16.605237  image/svg+xml    Matplotlib v3.3.1, https://matplotlib.org/                                                                                                                                                                                                                                                                                                                                                                                                                                                                                                                                                                                                                                                                                                                                                                                                                                                                                                                                                                                                                                                                                                                                                                                                                                                                                                                                                                                                              2020-08-25T01:10:16.729543  image/svg+xml    Matplotlib v3.3.1, https://matplotlib.org/                                                                                                                                                                                                                                                                                                                                                                                                                                                                                                                                                                                                                                                                                                                                                                                                                                                                                                                                                                                                                                                                                                                                                                                                                                                                                                                                                                                                                                                                2020-08-25T01:10:16.867222  image/svg+xml    Matplotlib v3.3.1, https://matplotlib.org/                                                                                                                                                                                                                                                                                                                                                                                                                                                                                                                                                                                                                                                                                                                                                                                                                                                                                                                                                                                                                                                                                                                                                                                                                                                                                                                                                                                    2020-08-25T01:10:16.993620  image/svg+xml    Matplotlib v3.3.1, https://matplotlib.org/                                                                                                                                                                                                                                                                                                                                                                                                                                                                                                                                                                                                                                                                                                                                                                                                                                                                                                                                                                                                                                                                                                                                                                                                                                                                                                                                                                            2020-08-25T01:10:17.112556  image/svg+xml    Matplotlib v3.3.1, https://matplotlib.org/                                                                                                                                                                                                                                                                                                                                                                                                                                                                                                                                                                                                                                                                                                                                                                                                                                                                                                                                                                                                                                                                                                                                                                                                                                                                                                                                                                                                                                  2020-08-25T01:10:17.239949  image/svg+xml    Matplotlib v3.3.1, https://matplotlib.org/                                                                                                                                                                                                                                                                                                                                                                                                                                                                                                                                                                                                                                                                                                                                                                                                                                                                                                                                                                                                                                                                                                                                                                                                                                                                                                                                                                                                                                                                                2020-08-25T01:10:17.379019  image/svg+xml    Matplotlib v3.3.1, https://matplotlib.org/                                                                                                                                                                                                                                                                                                                                                                                                                                                                                                                                                                                                                                                                                                                                                                                                                                                                                                                                                                                                                                                                                                                                                                                                                                                                                                                                                                                                                                                  2020-08-25T01:10:17.511416  image/svg+xml    Matplotlib v3.3.1, https://matplotlib.org/                                                                                                                                                                                                                                                                                                                                                                                                                                                                                                                                                                                                                                                                                                                                                                                                                                                                                                                                                                                                                                                                                                                                                                                                                                                                                                                                                                                                              2020-08-25T01:10:17.635082  image/svg+xml    Matplotlib v3.3.1, https://matplotlib.org/                                                                                                                                                                                                                                                                                                                                                                                                                                                                                                                                                                                                                                                                                                                                                                                                                                                                                                                                                                                                                                                                                                                                                                                                                                                                                                                                                                                  2020-08-25T01:10:17.752090  image/svg+xml    Matplotlib v3.3.1, https://matplotlib.org/                                                                                                                                                                                                                                                                                                                                                                                                                                                                                                                                                                                                                                                                                                                                                                                                                                                                                                                                                                                                                                                                                                                                                                                                                                                                                                                                                                                                                                                  2020-08-25T01:10:17.890859  image/svg+xml    Matplotlib v3.3.1, https://matplotlib.org/                                                                                                                                                                                                                                                                                                                                                                                                                                                                                                                                                                                                                                                                                                                                                                                                                                                                                                                                                                                                                                                                                                                                                                                                                                                                                                                                                                                                              2020-08-25T01:10:18.017261  image/svg+xml    Matplotlib v3.3.1, https://matplotlib.org/                                                                                                                                                                                                                                                                                                                                                                                                                                                                                                                                                                                                                                                                                                                                                                                                                                                                                                                                                                                                                                                                                                                                                                                                                                                                                                                                                                                                                                      2020-08-25T01:10:18.151890  image/svg+xml    Matplotlib v3.3.1, https://matplotlib.org/                                                                                                                                                                                                                                                                                                                                                                                                                                                                                                                                                                                                                                                                                                                                                                                                                                                                                                                                                                                                                                                                                                                                                                                                                                                                                                                                                                                                                             29   17   25   19   10   11   30   26   14   2   6   18   7   15   13   24   3   4   27              2020-08-25T01:10:18.273870  image/svg+xml    Matplotlib v3.3.1, https://matplotlib.org/                                                                                                                                                                                                                                                                                                                                                                                                                                                                                                                                                                                                                                                                                                                                                                                                                                                                                                                                                                                                                                                                                                                                                                                                                                                                                                                                                                                                                                      2020-08-25T01:10:18.394987  image/svg+xml    Matplotlib v3.3.1, https://matplotlib.org/                                                                                                                                                                                                                                                                                                                                                                                                                                                                                                                                                                                                                                                                                                                                                                                                                                                                                                                                                                                                                                                                                                                                                                                                                                                                                                                                                                                                                                                            2020-08-25T01:10:18.525578  image/svg+xml    Matplotlib v3.3.1, https://matplotlib.org/                                                                                                                                                                                                                                                                                                                                                                                                                                                                                                                                                                                                                                                                                                                                                                                                                                                                                                                                                                                                                                                                                                                                                                                                                                                                                                                                                                                                                                                      2020-08-25T01:10:18.653006  image/svg+xml    Matplotlib v3.3.1, https://matplotlib.org/                                                                                                                                                                                                                                                                                                                                                                                                                                                                                                                                                                                                                                                                                                                                                                                                                                                                                                                                                                                                                                                                                                                                                                                                                                                                                                                                                                                                                            2020-08-25T01:10:18.780266  image/svg+xml    Matplotlib v3.3.1, https://matplotlib.org/                                                                                                                                                                                                                                                                                                                                                                                                                                                                                                                                                                                                                                                                                                                                                                                                                                                                                                                                                                                                                                                                                                                                                                                                                                                                                                                                                                                                                                                                                    2020-08-25T01:10:18.915427  image/svg+xml    Matplotlib v3.3.1, https://matplotlib.org/                                                                                                                                                                                                                                                                                                                                                                                                                                                                                                                                                                                                                                                                                                                                                                                                                                                                                                                                                                                                                                                                                                                                                                                                                                                                                                                                                                                                                  2020-08-25T01:10:19.034005  image/svg+xml    Matplotlib v3.3.1, https://matplotlib.org/                                                                                                                                                                                                                                                                                                                                                                                                                                                                                                                                                                                                                                                                                                                                                                                                                                                                                                                                                                                                                                                                                                                                                                                                                                                                                                                                                                                                    2020-08-25T01:10:19.150361  image/svg+xml    Matplotlib v3.3.1, https://matplotlib.org/                                                                                                                                                                                                                                                                                                                                                                                                                                                                                                                                                                                                                                                                                                                                                                                                                                                                                                                                                                                                                                                                                                                                                                                                                                                                                                                                                                                                                                                      2020-08-25T01:10:19.276556  image/svg+xml    Matplotlib v3.3.1, https://matplotlib.org/                                                                                                                                                                                                                                                                                                                                                                                                                                                                                                                                                                                                                                                                                                                                                                                                                                                                                                                                                                                                                                                                                                                                                                                                                                                                                                                                                                          2020-08-25T01:10:19.392716  image/svg+xml    Matplotlib v3.3.1, https://matplotlib.org/                                                                                                                                                                                                                                                                                                                                                                                                                                                                                                                                                                                                                                                                                                                                                                                                                                                                                                                                                                                                                                                                                                                                                                                                                                                                                                                                                              2020-08-25T01:10:19.683994  image/svg+xml    Matplotlib v3.3.1, https://matplotlib.org/                                                                                                                                                                                                                                                                                                                                                                                                                                                                                                                                                                                                                                                                                                                                                                                                                                                                                                                                                                                                                                                                                                                                                                                                                                                                                                                                                                                                                        2020-08-25T01:10:19.808342  image/svg+xml    Matplotlib v3.3.1, https://matplotlib.org/                                                                                                                                                                                                                                                                                                                                                                                                                                                                                                                                                                                                                                                                                                                                                                                                                                                                                                                                                                                                                                                                                                                                                                                                                                                                                                                                                                                                                                                                      2020-08-25T01:10:19.938164  image/svg+xml    Matplotlib v3.3.1, https://matplotlib.org/                                                                                                                                                                                                                                                                                                                                                                                                                                                                                                                                                                                                                                                                                                                                                                                                                                                                                                                                                                                                                                                                                                                                                                                                                                                                                                                                                                                                                                    2020-08-25T01:10:20.063973  image/svg+xml    Matplotlib v3.3.1, https://matplotlib.org/                                                                                                                                                                                                                                                                                                                                                                                                                                                                                                                                                                                                                                                                                                                                                                                                                                                                                                                                                                                                                                                                                                                                                                                                                                                                                                                                                                                                    2020-08-25T01:10:20.184673  image/svg+xml    Matplotlib v3.3.1, https://matplotlib.org/                                                                                                                                                                                                                                                                                                                                                                                                                                                                                                                                                                                                                                                                                                                                                                                                                                                                                                                                                                                                                                                                                                                                                                                                                                                                                                                                                                        2020-08-25T01:10:20.296923  image/svg+xml    Matplotlib v3.3.1, https://matplotlib.org/                                                                                                                                                                                                                                                                                                                                                                                                                                                                                                                                                                                                                                                                                                                                                                                                                                                                                                                                                                                                                                                                                                                                                                                                                                                                                                                                                                                                                                        2020-08-25T01:10:20.424290  image/svg+xml    Matplotlib v3.3.1, https://matplotlib.org/                                                                                                                                                                                                                                                                                                                                                                                                                                                                                                                                                                                                                                                                                                                                                                                                                                                                                                                                                                                                                                                                                                                                                                                                                                                                                                                                                                                                    2020-08-25T01:10:20.550788  image/svg+xml    Matplotlib v3.3.1, https://matplotlib.org/                                                                                                                                                                                                                                                                                                                                                                                                                                                                                                                                                                                                                                                                                                                                                                                                                                                                                                                                                                                                                                                                                                                                                                                                                                                                                                                                                                                                                            2020-08-25T01:10:20.676725  image/svg+xml    Matplotlib v3.3.1, https://matplotlib.org/                                                                                                                                                                                                                                                                                                                                                                                                                                                                                                                                                                                                                                                                                                                                                                                                                                                                                                                                                                                                                                                                                                                                                                                                                                                                                                                                                                                                                   29   17   25   19   10   11   30   26   14   2   6   18   7   15   13   24   3   4   27              2020-08-25T01:10:20.794041  image/svg+xml    Matplotlib v3.3.1, https://matplotlib.org/                                                                                                                                                                                                                                                                                                                                                                                                                                                                                                                                                                                                                                                                                                                                                                                                                                                                                                                                                                                                                                                                                                                                                                                                                                                                                                                                                                                                                                                                                            2020-08-25T01:10:20.933560  image/svg+xml    Matplotlib v3.3.1, https://matplotlib.org/                                                                                                                                                                                                                                                                                                                                                                                                                                                                                                                                                                                                                                                                                                                                                                                                                                                                                                                                                                                                                                                                                                                                                                                                                                                                                                                                                                                                                                                                                                  2020-08-25T01:10:21.080075  image/svg+xml    Matplotlib v3.3.1, https://matplotlib.org/                                                                                                                                                                                                                                                                                                                                                                                                                                                                                                                                                                                                                                                                                                                                                                                                                                                                                                                                                                                                                                                                                                                                                                                                                                                                                                                                                                                                                                                                                                            2020-08-25T01:10:21.228466  image/svg+xml    Matplotlib v3.3.1, https://matplotlib.org/                                                                                                                                                                                                                                                                                                                                                                                                                                                                                                                                                                                                                                                                                                                                                                                                                                                                                                                                                                                                                                                                                                                                                                                                                                                                                                                                                                                                                                                                              2020-08-25T01:10:21.363634  image/svg+xml    Matplotlib v3.3.1, https://matplotlib.org/                                                                                                                                                                                                                                                                                                                                                                                                                                                                                                                                                                                                                                                                                                                                                                                                                                                                                                                                                                                                                                                                                                                                                                                                                                                                                                                                                                                                                                                                                                                                          2020-08-25T01:10:21.513971  image/svg+xml    Matplotlib v3.3.1, https://matplotlib.org/                                                                                                                                                                                                                                                                                                                                                                                                                                                                                                                                                                                                                                                                                                                                                                                                                                                                                                                                                                                                                                                                                                                                                                                                                                                                                                                                                                                                                                                                2020-08-25T01:10:21.645453  image/svg+xml    Matplotlib v3.3.1, https://matplotlib.org/                                                                                                                                                                                                                                                                                                                                                                                                                                                                                                                                                                                                                                                                                                                                                                                                                                                                                                                                                                                                                                                                                                                                                                                                                                                                                                                                                                                                                                                  2020-08-25T01:10:21.776138  image/svg+xml    Matplotlib v3.3.1, https://matplotlib.org/                                                                                                                                                                                                                                                                                                                                                                                                                                                                                                                                                                                                                                                                                                                                                                                                                                                                                                                                                                                                                                                                                                                                                                                                                                                                                                                                                                                                                                                                                              2020-08-25T01:10:21.917477  image/svg+xml    Matplotlib v3.3.1, https://matplotlib.org/                                                                                                                                                                                                                                                                                                                                                                                                                                                                                                                                                                                                                                                                                                                                                                                                                                                                                                                                                                                                                                                                                                                                                                                                                                                                                                                                                                                                                                2020-08-25T01:10:22.049072  image/svg+xml    Matplotlib v3.3.1, https://matplotlib.org/                                                                                                                                                                                                                                                                                                                                                                                                                                                                                                                                                                                                                                                                                                                                                                                                                                                                                                                                                                                                                                                                                                                                                                                                                                                                                                                                                                                                                        2020-08-25T01:10:22.177271  image/svg+xml    Matplotlib v3.3.1, https://matplotlib.org/                                                                                                                                                                                                                                                                                                                                                                                                                                                                                                                                                                                                                                                                                                                                                                                                                                                                                                                                                                                                                                                                                                                                                                                                                                                                                                                                                                                                                                                                2020-08-25T01:10:22.312151  image/svg+xml    Matplotlib v3.3.1, https://matplotlib.org/                                                                                                                                                                                                                                                                                                                                                                                                                                                                                                                                                                                                                                                                                                                                                                                                                                                                                                                                                                                                                                                                                                                                                                                                                                                                                                                                                                                                                                                                                                                    2020-08-25T01:10:22.454468  image/svg+xml    Matplotlib v3.3.1, https://matplotlib.org/                                                                                                                                                                                                                                                                                                                                                                                                                                                                                                                                                                                                                                                                                                                                                                                                                                                                                                                                                                                                                                                                                                                                                                                                                                                                                                                                                                                                                                                                                          2020-08-25T01:10:22.594274  image/svg+xml    Matplotlib v3.3.1, https://matplotlib.org/                                                                                                                                                                                                                                                                                                                                                                                                                                                                                                                                                                                                                                                                                                                                                                                                                                                                                                                                                                                                                                                                                                                                                                                                                                                                                                                                                                                                                                                          2020-08-25T01:10:22.726587  image/svg+xml    Matplotlib v3.3.1, https://matplotlib.org/                                                                                                                                                                                                                                                                                                                                                                                                                                                                                                                                                                                                                                                                                                                                                                                                                                                                                                                                                                                                                                                                                                                                                                                                                                                                                                                                                                                                                              2020-08-25T01:10:22.862430  image/svg+xml    Matplotlib v3.3.1, https://matplotlib.org/                                                                                                                                                                                                                                                                                                                                                                                                                                                                                                                                                                                                                                                                                                                                                                                                                                                                                                                                                                                                                                                                                                                                                                                                                                                                                                                                                                                                                                                                                              2020-08-25T01:10:23.005945  image/svg+xml    Matplotlib v3.3.1, https://matplotlib.org/                                                                                                                                                                                                                                                                                                                                                                                                                                                                                                                                                                                                                                                                                                                                                                                                                                                                                                                                                                                                                                                                                                                                                                                                                                                                                                                                                                                                                                                          2020-08-25T01:10:23.146686  image/svg+xml    Matplotlib v3.3.1, https://matplotlib.org/                                                                                                                                                                                                                                                                                                                                                                                                                                                                                                                                                                                                                                                                                                                                                                                                                                                                                                                                                                                                                                                                                                                                                                                                                                                                                                                                                                                                                                                                      2020-08-25T01:10:23.287568  image/svg+xml    Matplotlib v3.3.1, https://matplotlib.org/                                                                                                                                                                                                                                                                                                                                                                                                                                                                                                                                                                                                                                                                                                                                                                                                                                                                                                                                                                                                                                                                                                                                                                                                                                                                                                                                                                                                                                                         29   17   25   19   10   11   30   26   14   2   6   18   7   15   13   24   3   4   27              2020-08-25T01:10:23.421046  image/svg+xml    Matplotlib v3.3.1, https://matplotlib.org/                                                                                                                                                                                                                                                                                                                                                                                                                                                                                                                                                                                                                                                                                                                                                                                                                                                                                                                                                                                                                                                                                                                                                                                                                                                                                                                                                                                                                                                                                                                                      2020-08-25T01:10:23.572941  image/svg+xml    Matplotlib v3.3.1, https://matplotlib.org/                                                                                                                                                                                                                                                                                                                                                                                                                                                                                                                                                                                                                                                                                                                                                                                                                                                                                                                                                                                                                                                                                                                                                                                                                                                                                                                                                                                                                                                                                                                                                        2020-08-25T01:10:23.729246  image/svg+xml    Matplotlib v3.3.1, https://matplotlib.org/                                                                                                                                                                                                                                                                                                                                                                                                                                                                                                                                                                                                                                                                                                                                                                                                                                                                                                                                                                                                                                                                                                                                                                                                                                                                                                                                                                                                                                                                                                                                                      2020-08-25T01:10:24.069518  image/svg+xml    Matplotlib v3.3.1, https://matplotlib.org/                                                                                                                                                                                                                                                                                                                                                                                                                                                                                                                                                                                                                                                                                                                                                                                                                                                                                                                                                                                                                                                                                                                                                                                                                                                                                                                                                                                                                                                                                                                        2020-08-25T01:10:24.216237  image/svg+xml    Matplotlib v3.3.1, https://matplotlib.org/                                                                                                                                                                                                                                                                                                                                                                                                                                                                                                                                                                                                                                                                                                                                                                                                                                                                                                                                                                                                                                                                                                                                                                                                                                                                                                                                                                                                                                                                                                                                                                                    2020-08-25T01:10:24.371701  image/svg+xml    Matplotlib v3.3.1, https://matplotlib.org/                                                                                                                                                                                                                                                                                                                                                                                                                                                                                                                                                                                                                                                                                                                                                                                                                                                                                                                                                                                                                                                                                                                                                                                                                                                                                                                                                                                                                                                                                                                  2020-08-25T01:10:24.513557  image/svg+xml    Matplotlib v3.3.1, https://matplotlib.org/                                                                                                                                                                                                                                                                                                                                                                                                                                                                                                                                                                                                                                                                                                                                                                                                                                                                                                                                                                                                                                                                                                                                                                                                                                                                                                                                                                                                                                                                                                2020-08-25T01:10:24.655961  image/svg+xml    Matplotlib v3.3.1, https://matplotlib.org/                                                                                                                                                                                                                                                                                                                                                                                                                                                                                                                                                                                                                                                                                                                                                                                                                                                                                                                                                                                                                                                                                                                                                                                                                                                                                                                                                                                                                                                                                                                                                    2020-08-25T01:10:24.806529  image/svg+xml    Matplotlib v3.3.1, https://matplotlib.org/                                                                                                                                                                                                                                                                                                                                                                                                                                                                                                                                                                                                                                                                                                                                                                                                                                                                                                                                                                                                                                                                                                                                                                                                                                                                                                                                                                                                                                                                              2020-08-25T01:10:24.948302  image/svg+xml    Matplotlib v3.3.1, https://matplotlib.org/                                                                                                                                                                                                                                                                                                                                                                                                                                                                                                                                                                                                                                                                                                                                                                                                                                                                                                                                                                                                                                                                                                                                                                                                                                                                                                                                                                                                                                                                      2020-08-25T01:10:25.088049  image/svg+xml    Matplotlib v3.3.1, https://matplotlib.org/                                                                                                                                                                                                                                                                                                                                                                                                                                                                                                                                                                                                                                                                                                                                                                                                                                                                                                                                                                                                                                                                                                                                                                                                                                                                                                                                                                                                                                                                                                                      2020-08-25T01:10:25.236075  image/svg+xml    Matplotlib v3.3.1, https://matplotlib.org/                                                                                                                                                                                                                                                                                                                                                                                                                                                                                                                                                                                                                                                                                                                                                                                                                                                                                                                                                                                                                                                                                                                                                                                                                                                                                                                                                                                                                                                                                                                                                              2020-08-25T01:10:25.392782  image/svg+xml    Matplotlib v3.3.1, https://matplotlib.org/                                                                                                                                                                                                                                                                                                                                                                                                                                                                                                                                                                                                                                                                                                                                                                                                                                                                                                                                                                                                                                                                                                                                                                                                                                                                                                                                                                                                                                                                                                                                    2020-08-25T01:10:25.543050  image/svg+xml    Matplotlib v3.3.1, https://matplotlib.org/
[truncated: 218,345 more chars]
