# Supplementary material for: TPOT-NN: augmenting tree-based automated machine learning with neural network estimators
Source: Genet Program Evolvable Mach. Author manuscript; Available in PMC 2025 Aug 6. (PMC12327408; doi:10.1007/s10710-021-09401-z)
Supplement: Supplementary Material 1 [file NIHMS2035489-supplement-Supplementary_Material_1.zip › New folder/Hill_Valley_with_noise.html]

Hill\_Valley\_with\_noise 

Toggle navigationHill\_Valley\_with\_noise

- Overview
- Variables
- Interactions
- Correlations
- Missing values
- Sample

# Overview

- Overview
- Reproduction
- Warnings 19

Dataset statistics

|  |  |
| --- | --- |
| Number of variables | 20 |
| Number of observations | 1212 |
| Missing cells | 0 |
| Missing cells (%) | 0.0% |
| Duplicate rows | 0 |
| Duplicate rows (%) | 0.0% |
| Total size in memory | 189.5 KiB |
| Average record size in memory | 160.1 B |

Variable types

|  |  |
| --- | --- |
| NUM | 19 |
| BOOL | 1 |

Reproduction

|  |  |
| --- | --- |
| Analysis started | 2020-08-25 01:00:48.582839 |
| Analysis finished | 2020-08-25 01:01:42.960485 |
| Duration | 54.38 seconds |
| Version | pandas-profiling v2.8.0 |
| Command line | `pandas_profiling --config_file config.yaml [YOUR_FILE.csv]` |
| Download configuration | config.yaml |

Warnings

|  |  |
| --- | --- |
| `X54` is highly correlated with `X84` and 17 other fields | High correlation |
| `X84` is highly correlated with `X54` and 17 other fields | High correlation |
| `X71` is highly correlated with `X84` and 17 other fields | High correlation |
| `X46` is highly correlated with `X84` and 17 other fields | High correlation |
| `X45` is highly correlated with `X84` and 17 other fields | High correlation |
| `X40` is highly correlated with `X84` and 17 other fields | High correlation |
| `X23` is highly correlated with `X84` and 17 other fields | High correlation |
| `X81` is highly correlated with `X84` and 17 other fields | High correlation |
| `X11` is highly correlated with `X84` and 17 other fields | High correlation |
| `X1` is highly correlated with `X84` and 17 other fields | High correlation |
| `X19` is highly correlated with `X84` and 17 other fields | High correlation |
| `X31` is highly correlated with `X84` and 17 other fields | High correlation |
| `X74` is highly correlated with `X84` and 17 other fields | High correlation |
| `X34` is highly correlated with `X84` and 17 other fields | High correlation |
| `X91` is highly correlated with `X84` and 17 other fields | High correlation |
| `X5` is highly correlated with `X84` and 17 other fields | High correlation |
| `X77` is highly correlated with `X84` and 17 other fields | High correlation |
| `X78` is highly correlated with `X84` and 17 other fields | High correlation |
| `X13` is highly correlated with `X84` and 17 other fields | High correlation |

# Variables

X84  
Real number (ℝ≥0)

`HIGH CORRELATION`

|  |  |
| --- | --- |
| Distinct count | 1165 |
| Unique (%) | 96.1% |
| Missing | 0 |
| Missing (%) | 0.0% |
| Infinite | 0 |
| Infinite (%) | 0.0% |

|  |  |
| --- | --- |
| Mean | 8089.071014851485 |
| Minimum | 0.85 |
| Maximum | 108777.51 |
| Zeros | 0 |
| Zeros (%) | 0.0% |
| Memory size | 9.6 KiB |

2020-08-25T01:01:43.010605image/svg+xmlMatplotlib v3.3.1, https://matplotlib.org/

Toggle details

- Statistics
- Histogram(s)
- Common values
- Extreme values

Quantile statistics

|  |  |
| --- | --- |
| Minimum | 0.85 |
| 5-th percentile | 1.8455 |
| Q1 | 18.5075 |
| median | 302.05 |
| Q3 | 5502.335 |
| 95-th percentile | 51115.7935 |
| Maximum | 108777.51 |
| Range | 108776.66 |
| Interquartile range (IQR) | 5483.8275 |

Descriptive statistics

|  |  |
| --- | --- |
| Standard deviation | 17668.98386 |
| Coefficient of variation (CV) | 2.18430322 |
| Kurtosis | 8.989386569 |
| Mean | 8089.071015 |
| Median Absolute Deviation (MAD) | 300.02 |
| Skewness | 2.942563927 |
| Sum | 9803954.07 |
| Variance | 312192990.8 |

- Histogram

2020-08-25T01:01:43.108098image/svg+xmlMatplotlib v3.3.1, https://matplotlib.org/ 

**Histogram with fixed size bins** (bins=10)

| Value | Count | Frequency (%) |  |
| --- | --- | --- | --- |
| 1.17 | 4 | 0.3% |  |
| 1.5 | 4 | 0.3% |  |
| 7.98 | 4 | 0.3% |  |
| 1.41 | 3 | 0.2% |  |
| 3.16 | 2 | 0.2% |  |
| 13.6 | 2 | 0.2% |  |
| 2.82 | 2 | 0.2% |  |
| 2.34 | 2 | 0.2% |  |
| 1.34 | 2 | 0.2% |  |
| 2.48 | 2 | 0.2% |  |
| 1.72 | 2 | 0.2% |  |
| 1.66 | 2 | 0.2% |  |
| 4.8 | 2 | 0.2% |  |
| 52.14 | 2 | 0.2% |  |
| 5.64 | 2 | 0.2% |  |
| 1.15 | 2 | 0.2% |  |
| 2.21 | 2 | 0.2% |  |
| 1.14 | 2 | 0.2% |  |
| 1.99 | 2 | 0.2% |  |
| 2.64 | 2 | 0.2% |  |
| 4.3 | 2 | 0.2% |  |
| 7.13 | 2 | 0.2% |  |
| 1.7 | 2 | 0.2% |  |
| 6.93 | 2 | 0.2% |  |
| 9.61 | 2 | 0.2% |  |
| Other values (1140) | 1155 | 95.3% |  |

- Minimum 5 values
- Maximum 5 values

| Value | Count | Frequency (%) |  |
| --- | --- | --- | --- |
| 0.85 | 1 | 0.1% |  |
| 0.86 | 1 | 0.1% |  |
| 0.89 | 1 | 0.1% |  |
| 0.91 | 1 | 0.1% |  |
| 0.93 | 1 | 0.1% |  |
| 0.97 | 1 | 0.1% |  |
| 0.99 | 1 | 0.1% |  |
| 1.01 | 1 | 0.1% |  |
| 1.03 | 1 | 0.1% |  |
| 1.07 | 1 | 0.1% |  |

| Value | Count | Frequency (%) |  |
| --- | --- | --- | --- |
| 108777.51 | 1 | 0.1% |  |
| 105167.09 | 1 | 0.1% |  |
| 105018.87 | 1 | 0.1% |  |
| 104117.45 | 1 | 0.1% |  |
| 99547.47 | 1 | 0.1% |  |
| 95821.1 | 1 | 0.1% |  |
| 95567.01 | 1 | 0.1% |  |
| 87780.64 | 1 | 0.1% |  |
| 86565.53 | 1 | 0.1% |  |
| 86484.82 | 1 | 0.1% |  |

X54  
Real number (ℝ≥0)

`HIGH CORRELATION`

|  |  |
| --- | --- |
| Distinct count | 1179 |
| Unique (%) | 97.3% |
| Missing | 0 |
| Missing (%) | 0.0% |
| Infinite | 0 |
| Infinite (%) | 0.0% |

|  |  |
| --- | --- |
| Mean | 8260.25038778878 |
| Minimum | 0.88 |
| Maximum | 112189.42 |
| Zeros | 0 |
| Zeros (%) | 0.0% |
| Memory size | 9.6 KiB |

2020-08-25T01:01:43.217417image/svg+xmlMatplotlib v3.3.1, https://matplotlib.org/

Toggle details

- Statistics
- Histogram(s)
- Common values
- Extreme values

Quantile statistics

|  |  |
| --- | --- |
| Minimum | 0.88 |
| 5-th percentile | 1.8055 |
| Q1 | 18.6675 |
| median | 288.245 |
| Q3 | 5247.8825 |
| 95-th percentile | 53970.7295 |
| Maximum | 112189.42 |
| Range | 112188.54 |
| Interquartile range (IQR) | 5229.215 |

Descriptive statistics

|  |  |
| --- | --- |
| Standard deviation | 18231.47789 |
| Coefficient of variation (CV) | 2.207133808 |
| Kurtosis | 9.304983358 |
| Mean | 8260.250388 |
| Median Absolute Deviation (MAD) | 286.3 |
| Skewness | 2.984538321 |
| Sum | 10011423.47 |
| Variance | 332386786.1 |

- Histogram

2020-08-25T01:01:43.314793image/svg+xmlMatplotlib v3.3.1, https://matplotlib.org/ 

**Histogram with fixed size bins** (bins=10)

| Value | Count | Frequency (%) |  |
| --- | --- | --- | --- |
| 1.46 | 3 | 0.2% |  |
| 0.99 | 3 | 0.2% |  |
| 1.08 | 3 | 0.2% |  |
| 2.32 | 3 | 0.2% |  |
| 2.01 | 2 | 0.2% |  |
| 1.28 | 2 | 0.2% |  |
| 5.84 | 2 | 0.2% |  |
| 4.43 | 2 | 0.2% |  |
| 3.92 | 2 | 0.2% |  |
| 1.93 | 2 | 0.2% |  |
| 3.48 | 2 | 0.2% |  |
| 1.39 | 2 | 0.2% |  |
| 96.06 | 2 | 0.2% |  |
| 1.91 | 2 | 0.2% |  |
| 57.7 | 2 | 0.2% |  |
| 1.29 | 2 | 0.2% |  |
| 2.86 | 2 | 0.2% |  |
| 2.15 | 2 | 0.2% |  |
| 1.19 | 2 | 0.2% |  |
| 1.72 | 2 | 0.2% |  |
| 2.82 | 2 | 0.2% |  |
| 1.73 | 2 | 0.2% |  |
| 2.62 | 2 | 0.2% |  |
| 3.23 | 2 | 0.2% |  |
| 1.22 | 2 | 0.2% |  |
| Other values (1154) | 1158 | 95.5% |  |

- Minimum 5 values
- Maximum 5 values

| Value | Count | Frequency (%) |  |
| --- | --- | --- | --- |
| 0.88 | 1 | 0.1% |  |
| 0.9 | 1 | 0.1% |  |
| 0.91 | 1 | 0.1% |  |
| 0.92 | 1 | 0.1% |  |
| 0.93 | 1 | 0.1% |  |
| 0.94 | 1 | 0.1% |  |
| 0.96 | 1 | 0.1% |  |
| 0.97 | 1 | 0.1% |  |
| 0.98 | 1 | 0.1% |  |
| 0.99 | 3 | 0.2% |  |

| Value | Count | Frequency (%) |  |
| --- | --- | --- | --- |
| 112189.42 | 1 | 0.1% |  |
| 111316.42 | 1 | 0.1% |  |
| 108229.54 | 1 | 0.1% |  |
| 108219.65 | 1 | 0.1% |  |
| 106060.5 | 1 | 0.1% |  |
| 103743.64 | 1 | 0.1% |  |
| 100770.73 | 1 | 0.1% |  |
| 95438.61 | 1 | 0.1% |  |
| 94570.75 | 1 | 0.1% |  |
| 92459.03 | 1 | 0.1% |  |

X71  
Real number (ℝ≥0)

`HIGH CORRELATION`

|  |  |
| --- | --- |
| Distinct count | 1164 |
| Unique (%) | 96.0% |
| Missing | 0 |
| Missing (%) | 0.0% |
| Infinite | 0 |
| Infinite (%) | 0.0% |

|  |  |
| --- | --- |
| Mean | 8214.853580858085 |
| Minimum | 0.84 |
| Maximum | 123224.27 |
| Zeros | 0 |
| Zeros (%) | 0.0% |
| Memory size | 9.6 KiB |

2020-08-25T01:01:43.424129image/svg+xmlMatplotlib v3.3.1, https://matplotlib.org/

Toggle details

- Statistics
- Histogram(s)
- Common values
- Extreme values

Quantile statistics

|  |  |
| --- | --- |
| Minimum | 0.84 |
| 5-th percentile | 1.8075 |
| Q1 | 18.36 |
| median | 301.005 |
| Q3 | 5336.08 |
| 95-th percentile | 52643.6035 |
| Maximum | 123224.27 |
| Range | 123223.43 |
| Interquartile range (IQR) | 5317.72 |

Descriptive statistics

|  |  |
| --- | --- |
| Standard deviation | 18166.75209 |
| Coefficient of variation (CV) | 2.211451721 |
| Kurtosis | 9.886406369 |
| Mean | 8214.853581 |
| Median Absolute Deviation (MAD) | 298.975 |
| Skewness | 3.036859777 |
| Sum | 9956402.54 |
| Variance | 330030881.4 |

- Histogram

2020-08-25T01:01:43.529207image/svg+xmlMatplotlib v3.3.1, https://matplotlib.org/ 

**Histogram with fixed size bins** (bins=10)

| Value | Count | Frequency (%) |  |
| --- | --- | --- | --- |
| 1.65 | 5 | 0.4% |  |
| 2.12 | 4 | 0.3% |  |
| 1.96 | 3 | 0.2% |  |
| 2.03 | 3 | 0.2% |  |
| 1.07 | 3 | 0.2% |  |
| 3.88 | 3 | 0.2% |  |
| 1.32 | 3 | 0.2% |  |
| 1.38 | 3 | 0.2% |  |
| 1.78 | 3 | 0.2% |  |
| 1.28 | 3 | 0.2% |  |
| 2.97 | 2 | 0.2% |  |
| 4.68 | 2 | 0.2% |  |
| 5.44 | 2 | 0.2% |  |
| 2.23 | 2 | 0.2% |  |
| 2.77 | 2 | 0.2% |  |
| 3.17 | 2 | 0.2% |  |
| 1.18 | 2 | 0.2% |  |
| 11.87 | 2 | 0.2% |  |
| 2.64 | 2 | 0.2% |  |
| 1.67 | 2 | 0.2% |  |
| 6.77 | 2 | 0.2% |  |
| 1.54 | 2 | 0.2% |  |
| 1.97 | 2 | 0.2% |  |
| 63.76 | 2 | 0.2% |  |
| 2.27 | 2 | 0.2% |  |
| Other values (1139) | 1149 | 94.8% |  |

- Minimum 5 values
- Maximum 5 values

| Value | Count | Frequency (%) |  |
| --- | --- | --- | --- |
| 0.84 | 1 | 0.1% |  |
| 0.91 | 1 | 0.1% |  |
| 0.92 | 1 | 0.1% |  |
| 0.95 | 1 | 0.1% |  |
| 1 | 2 | 0.2% |  |
| 1.02 | 1 | 0.1% |  |
| 1.04 | 2 | 0.2% |  |
| 1.05 | 1 | 0.1% |  |
| 1.06 | 2 | 0.2% |  |
| 1.07 | 3 | 0.2% |  |

| Value | Count | Frequency (%) |  |
| --- | --- | --- | --- |
| 123224.27 | 1 | 0.1% |  |
| 111747.72 | 1 | 0.1% |  |
| 111377.43 | 1 | 0.1% |  |
| 109714.38 | 1 | 0.1% |  |
| 107932.3 | 1 | 0.1% |  |
| 104136.75 | 1 | 0.1% |  |
| 99959.93 | 1 | 0.1% |  |
| 98931.23 | 1 | 0.1% |  |
| 97362.85 | 1 | 0.1% |  |
| 87340.35 | 1 | 0.1% |  |

X46  
Real number (ℝ≥0)

`HIGH CORRELATION`

|  |  |
| --- | --- |
| Distinct count | 1157 |
| Unique (%) | 95.5% |
| Missing | 0 |
| Missing (%) | 0.0% |
| Infinite | 0 |
| Infinite (%) | 0.0% |

|  |  |
| --- | --- |
| Mean | 8125.8930610561065 |
| Minimum | 0.9 |
| Maximum | 115898.37 |
| Zeros | 0 |
| Zeros (%) | 0.0% |
| Memory size | 9.6 KiB |

2020-08-25T01:01:43.639656image/svg+xmlMatplotlib v3.3.1, https://matplotlib.org/

Toggle details

- Statistics
- Histogram(s)
- Common values
- Extreme values

Quantile statistics

|  |  |
| --- | --- |
| Minimum | 0.9 |
| 5-th percentile | 1.83 |
| Q1 | 18.665 |
| median | 290.555 |
| Q3 | 5200.7025 |
| 95-th percentile | 51123.595 |
| Maximum | 115898.37 |
| Range | 115897.47 |
| Interquartile range (IQR) | 5182.0375 |

Descriptive statistics

|  |  |
| --- | --- |
| Standard deviation | 17866.39842 |
| Coefficient of variation (CV) | 2.198699674 |
| Kurtosis | 9.52901463 |
| Mean | 8125.893061 |
| Median Absolute Deviation (MAD) | 288.645 |
| Skewness | 2.99868572 |
| Sum | 9848582.39 |
| Variance | 319208192.5 |

- Histogram

2020-08-25T01:01:43.746435image/svg+xmlMatplotlib v3.3.1, https://matplotlib.org/ 

**Histogram with fixed size bins** (bins=10)

| Value | Count | Frequency (%) |  |
| --- | --- | --- | --- |
| 2.26 | 4 | 0.3% |  |
| 10.83 | 3 | 0.2% |  |
| 2.09 | 3 | 0.2% |  |
| 1.83 | 3 | 0.2% |  |
| 3.44 | 3 | 0.2% |  |
| 1.75 | 3 | 0.2% |  |
| 1.21 | 2 | 0.2% |  |
| 1.14 | 2 | 0.2% |  |
| 4.42 | 2 | 0.2% |  |
| 4.46 | 2 | 0.2% |  |
| 1.19 | 2 | 0.2% |  |
| 0.98 | 2 | 0.2% |  |
| 2.7 | 2 | 0.2% |  |
| 2.01 | 2 | 0.2% |  |
| 2.29 | 2 | 0.2% |  |
| 2.82 | 2 | 0.2% |  |
| 1.22 | 2 | 0.2% |  |
| 1.16 | 2 | 0.2% |  |
| 1.79 | 2 | 0.2% |  |
| 2.02 | 2 | 0.2% |  |
| 0.95 | 2 | 0.2% |  |
| 3.62 | 2 | 0.2% |  |
| 1.84 | 2 | 0.2% |  |
| 1.35 | 2 | 0.2% |  |
| 3.26 | 2 | 0.2% |  |
| Other values (1132) | 1155 | 95.3% |  |

- Minimum 5 values
- Maximum 5 values

| Value | Count | Frequency (%) |  |
| --- | --- | --- | --- |
| 0.9 | 1 | 0.1% |  |
| 0.95 | 2 | 0.2% |  |
| 0.96 | 2 | 0.2% |  |
| 0.98 | 2 | 0.2% |  |
| 1 | 1 | 0.1% |  |
| 1.03 | 1 | 0.1% |  |
| 1.04 | 1 | 0.1% |  |
| 1.05 | 1 | 0.1% |  |
| 1.09 | 1 | 0.1% |  |
| 1.11 | 1 | 0.1% |  |

| Value | Count | Frequency (%) |  |
| --- | --- | --- | --- |
| 115898.37 | 1 | 0.1% |  |
| 110472.47 | 1 | 0.1% |  |
| 109770.11 | 1 | 0.1% |  |
| 107931.2 | 1 | 0.1% |  |
| 100448.59 | 1 | 0.1% |  |
| 99519.28 | 1 | 0.1% |  |
| 99112.92 | 1 | 0.1% |  |
| 96978.76 | 1 | 0.1% |  |
| 89928.83 | 1 | 0.1% |  |
| 89512.89 | 1 | 0.1% |  |

X45  
Real number (ℝ≥0)

`HIGH CORRELATION`

|  |  |
| --- | --- |
| Distinct count | 1167 |
| Unique (%) | 96.3% |
| Missing | 0 |
| Missing (%) | 0.0% |
| Infinite | 0 |
| Infinite (%) | 0.0% |

|  |  |
| --- | --- |
| Mean | 8144.133580858086 |
| Minimum | 0.88 |
| Maximum | 114511.49 |
| Zeros | 0 |
| Zeros (%) | 0.0% |
| Memory size | 9.6 KiB |

2020-08-25T01:01:43.863599image/svg+xmlMatplotlib v3.3.1, https://matplotlib.org/

Toggle details

- Statistics
- Histogram(s)
- Common values
- Extreme values

Quantile statistics

|  |  |
| --- | --- |
| Minimum | 0.88 |
| 5-th percentile | 1.852 |
| Q1 | 18.7075 |
| median | 296.505 |
| Q3 | 5222.69 |
| 95-th percentile | 51298.6265 |
| Maximum | 114511.49 |
| Range | 114510.61 |
| Interquartile range (IQR) | 5203.9825 |

Descriptive statistics

|  |  |
| --- | --- |
| Standard deviation | 17910.58202 |
| Coefficient of variation (CV) | 2.199200424 |
| Kurtosis | 9.527984866 |
| Mean | 8144.133581 |
| Median Absolute Deviation (MAD) | 294.545 |
| Skewness | 3.001481273 |
| Sum | 9870689.9 |
| Variance | 320788948.3 |

- Histogram

2020-08-25T01:01:43.963038image/svg+xmlMatplotlib v3.3.1, https://matplotlib.org/ 

**Histogram with fixed size bins** (bins=10)

| Value | Count | Frequency (%) |  |
| --- | --- | --- | --- |
| 1.95 | 4 | 0.3% |  |
| 1.07 | 3 | 0.2% |  |
| 2.17 | 3 | 0.2% |  |
| 9 | 3 | 0.2% |  |
| 3.84 | 3 | 0.2% |  |
| 1.41 | 2 | 0.2% |  |
| 1.88 | 2 | 0.2% |  |
| 1.12 | 2 | 0.2% |  |
| 4.06 | 2 | 0.2% |  |
| 4.02 | 2 | 0.2% |  |
| 1.7 | 2 | 0.2% |  |
| 1.19 | 2 | 0.2% |  |
| 373.97 | 2 | 0.2% |  |
| 1.02 | 2 | 0.2% |  |
| 50.17 | 2 | 0.2% |  |
| 1.83 | 2 | 0.2% |  |
| 6.31 | 2 | 0.2% |  |
| 2.77 | 2 | 0.2% |  |
| 1.96 | 2 | 0.2% |  |
| 7.56 | 2 | 0.2% |  |
| 1.46 | 2 | 0.2% |  |
| 2.09 | 2 | 0.2% |  |
| 3.04 | 2 | 0.2% |  |
| 7.61 | 2 | 0.2% |  |
| 6.27 | 2 | 0.2% |  |
| Other values (1142) | 1156 | 95.4% |  |

- Minimum 5 values
- Maximum 5 values

| Value | Count | Frequency (%) |  |
| --- | --- | --- | --- |
| 0.88 | 2 | 0.2% |  |
| 0.9 | 1 | 0.1% |  |
| 0.93 | 1 | 0.1% |  |
| 0.94 | 1 | 0.1% |  |
| 0.98 | 1 | 0.1% |  |
| 1.02 | 2 | 0.2% |  |
| 1.03 | 2 | 0.2% |  |
| 1.04 | 1 | 0.1% |  |
| 1.06 | 1 | 0.1% |  |
| 1.07 | 3 | 0.2% |  |

| Value | Count | Frequency (%) |  |
| --- | --- | --- | --- |
| 114511.49 | 1 | 0.1% |  |
| 113103.39 | 1 | 0.1% |  |
| 108617.66 | 1 | 0.1% |  |
| 106110.44 | 1 | 0.1% |  |
| 105156.71 | 1 | 0.1% |  |
| 101013.39 | 1 | 0.1% |  |
| 98183.97 | 1 | 0.1% |  |
| 95212.21 | 1 | 0.1% |  |
| 90471.02 | 1 | 0.1% |  |
| 88305.75 | 1 | 0.1% |  |

X40  
Real number (ℝ≥0)

`HIGH CORRELATION`

|  |  |
| --- | --- |
| Distinct count | 1168 |
| Unique (%) | 96.4% |
| Missing | 0 |
| Missing (%) | 0.0% |
| Infinite | 0 |
| Infinite (%) | 0.0% |

|  |  |
| --- | --- |
| Mean | 8121.182780528054 |
| Minimum | 0.66 |
| Maximum | 114450.98 |
| Zeros | 0 |
| Zeros (%) | 0.0% |
| Memory size | 9.6 KiB |

2020-08-25T01:01:44.073026image/svg+xmlMatplotlib v3.3.1, https://matplotlib.org/

Toggle details

- Statistics
- Histogram(s)
- Common values
- Extreme values

Quantile statistics

|  |  |
| --- | --- |
| Minimum | 0.66 |
| 5-th percentile | 1.852 |
| Q1 | 19.0825 |
| median | 286.63 |
| Q3 | 5300.7075 |
| 95-th percentile | 50960.3335 |
| Maximum | 114450.98 |
| Range | 114450.32 |
| Interquartile range (IQR) | 5281.625 |

Descriptive statistics

|  |  |
| --- | --- |
| Standard deviation | 17823.33861 |
| Coefficient of variation (CV) | 2.194672757 |
| Kurtosis | 9.215259777 |
| Mean | 8121.182781 |
| Median Absolute Deviation (MAD) | 284.735 |
| Skewness | 2.972235441 |
| Sum | 9842873.53 |
| Variance | 317671399.1 |

- Histogram

2020-08-25T01:01:44.171527image/svg+xmlMatplotlib v3.3.1, https://matplotlib.org/ 

**Histogram with fixed size bins** (bins=10)

| Value | Count | Frequency (%) |  |
| --- | --- | --- | --- |
| 1 | 4 | 0.3% |  |
| 1.16 | 4 | 0.3% |  |
| 5.06 | 3 | 0.2% |  |
| 3.82 | 3 | 0.2% |  |
| 4.95 | 2 | 0.2% |  |
| 2.33 | 2 | 0.2% |  |
| 2.27 | 2 | 0.2% |  |
| 1.95 | 2 | 0.2% |  |
| 5.72 | 2 | 0.2% |  |
| 2.2 | 2 | 0.2% |  |
| 3.06 | 2 | 0.2% |  |
| 1.17 | 2 | 0.2% |  |
| 1.44 | 2 | 0.2% |  |
| 1.33 | 2 | 0.2% |  |
| 2.44 | 2 | 0.2% |  |
| 2.56 | 2 | 0.2% |  |
| 28.87 | 2 | 0.2% |  |
| 1.97 | 2 | 0.2% |  |
| 6.43 | 2 | 0.2% |  |
| 9.89 | 2 | 0.2% |  |
| 11.27 | 2 | 0.2% |  |
| 1.57 | 2 | 0.2% |  |
| 3.49 | 2 | 0.2% |  |
| 2.03 | 2 | 0.2% |  |
| 3.89 | 2 | 0.2% |  |
| Other values (1143) | 1156 | 95.4% |  |

- Minimum 5 values
- Maximum 5 values

| Value | Count | Frequency (%) |  |
| --- | --- | --- | --- |
| 0.66 | 1 | 0.1% |  |
| 0.87 | 1 | 0.1% |  |
| 0.92 | 1 | 0.1% |  |
| 0.95 | 1 | 0.1% |  |
| 0.97 | 2 | 0.2% |  |
| 1 | 4 | 0.3% |  |
| 1.02 | 1 | 0.1% |  |
| 1.03 | 1 | 0.1% |  |
| 1.06 | 1 | 0.1% |  |
| 1.08 | 1 | 0.1% |  |

| Value | Count | Frequency (%) |  |
| --- | --- | --- | --- |
| 114450.98 | 1 | 0.1% |  |
| 110620.12 | 1 | 0.1% |  |
| 109677.99 | 1 | 0.1% |  |
| 101093.35 | 1 | 0.1% |  |
| 99532.36 | 1 | 0.1% |  |
| 98890.36 | 1 | 0.1% |  |
| 96122.67 | 1 | 0.1% |  |
| 87892.66 | 1 | 0.1% |  |
| 86995.92 | 1 | 0.1% |  |
| 84197.13 | 1 | 0.1% |  |

X23  
Real number (ℝ≥0)

`HIGH CORRELATION`

|  |  |
| --- | --- |
| Distinct count | 1172 |
| Unique (%) | 96.7% |
| Missing | 0 |
| Missing (%) | 0.0% |
| Infinite | 0 |
| Infinite (%) | 0.0% |

|  |  |
| --- | --- |
| Mean | 8189.066526402639 |
| Minimum | 0.89 |
| Maximum | 136823.46 |
| Zeros | 0 |
| Zeros (%) | 0.0% |
| Memory size | 9.6 KiB |

2020-08-25T01:01:44.278559image/svg+xmlMatplotlib v3.3.1, https://matplotlib.org/

Toggle details

- Statistics
- Histogram(s)
- Common values
- Extreme values

Quantile statistics

|  |  |
| --- | --- |
| Minimum | 0.89 |
| 5-th percentile | 1.8655 |
| Q1 | 19.18 |
| median | 294.515 |
| Q3 | 5278.845 |
| 95-th percentile | 52715.676 |
| Maximum | 136823.46 |
| Range | 136822.57 |
| Interquartile range (IQR) | 5259.665 |

Descriptive statistics

|  |  |
| --- | --- |
| Standard deviation | 18092.02661 |
| Coefficient of variation (CV) | 2.209290467 |
| Kurtosis | 10.11517946 |
| Mean | 8189.066526 |
| Median Absolute Deviation (MAD) | 292.54 |
| Skewness | 3.044420676 |
| Sum | 9925148.63 |
| Variance | 327321426.8 |

- Histogram

2020-08-25T01:01:44.383496image/svg+xmlMatplotlib v3.3.1, https://matplotlib.org/ 

**Histogram with fixed size bins** (bins=10)

| Value | Count | Frequency (%) |  |
| --- | --- | --- | --- |
| 0.97 | 4 | 0.3% |  |
| 1.93 | 3 | 0.2% |  |
| 1.46 | 3 | 0.2% |  |
| 3.7 | 3 | 0.2% |  |
| 1.26 | 3 | 0.2% |  |
| 2 | 2 | 0.2% |  |
| 3.02 | 2 | 0.2% |  |
| 1.8 | 2 | 0.2% |  |
| 7.84 | 2 | 0.2% |  |
| 1.17 | 2 | 0.2% |  |
| 1.91 | 2 | 0.2% |  |
| 2.13 | 2 | 0.2% |  |
| 5.53 | 2 | 0.2% |  |
| 1.51 | 2 | 0.2% |  |
| 1.98 | 2 | 0.2% |  |
| 2.21 | 2 | 0.2% |  |
| 8.88 | 2 | 0.2% |  |
| 246.53 | 2 | 0.2% |  |
| 1.32 | 2 | 0.2% |  |
| 2.3 | 2 | 0.2% |  |
| 2.44 | 2 | 0.2% |  |
| 4.13 | 2 | 0.2% |  |
| 1.83 | 2 | 0.2% |  |
| 2.17 | 2 | 0.2% |  |
| 6.2 | 2 | 0.2% |  |
| Other values (1147) | 1156 | 95.4% |  |

- Minimum 5 values
- Maximum 5 values

| Value | Count | Frequency (%) |  |
| --- | --- | --- | --- |
| 0.89 | 1 | 0.1% |  |
| 0.91 | 1 | 0.1% |  |
| 0.96 | 1 | 0.1% |  |
| 0.97 | 4 | 0.3% |  |
| 0.99 | 1 | 0.1% |  |
| 1.02 | 1 | 0.1% |  |
| 1.03 | 1 | 0.1% |  |
| 1.04 | 1 | 0.1% |  |
| 1.1 | 1 | 0.1% |  |
| 1.11 | 1 | 0.1% |  |

| Value | Count | Frequency (%) |  |
| --- | --- | --- | --- |
| 136823.46 | 1 | 0.1% |  |
| 114039.33 | 1 | 0.1% |  |
| 111633.47 | 1 | 0.1% |  |
| 102636.79 | 1 | 0.1% |  |
| 101430.89 | 1 | 0.1% |  |
| 100252.03 | 1 | 0.1% |  |
| 99611.42 | 1 | 0.1% |  |
| 99078.75 | 1 | 0.1% |  |
| 90355.46 | 1 | 0.1% |  |
| 83533.85 | 1 | 0.1% |  |

X81  
Real number (ℝ≥0)

`HIGH CORRELATION`

|  |  |
| --- | --- |
| Distinct count | 1178 |
| Unique (%) | 97.2% |
| Missing | 0 |
| Missing (%) | 0.0% |
| Infinite | 0 |
| Infinite (%) | 0.0% |

|  |  |
| --- | --- |
| Mean | 8189.425470297029 |
| Minimum | 0.8 |
| Maximum | 113272.64 |
| Zeros | 0 |
| Zeros (%) | 0.0% |
| Memory size | 9.6 KiB |

2020-08-25T01:01:44.498260image/svg+xmlMatplotlib v3.3.1, https://matplotlib.org/

Toggle details

- Statistics
- Histogram(s)
- Common values
- Extreme values

Quantile statistics

|  |  |
| --- | --- |
| Minimum | 0.8 |
| 5-th percentile | 1.85 |
| Q1 | 18.4375 |
| median | 310.08 |
| Q3 | 5321.775 |
| 95-th percentile | 52381.2005 |
| Maximum | 113272.64 |
| Range | 113271.84 |
| Interquartile range (IQR) | 5303.3375 |

Descriptive statistics

|  |  |
| --- | --- |
| Standard deviation | 18044.41768 |
| Coefficient of variation (CV) | 2.203380169 |
| Kurtosis | 9.190726691 |
| Mean | 8189.42547 |
| Median Absolute Deviation (MAD) | 307.97 |
| Skewness | 2.976933251 |
| Sum | 9925583.67 |
| Variance | 325601009.4 |

- Histogram

2020-08-25T01:01:44.592843image/svg+xmlMatplotlib v3.3.1, https://matplotlib.org/ 

**Histogram with fixed size bins** (bins=10)

| Value | Count | Frequency (%) |  |
| --- | --- | --- | --- |
| 3.58 | 3 | 0.2% |  |
| 3.86 | 3 | 0.2% |  |
| 1.27 | 3 | 0.2% |  |
| 1.52 | 3 | 0.2% |  |
| 1.17 | 3 | 0.2% |  |
| 2.27 | 2 | 0.2% |  |
| 4.72 | 2 | 0.2% |  |
| 1.13 | 2 | 0.2% |  |
| 2.15 | 2 | 0.2% |  |
| 1.38 | 2 | 0.2% |  |
| 1.43 | 2 | 0.2% |  |
| 22.41 | 2 | 0.2% |  |
| 1.98 | 2 | 0.2% |  |
| 2.51 | 2 | 0.2% |  |
| 4.35 | 2 | 0.2% |  |
| 1.1 | 2 | 0.2% |  |
| 2.33 | 2 | 0.2% |  |
| 1.85 | 2 | 0.2% |  |
| 5.22 | 2 | 0.2% |  |
| 11.05 | 2 | 0.2% |  |
| 1.67 | 2 | 0.2% |  |
| 4.42 | 2 | 0.2% |  |
| 1.25 | 2 | 0.2% |  |
| 1.75 | 2 | 0.2% |  |
| 12.56 | 2 | 0.2% |  |
| Other values (1153) | 1157 | 95.5% |  |

- Minimum 5 values
- Maximum 5 values

| Value | Count | Frequency (%) |  |
| --- | --- | --- | --- |
| 0.8 | 1 | 0.1% |  |
| 0.92 | 1 | 0.1% |  |
| 0.93 | 1 | 0.1% |  |
| 0.94 | 1 | 0.1% |  |
| 0.96 | 2 | 0.2% |  |
| 1 | 1 | 0.1% |  |
| 1.01 | 1 | 0.1% |  |
| 1.02 | 1 | 0.1% |  |
| 1.04 | 1 | 0.1% |  |
| 1.1 | 2 | 0.2% |  |

| Value | Count | Frequency (%) |  |
| --- | --- | --- | --- |
| 113272.64 | 1 | 0.1% |  |
| 105386.69 | 1 | 0.1% |  |
| 100942.33 | 1 | 0.1% |  |
| 99156.05 | 1 | 0.1% |  |
| 98036.88 | 1 | 0.1% |  |
| 96380.07 | 1 | 0.1% |  |
| 95926.08 | 1 | 0.1% |  |
| 95902.21 | 1 | 0.1% |  |
| 94969.04 | 1 | 0.1% |  |
| 94679.54 | 1 | 0.1% |  |

X11  
Real number (ℝ≥0)

`HIGH CORRELATION`

|  |  |
| --- | --- |
| Distinct count | 1173 |
| Unique (%) | 96.8% |
| Missing | 0 |
| Missing (%) | 0.0% |
| Infinite | 0 |
| Infinite (%) | 0.0% |

|  |  |
| --- | --- |
| Mean | 8194.712037953796 |
| Minimum | 0.8 |
| Maximum | 120882.32 |
| Zeros | 0 |
| Zeros (%) | 0.0% |
| Memory size | 9.6 KiB |

2020-08-25T01:01:44.698262image/svg+xmlMatplotlib v3.3.1, https://matplotlib.org/

Toggle details

- Statistics
- Histogram(s)
- Common values
- Extreme values

Quantile statistics

|  |  |
| --- | --- |
| Minimum | 0.8 |
| 5-th percentile | 1.8455 |
| Q1 | 19.53 |
| median | 297.465 |
| Q3 | 5476.625 |
| 95-th percentile | 52112.0865 |
| Maximum | 120882.32 |
| Range | 120881.52 |
| Interquartile range (IQR) | 5457.095 |

Descriptive statistics

|  |  |
| --- | --- |
| Standard deviation | 18117.19427 |
| Coefficient of variation (CV) | 2.210839647 |
| Kurtosis | 9.980153779 |
| Mean | 8194.712038 |
| Median Absolute Deviation (MAD) | 295.5 |
| Skewness | 3.048245949 |
| Sum | 9931990.99 |
| Variance | 328232728.3 |

- Histogram

2020-08-25T01:01:44.797844image/svg+xmlMatplotlib v3.3.1, https://matplotlib.org/ 

**Histogram with fixed size bins** (bins=10)

| Value | Count | Frequency (%) |  |
| --- | --- | --- | --- |
| 2.29 | 3 | 0.2% |  |
| 1.73 | 3 | 0.2% |  |
| 1.33 | 3 | 0.2% |  |
| 9.38 | 2 | 0.2% |  |
| 31.27 | 2 | 0.2% |  |
| 3.43 | 2 | 0.2% |  |
| 3.07 | 2 | 0.2% |  |
| 2.21 | 2 | 0.2% |  |
| 2.28 | 2 | 0.2% |  |
| 2.07 | 2 | 0.2% |  |
| 2.26 | 2 | 0.2% |  |
| 5.54 | 2 | 0.2% |  |
| 1.98 | 2 | 0.2% |  |
| 16.06 | 2 | 0.2% |  |
| 61.85 | 2 | 0.2% |  |
| 1.07 | 2 | 0.2% |  |
| 7.54 | 2 | 0.2% |  |
| 4.67 | 2 | 0.2% |  |
| 1.05 | 2 | 0.2% |  |
| 1.86 | 2 | 0.2% |  |
| 12.95 | 2 | 0.2% |  |
| 1.52 | 2 | 0.2% |  |
| 1.58 | 2 | 0.2% |  |
| 11.01 | 2 | 0.2% |  |
| 5.27 | 2 | 0.2% |  |
| Other values (1148) | 1159 | 95.6% |  |

- Minimum 5 values
- Maximum 5 values

| Value | Count | Frequency (%) |  |
| --- | --- | --- | --- |
| 0.8 | 1 | 0.1% |  |
| 0.91 | 1 | 0.1% |  |
| 0.92 | 1 | 0.1% |  |
| 0.96 | 2 | 0.2% |  |
| 0.97 | 1 | 0.1% |  |
| 0.98 | 1 | 0.1% |  |
| 1.04 | 1 | 0.1% |  |
| 1.05 | 2 | 0.2% |  |
| 1.06 | 1 | 0.1% |  |
| 1.07 | 2 | 0.2% |  |

| Value | Count | Frequency (%) |  |
| --- | --- | --- | --- |
| 120882.32 | 1 | 0.1% |  |
| 115155.06 | 1 | 0.1% |  |
| 111119.75 | 1 | 0.1% |  |
| 110947.2 | 1 | 0.1% |  |
| 108600.92 | 1 | 0.1% |  |
| 105179.27 | 1 | 0.1% |  |
| 100704.71 | 1 | 0.1% |  |
| 92565.49 | 1 | 0.1% |  |
| 91167.77 | 1 | 0.1% |  |
| 89774.99 | 1 | 0.1% |  |

X1  
Real number (ℝ≥0)

`HIGH CORRELATION`

|  |  |
| --- | --- |
| Distinct count | 1170 |
| Unique (%) | 96.5% |
| Missing | 0 |
| Missing (%) | 0.0% |
| Infinite | 0 |
| Infinite (%) | 0.0% |

|  |  |
| --- | --- |
| Mean | 8169.091881188118 |
| Minimum | 0.92 |
| Maximum | 117807.87 |
| Zeros | 0 |
| Zeros (%) | 0.0% |
| Memory size | 9.6 KiB |

2020-08-25T01:01:44.909409image/svg+xmlMatplotlib v3.3.1, https://matplotlib.org/

Toggle details

- Statistics
- Histogram(s)
- Common values
- Extreme values

Quantile statistics

|  |  |
| --- | --- |
| Minimum | 0.92 |
| 5-th percentile | 1.881 |
| Q1 | 19.6025 |
| median | 301.425 |
| Q3 | 5358.795 |
| 95-th percentile | 53566.5665 |
| Maximum | 117807.87 |
| Range | 117806.95 |
| Interquartile range (IQR) | 5339.1925 |

Descriptive statistics

|  |  |
| --- | --- |
| Standard deviation | 17974.95046 |
| Coefficient of variation (CV) | 2.200360912 |
| Kurtosis | 9.255560944 |
| Mean | 8169.091881 |
| Median Absolute Deviation (MAD) | 299.405 |
| Skewness | 2.976090722 |
| Sum | 9900939.36 |
| Variance | 323098844.1 |

- Histogram

2020-08-25T01:01:45.008420image/svg+xmlMatplotlib v3.3.1, https://matplotlib.org/ 

**Histogram with fixed size bins** (bins=10)

| Value | Count | Frequency (%) |  |
| --- | --- | --- | --- |
| 1.25 | 3 | 0.2% |  |
| 1.27 | 3 | 0.2% |  |
| 2.17 | 3 | 0.2% |  |
| 3.78 | 3 | 0.2% |  |
| 4.13 | 3 | 0.2% |  |
| 5.34 | 2 | 0.2% |  |
| 2.58 | 2 | 0.2% |  |
| 10.43 | 2 | 0.2% |  |
| 1.05 | 2 | 0.2% |  |
| 3.1 | 2 | 0.2% |  |
| 1.86 | 2 | 0.2% |  |
| 7.45 | 2 | 0.2% |  |
| 23.09 | 2 | 0.2% |  |
| 1.96 | 2 | 0.2% |  |
| 0.94 | 2 | 0.2% |  |
| 9.83 | 2 | 0.2% |  |
| 1.42 | 2 | 0.2% |  |
| 1.46 | 2 | 0.2% |  |
| 1.87 | 2 | 0.2% |  |
| 1.15 | 2 | 0.2% |  |
| 7.92 | 2 | 0.2% |  |
| 1.83 | 2 | 0.2% |  |
| 5.7 | 2 | 0.2% |  |
| 2.23 | 2 | 0.2% |  |
| 11.11 | 2 | 0.2% |  |
| Other values (1145) | 1157 | 95.5% |  |

- Minimum 5 values
- Maximum 5 values

| Value | Count | Frequency (%) |  |
| --- | --- | --- | --- |
| 0.92 | 1 | 0.1% |  |
| 0.93 | 1 | 0.1% |  |
| 0.94 | 2 | 0.2% |  |
| 0.95 | 1 | 0.1% |  |
| 0.98 | 1 | 0.1% |  |
| 1 | 2 | 0.2% |  |
| 1.03 | 1 | 0.1% |  |
| 1.04 | 1 | 0.1% |  |
| 1.05 | 2 | 0.2% |  |
| 1.08 | 1 | 0.1% |  |

| Value | Count | Frequency (%) |  |
| --- | --- | --- | --- |
| 117807.87 | 1 | 0.1% |  |
| 109366.06 | 1 | 0.1% |  |
| 105749.69 | 1 | 0.1% |  |
| 103032.84 | 1 | 0.1% |  |
| 102748.8 | 1 | 0.1% |  |
| 102154.35 | 1 | 0.1% |  |
| 94471.26 | 1 | 0.1% |  |
| 93351.7 | 1 | 0.1% |  |
| 93215.39 | 1 | 0.1% |  |
| 90526.5 | 1 | 0.1% |  |

X19  
Real number (ℝ≥0)

`HIGH CORRELATION`

|  |  |
| --- | --- |
| Distinct count | 1164 |
| Unique (%) | 96.0% |
| Missing | 0 |
| Missing (%) | 0.0% |
| Infinite | 0 |
| Infinite (%) | 0.0% |

|  |  |
| --- | --- |
| Mean | 8114.120420792078 |
| Minimum | 0.92 |
| Maximum | 114216.32 |
| Zeros | 0 |
| Zeros (%) | 0.0% |
| Memory size | 9.6 KiB |

2020-08-25T01:01:45.124365image/svg+xmlMatplotlib v3.3.1, https://matplotlib.org/

Toggle details

- Statistics
- Histogram(s)
- Common values
- Extreme values

Quantile statistics

|  |  |
| --- | --- |
| Minimum | 0.92 |
| 5-th percentile | 1.812 |
| Q1 | 18.7225 |
| median | 292.35 |
| Q3 | 5215.1925 |
| 95-th percentile | 53388.67 |
| Maximum | 114216.32 |
| Range | 114215.4 |
| Interquartile range (IQR) | 5196.47 |

Descriptive statistics

|  |  |
| --- | --- |
| Standard deviation | 17853.24499 |
| Coefficient of variation (CV) | 2.200268676 |
| Kurtosis | 9.531589629 |
| Mean | 8114.120421 |
| Median Absolute Deviation (MAD) | 290.44 |
| Skewness | 2.998353822 |
| Sum | 9834313.95 |
| Variance | 318738356.8 |

- Histogram

2020-08-25T01:01:45.226633image/svg+xmlMatplotlib v3.3.1, https://matplotlib.org/ 

**Histogram with fixed size bins** (bins=10)

| Value | Count | Frequency (%) |  |
| --- | --- | --- | --- |
| 2.08 | 4 | 0.3% |  |
| 1.42 | 3 | 0.2% |  |
| 2.98 | 3 | 0.2% |  |
| 3.14 | 3 | 0.2% |  |
| 1.19 | 3 | 0.2% |  |
| 0.95 | 3 | 0.2% |  |
| 1.69 | 3 | 0.2% |  |
| 1.76 | 2 | 0.2% |  |
| 1.09 | 2 | 0.2% |  |
| 9.91 | 2 | 0.2% |  |
| 3.69 | 2 | 0.2% |  |
| 14.42 | 2 | 0.2% |  |
| 1.59 | 2 | 0.2% |  |
| 2.27 | 2 | 0.2% |  |
| 2.4 | 2 | 0.2% |  |
| 5.37 | 2 | 0.2% |  |
| 6.9 | 2 | 0.2% |  |
| 2.09 | 2 | 0.2% |  |
| 1.6 | 2 | 0.2% |  |
| 5.69 | 2 | 0.2% |  |
| 1.43 | 2 | 0.2% |  |
| 2.04 | 2 | 0.2% |  |
| 1.89 | 2 | 0.2% |  |
| 7.6 | 2 | 0.2% |  |
| 1.04 | 2 | 0.2% |  |
| Other values (1139) | 1154 | 95.2% |  |

- Minimum 5 values
- Maximum 5 values

| Value | Count | Frequency (%) |  |
| --- | --- | --- | --- |
| 0.92 | 2 | 0.2% |  |
| 0.95 | 3 | 0.2% |  |
| 0.97 | 1 | 0.1% |  |
| 1.02 | 1 | 0.1% |  |
| 1.04 | 2 | 0.2% |  |
| 1.06 | 1 | 0.1% |  |
| 1.07 | 1 | 0.1% |  |
| 1.08 | 1 | 0.1% |  |
| 1.09 | 2 | 0.2% |  |
| 1.11 | 2 | 0.2% |  |

| Value | Count | Frequency (%) |  |
| --- | --- | --- | --- |
| 114216.32 | 1 | 0.1% |  |
| 113687.66 | 1 | 0.1% |  |
| 110852.31 | 1 | 0.1% |  |
| 108154.71 | 1 | 0.1% |  |
| 107024.48 | 1 | 0.1% |  |
| 98515.31 | 1 | 0.1% |  |
| 96790.53 | 1 | 0.1% |  |
| 95402.47 | 1 | 0.1% |  |
| 85594.86 | 1 | 0.1% |  |
| 83411.84 | 1 | 0.1% |  |

X31  
Real number (ℝ≥0)

`HIGH CORRELATION`

|  |  |
| --- | --- |
| Distinct count | 1174 |
| Unique (%) | 96.9% |
| Missing | 0 |
| Missing (%) | 0.0% |
| Infinite | 0 |
| Infinite (%) | 0.0% |

|  |  |
| --- | --- |
| Mean | 8132.08099009901 |
| Minimum | 0.71 |
| Maximum | 110662.75 |
| Zeros | 0 |
| Zeros (%) | 0.0% |
| Memory size | 9.6 KiB |

2020-08-25T01:01:45.509450image/svg+xmlMatplotlib v3.3.1, https://matplotlib.org/

Toggle details

- Statistics
- Histogram(s)
- Common values
- Extreme values

Quantile statistics

|  |  |
| --- | --- |
| Minimum | 0.71 |
| 5-th percentile | 1.831 |
| Q1 | 18.58 |
| median | 292 |
| Q3 | 5430.5275 |
| 95-th percentile | 51820.188 |
| Maximum | 110662.75 |
| Range | 110662.04 |
| Interquartile range (IQR) | 5411.9475 |

Descriptive statistics

|  |  |
| --- | --- |
| Standard deviation | 17801.63567 |
| Coefficient of variation (CV) | 2.189062762 |
| Kurtosis | 9.012473117 |
| Mean | 8132.08099 |
| Median Absolute Deviation (MAD) | 289.96 |
| Skewness | 2.944507684 |
| Sum | 9856082.16 |
| Variance | 316898232.7 |

- Histogram

2020-08-25T01:01:45.605346image/svg+xmlMatplotlib v3.3.1, https://matplotlib.org/ 

**Histogram with fixed size bins** (bins=10)

| Value | Count | Frequency (%) |  |
| --- | --- | --- | --- |
| 2.04 | 6 | 0.5% |  |
| 2.05 | 3 | 0.2% |  |
| 1.82 | 3 | 0.2% |  |
| 5.2 | 3 | 0.2% |  |
| 1.81 | 3 | 0.2% |  |
| 1.25 | 2 | 0.2% |  |
| 1.09 | 2 | 0.2% |  |
| 21.54 | 2 | 0.2% |  |
| 10.76 | 2 | 0.2% |  |
| 1.36 | 2 | 0.2% |  |
| 7.36 | 2 | 0.2% |  |
| 9.05 | 2 | 0.2% |  |
| 2.73 | 2 | 0.2% |  |
| 4.31 | 2 | 0.2% |  |
| 1.17 | 2 | 0.2% |  |
| 126.27 | 2 | 0.2% |  |
| 5.6 | 2 | 0.2% |  |
| 2.26 | 2 | 0.2% |  |
| 2.71 | 2 | 0.2% |  |
| 1.08 | 2 | 0.2% |  |
| 3.27 | 2 | 0.2% |  |
| 1.72 | 2 | 0.2% |  |
| 1.24 | 2 | 0.2% |  |
| 4.8 | 2 | 0.2% |  |
| 1.28 | 2 | 0.2% |  |
| Other values (1149) | 1154 | 95.2% |  |

- Minimum 5 values
- Maximum 5 values

| Value | Count | Frequency (%) |  |
| --- | --- | --- | --- |
| 0.71 | 1 | 0.1% |  |
| 0.86 | 1 | 0.1% |  |
| 0.87 | 1 | 0.1% |  |
| 0.9 | 1 | 0.1% |  |
| 0.91 | 1 | 0.1% |  |
| 0.94 | 1 | 0.1% |  |
| 0.98 | 1 | 0.1% |  |
| 1.03 | 1 | 0.1% |  |
| 1.04 | 1 | 0.1% |  |
| 1.05 | 1 | 0.1% |  |

| Value | Count | Frequency (%) |  |
| --- | --- | --- | --- |
| 110662.75 | 1 | 0.1% |  |
| 110046.16 | 1 | 0.1% |  |
| 106278.01 | 1 | 0.1% |  |
| 99824.35 | 1 | 0.1% |  |
| 98485.08 | 1 | 0.1% |  |
| 96324.7 | 1 | 0.1% |  |
| 95835.96 | 1 | 0.1% |  |
| 94002.12 | 1 | 0.1% |  |
| 92520.55 | 1 | 0.1% |  |
| 91378.88 | 1 | 0.1% |  |

X74  
Real number (ℝ≥0)

`HIGH CORRELATION`

|  |  |
| --- | --- |
| Distinct count | 1176 |
| Unique (%) | 97.0% |
| Missing | 0 |
| Missing (%) | 0.0% |
| Infinite | 0 |
| Infinite (%) | 0.0% |

|  |  |
| --- | --- |
| Mean | 8269.849257425743 |
| Minimum | 0.68 |
| Maximum | 129800.41 |
| Zeros | 0 |
| Zeros (%) | 0.0% |
| Memory size | 9.6 KiB |

2020-08-25T01:01:45.713295image/svg+xmlMatplotlib v3.3.1, https://matplotlib.org/

Toggle details

- Statistics
- Histogram(s)
- Common values
- Extreme values

Quantile statistics

|  |  |
| --- | --- |
| Minimum | 0.68 |
| 5-th percentile | 1.841 |
| Q1 | 19.22 |
| median | 301.71 |
| Q3 | 5420.755 |
| 95-th percentile | 52058.0895 |
| Maximum | 129800.41 |
| Range | 129799.73 |
| Interquartile range (IQR) | 5401.535 |

Descriptive statistics

|  |  |
| --- | --- |
| Standard deviation | 18416.02837 |
| Coefficient of variation (CV) | 2.226888036 |
| Kurtosis | 10.83422395 |
| Mean | 8269.849257 |
| Median Absolute Deviation (MAD) | 299.715 |
| Skewness | 3.132105504 |
| Sum | 10023057.3 |
| Variance | 339150100.9 |

- Histogram

2020-08-25T01:01:45.811196image/svg+xmlMatplotlib v3.3.1, https://matplotlib.org/ 

**Histogram with fixed size bins** (bins=10)

| Value | Count | Frequency (%) |  |
| --- | --- | --- | --- |
| 1.31 | 3 | 0.2% |  |
| 9.19 | 3 | 0.2% |  |
| 2.39 | 3 | 0.2% |  |
| 1.47 | 3 | 0.2% |  |
| 4.26 | 2 | 0.2% |  |
| 1.13 | 2 | 0.2% |  |
| 3.63 | 2 | 0.2% |  |
| 1.49 | 2 | 0.2% |  |
| 4.66 | 2 | 0.2% |  |
| 2.03 | 2 | 0.2% |  |
| 2.26 | 2 | 0.2% |  |
| 2.65 | 2 | 0.2% |  |
| 1.98 | 2 | 0.2% |  |
| 11.56 | 2 | 0.2% |  |
| 4.99 | 2 | 0.2% |  |
| 2.81 | 2 | 0.2% |  |
| 1.3 | 2 | 0.2% |  |
| 18.49 | 2 | 0.2% |  |
| 1.01 | 2 | 0.2% |  |
| 2.12 | 2 | 0.2% |  |
| 2.25 | 2 | 0.2% |  |
| 1.5 | 2 | 0.2% |  |
| 3.13 | 2 | 0.2% |  |
| 3.27 | 2 | 0.2% |  |
| 1.68 | 2 | 0.2% |  |
| Other values (1151) | 1158 | 95.5% |  |

- Minimum 5 values
- Maximum 5 values

| Value | Count | Frequency (%) |  |
| --- | --- | --- | --- |
| 0.68 | 1 | 0.1% |  |
| 0.89 | 1 | 0.1% |  |
| 0.92 | 1 | 0.1% |  |
| 0.93 | 2 | 0.2% |  |
| 0.96 | 1 | 0.1% |  |
| 0.98 | 1 | 0.1% |  |
| 0.99 | 1 | 0.1% |  |
| 1.01 | 2 | 0.2% |  |
| 1.02 | 1 | 0.1% |  |
| 1.03 | 1 | 0.1% |  |

| Value | Count | Frequency (%) |  |
| --- | --- | --- | --- |
| 129800.41 | 1 | 0.1% |  |
| 121442.63 | 1 | 0.1% |  |
| 116491.79 | 1 | 0.1% |  |
| 113674.45 | 1 | 0.1% |  |
| 110098.32 | 1 | 0.1% |  |
| 108625.69 | 1 | 0.1% |  |
| 105446.39 | 1 | 0.1% |  |
| 101117.16 | 1 | 0.1% |  |
| 99367.97 | 1 | 0.1% |  |
| 93169.88 | 1 | 0.1% |  |

X34  
Real number (ℝ≥0)

`HIGH CORRELATION`

|  |  |
| --- | --- |
| Distinct count | 1167 |
| Unique (%) | 96.3% |
| Missing | 0 |
| Missing (%) | 0.0% |
| Infinite | 0 |
| Infinite (%) | 0.0% |

|  |  |
| --- | --- |
| Mean | 8152.29894389439 |
| Minimum | 0.79 |
| Maximum | 117562.76 |
| Zeros | 0 |
| Zeros (%) | 0.0% |
| Memory size | 9.6 KiB |

2020-08-25T01:01:45.923207image/svg+xmlMatplotlib v3.3.1, https://matplotlib.org/

Toggle details

- Statistics
- Histogram(s)
- Common values
- Extreme values

Quantile statistics

|  |  |
| --- | --- |
| Minimum | 0.79 |
| 5-th percentile | 1.891 |
| Q1 | 18.78 |
| median | 293.98 |
| Q3 | 5351.5025 |
| 95-th percentile | 52727.371 |
| Maximum | 117562.76 |
| Range | 117561.97 |
| Interquartile range (IQR) | 5332.7225 |

Descriptive statistics

|  |  |
| --- | --- |
| Standard deviation | 17840.07304 |
| Coefficient of variation (CV) | 2.18834873 |
| Kurtosis | 9.227899798 |
| Mean | 8152.298944 |
| Median Absolute Deviation (MAD) | 291.97 |
| Skewness | 2.961601698 |
| Sum | 9880586.32 |
| Variance | 318268205.9 |

- Histogram

2020-08-25T01:01:46.022856image/svg+xmlMatplotlib v3.3.1, https://matplotlib.org/ 

**Histogram with fixed size bins** (bins=10)

| Value | Count | Frequency (%) |  |
| --- | --- | --- | --- |
| 2.05 | 4 | 0.3% |  |
| 1.12 | 3 | 0.2% |  |
| 1.72 | 3 | 0.2% |  |
| 0.92 | 3 | 0.2% |  |
| 1.48 | 3 | 0.2% |  |
| 5.53 | 2 | 0.2% |  |
| 23.75 | 2 | 0.2% |  |
| 124.06 | 2 | 0.2% |  |
| 2.68 | 2 | 0.2% |  |
| 1.53 | 2 | 0.2% |  |
| 2.06 | 2 | 0.2% |  |
| 2.71 | 2 | 0.2% |  |
| 4.45 | 2 | 0.2% |  |
| 7.95 | 2 | 0.2% |  |
| 1.21 | 2 | 0.2% |  |
| 10.91 | 2 | 0.2% |  |
| 2.85 | 2 | 0.2% |  |
| 2.86 | 2 | 0.2% |  |
| 1.15 | 2 | 0.2% |  |
| 31.09 | 2 | 0.2% |  |
| 216.47 | 2 | 0.2% |  |
| 3.6 | 2 | 0.2% |  |
| 5.23 | 2 | 0.2% |  |
| 60.6 | 2 | 0.2% |  |
| 5 | 2 | 0.2% |  |
| Other values (1142) | 1156 | 95.4% |  |

- Minimum 5 values
- Maximum 5 values

| Value | Count | Frequency (%) |  |
| --- | --- | --- | --- |
| 0.79 | 2 | 0.2% |  |
| 0.87 | 1 | 0.1% |  |
| 0.92 | 3 | 0.2% |  |
| 0.97 | 1 | 0.1% |  |
| 0.99 | 1 | 0.1% |  |
| 1.02 | 2 | 0.2% |  |
| 1.03 | 1 | 0.1% |  |
| 1.04 | 1 | 0.1% |  |
| 1.05 | 1 | 0.1% |  |
| 1.06 | 1 | 0.1% |  |

| Value | Count | Frequency (%) |  |
| --- | --- | --- | --- |
| 117562.76 | 1 | 0.1% |  |
| 116097.78 | 1 | 0.1% |  |
| 105608.89 | 1 | 0.1% |  |
| 100059.62 | 1 | 0.1% |  |
| 97245.51 | 1 | 0.1% |  |
| 96526.47 | 1 | 0.1% |  |
| 96391.23 | 1 | 0.1% |  |
| 90093.77 | 1 | 0.1% |  |
| 88073.59 | 1 | 0.1% |  |
| 87595.25 | 1 | 0.1% |  |

X91  
Real number (ℝ≥0)

`HIGH CORRELATION`

|  |  |
| --- | --- |
| Distinct count | 1174 |
| Unique (%) | 96.9% |
| Missing | 0 |
| Missing (%) | 0.0% |
| Infinite | 0 |
| Infinite (%) | 0.0% |

|  |  |
| --- | --- |
| Mean | 8115.970321782179 |
| Minimum | 0.91 |
| Maximum | 110158.85 |
| Zeros | 0 |
| Zeros (%) | 0.0% |
| Memory size | 9.6 KiB |

2020-08-25T01:01:46.135981image/svg+xmlMatplotlib v3.3.1, https://matplotlib.org/

Toggle details

- Statistics
- Histogram(s)
- Common values
- Extreme values

Quantile statistics

|  |  |
| --- | --- |
| Minimum | 0.91 |
| 5-th percentile | 1.8565 |
| Q1 | 19.3575 |
| median | 295.945 |
| Q3 | 5345.99 |
| 95-th percentile | 52125.142 |
| Maximum | 110158.85 |
| Range | 110157.94 |
| Interquartile range (IQR) | 5326.6325 |

Descriptive statistics

|  |  |
| --- | --- |
| Standard deviation | 17689.41909 |
| Coefficient of variation (CV) | 2.179581539 |
| Kurtosis | 8.864728952 |
| Mean | 8115.970322 |
| Median Absolute Deviation (MAD) | 293.975 |
| Skewness | 2.923549539 |
| Sum | 9836556.03 |
| Variance | 312915547.7 |

- Histogram

2020-08-25T01:01:46.245045image/svg+xmlMatplotlib v3.3.1, https://matplotlib.org/ 

**Histogram with fixed size bins** (bins=10)

| Value | Count | Frequency (%) |  |
| --- | --- | --- | --- |
| 1.24 | 3 | 0.2% |  |
| 2.68 | 3 | 0.2% |  |
| 1.1 | 3 | 0.2% |  |
| 0.96 | 3 | 0.2% |  |
| 1.93 | 3 | 0.2% |  |
| 1.71 | 3 | 0.2% |  |
| 2.16 | 2 | 0.2% |  |
| 5.11 | 2 | 0.2% |  |
| 1.92 | 2 | 0.2% |  |
| 26.48 | 2 | 0.2% |  |
| 1.97 | 2 | 0.2% |  |
| 2.05 | 2 | 0.2% |  |
| 2.86 | 2 | 0.2% |  |
| 22.36 | 2 | 0.2% |  |
| 3.86 | 2 | 0.2% |  |
| 1.38 | 2 | 0.2% |  |
| 1.59 | 2 | 0.2% |  |
| 3.39 | 2 | 0.2% |  |
| 1.44 | 2 | 0.2% |  |
| 4.82 | 2 | 0.2% |  |
| 2.11 | 2 | 0.2% |  |
| 1.08 | 2 | 0.2% |  |
| 2.76 | 2 | 0.2% |  |
| 6.54 | 2 | 0.2% |  |
| 2.62 | 2 | 0.2% |  |
| Other values (1149) | 1156 | 95.4% |  |

- Minimum 5 values
- Maximum 5 values

| Value | Count | Frequency (%) |  |
| --- | --- | --- | --- |
| 0.91 | 1 | 0.1% |  |
| 0.93 | 1 | 0.1% |  |
| 0.95 | 1 | 0.1% |  |
| 0.96 | 3 | 0.2% |  |
| 0.97 | 1 | 0.1% |  |
| 1.03 | 1 | 0.1% |  |
| 1.08 | 2 | 0.2% |  |
| 1.1 | 3 | 0.2% |  |
| 1.11 | 1 | 0.1% |  |
| 1.12 | 1 | 0.1% |  |

| Value | Count | Frequency (%) |  |
| --- | --- | --- | --- |
| 110158.85 | 1 | 0.1% |  |
| 104682.06 | 1 | 0.1% |  |
| 102921.22 | 1 | 0.1% |  |
| 101072.9 | 1 | 0.1% |  |
| 101058.01 | 1 | 0.1% |  |
| 99175.92 | 1 | 0.1% |  |
| 98221.62 | 1 | 0.1% |  |
| 96492.55 | 1 | 0.1% |  |
| 91662.24 | 1 | 0.1% |  |
| 86662.42 | 1 | 0.1% |  |

X5  
Real number (ℝ≥0)

`HIGH CORRELATION`

|  |  |
| --- | --- |
| Distinct count | 1174 |
| Unique (%) | 96.9% |
| Missing | 0 |
| Missing (%) | 0.0% |
| Infinite | 0 |
| Infinite (%) | 0.0% |

|  |  |
| --- | --- |
| Mean | 8128.297211221121 |
| Minimum | 0.88 |
| Maximum | 113000.47 |
| Zeros | 0 |
| Zeros (%) | 0.0% |
| Memory size | 9.6 KiB |

2020-08-25T01:01:46.354244image/svg+xmlMatplotlib v3.3.1, https://matplotlib.org/

Toggle details

- Statistics
- Histogram(s)
- Common values
- Extreme values

Quantile statistics

|  |  |
| --- | --- |
| Minimum | 0.88 |
| 5-th percentile | 1.8855 |
| Q1 | 19.21 |
| median | 295.115 |
| Q3 | 5321.9875 |
| 95-th percentile | 52753.018 |
| Maximum | 113000.47 |
| Range | 112999.59 |
| Interquartile range (IQR) | 5302.7775 |

Descriptive statistics

|  |  |
| --- | --- |
| Standard deviation | 17846.75796 |
| Coefficient of variation (CV) | 2.195633046 |
| Kurtosis | 9.226939876 |
| Mean | 8128.297211 |
| Median Absolute Deviation (MAD) | 293.09 |
| Skewness | 2.970150623 |
| Sum | 9851496.22 |
| Variance | 318506769.8 |

- Histogram

2020-08-25T01:01:46.454933image/svg+xmlMatplotlib v3.3.1, https://matplotlib.org/ 

**Histogram with fixed size bins** (bins=10)

| Value | Count | Frequency (%) |  |
| --- | --- | --- | --- |
| 1.13 | 3 | 0.2% |  |
| 1.39 | 3 | 0.2% |  |
| 3.9 | 3 | 0.2% |  |
| 0.95 | 3 | 0.2% |  |
| 5.05 | 2 | 0.2% |  |
| 3.69 | 2 | 0.2% |  |
| 1.54 | 2 | 0.2% |  |
| 1.86 | 2 | 0.2% |  |
| 1.61 | 2 | 0.2% |  |
| 1.03 | 2 | 0.2% |  |
| 3.2 | 2 | 0.2% |  |
| 1.98 | 2 | 0.2% |  |
| 141.78 | 2 | 0.2% |  |
| 68.17 | 2 | 0.2% |  |
| 1.59 | 2 | 0.2% |  |
| 1.17 | 2 | 0.2% |  |
| 44.3 | 2 | 0.2% |  |
| 2.84 | 2 | 0.2% |  |
| 9.58 | 2 | 0.2% |  |
| 2.35 | 2 | 0.2% |  |
| 2.15 | 2 | 0.2% |  |
| 2.43 | 2 | 0.2% |  |
| 2.07 | 2 | 0.2% |  |
| 4.8 | 2 | 0.2% |  |
| 1.34 | 2 | 0.2% |  |
| Other values (1149) | 1158 | 95.5% |  |

- Minimum 5 values
- Maximum 5 values

| Value | Count | Frequency (%) |  |
| --- | --- | --- | --- |
| 0.88 | 1 | 0.1% |  |
| 0.92 | 1 | 0.1% |  |
| 0.95 | 3 | 0.2% |  |
| 0.96 | 1 | 0.1% |  |
| 0.97 | 2 | 0.2% |  |
| 0.99 | 1 | 0.1% |  |
| 1.03 | 2 | 0.2% |  |
| 1.04 | 1 | 0.1% |  |
| 1.05 | 1 | 0.1% |  |
| 1.09 | 1 | 0.1% |  |

| Value | Count | Frequency (%) |  |
| --- | --- | --- | --- |
| 113000.47 | 1 | 0.1% |  |
| 108930.41 | 1 | 0.1% |  |
| 106147.52 | 1 | 0.1% |  |
| 103089.26 | 1 | 0.1% |  |
| 102470.56 | 1 | 0.1% |  |
| 102205.12 | 1 | 0.1% |  |
| 100339.57 | 1 | 0.1% |  |
| 97517.8 | 1 | 0.1% |  |
| 92759.27 | 1 | 0.1% |  |
| 83642.86 | 1 | 0.1% |  |

X77  
Real number (ℝ≥0)

`HIGH CORRELATION`

|  |  |
| --- | --- |
| Distinct count | 1170 |
| Unique (%) | 96.5% |
| Missing | 0 |
| Missing (%) | 0.0% |
| Infinite | 0 |
| Infinite (%) | 0.0% |

|  |  |
| --- | --- |
| Mean | 8255.866336633664 |
| Minimum | 0.64 |
| Maximum | 112284.99 |
| Zeros | 0 |
| Zeros (%) | 0.0% |
| Memory size | 9.6 KiB |

2020-08-25T01:01:46.562927image/svg+xmlMatplotlib v3.3.1, https://matplotlib.org/

Toggle details

- Statistics
- Histogram(s)
- Common values
- Extreme values

Quantile statistics

|  |  |
| --- | --- |
| Minimum | 0.64 |
| 5-th percentile | 1.781 |
| Q1 | 18.99 |
| median | 305.305 |
| Q3 | 5198.3375 |
| 95-th percentile | 50920.2405 |
| Maximum | 112284.99 |
| Range | 112284.35 |
| Interquartile range (IQR) | 5179.3475 |

Descriptive statistics

|  |  |
| --- | --- |
| Standard deviation | 18299.87238 |
| Coefficient of variation (CV) | 2.216590196 |
| Kurtosis | 9.575378032 |
| Mean | 8255.866337 |
| Median Absolute Deviation (MAD) | 303.27 |
| Skewness | 3.024104504 |
| Sum | 10006110 |
| Variance | 334885329.2 |

- Histogram

2020-08-25T01:01:46.662025image/svg+xmlMatplotlib v3.3.1, https://matplotlib.org/ 

**Histogram with fixed size bins** (bins=10)

| Value | Count | Frequency (%) |  |
| --- | --- | --- | --- |
| 1.37 | 3 | 0.2% |  |
| 1.48 | 3 | 0.2% |  |
| 1.05 | 3 | 0.2% |  |
| 1.03 | 3 | 0.2% |  |
| 1.99 | 2 | 0.2% |  |
| 2.3 | 2 | 0.2% |  |
| 1.27 | 2 | 0.2% |  |
| 4.06 | 2 | 0.2% |  |
| 0.97 | 2 | 0.2% |  |
| 1.21 | 2 | 0.2% |  |
| 1.19 | 2 | 0.2% |  |
| 2.58 | 2 | 0.2% |  |
| 2.73 | 2 | 0.2% |  |
| 31.89 | 2 | 0.2% |  |
| 7.97 | 2 | 0.2% |  |
| 2.53 | 2 | 0.2% |  |
| 3.13 | 2 | 0.2% |  |
| 2.03 | 2 | 0.2% |  |
| 3.96 | 2 | 0.2% |  |
| 8.74 | 2 | 0.2% |  |
| 1.9 | 2 | 0.2% |  |
| 2.09 | 2 | 0.2% |  |
| 1.93 | 2 | 0.2% |  |
| 2.16 | 2 | 0.2% |  |
| 2.83 | 2 | 0.2% |  |
| Other values (1145) | 1158 | 95.5% |  |

- Minimum 5 values
- Maximum 5 values

| Value | Count | Frequency (%) |  |
| --- | --- | --- | --- |
| 0.64 | 1 | 0.1% |  |
| 0.92 | 1 | 0.1% |  |
| 0.93 | 2 | 0.2% |  |
| 0.97 | 2 | 0.2% |  |
| 0.99 | 1 | 0.1% |  |
| 1.03 | 3 | 0.2% |  |
| 1.04 | 1 | 0.1% |  |
| 1.05 | 3 | 0.2% |  |
| 1.06 | 1 | 0.1% |  |
| 1.07 | 1 | 0.1% |  |

| Value | Count | Frequency (%) |  |
| --- | --- | --- | --- |
| 112284.99 | 1 | 0.1% |  |
| 109124.59 | 1 | 0.1% |  |
| 103990.75 | 1 | 0.1% |  |
| 103852.61 | 1 | 0.1% |  |
| 103710.63 | 1 | 0.1% |  |
| 103503.27 | 1 | 0.1% |  |
| 102647.2 | 1 | 0.1% |  |
| 97519.5 | 1 | 0.1% |  |
| 96861.93 | 1 | 0.1% |  |
| 94953.83 | 1 | 0.1% |  |

X78  
Real number (ℝ≥0)

`HIGH CORRELATION`

|  |  |
| --- | --- |
| Distinct count | 1152 |
| Unique (%) | 95.0% |
| Missing | 0 |
| Missing (%) | 0.0% |
| Infinite | 0 |
| Infinite (%) | 0.0% |

|  |  |
| --- | --- |
| Mean | 8235.94214521452 |
| Minimum | 0.54 |
| Maximum | 122485.87 |
| Zeros | 0 |
| Zeros (%) | 0.0% |
| Memory size | 9.6 KiB |

2020-08-25T01:01:46.770236image/svg+xmlMatplotlib v3.3.1, https://matplotlib.org/

Toggle details

- Statistics
- Histogram(s)
- Common values
- Extreme values

Quantile statistics

|  |  |
| --- | --- |
| Minimum | 0.54 |
| 5-th percentile | 1.761 |
| Q1 | 18.9475 |
| median | 300.82 |
| Q3 | 5306.425 |
| 95-th percentile | 52768.7335 |
| Maximum | 122485.87 |
| Range | 122485.33 |
| Interquartile range (IQR) | 5287.4775 |

Descriptive statistics

|  |  |
| --- | --- |
| Standard deviation | 18301.06364 |
| Coefficient of variation (CV) | 2.222097159 |
| Kurtosis | 9.91715723 |
| Mean | 8235.942145 |
| Median Absolute Deviation (MAD) | 298.81 |
| Skewness | 3.053218663 |
| Sum | 9981961.88 |
| Variance | 334928930.4 |

- Histogram

2020-08-25T01:01:46.870510image/svg+xmlMatplotlib v3.3.1, https://matplotlib.org/ 

**Histogram with fixed size bins** (bins=10)

| Value | Count | Frequency (%) |  |
| --- | --- | --- | --- |
| 2.15 | 4 | 0.3% |  |
| 1 | 4 | 0.3% |  |
| 0.97 | 4 | 0.3% |  |
| 1.64 | 3 | 0.2% |  |
| 2.11 | 3 | 0.2% |  |
| 1.3 | 3 | 0.2% |  |
| 1.19 | 3 | 0.2% |  |
| 1.75 | 3 | 0.2% |  |
| 1.37 | 3 | 0.2% |  |
| 1.63 | 3 | 0.2% |  |
| 1.85 | 3 | 0.2% |  |
| 3.64 | 2 | 0.2% |  |
| 10.97 | 2 | 0.2% |  |
| 5.14 | 2 | 0.2% |  |
| 8.33 | 2 | 0.2% |  |
| 3.92 | 2 | 0.2% |  |
| 1.45 | 2 | 0.2% |  |
| 1.99 | 2 | 0.2% |  |
| 2.68 | 2 | 0.2% |  |
| 14.15 | 2 | 0.2% |  |
| 5.92 | 2 | 0.2% |  |
| 4.22 | 2 | 0.2% |  |
| 1.29 | 2 | 0.2% |  |
| 1.73 | 2 | 0.2% |  |
| 4.96 | 2 | 0.2% |  |
| Other values (1127) | 1148 | 94.7% |  |

- Minimum 5 values
- Maximum 5 values

| Value | Count | Frequency (%) |  |
| --- | --- | --- | --- |
| 0.54 | 1 | 0.1% |  |
| 0.97 | 4 | 0.3% |  |
| 0.99 | 1 | 0.1% |  |
| 1 | 4 | 0.3% |  |
| 1.02 | 2 | 0.2% |  |
| 1.03 | 1 | 0.1% |  |
| 1.04 | 1 | 0.1% |  |
| 1.05 | 1 | 0.1% |  |
| 1.12 | 1 | 0.1% |  |
| 1.13 | 2 | 0.2% |  |

| Value | Count | Frequency (%) |  |
| --- | --- | --- | --- |
| 122485.87 | 1 | 0.1% |  |
| 113539.46 | 1 | 0.1% |  |
| 110222.9 | 1 | 0.1% |  |
| 106563.41 | 1 | 0.1% |  |
| 104879.88 | 1 | 0.1% |  |
| 101163.94 | 1 | 0.1% |  |
| 99440.63 | 1 | 0.1% |  |
| 99414.04 | 1 | 0.1% |  |
| 95925.21 | 1 | 0.1% |  |
| 95033.58 | 1 | 0.1% |  |

X13  
Real number (ℝ≥0)

`HIGH CORRELATION`

|  |  |
| --- | --- |
| Distinct count | 1168 |
| Unique (%) | 96.4% |
| Missing | 0 |
| Missing (%) | 0.0% |
| Infinite | 0 |
| Infinite (%) | 0.0% |

|  |  |
| --- | --- |
| Mean | 8122.662136963696 |
| Minimum | 0.84 |
| Maximum | 137461.68 |
| Zeros | 0 |
| Zeros (%) | 0.0% |
| Memory size | 9.6 KiB |

2020-08-25T01:01:46.983971image/svg+xmlMatplotlib v3.3.1, https://matplotlib.org/

Toggle details

- Statistics
- Histogram(s)
- Common values
- Extreme values

Quantile statistics

|  |  |
| --- | --- |
| Minimum | 0.84 |
| 5-th percentile | 1.85 |
| Q1 | 19.25 |
| median | 298.15 |
| Q3 | 5254.4625 |
| 95-th percentile | 52384.3475 |
| Maximum | 137461.68 |
| Range | 137460.84 |
| Interquartile range (IQR) | 5235.2125 |

Descriptive statistics

|  |  |
| --- | --- |
| Standard deviation | 17978.71206 |
| Coefficient of variation (CV) | 2.213401438 |
| Kurtosis | 10.80284182 |
| Mean | 8122.662137 |
| Median Absolute Deviation (MAD) | 296.19 |
| Skewness | 3.108209421 |
| Sum | 9844666.51 |
| Variance | 323234087.2 |

- Histogram

2020-08-25T01:01:47.087934image/svg+xmlMatplotlib v3.3.1, https://matplotlib.org/ 

**Histogram with fixed size bins** (bins=10)

| Value | Count | Frequency (%) |  |
| --- | --- | --- | --- |
| 1.85 | 3 | 0.2% |  |
| 1.99 | 3 | 0.2% |  |
| 2.87 | 3 | 0.2% |  |
| 5.11 | 3 | 0.2% |  |
| 1.74 | 2 | 0.2% |  |
| 4.14 | 2 | 0.2% |  |
| 4.17 | 2 | 0.2% |  |
| 1.31 | 2 | 0.2% |  |
| 36.14 | 2 | 0.2% |  |
| 2.35 | 2 | 0.2% |  |
| 1.36 | 2 | 0.2% |  |
| 11.21 | 2 | 0.2% |  |
| 4.67 | 2 | 0.2% |  |
| 13.19 | 2 | 0.2% |  |
| 3.6 | 2 | 0.2% |  |
| 1.46 | 2 | 0.2% |  |
| 1.43 | 2 | 0.2% |  |
| 0.91 | 2 | 0.2% |  |
| 23.01 | 2 | 0.2% |  |
| 3.58 | 2 | 0.2% |  |
| 1.97 | 2 | 0.2% |  |
| 9.74 | 2 | 0.2% |  |
| 1.4 | 2 | 0.2% |  |
| 3.22 | 2 | 0.2% |  |
| 1.62 | 2 | 0.2% |  |
| Other values (1143) | 1158 | 95.5% |  |

- Minimum 5 values
- Maximum 5 values

| Value | Count | Frequency (%) |  |
| --- | --- | --- | --- |
| 0.84 | 1 | 0.1% |  |
| 0.91 | 2 | 0.2% |  |
| 0.92 | 1 | 0.1% |  |
| 0.94 | 1 | 0.1% |  |
| 0.95 | 1 | 0.1% |  |
| 1 | 1 | 0.1% |  |
| 1.02 | 2 | 0.2% |  |
| 1.04 | 1 | 0.1% |  |
| 1.05 | 1 | 0.1% |  |
| 1.06 | 1 | 0.1% |  |

| Value | Count | Frequency (%) |  |
| --- | --- | --- | --- |
| 137461.68 | 1 | 0.1% |  |
| 116368.39 | 1 | 0.1% |  |
| 113454.76 | 1 | 0.1% |  |
| 112838.09 | 1 | 0.1% |  |
| 112356.17 | 1 | 0.1% |  |
| 104239.27 | 1 | 0.1% |  |
| 95083.31 | 1 | 0.1% |  |
| 89504.12 | 1 | 0.1% |  |
| 89282.68 | 1 | 0.1% |  |
| 82334.25 | 1 | 0.1% |  |

target  
Boolean

|  |  |
| --- | --- |
| Distinct count | 2 |
| Unique (%) | 0.2% |
| Missing | 0 |
| Missing (%) | 0.0% |
| Memory size | 9.6 KiB |

|  |  |
| --- | --- |
| 1 | 606 |
| 0 | 606 |

Toggle details

- Frequency Table

| Value | Count | Frequency (%) |  |
| --- | --- | --- | --- |
| 1 | 606 | 50.0% |  |
| 0 | 606 | 50.0% |  |

# Interactions

X84 X54 X71 X46 X45 X40 X23 X81 X11 X1 X19 X31 X74 X34 X91 X5 X77 X78 X13

X84 X54 X71 X46 X45 X40 X23 X81 X11 X1 X19 X31 X74 X34 X91 X5 X77 X78 X13

2020-08-25T01:00:49.933657image/svg+xmlMatplotlib v3.3.1, https://matplotlib.org/

2020-08-25T01:00:50.072950image/svg+xmlMatplotlib v3.3.1, https://matplotlib.org/

2020-08-25T01:00:50.372298image/svg+xmlMatplotlib v3.3.1, https://matplotlib.org/

2020-08-25T01:00:50.503450image/svg+xmlMatplotlib v3.3.1, https://matplotlib.org/

2020-08-25T01:00:50.635929image/svg+xmlMatplotlib v3.3.1, https://matplotlib.org/

2020-08-25T01:00:50.774236image/svg+xmlMatplotlib v3.3.1, https://matplotlib.org/

2020-08-25T01:00:50.905761image/svg+xmlMatplotlib v3.3.1, https://matplotlib.org/

2020-08-25T01:00:51.048434image/svg+xmlMatplotlib v3.3.1, https://matplotlib.org/

2020-08-25T01:00:51.176547image/svg+xmlMatplotlib v3.3.1, https://matplotlib.org/

2020-08-25T01:00:51.309794image/svg+xmlMatplotlib v3.3.1, https://matplotlib.org/

2020-08-25T01:00:51.441955image/svg+xmlMatplotlib v3.3.1, https://matplotlib.org/

2020-08-25T01:00:51.573177image/svg+xmlMatplotlib v3.3.1, https://matplotlib.org/

2020-08-25T01:00:51.703473image/svg+xmlMatplotlib v3.3.1, https://matplotlib.org/

2020-08-25T01:00:51.836210image/svg+xmlMatplotlib v3.3.1, https://matplotlib.org/

2020-08-25T01:00:51.967737image/svg+xmlMatplotlib v3.3.1, https://matplotlib.org/

2020-08-25T01:00:52.098039image/svg+xmlMatplotlib v3.3.1, https://matplotlib.org/

2020-08-25T01:00:52.225965image/svg+xmlMatplotlib v3.3.1, https://matplotlib.org/

2020-08-25T01:00:52.354652image/svg+xmlMatplotlib v3.3.1, https://matplotlib.org/

2020-08-25T01:00:52.487121image/svg+xmlMatplotlib v3.3.1, https://matplotlib.org/

X84 X54 X71 X46 X45 X40 X23 X81 X11 X1 X19 X31 X74 X34 X91 X5 X77 X78 X13

2020-08-25T01:00:52.637555image/svg+xmlMatplotlib v3.3.1, https://matplotlib.org/

2020-08-25T01:00:52.767001image/svg+xmlMatplotlib v3.3.1, https://matplotlib.org/

2020-08-25T01:00:52.895342image/svg+xmlMatplotlib v3.3.1, https://matplotlib.org/

2020-08-25T01:00:53.030220image/svg+xmlMatplotlib v3.3.1, https://matplotlib.org/

2020-08-25T01:00:53.169757image/svg+xmlMatplotlib v3.3.1, https://matplotlib.org/

2020-08-25T01:00:53.302793image/svg+xmlMatplotlib v3.3.1, https://matplotlib.org/

2020-08-25T01:00:53.441894image/svg+xmlMatplotlib v3.3.1, https://matplotlib.org/

2020-08-25T01:00:53.583443image/svg+xmlMatplotlib v3.3.1, https://matplotlib.org/

2020-08-25T01:00:53.722687image/svg+xmlMatplotlib v3.3.1, https://matplotlib.org/

2020-08-25T01:00:53.858649image/svg+xmlMatplotlib v3.3.1, https://matplotlib.org/

2020-08-25T01:00:53.996326image/svg+xmlMatplotlib v3.3.1, https://matplotlib.org/

2020-08-25T01:00:54.130556image/svg+xmlMatplotlib v3.3.1, https://matplotlib.org/

2020-08-25T01:00:54.260958image/svg+xmlMatplotlib v3.3.1, https://matplotlib.org/

2020-08-25T01:00:54.571038image/svg+xmlMatplotlib v3.3.1, https://matplotlib.org/

2020-08-25T01:00:54.708742image/svg+xmlMatplotlib v3.3.1, https://matplotlib.org/

2020-08-25T01:00:54.846673image/svg+xmlMatplotlib v3.3.1, https://matplotlib.org/

2020-08-25T01:00:54.977393image/svg+xmlMatplotlib v3.3.1, https://matplotlib.org/

2020-08-25T01:00:55.113064image/svg+xmlMatplotlib v3.3.1, https://matplotlib.org/

2020-08-25T01:00:55.249221image/svg+xmlMatplotlib v3.3.1, https://matplotlib.org/

X84 X54 X71 X46 X45 X40 X23 X81 X11 X1 X19 X31 X74 X34 X91 X5 X77 X78 X13

2020-08-25T01:00:55.395622image/svg+xmlMatplotlib v3.3.1, https://matplotlib.org/

2020-08-25T01:00:55.530723image/svg+xmlMatplotlib v3.3.1, https://matplotlib.org/

2020-08-25T01:00:55.678414image/svg+xmlMatplotlib v3.3.1, https://matplotlib.org/

2020-08-25T01:00:55.821033image/svg+xmlMatplotlib v3.3.1, https://matplotlib.org/

2020-08-25T01:00:55.961945image/svg+xmlMatplotlib v3.3.1, https://matplotlib.org/

2020-08-25T01:00:56.157957image/svg+xmlMatplotlib v3.3.1, https://matplotlib.org/

2020-08-25T01:00:56.343865image/svg+xmlMatplotlib v3.3.1, https://matplotlib.org/

2020-08-25T01:00:56.493545image/svg+xmlMatplotlib v3.3.1, https://matplotlib.org/

2020-08-25T01:00:56.630405image/svg+xmlMatplotlib v3.3.1, https://matplotlib.org/

2020-08-25T01:00:56.773757image/svg+xmlMatplotlib v3.3.1, https://matplotlib.org/

2020-08-25T01:00:56.922391image/svg+xmlMatplotlib v3.3.1, https://matplotlib.org/

2020-08-25T01:00:57.065473image/svg+xmlMatplotlib v3.3.1, https://matplotlib.org/

2020-08-25T01:00:57.204043image/svg+xmlMatplotlib v3.3.1, https://matplotlib.org/

2020-08-25T01:00:57.343040image/svg+xmlMatplotlib v3.3.1, https://matplotlib.org/

2020-08-25T01:00:57.484754image/svg+xmlMatplotlib v3.3.1, https://matplotlib.org/

2020-08-25T01:00:57.629412image/svg+xmlMatplotlib v3.3.1, https://matplotlib.org/

2020-08-25T01:00:57.778186image/svg+xmlMatplotlib v3.3.1, https://matplotlib.org/

2020-08-25T01:00:57.915422image/svg+xmlMatplotlib v3.3.1, https://matplotlib.org/

2020-08-25T01:00:58.051376image/svg+xmlMatplotlib v3.3.1, https://matplotlib.org/

X84 X54 X71 X46 X45 X40 X23 X81 X11 X1 X19 X31 X74 X34 X91 X5 X77 X78 X13

2020-08-25T01:00:58.195797image/svg+xmlMatplotlib v3.3.1, https://matplotlib.org/

2020-08-25T01:00:58.331906image/svg+xmlMatplotlib v3.3.1, https://matplotlib.org/

2020-08-25T01:00:58.464862image/svg+xmlMatplotlib v3.3.1, https://matplotlib.org/

2020-08-25T01:00:58.608698image/svg+xmlMatplotlib v3.3.1, https://matplotlib.org/

2020-08-25T01:00:58.765985image/svg+xmlMatplotlib v3.3.1, https://matplotlib.org/

2020-08-25T01:00:59.075156image/svg+xmlMatplotlib v3.3.1, https://matplotlib.org/

2020-08-25T01:00:59.216468image/svg+xmlMatplotlib v3.3.1, https://matplotlib.org/

2020-08-25T01:00:59.359740image/svg+xmlMatplotlib v3.3.1, https://matplotlib.org/

2020-08-25T01:00:59.495752image/svg+xmlMatplotlib v3.3.1, https://matplotlib.org/

2020-08-25T01:00:59.635257image/svg+xmlMatplotlib v3.3.1, https://matplotlib.org/

2020-08-25T01:00:59.773746image/svg+xmlMatplotlib v3.3.1, https://matplotlib.org/

2020-08-25T01:00:59.908479image/svg+xmlMatplotlib v3.3.1, https://matplotlib.org/

2020-08-25T01:01:00.047681image/svg+xmlMatplotlib v3.3.1, https://matplotlib.org/

2020-08-25T01:01:00.189684image/svg+xmlMatplotlib v3.3.1, https://matplotlib.org/

2020-08-25T01:01:00.324648image/svg+xmlMatplotlib v3.3.1, https://matplotlib.org/

2020-08-25T01:01:00.458779image/svg+xmlMatplotlib v3.3.1, https://matplotlib.org/

2020-08-25T01:01:00.590863image/svg+xmlMatplotlib v3.3.1, https://matplotlib.org/

2020-08-25T01:01:00.726645image/svg+xmlMatplotlib v3.3.1, https://matplotlib.org/

2020-08-25T01:01:00.863809image/svg+xmlMatplotlib v3.3.1, https://matplotlib.org/

X84 X54 X71 X46 X45 X40 X23 X81 X11 X1 X19 X31 X74 X34 X91 X5 X77 X78 X13

2020-08-25T01:01:01.006217image/svg+xmlMatplotlib v3.3.1, https://matplotlib.org/

2020-08-25T01:01:01.144511image/svg+xmlMatplotlib v3.3.1, https://matplotlib.org/

2020-08-25T01:01:01.280335image/svg+xmlMatplotlib v3.3.1, https://matplotlib.org/

2020-08-25T01:01:01.418563image/svg+xmlMatplotlib v3.3.1, https://matplotlib.org/

2020-08-25T01:01:01.555624image/svg+xmlMatplotlib v3.3.1, https://matplotlib.org/

2020-08-25T01:01:01.693404image/svg+xmlMatplotlib v3.3.1, https://matplotlib.org/

2020-08-25T01:01:01.832130image/svg+xmlMatplotlib v3.3.1, https://matplotlib.org/

2020-08-25T01:01:01.975863image/svg+xmlMatplotlib v3.3.1, https://matplotlib.org/

2020-08-25T01:01:02.111846image/svg+xmlMatplotlib v3.3.1, https://matplotlib.org/

2020-08-25T01:01:02.254565image/svg+xmlMatplotlib v3.3.1, https://matplotlib.org/

2020-08-25T01:01:02.391659image/svg+xmlMatplotlib v3.3.1, https://matplotlib.org/

2020-08-25T01:01:02.528389image/svg+xmlMatplotlib v3.3.1, https://matplotlib.org/

2020-08-25T01:01:02.664686image/svg+xmlMatplotlib v3.3.1, https://matplotlib.org/

2020-08-25T01:01:02.807120image/svg+xmlMatplotlib v3.3.1, https://matplotlib.org/

2020-08-25T01:01:02.947888image/svg+xmlMatplotlib v3.3.1, https://matplotlib.org/

2020-08-25T01:01:03.086490image/svg+xmlMatplotlib v3.3.1, https://matplotlib.org/

2020-08-25T01:01:03.401811image/svg+xmlMatplotlib v3.3.1, https://matplotlib.org/

2020-08-25T01:01:03.535237image/svg+xmlMatplotlib v3.3.1, https://matplotlib.org/

2020-08-25T01:01:03.679099image/svg+xmlMatplotlib v3.3.1, https://matplotlib.org/

X84 X54 X71 X46 X45 X40 X23 X81 X11 X1 X19 X31 X74 X34 X91 X5 X77 X78 X13

2020-08-25T01:01:03.834433image/svg+xmlMatplotlib v3.3.1, https://matplotlib.org/

2020-08-25T01:01:03.972672image/svg+xmlMatplotlib v3.3.1, https://matplotlib.org/

2020-08-25T01:01:04.108422image/svg+xmlMatplotlib v3.3.1, https://matplotlib.org/

2020-08-25T01:01:04.251757image/svg+xmlMatplotlib v3.3.1, https://matplotlib.org/

2020-08-25T01:01:04.389901image/svg+xmlMatplotlib v3.3.1, https://matplotlib.org/

2020-08-25T01:01:04.530571image/svg+xmlMatplotlib v3.3.1, https://matplotlib.org/

2020-08-25T01:01:04.674587image/svg+xmlMatplotlib v3.3.1, https://matplotlib.org/

2020-08-25T01:01:04.818705image/svg+xmlMatplotlib v3.3.1, https://matplotlib.org/

2020-08-25T01:01:04.952060image/svg+xmlMatplotlib v3.3.1, https://matplotlib.org/

2020-08-25T01:01:05.089276image/svg+xmlMatplotlib v3.3.1, https://matplotlib.org/

2020-08-25T01:01:05.228956image/svg+xmlMatplotlib v3.3.1, https://matplotlib.org/

2020-08-25T01:01:05.365521image/svg+xmlMatplotlib v3.3.1, https://matplotlib.org/

2020-08-25T01:01:05.500633image/svg+xmlMatplotlib v3.3.1, https://matplotlib.org/

2020-08-25T01:01:05.639566image/svg+xmlMatplotlib v3.3.1, https://matplotlib.org/

2020-08-25T01:01:05.781335image/svg+xmlMatplotlib v3.3.1, https://matplotlib.org/

2020-08-25T01:01:05.914636image/svg+xmlMatplotlib v3.3.1, https://matplotlib.org/

2020-08-25T01:01:06.049453image/svg+xmlMatplotlib v3.3.1, https://matplotlib.org/

2020-08-25T01:01:06.184718image/svg+xmlMatplotlib v3.3.1, https://matplotlib.org/

2020-08-25T01:01:06.326036image/svg+xmlMatplotlib v3.3.1, https://matplotlib.org/

X84 X54 X71 X46 X45 X40 X23 X81 X11 X1 X19 X31 X74 X34 X91 X5 X77 X78 X13

2020-08-25T01:01:06.469043image/svg+xmlMatplotlib v3.3.1, https://matplotlib.org/

2020-08-25T01:01:06.604974image/svg+xmlMatplotlib v3.3.1, https://matplotlib.org/

2020-08-25T01:01:06.743887image/svg+xmlMatplotlib v3.3.1, https://matplotlib.org/

2020-08-25T01:01:06.887363image/svg+xmlMatplotlib v3.3.1, https://matplotlib.org/

2020-08-25T01:01:07.028075image/svg+xmlMatplotlib v3.3.1, https://matplotlib.org/

2020-08-25T01:01:07.169724image/svg+xmlMatplotlib v3.3.1, https://matplotlib.org/

2020-08-25T01:01:07.316204image/svg+xmlMatplotlib v3.3.1, https://matplotlib.org/

2020-08-25T01:01:07.463910image/svg+xmlMatplotlib v3.3.1, https://matplotlib.org/

2020-08-25T01:01:07.772533image/svg+xmlMatplotlib v3.3.1, https://matplotlib.org/

2020-08-25T01:01:07.918316image/svg+xmlMatplotlib v3.3.1, https://matplotlib.org/

2020-08-25T01:01:08.065845image/svg+xmlMatplotlib v3.3.1, https://matplotlib.org/

2020-08-25T01:01:08.207639image/svg+xmlMatplotlib v3.3.1, https://matplotlib.org/

2020-08-25T01:01:08.351774image/svg+xmlMatplotlib v3.3.1, https://matplotlib.org/

2020-08-25T01:01:08.496956image/svg+xmlMatplotlib v3.3.1, https://matplotlib.org/

2020-08-25T01:01:08.641214image/svg+xmlMatplotlib v3.3.1, https://matplotlib.org/

2020-08-25T01:01:08.786265image/svg+xmlMatplotlib v3.3.1, https://matplotlib.org/

2020-08-25T01:01:08.937384image/svg+xmlMatplotlib v3.3.1, https://matplotlib.org/

2020-08-25T01:01:09.089455image/svg+xmlMatplotlib v3.3.1, https://matplotlib.org/

2020-08-25T01:01:09.233261image/svg+xmlMatplotlib v3.3.1, https://matplotlib.org/

X84 X54 X71 X46 X45 X40 X23 X81 X11 X1 X19 X31 X74 X34 X91 X5 X77 X78 X13

2020-08-25T01:01:09.382476image/svg+xmlMatplotlib v3.3.1, https://matplotlib.org/

2020-08-25T01:01:09.511410image/svg+xmlMatplotlib v3.3.1, https://matplotlib.org/

2020-08-25T01:01:09.642336image/svg+xmlMatplotlib v3.3.1, https://matplotlib.org/

2020-08-25T01:01:09.777873image/svg+xmlMatplotlib v3.3.1, https://matplotlib.org/

2020-08-25T01:01:09.918714image/svg+xmlMatplotlib v3.3.1, https://matplotlib.org/

2020-08-25T01:01:10.055918image/svg+xmlMatplotlib v3.3.1, https://matplotlib.org/

2020-08-25T01:01:10.192390image/svg+xmlMatplotlib v3.3.1, https://matplotlib.org/

2020-08-25T01:01:10.329157image/svg+xmlMatplotlib v3.3.1, https://matplotlib.org/

2020-08-25T01:01:10.466374image/svg+xmlMatplotlib v3.3.1, https://matplotlib.org/

2020-08-25T01:01:10.600022image/svg+xmlMatplotlib v3.3.1, https://matplotlib.org/

2020-08-25T01:01:10.732262image/svg+xmlMatplotlib v3.3.1, https://matplotlib.org/

2020-08-25T01:01:10.862091image/svg+xmlMatplotlib v3.3.1, https://matplotlib.org/

2020-08-25T01:01:10.992454image/svg+xmlMatplotlib v3.3.1, https://matplotlib.org/

2020-08-25T01:01:11.126210image/svg+xmlMatplotlib v3.3.1, https://matplotlib.org/

2020-08-25T01:01:11.259935image/svg+xmlMatplotlib v3.3.1, https://matplotlib.org/

2020-08-25T01:01:11.389371image/svg+xmlMatplotlib v3.3.1, https://matplotlib.org/

2020-08-25T01:01:11.519666image/svg+xmlMatplotlib v3.3.1, https://matplotlib.org/

2020-08-25T01:01:11.647727image/svg+xmlMatplotlib v3.3.1, https://matplotlib.org/

2020-08-25T01:01:11.781457image/svg+xmlMatplotlib v3.3.1, https://matplotlib.org/

X84 X54 X71 X46 X45 X40 X23 X81 X11 X1 X19 X31 X74 X34 X91 X5 X77 X78 X13

2020-08-25T01:01:12.089475image/svg+xmlMatplotlib v3.3.1, https://matplotlib.org/

2020-08-25T01:01:12.223779image/svg+xmlMatplotlib v3.3.1, https://matplotlib.org/

2020-08-25T01:01:12.360466image/svg+xmlMatplotlib v3.3.1, https://matplotlib.org/

2020-08-25T01:01:12.504802image/svg+xmlMatplotlib v3.3.1, https://matplotlib.org/

2020-08-25T01:01:12.650135image/svg+xmlMatplotlib v3.3.1, https://matplotlib.org/

2020-08-25T01:01:12.792428image/svg+xmlMatplotlib v3.3.1, https://matplotlib.org/

2020-08-25T01:01:12.932212image/svg+xmlMatplotlib v3.3.1, https://matplotlib.org/

2020-08-25T01:01:13.086569image/svg+xmlMatplotlib v3.3.1, https://matplotlib.org/

2020-08-25T01:01:13.220071image/svg+xmlMatplotlib v3.3.1, https://matplotlib.org/

2020-08-25T01:01:13.355078image/svg+xmlMatplotlib v3.3.1, https://matplotlib.org/

2020-08-25T01:01:13.496342image/svg+xmlMatplotlib v3.3.1, https://matplotlib.org/

2020-08-25T01:01:13.631138image/svg+xmlMatplotlib v3.3.1, https://matplotlib.org/

2020-08-25T01:01:13.776306image/svg+xmlMatplotlib v3.3.1, https://matplotlib.org/

2020-08-25T01:01:13.921707image/svg+xmlMatplotlib v3.3.1, https://matplotlib.org/

2020-08-25T01:01:14.066388image/svg+xmlMatplotlib v3.3.1, https://matplotlib.org/

2020-08-25T01:01:14.252840image/svg+xmlMatplotlib v3.3.1, https://matplotlib.org/

2020-08-25T01:01:14.393347image/svg+xmlMatplotlib v3.3.1, https://matplotlib.org/

2020-08-25T01:01:14.532634image/svg+xmlMatplotlib v3.3.1, https://matplotlib.org/

2020-08-25T01:01:14.670153image/svg+xmlMatplotlib v3.3.1, https://matplotlib.org/

X84 X54 X71 X46 X45 X40 X23 X81 X11 X1 X19 X31 X74 X34 X91 X5 X77 X78 X13

2020-08-25T01:01:14.816425image/svg+xmlMatplotlib v3.3.1, https://matplotlib.org/

2020-08-25T01:01:14.952708image/svg+xmlMatplotlib v3.3.1, https://matplotlib.org/

2020-08-25T01:01:15.089899image/svg+xmlMatplotlib v3.3.1, https://matplotlib.org/

2020-08-25T01:01:15.230321image/svg+xmlMatplotlib v3.3.1, https://matplotlib.org/

2020-08-25T01:01:15.368685image/svg+xmlMatplotlib v3.3.1, https://matplotlib.org/

2020-08-25T01:01:15.516227image/svg+xmlMatplotlib v3.3.1, https://matplotlib.org/

2020-08-25T01:01:15.655592image/svg+xmlMatplotlib v3.3.1, https://matplotlib.org/

2020-08-25T01:01:15.798290image/svg+xmlMatplotlib v3.3.1, https://matplotlib.org/

2020-08-25T01:01:15.936650image/svg+xmlMatplotlib v3.3.1, https://matplotlib.org/

2020-08-25T01:01:16.076298image/svg+xmlMatplotlib v3.3.1, https://matplotlib.org/

2020-08-25T01:01:16.212581image/svg+xmlMatplotlib v3.3.1, https://matplotlib.org/

2020-08-25T01:01:16.514628image/svg+xmlMatplotlib v3.3.1, https://matplotlib.org/

2020-08-25T01:01:16.646194image/svg+xmlMatplotlib v3.3.1, https://matplotlib.org/

2020-08-25T01:01:16.789172image/svg+xmlMatplotlib v3.3.1, https://matplotlib.org/

2020-08-25T01:01:16.925781image/svg+xmlMatplotlib v3.3.1, https://matplotlib.org/

2020-08-25T01:01:17.060443image/svg+xmlMatplotlib v3.3.1, https://matplotlib.org/

2020-08-25T01:01:17.195077image/svg+xmlMatplotlib v3.3.1, https://matplotlib.org/

2020-08-25T01:01:17.330681image/svg+xmlMatplotlib v3.3.1, https://matplotlib.org/

2020-08-25T01:01:17.471037image/svg+xmlMatplotlib v3.3.1, https://matplotlib.org/

X84 X54 X71 X46 X45 X40 X23 X81 X11 X1 X19 X31 X74 X34 X91 X5 X77 X78 X13

2020-08-25T01:01:17.612739image/svg+xmlMatplotlib v3.3.1, https://matplotlib.org/

2020-08-25T01:01:17.740680image/svg+xmlMatplotlib v3.3.1, https://matplotlib.org/

2020-08-25T01:01:17.879452image/svg+xmlMatplotlib v3.3.1, https://matplotlib.org/

2020-08-25T01:01:18.020758image/svg+xmlMatplotlib v3.3.1, https://matplotlib.org/

2020-08-25T01:01:18.152832image/svg+xmlMatplotlib v3.3.1, https://matplotlib.org/

2020-08-25T01:01:18.285302image/svg+xmlMatplotlib v3.3.1, https://matplotlib.org/

2020-08-25T01:01:18.416195image/svg+xmlMatplotlib v3.3.1, https://matplotlib.org/

2020-08-25T01:01:18.556826image/svg+xmlMatplotlib v3.3.1, https://matplotlib.org/

2020-08-25T01:01:18.691965image/svg+xmlMatplotlib v3.3.1, https://matplotlib.org/

2020-08-25T01:01:18.833446image/svg+xmlMatplotlib v3.3.1, https://matplotlib.org/

2020-08-25T01:01:18.969170image/svg+xmlMatplotlib v3.3.1, https://matplotlib.org/

2020-08-25T01:01:19.100833image/svg+xmlMatplotlib v3.3.1, https://matplotlib.org/

2020-08-25T01:01:19.234343image/svg+xmlMatplotlib v3.3.1, https://matplotlib.org/

2020-08-25T01:01:19.384924image/svg+xmlMatplotlib v3.3.1, https://matplotlib.org/

2020-08-25T01:01:19.534555image/svg+xmlMatplotlib v3.3.1, https://matplotlib.org/

2020-08-25T01:01:19.667309image/svg+xmlMatplotlib v3.3.1, https://matplotlib.org/

2020-08-25T01:01:19.799248image/svg+xmlMatplotlib v3.3.1, https://matplotlib.org/

2020-08-25T01:01:19.932564image/svg+xmlMatplotlib v3.3.1, https://matplotlib.org/

2020-08-25T01:01:20.070388image/svg+xmlMatplotlib v3.3.1, https://matplotlib.org/

X84 X54 X71 X46 X45 X40 X23 X81 X11 X1 X19 X31 X74 X34 X91 X5 X77 X78 X13

2020-08-25T01:01:20.207033image/svg+xmlMatplotlib v3.3.1, https://matplotlib.org/

2020-08-25T01:01:20.335174image/svg+xmlMatplotlib v3.3.1, https://matplotlib.org/

2020-08-25T01:01:20.463466image/svg+xmlMatplotlib v3.3.1, https://matplotlib.org/

2020-08-25T01:01:20.765363image/svg+xmlMatplotlib v3.3.1, https://matplotlib.org/

2020-08-25T01:01:20.898309image/svg+xmlMatplotlib v3.3.1, https://matplotlib.org/

2020-08-25T01:01:21.032755image/svg+xmlMatplotlib v3.3.1, https://matplotlib.org/

2020-08-25T01:01:21.166998image/svg+xmlMatplotlib v3.3.1, https://matplotlib.org/

2020-08-25T01:01:21.304475image/svg+xmlMatplotlib v3.3.1, https://matplotlib.org/

2020-08-25T01:01:21.434134image/svg+xmlMatplotlib v3.3.1, https://matplotlib.org/

2020-08-25T01:01:21.568583image/svg+xmlMatplotlib v3.3.1, https://matplotlib.org/

2020-08-25T01:01:21.700773image/svg+xmlMatplotlib v3.3.1, https://matplotlib.org/

2020-08-25T01:01:21.829636image/svg+xmlMatplotlib v3.3.1, https://matplotlib.org/

2020-08-25T01:01:21.959783image/svg+xmlMatplotlib v3.3.1, https://matplotlib.org/

2020-08-25T01:01:22.099952image/svg+xmlMatplotlib v3.3.1, https://matplotlib.org/

2020-08-25T01:01:22.233225image/svg+xmlMatplotlib v3.3.1, https://matplotlib.org/

2020-08-25T01:01:22.363817image/svg+xmlMatplotlib v3.3.1, https://matplotlib.org/

2020-08-25T01:01:22.493983image/svg+xmlMatplotlib v3.3.1, https://matplotlib.org/

2020-08-25T01:01:22.626278image/svg+xmlMatplotlib v3.3.1, https://matplotlib.org/

2020-08-25T01:01:22.760093image/svg+xmlMatplotlib v3.3.1, https://matplotlib.org/

X84 X54 X71 X46 X45 X40 X23 X81 X11 X1 X19 X31 X74 X34 X91 X5 X77 X78 X13

2020-08-25T01:01:22.899078image/svg+xmlMatplotlib v3.3.1, https://matplotlib.org/

2020-08-25T01:01:23.032537image/svg+xmlMatplotlib v3.3.1, https://matplotlib.org/

2020-08-25T01:01:23.174678image/svg+xmlMatplotlib v3.3.1, https://matplotlib.org/

2020-08-25T01:01:23.312586image/svg+xmlMatplotlib v3.3.1, https://matplotlib.org/

2020-08-25T01:01:23.452559image/svg+xmlMatplotlib v3.3.1, https://matplotlib.org/

2020-08-25T01:01:23.596755image/svg+xmlMatplotlib v3.3.1, https://matplotlib.org/

2020-08-25T01:01:23.733014image/svg+xmlMatplotlib v3.3.1, https://matplotlib.org/

2020-08-25T01:01:23.893782image/svg+xmlMatplotlib v3.3.1, https://matplotlib.org/

2020-08-25T01:01:24.029792image/svg+xmlMatplotlib v3.3.1, https://matplotlib.org/

2020-08-25T01:01:24.174124image/svg+xmlMatplotlib v3.3.1, https://matplotlib.org/

2020-08-25T01:01:24.320736image/svg+xmlMatplotlib v3.3.1, https://matplotlib.org/

2020-08-25T01:01:24.458021image/svg+xmlMatplotlib v3.3.1, https://matplotlib.org/

2020-08-25T01:01:24.625899image/svg+xmlMatplotlib v3.3.1, https://matplotlib.org/

2020-08-25T01:01:24.763336image/svg+xmlMatplotlib v3.3.1, https://matplotlib.org/

2020-08-25T01:01:25.081952image/svg+xmlMatplotlib v3.3.1, https://matplotlib.org/

2020-08-25T01:01:25.217317image/svg+xmlMatplotlib v3.3.1, https://matplotlib.org/

2020-08-25T01:01:25.356014image/svg+xmlMatplotlib v3.3.1, https://matplotlib.org/

2020-08-25T01:01:25.494393image/svg+xmlMatplotlib v3.3.1, https://matplotlib.org/

2020-08-25T01:01:25.637197image/svg+xmlMatplotlib v3.3.1, https://matplotlib.org/

X84 X54 X71 X46 X45 X40 X23 X81 X11 X1 X19 X31 X74 X34 X91 X5 X77 X78 X13

2020-08-25T01:01:25.780552image/svg+xmlMatplotlib v3.3.1, https://matplotlib.org/

2020-08-25T01:01:25.917649image/svg+xmlMatplotlib v3.3.1, https://matplotlib.org/

2020-08-25T01:01:26.058539image/svg+xmlMatplotlib v3.3.1, https://matplotlib.org/

2020-08-25T01:01:26.203097image/svg+xmlMatplotlib v3.3.1, https://matplotlib.org/
