# Supplementary material for: TPOT-NN: augmenting tree-based automated machine learning with neural network estimators
Source: Genet Program Evolvable Mach. Author manuscript; Available in PMC 2025 Aug 6. (PMC12327408; doi:10.1007/s10710-021-09401-z)
Supplement: Supplementary Material 1 [file NIHMS2035489-supplement-Supplementary_Material_1.zip › New folder/ionosphere.html]

ionosphere 

Toggle navigationionosphere

- Overview
- Variables
- Interactions
- Correlations
- Missing values
- Sample
- Duplicate rows

# Overview

- Overview
- Reproduction
- Warnings 18

Dataset statistics

|  |  |
| --- | --- |
| Number of variables | 20 |
| Number of observations | 351 |
| Missing cells | 0 |
| Missing cells (%) | 0.0% |
| Duplicate rows | 1 |
| Duplicate rows (%) | 0.3% |
| Total size in memory | 55.0 KiB |
| Average record size in memory | 160.4 B |

Variable types

|  |  |
| --- | --- |
| NUM | 17 |
| BOOL | 3 |

Reproduction

|  |  |
| --- | --- |
| Analysis started | 2020-08-25 01:26:02.917478 |
| Analysis finished | 2020-08-25 01:26:50.632364 |
| Duration | 47.71 seconds |
| Version | pandas-profiling v2.8.0 |
| Command line | `pandas_profiling --config_file config.yaml [YOUR_FILE.csv]` |
| Download configuration | config.yaml |

Warnings

|  |  |
| --- | --- |
| `1` has constant value "0" | Constant |
| Dataset has 1 (0.3%) duplicate rows | Duplicates |
| `15` has 45 (12.8%) zeros | Zeros |
| `19` has 34 (9.7%) zeros | Zeros |
| `27` has 4 (1.1%) zeros | Zeros |
| `8` has 32 (9.1%) zeros | Zeros |
| `24` has 24 (6.8%) zeros | Zeros |
| `21` has 37 (10.5%) zeros | Zeros |
| `12` has 31 (8.8%) zeros | Zeros |
| `32` has 48 (13.7%) zeros | Zeros |
| `9` has 39 (11.1%) zeros | Zeros |
| `4` has 38 (10.8%) zeros | Zeros |
| `16` has 25 (7.1%) zeros | Zeros |
| `17` has 30 (8.5%) zeros | Zeros |
| `5` has 46 (13.1%) zeros | Zeros |
| `13` has 37 (10.5%) zeros | Zeros |
| `11` has 37 (10.5%) zeros | Zeros |
| `2` has 25 (7.1%) zeros | Zeros |

# Variables

15  
Real number (ℝ)

`ZEROS`

|  |  |
| --- | --- |
| Distinct count | 270 |
| Unique (%) | 76.9% |
| Missing | 0 |
| Missing (%) | 0.0% |
| Infinite | 0 |
| Infinite (%) | 0.0% |

|  |  |
| --- | --- |
| Mean | 0.07113233618233618 |
| Minimum | -1.0 |
| Maximum | 1.0 |
| Zeros | 45 |
| Zeros (%) | 12.8% |
| Memory size | 2.9 KiB |

2020-08-25T01:26:50.676532image/svg+xmlMatplotlib v3.3.1, https://matplotlib.org/

Toggle details

- Statistics
- Histogram(s)
- Common values
- Extreme values

Quantile statistics

|  |  |
| --- | --- |
| Minimum | -1 |
| 5-th percentile | -1 |
| Q1 | -0.081705 |
| median | 0 |
| Q3 | 0.308975 |
| 95-th percentile | 1 |
| Maximum | 1 |
| Range | 2 |
| Interquartile range (IQR) | 0.39068 |

Descriptive statistics

|  |  |
| --- | --- |
| Standard deviation | 0.4583706723 |
| Coefficient of variation (CV) | 6.443914215 |
| Kurtosis | 0.5508428289 |
| Mean | 0.07113233618 |
| Median Absolute Deviation (MAD) | 0.15727 |
| Skewness | -0.09810551086 |
| Sum | 24.96745 |
| Variance | 0.2101036732 |

- Histogram

2020-08-25T01:26:50.781260image/svg+xmlMatplotlib v3.3.1, https://matplotlib.org/ 

**Histogram with fixed size bins** (bins=10)

| Value | Count | Frequency (%) |  |
| --- | --- | --- | --- |
| 0 | 45 | 12.8% |  |
| -1 | 20 | 5.7% |  |
| 1 | 19 | 5.4% |  |
| -0.01942 | 1 | 0.3% |  |
| 0.27322 | 1 | 0.3% |  |
| 0.01151 | 1 | 0.3% |  |
| 0.41743 | 1 | 0.3% |  |
| 0.14638 | 1 | 0.3% |  |
| 0.01858 | 1 | 0.3% |  |
| -0.03352 | 1 | 0.3% |  |
| 0.07027 | 1 | 0.3% |  |
| 0.4836 | 1 | 0.3% |  |
| 0.775 | 1 | 0.3% |  |
| 0.03366 | 1 | 0.3% |  |
| -0.59017 | 1 | 0.3% |  |
| 0.93682 | 1 | 0.3% |  |
| 0.00299 | 1 | 0.3% |  |
| 0.2738 | 1 | 0.3% |  |
| 0.79523 | 1 | 0.3% |  |
| -0.07917 | 1 | 0.3% |  |
| 0.31439 | 1 | 0.3% |  |
| 0.38778 | 1 | 0.3% |  |
| 0.41471 | 1 | 0.3% |  |
| 0.10195 | 1 | 0.3% |  |
| 0.05058 | 1 | 0.3% |  |
| Other values (245) | 245 | 69.8% |  |

- Minimum 5 values
- Maximum 5 values

| Value | Count | Frequency (%) |  |
| --- | --- | --- | --- |
| -1 | 20 | 5.7% |  |
| -0.97515 | 1 | 0.3% |  |
| -0.83519 | 1 | 0.3% |  |
| -0.68065 | 1 | 0.3% |  |
| -0.67708 | 1 | 0.3% |  |
| -0.63668 | 1 | 0.3% |  |
| -0.61436 | 1 | 0.3% |  |
| -0.59017 | 1 | 0.3% |  |
| -0.58899 | 1 | 0.3% |  |
| -0.57535 | 1 | 0.3% |  |

| Value | Count | Frequency (%) |  |
| --- | --- | --- | --- |
| 1 | 19 | 5.4% |  |
| 0.93682 | 1 | 0.3% |  |
| 0.91404 | 1 | 0.3% |  |
| 0.90665 | 1 | 0.3% |  |
| 0.89274 | 1 | 0.3% |  |
| 0.88444 | 1 | 0.3% |  |
| 0.86284 | 1 | 0.3% |  |
| 0.85038 | 1 | 0.3% |  |
| 0.84804 | 1 | 0.3% |  |
| 0.8429 | 1 | 0.3% |  |

19  
Real number (ℝ)

`ZEROS`

|  |  |
| --- | --- |
| Distinct count | 266 |
| Unique (%) | 75.8% |
| Missing | 0 |
| Missing (%) | 0.0% |
| Infinite | 0 |
| Infinite (%) | 0.0% |

|  |  |
| --- | --- |
| Mean | -0.024024700854700848 |
| Minimum | -1.0 |
| Maximum | 1.0 |
| Zeros | 34 |
| Zeros (%) | 9.7% |
| Memory size | 2.9 KiB |

2020-08-25T01:26:50.901067image/svg+xmlMatplotlib v3.3.1, https://matplotlib.org/

Toggle details

- Statistics
- Histogram(s)
- Common values
- Extreme values

Quantile statistics

|  |  |
| --- | --- |
| Minimum | -1 |
| 5-th percentile | -1 |
| Q1 | -0.23467 |
| median | 0 |
| Q3 | 0.13437 |
| 95-th percentile | 1 |
| Maximum | 1 |
| Range | 2 |
| Interquartile range (IQR) | 0.36904 |

Descriptive statistics

|  |  |
| --- | --- |
| Standard deviation | 0.5190760918 |
| Coefficient of variation (CV) | -21.60593362 |
| Kurtosis | -0.1031621599 |
| Mean | -0.02402470085 |
| Median Absolute Deviation (MAD) | 0.19444 |
| Skewness | 0.05960111262 |
| Sum | -8.43267 |
| Variance | 0.2694399891 |

- Histogram

2020-08-25T01:26:51.006655image/svg+xmlMatplotlib v3.3.1, https://matplotlib.org/ 

**Histogram with fixed size bins** (bins=10)

| Value | Count | Frequency (%) |  |
| --- | --- | --- | --- |
| 0 | 34 | 9.7% |  |
| -1 | 28 | 8.0% |  |
| 1 | 24 | 6.8% |  |
| 0.12727 | 2 | 0.6% |  |
| 0.1 | 2 | 0.6% |  |
| 0.03617 | 1 | 0.3% |  |
| -0.32192 | 1 | 0.3% |  |
| -0.00991 | 1 | 0.3% |  |
| 0.55102 | 1 | 0.3% |  |
| 0.2659 | 1 | 0.3% |  |
| -0.13652 | 1 | 0.3% |  |
| 0.03566 | 1 | 0.3% |  |
| -0.01266 | 1 | 0.3% |  |
| 0.78036 | 1 | 0.3% |  |
| 0.05279 | 1 | 0.3% |  |
| -0.88952 | 1 | 0.3% |  |
| 0.01408 | 1 | 0.3% |  |
| 0.65747 | 1 | 0.3% |  |
| -0.32963 | 1 | 0.3% |  |
| 0.5307 | 1 | 0.3% |  |
| 0.01386 | 1 | 0.3% |  |
| 0.06618 | 1 | 0.3% |  |
| -0.73641 | 1 | 0.3% |  |
| 0.75554 | 1 | 0.3% |  |
| 0.05263 | 1 | 0.3% |  |
| Other values (241) | 241 | 68.7% |  |

- Minimum 5 values
- Maximum 5 values

| Value | Count | Frequency (%) |  |
| --- | --- | --- | --- |
| -1 | 28 | 8.0% |  |
| -0.93599 | 1 | 0.3% |  |
| -0.88952 | 1 | 0.3% |  |
| -0.87787 | 1 | 0.3% |  |
| -0.86275 | 1 | 0.3% |  |
| -0.86063 | 1 | 0.3% |  |
| -0.86002 | 1 | 0.3% |  |
| -0.85654 | 1 | 0.3% |  |
| -0.85583 | 1 | 0.3% |  |
| -0.84848 | 1 | 0.3% |  |

| Value | Count | Frequency (%) |  |
| --- | --- | --- | --- |
| 1 | 24 | 6.8% |  |
| 0.95099 | 1 | 0.3% |  |
| 0.93109 | 1 | 0.3% |  |
| 0.92949 | 1 | 0.3% |  |
| 0.85952 | 1 | 0.3% |  |
| 0.84922 | 1 | 0.3% |  |
| 0.79777 | 1 | 0.3% |  |
| 0.79749 | 1 | 0.3% |  |
| 0.79617 | 1 | 0.3% |  |
| 0.79525 | 1 | 0.3% |  |

27  
Real number (ℝ)

`ZEROS`

|  |  |
| --- | --- |
| Distinct count | 281 |
| Unique (%) | 80.1% |
| Missing | 0 |
| Missing (%) | 0.0% |
| Infinite | 0 |
| Infinite (%) | 0.0% |

|  |  |
| --- | --- |
| Mean | -0.06953760683760683 |
| Minimum | -1.0 |
| Maximum | 1.0 |
| Zeros | 4 |
| Zeros (%) | 1.1% |
| Memory size | 2.9 KiB |

2020-08-25T01:26:51.124417image/svg+xmlMatplotlib v3.3.1, https://matplotlib.org/

Toggle details

- Statistics
- Histogram(s)
- Common values
- Extreme values

Quantile statistics

|  |  |
| --- | --- |
| Minimum | -1 |
| 5-th percentile | -1 |
| Q1 | -0.443165 |
| median | -0.01769 |
| Q3 | 0.153535 |
| 95-th percentile | 1 |
| Maximum | 1 |
| Range | 2 |
| Interquartile range (IQR) | 0.5967 |

Descriptive statistics

|  |  |
| --- | --- |
| Standard deviation | 0.5500252428 |
| Coefficient of variation (CV) | -7.909752259 |
| Kurtosis | -0.3431892824 |
| Mean | -0.06953760684 |
| Median Absolute Deviation (MAD) | 0.23985 |
| Skewness | 0.0667074171 |
| Sum | -24.4077 |
| Variance | 0.3025277677 |

- Histogram

2020-08-25T01:26:51.232310image/svg+xmlMatplotlib v3.3.1, https://matplotlib.org/ 

**Histogram with fixed size bins** (bins=10)

| Value | Count | Frequency (%) |  |
| --- | --- | --- | --- |
| -1 | 40 | 11.4% |  |
| 1 | 29 | 8.3% |  |
| 0 | 4 | 1.1% |  |
| -0.00838 | 1 | 0.3% |  |
| -0.16837 | 1 | 0.3% |  |
| 0.05503 | 1 | 0.3% |  |
| -0.81372 | 1 | 0.3% |  |
| -0.04822 | 1 | 0.3% |  |
| -0.71504 | 1 | 0.3% |  |
| -0.53206 | 1 | 0.3% |  |
| -0.01769 | 1 | 0.3% |  |
| -0.00128 | 1 | 0.3% |  |
| 0.01096 | 1 | 0.3% |  |
| 0.13282 | 1 | 0.3% |  |
| -0.61959 | 1 | 0.3% |  |
| 0.02226 | 1 | 0.3% |  |
| 0.20225 | 1 | 0.3% |  |
| -0.01358 | 1 | 0.3% |  |
| 0.66335 | 1 | 0.3% |  |
| -0.23864 | 1 | 0.3% |  |
| 0.02019 | 1 | 0.3% |  |
| -0.10897 | 1 | 0.3% |  |
| 0.69129 | 1 | 0.3% |  |
| -0.76667 | 1 | 0.3% |  |
| 0.08165 | 1 | 0.3% |  |
| Other values (256) | 256 | 72.9% |  |

- Minimum 5 values
- Maximum 5 values

| Value | Count | Frequency (%) |  |
| --- | --- | --- | --- |
| -1 | 40 | 11.4% |  |
| -0.93939 | 1 | 0.3% |  |
| -0.93296 | 1 | 0.3% |  |
| -0.91221 | 1 | 0.3% |  |
| -0.86583 | 1 | 0.3% |  |
| -0.865 | 1 | 0.3% |  |
| -0.86122 | 1 | 0.3% |  |
| -0.85669 | 1 | 0.3% |  |
| -0.85137 | 1 | 0.3% |  |
| -0.8251 | 1 | 0.3% |  |

| Value | Count | Frequency (%) |  |
| --- | --- | --- | --- |
| 1 | 29 | 8.3% |  |
| 0.88248 | 1 | 0.3% |  |
| 0.83796 | 1 | 0.3% |  |
| 0.78541 | 1 | 0.3% |  |
| 0.78004 | 1 | 0.3% |  |
| 0.75 | 1 | 0.3% |  |
| 0.73885 | 1 | 0.3% |  |
| 0.72386 | 1 | 0.3% |  |
| 0.71613 | 1 | 0.3% |  |
| 0.71374 | 1 | 0.3% |  |

26  
Real number (ℝ)

|  |  |
| --- | --- |
| Distinct count | 256 |
| Unique (%) | 72.9% |
| Missing | 0 |
| Missing (%) | 0.0% |
| Infinite | 0 |
| Infinite (%) | 0.0% |

|  |  |
| --- | --- |
| Mean | 0.5416407977207977 |
| Minimum | -1.0 |
| Maximum | 1.0 |
| Zeros | 0 |
| Zeros (%) | 0.0% |
| Memory size | 2.9 KiB |

2020-08-25T01:26:51.354892image/svg+xmlMatplotlib v3.3.1, https://matplotlib.org/

Toggle details

- Statistics
- Histogram(s)
- Common values
- Extreme values

Quantile statistics

|  |  |
| --- | --- |
| Minimum | -1 |
| 5-th percentile | -0.64201 |
| Q1 | 0.286435 |
| median | 0.70824 |
| Q3 | 0.999945 |
| 95-th percentile | 1 |
| Maximum | 1 |
| Range | 2 |
| Interquartile range (IQR) | 0.71351 |

Descriptive statistics

|  |  |
| --- | --- |
| Standard deviation | 0.5162046654 |
| Coefficient of variation (CV) | 0.9530387437 |
| Kurtosis | 1.021871153 |
| Mean | 0.5416407977 |
| Median Absolute Deviation (MAD) | 0.29176 |
| Skewness | -1.287052977 |
| Sum | 190.11592 |
| Variance | 0.2664672566 |

- Histogram

2020-08-25T01:26:51.470541image/svg+xmlMatplotlib v3.3.1, https://matplotlib.org/ 

**Histogram with fixed size bins** (bins=10)

| Value | Count | Frequency (%) |  |
| --- | --- | --- | --- |
| 1 | 88 | 25.1% |  |
| -1 | 9 | 2.6% |  |
| 0.70824 | 1 | 0.3% |  |
| 0.61847 | 1 | 0.3% |  |
| -0.22839 | 1 | 0.3% |  |
| -1e-05 | 1 | 0.3% |  |
| 0.25758 | 1 | 0.3% |  |
| 0.68656 | 1 | 0.3% |  |
| 0.16813 | 1 | 0.3% |  |
| 0.29208 | 1 | 0.3% |  |
| 0.67538 | 1 | 0.3% |  |
| 0.76717 | 1 | 0.3% |  |
| -0.1994 | 1 | 0.3% |  |
| -0.19149 | 1 | 0.3% |  |
| -0.63237 | 1 | 0.3% |  |
| 0.57577 | 1 | 0.3% |  |
| -0.16556 | 1 | 0.3% |  |
| 0.46514 | 1 | 0.3% |  |
| 0.56804 | 1 | 0.3% |  |
| 0.83796 | 1 | 0.3% |  |
| 0.65419 | 1 | 0.3% |  |
| -0.70352 | 1 | 0.3% |  |
| 0.92148 | 1 | 0.3% |  |
| -0.0153 | 1 | 0.3% |  |
| 0.39752 | 1 | 0.3% |  |
| Other values (231) | 231 | 65.8% |  |

- Minimum 5 values
- Maximum 5 values

| Value | Count | Frequency (%) |  |
| --- | --- | --- | --- |
| -1 | 9 | 2.6% |  |
| -0.83314 | 1 | 0.3% |  |
| -0.77097 | 1 | 0.3% |  |
| -0.71844 | 1 | 0.3% |  |
| -0.71731 | 1 | 0.3% |  |
| -0.70352 | 1 | 0.3% |  |
| -0.67925 | 1 | 0.3% |  |
| -0.67557 | 1 | 0.3% |  |
| -0.66651 | 1 | 0.3% |  |
| -0.65165 | 1 | 0.3% |  |

| Value | Count | Frequency (%) |  |
| --- | --- | --- | --- |
| 1 | 88 | 25.1% |  |
| 0.99989 | 1 | 0.3% |  |
| 0.99842 | 1 | 0.3% |  |
| 0.99188 | 1 | 0.3% |  |
| 0.9882 | 1 | 0.3% |  |
| 0.98556 | 1 | 0.3% |  |
| 0.98033 | 1 | 0.3% |  |
| 0.9754 | 1 | 0.3% |  |
| 0.96974 | 1 | 0.3% |  |
| 0.96709 | 1 | 0.3% |  |

8  
Real number (ℝ)

`ZEROS`

|  |  |
| --- | --- |
| Distinct count | 244 |
| Unique (%) | 69.5% |
| Missing | 0 |
| Missing (%) | 0.0% |
| Infinite | 0 |
| Infinite (%) | 0.0% |

|  |  |
| --- | --- |
| Mean | 0.5118480911680912 |
| Minimum | -1.0 |
| Maximum | 1.0 |
| Zeros | 32 |
| Zeros (%) | 9.1% |
| Memory size | 2.9 KiB |

2020-08-25T01:26:51.592391image/svg+xmlMatplotlib v3.3.1, https://matplotlib.org/

Toggle details

- Statistics
- Histogram(s)
- Common values
- Extreme values

Quantile statistics

|  |  |
| --- | --- |
| Minimum | -1 |
| 5-th percentile | -0.355485 |
| Q1 | 0.08711 |
| median | 0.68421 |
| Q3 | 0.95324 |
| 95-th percentile | 1 |
| Maximum | 1 |
| Range | 2 |
| Interquartile range (IQR) | 0.86613 |

Descriptive statistics

|  |  |
| --- | --- |
| Standard deviation | 0.5070655269 |
| Coefficient of variation (CV) | 0.9906562818 |
| Kurtosis | 0.6873606011 |
| Mean | 0.5118480912 |
| Median Absolute Deviation (MAD) | 0.31579 |
| Skewness | -1.091865491 |
| Sum | 179.65868 |
| Variance | 0.2571154485 |

- Histogram

2020-08-25T01:26:51.698834image/svg+xmlMatplotlib v3.3.1, https://matplotlib.org/ 

**Histogram with fixed size bins** (bins=10)

| Value | Count | Frequency (%) |  |
| --- | --- | --- | --- |
| 1 | 64 | 18.2% |  |
| 0 | 32 | 9.1% |  |
| -1 | 11 | 3.1% |  |
| 0.63636 | 2 | 0.6% |  |
| -0.33333 | 2 | 0.6% |  |
| 0.8 | 2 | 0.6% |  |
| 0.55717 | 1 | 0.3% |  |
| 0.2381 | 1 | 0.3% |  |
| -0.42625 | 1 | 0.3% |  |
| 0.6932 | 1 | 0.3% |  |
| -0.03643 | 1 | 0.3% |  |
| 0.92765 | 1 | 0.3% |  |
| 0.74273 | 1 | 0.3% |  |
| 0.71157 | 1 | 0.3% |  |
| -0.37764 | 1 | 0.3% |  |
| -0.85 | 1 | 0.3% |  |
| 0.9 | 1 | 0.3% |  |
| 0.98602 | 1 | 0.3% |  |
| 0.95878 | 1 | 0.3% |  |
| -0.2697 | 1 | 0.3% |  |
| 0.07405 | 1 | 0.3% |  |
| 0.30508 | 1 | 0.3% |  |
| 0.73673 | 1 | 0.3% |  |
| 0.08776 | 1 | 0.3% |  |
| 0.7176 | 1 | 0.3% |  |
| Other values (219) | 219 | 62.4% |  |

- Minimum 5 values
- Maximum 5 values

| Value | Count | Frequency (%) |  |
| --- | --- | --- | --- |
| -1 | 11 | 3.1% |  |
| -0.87097 | 1 | 0.3% |  |
| -0.85 | 1 | 0.3% |  |
| -0.55941 | 1 | 0.3% |  |
| -0.4902 | 1 | 0.3% |  |
| -0.42625 | 1 | 0.3% |  |
| -0.38033 | 1 | 0.3% |  |
| -0.37764 | 1 | 0.3% |  |
| -0.33333 | 2 | 0.6% |  |
| -0.33221 | 1 | 0.3% |  |

| Value | Count | Frequency (%) |  |
| --- | --- | --- | --- |
| 1 | 64 | 18.2% |  |
| 0.99838 | 1 | 0.3% |  |
| 0.99709 | 1 | 0.3% |  |
| 0.99448 | 1 | 0.3% |  |
| 0.99374 | 1 | 0.3% |  |
| 0.9929 | 1 | 0.3% |  |
| 0.99061 | 1 | 0.3% |  |
| 0.9872 | 1 | 0.3% |  |
| 0.9863 | 1 | 0.3% |  |
| 0.98602 | 1 | 0.3% |  |

24  
Real number (ℝ)

`ZEROS`

|  |  |
| --- | --- |
| Distinct count | 256 |
| Unique (%) | 72.9% |
| Missing | 0 |
| Missing (%) | 0.0% |
| Infinite | 0 |
| Infinite (%) | 0.0% |

|  |  |
| --- | --- |
| Mean | 0.39613467236467237 |
| Minimum | -1.0 |
| Maximum | 1.0 |
| Zeros | 24 |
| Zeros (%) | 6.8% |
| Memory size | 2.9 KiB |

2020-08-25T01:26:51.815454image/svg+xmlMatplotlib v3.3.1, https://matplotlib.org/

Toggle details

- Statistics
- Histogram(s)
- Common values
- Extreme values

Quantile statistics

|  |  |
| --- | --- |
| Minimum | -1 |
| 5-th percentile | -0.87839 |
| Q1 | 0 |
| median | 0.55389 |
| Q3 | 0.90524 |
| 95-th percentile | 1 |
| Maximum | 1 |
| Range | 2 |
| Interquartile range (IQR) | 0.90524 |

Descriptive statistics

|  |  |
| --- | --- |
| Standard deviation | 0.5784508875 |
| Coefficient of variation (CV) | 1.460237964 |
| Kurtosis | -0.1370876483 |
| Mean | 0.3961346724 |
| Median Absolute Deviation (MAD) | 0.41963 |
| Skewness | -0.8815884425 |
| Sum | 139.04327 |
| Variance | 0.3346054293 |

- Histogram

2020-08-25T01:26:51.923211image/svg+xmlMatplotlib v3.3.1, https://matplotlib.org/ 

**Histogram with fixed size bins** (bins=10)

| Value | Count | Frequency (%) |  |
| --- | --- | --- | --- |
| 1 | 57 | 16.2% |  |
| 0 | 24 | 6.8% |  |
| -1 | 15 | 4.3% |  |
| 0.66667 | 2 | 0.6% |  |
| 0.5 | 2 | 0.6% |  |
| 0.87234 | 1 | 0.3% |  |
| 0.67077 | 1 | 0.3% |  |
| 0.69274 | 1 | 0.3% |  |
| 0.77336 | 1 | 0.3% |  |
| 0.84926 | 1 | 0.3% |  |
| 0.48526 | 1 | 0.3% |  |
| 0.84547 | 1 | 0.3% |  |
| 0.92049 | 1 | 0.3% |  |
| 0.88487 | 1 | 0.3% |  |
| -0.04119 | 1 | 0.3% |  |
| -0.08748 | 1 | 0.3% |  |
| 0.73413 | 1 | 0.3% |  |
| 0.47929 | 1 | 0.3% |  |
| 0.85106 | 1 | 0.3% |  |
| 0.77138 | 1 | 0.3% |  |
| 0.5375 | 1 | 0.3% |  |
| 0.70619 | 1 | 0.3% |  |
| 0.00838 | 1 | 0.3% |  |
| 0.56811 | 1 | 0.3% |  |
| -0.02862 | 1 | 0.3% |  |
| Other values (231) | 231 | 65.8% |  |

- Minimum 5 values
- Maximum 5 values

| Value | Count | Frequency (%) |  |
| --- | --- | --- | --- |
| -1 | 15 | 4.3% |  |
| -0.98988 | 1 | 0.3% |  |
| -0.91574 | 1 | 0.3% |  |
| -0.90302 | 1 | 0.3% |  |
| -0.85376 | 1 | 0.3% |  |
| -0.84792 | 1 | 0.3% |  |
| -0.83192 | 1 | 0.3% |  |
| -0.78334 | 1 | 0.3% |  |
| -0.75273 | 1 | 0.3% |  |
| -0.74265 | 1 | 0.3% |  |

| Value | Count | Frequency (%) |  |
| --- | --- | --- | --- |
| 1 | 57 | 16.2% |  |
| 0.99899 | 1 | 0.3% |  |
| 0.99695 | 1 | 0.3% |  |
| 0.98971 | 1 | 0.3% |  |
| 0.9875 | 1 | 0.3% |  |
| 0.98401 | 1 | 0.3% |  |
| 0.98164 | 1 | 0.3% |  |
| 0.97838 | 1 | 0.3% |  |
| 0.97352 | 1 | 0.3% |  |
| 0.97127 | 1 | 0.3% |  |

21  
Real number (ℝ)

`ZEROS`

|  |  |
| --- | --- |
| Distinct count | 265 |
| Unique (%) | 75.5% |
| Missing | 0 |
| Missing (%) | 0.0% |
| Infinite | 0 |
| Infinite (%) | 0.0% |

|  |  |
| --- | --- |
| Mean | 0.008295897435897433 |
| Minimum | -1.0 |
| Maximum | 1.0 |
| Zeros | 37 |
| Zeros (%) | 10.5% |
| Memory size | 2.9 KiB |

2020-08-25T01:26:52.044328image/svg+xmlMatplotlib v3.3.1, https://matplotlib.org/

Toggle details

- Statistics
- Histogram(s)
- Common values
- Extreme values

Quantile statistics

|  |  |
| --- | --- |
| Minimum | -1 |
| 5-th percentile | -1 |
| Q1 | -0.24387 |
| median | 0 |
| Q3 | 0.18876 |
| 95-th percentile | 1 |
| Maximum | 1 |
| Range | 2 |
| Interquartile range (IQR) | 0.43263 |

Descriptive statistics

|  |  |
| --- | --- |
| Standard deviation | 0.5181658868 |
| Coefficient of variation (CV) | 62.4604982 |
| Kurtosis | -0.128649046 |
| Mean | 0.008295897436 |
| Median Absolute Deviation (MAD) | 0.21326 |
| Skewness | 0.06680524004 |
| Sum | 2.91186 |
| Variance | 0.2684958863 |

- Histogram

2020-08-25T01:26:52.149675image/svg+xmlMatplotlib v3.3.1, https://matplotlib.org/ 

**Histogram with fixed size bins** (bins=10)

| Value | Count | Frequency (%) |  |
| --- | --- | --- | --- |
| 0 | 37 | 10.5% |  |
| 1 | 30 | 8.5% |  |
| -1 | 21 | 6.0% |  |
| 0.00075 | 2 | 0.6% |  |
| -0.03333 | 1 | 0.3% |  |
| -0.08039 | 1 | 0.3% |  |
| -0.55605 | 1 | 0.3% |  |
| -0.76316 | 1 | 0.3% |  |
| -0.04971 | 1 | 0.3% |  |
| -0.11953 | 1 | 0.3% |  |
| -0.3 | 1 | 0.3% |  |
| 0.5081 | 1 | 0.3% |  |
| -0.82217 | 1 | 0.3% |  |
| -0.00377 | 1 | 0.3% |  |
| -0.66278 | 1 | 0.3% |  |
| 0.14258 | 1 | 0.3% |  |
| -0.00299 | 1 | 0.3% |  |
| -0.11526 | 1 | 0.3% |  |
| -0.01891 | 1 | 0.3% |  |
| 0.14157 | 1 | 0.3% |  |
| -0.03377 | 1 | 0.3% |  |
| -0.23774 | 1 | 0.3% |  |
| 0.12195 | 1 | 0.3% |  |
| 0.30508 | 1 | 0.3% |  |
| 0.07677 | 1 | 0.3% |  |
| Other values (240) | 240 | 68.4% |  |

- Minimum 5 values
- Maximum 5 values

| Value | Count | Frequency (%) |  |
| --- | --- | --- | --- |
| -1 | 21 | 6.0% |  |
| -0.93596 | 1 | 0.3% |  |
| -0.92536 | 1 | 0.3% |  |
| -0.89991 | 1 | 0.3% |  |
| -0.89064 | 1 | 0.3% |  |
| -0.88999 | 1 | 0.3% |  |
| -0.86498 | 1 | 0.3% |  |
| -0.85597 | 1 | 0.3% |  |
| -0.85205 | 1 | 0.3% |  |
| -0.83007 | 1 | 0.3% |  |

| Value | Count | Frequency (%) |  |
| --- | --- | --- | --- |
| 1 | 30 | 8.5% |  |
| 0.9273 | 1 | 0.3% |  |
| 0.92001 | 1 | 0.3% |  |
| 0.89383 | 1 | 0.3% |  |
| 0.85615 | 1 | 0.3% |  |
| 0.85268 | 1 | 0.3% |  |
| 0.82162 | 1 | 0.3% |  |
| 0.80754 | 1 | 0.3% |  |
| 0.76969 | 1 | 0.3% |  |
| 0.76193 | 1 | 0.3% |  |

12  
Real number (ℝ)

`ZEROS`

|  |  |
| --- | --- |
| Distinct count | 238 |
| Unique (%) | 67.8% |
| Missing | 0 |
| Missing (%) | 0.0% |
| Infinite | 0 |
| Infinite (%) | 0.0% |

|  |  |
| --- | --- |
| Mean | 0.40080119658119656 |
| Minimum | -1.0 |
| Maximum | 1.0 |
| Zeros | 31 |
| Zeros (%) | 8.8% |
| Memory size | 2.9 KiB |

2020-08-25T01:26:52.268061image/svg+xmlMatplotlib v3.3.1, https://matplotlib.org/

Toggle details

- Statistics
- Histogram(s)
- Common values
- Extreme values

Quantile statistics

|  |  |
| --- | --- |
| Minimum | -1 |
| 5-th percentile | -0.93487 |
| Q1 | 0 |
| median | 0.64407 |
| Q3 | 0.955505 |
| 95-th percentile | 1 |
| Maximum | 1 |
| Range | 2 |
| Interquartile range (IQR) | 0.955505 |

Descriptive statistics

|  |  |
| --- | --- |
| Standard deviation | 0.622186124 |
| Coefficient of variation (CV) | 1.552355954 |
| Kurtosis | -0.4039937329 |
| Mean | 0.4008011966 |
| Median Absolute Deviation (MAD) | 0.35593 |
| Skewness | -0.8762252925 |
| Sum | 140.68122 |
| Variance | 0.387115573 |

- Histogram

2020-08-25T01:26:52.375664image/svg+xmlMatplotlib v3.3.1, https://matplotlib.org/ 

**Histogram with fixed size bins** (bins=10)

| Value | Count | Frequency (%) |  |
| --- | --- | --- | --- |
| 1 | 65 | 18.5% |  |
| 0 | 31 | 8.8% |  |
| -1 | 17 | 4.8% |  |
| 0.47368 | 2 | 0.6% |  |
| 0.16667 | 2 | 0.6% |  |
| 0.62099 | 2 | 0.6% |  |
| 0.85269 | 1 | 0.3% |  |
| 0.77937 | 1 | 0.3% |  |
| 0.59753 | 1 | 0.3% |  |
| 0.0925 | 1 | 0.3% |  |
| -0.61352 | 1 | 0.3% |  |
| -0.03736 | 1 | 0.3% |  |
| 0.6905 | 1 | 0.3% |  |
| -0.76051 | 1 | 0.3% |  |
| -0.03158 | 1 | 0.3% |  |
| 0.71685 | 1 | 0.3% |  |
| 0.76221 | 1 | 0.3% |  |
| 0.28931 | 1 | 0.3% |  |
| 0.97888 | 1 | 0.3% |  |
| 0.44675 | 1 | 0.3% |  |
| 0.12766 | 1 | 0.3% |  |
| 0.6726 | 1 | 0.3% |  |
| 0.64725 | 1 | 0.3% |  |
| 0.99676 | 1 | 0.3% |  |
| 0.95824 | 1 | 0.3% |  |
| Other values (213) | 213 | 60.7% |  |

- Minimum 5 values
- Maximum 5 values

| Value | Count | Frequency (%) |  |
| --- | --- | --- | --- |
| -1 | 17 | 4.8% |  |
| -0.93882 | 1 | 0.3% |  |
| -0.93092 | 1 | 0.3% |  |
| -0.92099 | 1 | 0.3% |  |
| -0.87192 | 1 | 0.3% |  |
| -0.86622 | 1 | 0.3% |  |
| -0.86443 | 1 | 0.3% |  |
| -0.84211 | 1 | 0.3% |  |
| -0.81857 | 1 | 0.3% |  |
| -0.81699 | 1 | 0.3% |  |

| Value | Count | Frequency (%) |  |
| --- | --- | --- | --- |
| 1 | 65 | 18.5% |  |
| 0.99745 | 1 | 0.3% |  |
| 0.99676 | 1 | 0.3% |  |
| 0.99173 | 1 | 0.3% |  |
| 0.98919 | 1 | 0.3% |  |
| 0.985 | 1 | 0.3% |  |
| 0.98343 | 1 | 0.3% |  |
| 0.9812 | 1 | 0.3% |  |
| 0.9798 | 1 | 0.3% |  |
| 0.97921 | 1 | 0.3% |  |

32  
Real number (ℝ)

`ZEROS`

|  |  |
| --- | --- |
| Distinct count | 245 |
| Unique (%) | 69.8% |
| Missing | 0 |
| Missing (%) | 0.0% |
| Infinite | 0 |
| Infinite (%) | 0.0% |

|  |  |
| --- | --- |
| Mean | 0.34936364672364667 |
| Minimum | -1.0 |
| Maximum | 1.0 |
| Zeros | 48 |
| Zeros (%) | 13.7% |
| Memory size | 2.9 KiB |

2020-08-25T01:26:52.668230image/svg+xmlMatplotlib v3.3.1, https://matplotlib.org/

Toggle details

- Statistics
- Histogram(s)
- Common values
- Extreme values

Quantile statistics

|  |  |
| --- | --- |
| Minimum | -1 |
| 5-th percentile | -0.65533 |
| Q1 | 0 |
| median | 0.40956 |
| Q3 | 0.813765 |
| 95-th percentile | 1 |
| Maximum | 1 |
| Range | 2 |
| Interquartile range (IQR) | 0.813765 |

Descriptive statistics

|  |  |
| --- | --- |
| Standard deviation | 0.5226633728 |
| Coefficient of variation (CV) | 1.496043958 |
| Kurtosis | -0.1689502847 |
| Mean | 0.3493636467 |
| Median Absolute Deviation (MAD) | 0.40956 |
| Skewness | -0.6060703823 |
| Sum | 122.62664 |
| Variance | 0.2731770013 |

- Histogram

2020-08-25T01:26:52.769064image/svg+xmlMatplotlib v3.3.1, https://matplotlib.org/ 

**Histogram with fixed size bins** (bins=10)

| Value | Count | Frequency (%) |  |
| --- | --- | --- | --- |
| 0 | 48 | 13.7% |  |
| 1 | 48 | 13.7% |  |
| -1 | 12 | 3.4% |  |
| -8e-05 | 2 | 0.6% |  |
| -0.07457 | 1 | 0.3% |  |
| 0.96523 | 1 | 0.3% |  |
| -0.13832 | 1 | 0.3% |  |
| 0.30421 | 1 | 0.3% |  |
| 0.30445 | 1 | 0.3% |  |
| -0.08937 | 1 | 0.3% |  |
| 0.92049 | 1 | 0.3% |  |
| 0.39559 | 1 | 0.3% |  |
| 0.42189 | 1 | 0.3% |  |
| -0.91903 | 1 | 0.3% |  |
| 0.40591 | 1 | 0.3% |  |
| 0.14694 | 1 | 0.3% |  |
| 0.73208 | 1 | 0.3% |  |
| 0.0699 | 1 | 0.3% |  |
| 0.50169 | 1 | 0.3% |  |
| 0.70833 | 1 | 0.3% |  |
| 0.75115 | 1 | 0.3% |  |
| 0.60073 | 1 | 0.3% |  |
| 0.33381 | 1 | 0.3% |  |
| 0.18641 | 1 | 0.3% |  |
| -0.67699 | 1 | 0.3% |  |
| Other values (220) | 220 | 62.7% |  |

- Minimum 5 values
- Maximum 5 values

| Value | Count | Frequency (%) |  |
| --- | --- | --- | --- |
| -1 | 12 | 3.4% |  |
| -0.91903 | 1 | 0.3% |  |
| -0.81383 | 1 | 0.3% |  |
| -0.8121 | 1 | 0.3% |  |
| -0.67699 | 1 | 0.3% |  |
| -0.67553 | 1 | 0.3% |  |
| -0.66932 | 1 | 0.3% |  |
| -0.64134 | 1 | 0.3% |  |
| -0.64056 | 1 | 0.3% |  |
| -0.59943 | 1 | 0.3% |  |

| Value | Count | Frequency (%) |  |
| --- | --- | --- | --- |
| 1 | 48 | 13.7% |  |
| 0.98971 | 1 | 0.3% |  |
| 0.98934 | 1 | 0.3% |  |
| 0.98816 | 1 | 0.3% |  |
| 0.98674 | 1 | 0.3% |  |
| 0.9842 | 1 | 0.3% |  |
| 0.97861 | 1 | 0.3% |  |
| 0.97561 | 1 | 0.3% |  |
| 0.97247 | 1 | 0.3% |  |
| 0.96778 | 1 | 0.3% |  |

9  
Real number (ℝ)

`ZEROS`

|  |  |
| --- | --- |
| Distinct count | 267 |
| Unique (%) | 76.1% |
| Missing | 0 |
| Missing (%) | 0.0% |
| Infinite | 0 |
| Infinite (%) | 0.0% |

|  |  |
| --- | --- |
| Mean | 0.18134538461538463 |
| Minimum | -1.0 |
| Maximum | 1.0 |
| Zeros | 39 |
| Zeros (%) | 11.1% |
| Memory size | 2.9 KiB |

2020-08-25T01:26:52.883420image/svg+xmlMatplotlib v3.3.1, https://matplotlib.org/

Toggle details

- Statistics
- Histogram(s)
- Common values
- Extreme values

Quantile statistics

|  |  |
| --- | --- |
| Minimum | -1 |
| 5-th percentile | -0.57299 |
| Q1 | -0.048075 |
| median | 0.01829 |
| Q3 | 0.534195 |
| 95-th percentile | 1 |
| Maximum | 1 |
| Range | 2 |
| Interquartile range (IQR) | 0.58227 |

Descriptive statistics

|  |  |
| --- | --- |
| Standard deviation | 0.4838508883 |
| Coefficient of variation (CV) | 2.668118019 |
| Kurtosis | 0.1376096263 |
| Mean | 0.1813453846 |
| Median Absolute Deviation (MAD) | 0.14329 |
| Skewness | -0.02873843003 |
| Sum | 63.65223 |
| Variance | 0.2341116821 |

- Histogram

2020-08-25T01:26:52.993318image/svg+xmlMatplotlib v3.3.1, https://matplotlib.org/ 

**Histogram with fixed size bins** (bins=10)

| Value | Count | Frequency (%) |  |
| --- | --- | --- | --- |
| 0 | 39 | 11.1% |  |
| 1 | 28 | 8.0% |  |
| -1 | 17 | 4.8% |  |
| -0.09091 | 2 | 0.6% |  |
| -0.14286 | 2 | 0.6% |  |
| -0.07514 | 2 | 0.6% |  |
| -0.25843 | 1 | 0.3% |  |
| -0.08337 | 1 | 0.3% |  |
| 0.32899 | 1 | 0.3% |  |
| -0.49962 | 1 | 0.3% |  |
| 0.01064 | 1 | 0.3% |  |
| -0.40446 | 1 | 0.3% |  |
| 0.89109 | 1 | 0.3% |  |
| 0.51042 | 1 | 0.3% |  |
| -0.09925 | 1 | 0.3% |  |
| 0.12961 | 1 | 0.3% |  |
| 0.90842 | 1 | 0.3% |  |
| -0.01622 | 1 | 0.3% |  |
| -0.03911 | 1 | 0.3% |  |
| 0.32711 | 1 | 0.3% |  |
| 0.9074 | 1 | 0.3% |  |
| 0.58304 | 1 | 0.3% |  |
| 0.01172 | 1 | 0.3% |  |
| 0.93182 | 1 | 0.3% |  |
| 0.95776 | 1 | 0.3% |  |
| Other values (242) | 242 | 68.9% |  |

- Minimum 5 values
- Maximum 5 values

| Value | Count | Frequency (%) |  |
| --- | --- | --- | --- |
| -1 | 17 | 4.8% |  |
| -0.63427 | 1 | 0.3% |  |
| -0.51171 | 1 | 0.3% |  |
| -0.5 | 1 | 0.3% |  |
| -0.49962 | 1 | 0.3% |  |
| -0.43107 | 1 | 0.3% |  |
| -0.40446 | 1 | 0.3% |  |
| -0.375 | 1 | 0.3% |  |
| -0.36174 | 1 | 0.3% |  |
| -0.35818 | 1 | 0.3% |  |

| Value | Count | Frequency (%) |  |
| --- | --- | --- | --- |
| 1 | 28 | 8.0% |  |
| 0.9782 | 1 | 0.3% |  |
| 0.96534 | 1 | 0.3% |  |
| 0.95833 | 1 | 0.3% |  |
| 0.95776 | 1 | 0.3% |  |
| 0.95193 | 1 | 0.3% |  |
| 0.95041 | 1 | 0.3% |  |
| 0.94415 | 1 | 0.3% |  |
| 0.93596 | 1 | 0.3% |  |
| 0.93345 | 1 | 0.3% |  |

0  
Boolean

|  |  |
| --- | --- |
| Distinct count | 2 |
| Unique (%) | 0.6% |
| Missing | 0 |
| Missing (%) | 0.0% |
| Memory size | 2.9 KiB |

|  |  |
| --- | --- |
| 1 | 313 |
| 0 | 38 |

Toggle details

- Frequency Table

| Value | Count | Frequency (%) |  |
| --- | --- | --- | --- |
| 1 | 313 | 89.2% |  |
| 0 | 38 | 10.8% |  |

4  
Real number (ℝ)

`ZEROS`

|  |  |
| --- | --- |
| Distinct count | 204 |
| Unique (%) | 58.1% |
| Missing | 0 |
| Missing (%) | 0.0% |
| Infinite | 0 |
| Infinite (%) | 0.0% |

|  |  |
| --- | --- |
| Mean | 0.6010678917378918 |
| Minimum | -1.0 |
| Maximum | 1.0 |
| Zeros | 38 |
| Zeros (%) | 10.8% |
| Memory size | 2.9 KiB |

2020-08-25T01:26:53.111368image/svg+xmlMatplotlib v3.3.1, https://matplotlib.org/

Toggle details

- Statistics
- Histogram(s)
- Common values
- Extreme values

Quantile statistics

|  |  |
| --- | --- |
| Minimum | -1 |
| 5-th percentile | -0.745815 |
| Q1 | 0.41266 |
| median | 0.8092 |
| Q3 | 1 |
| 95-th percentile | 1 |
| Maximum | 1 |
| Range | 2 |
| Interquartile range (IQR) | 0.58734 |

Descriptive statistics

|  |  |
| --- | --- |
| Standard deviation | 0.5198615134 |
| Coefficient of variation (CV) | 0.864896496 |
| Kurtosis | 2.129334839 |
| Mean | 0.6010678917 |
| Median Absolute Deviation (MAD) | 0.1908 |
| Skewness | -1.62777768 |
| Sum | 210.97483 |
| Variance | 0.2702559931 |

- Histogram

2020-08-25T01:26:53.218033image/svg+xmlMatplotlib v3.3.1, https://matplotlib.org/ 

**Histogram with fixed size bins** (bins=10)

| Value | Count | Frequency (%) |  |
| --- | --- | --- | --- |
| 1 | 96 | 27.4% |  |
| 0 | 38 | 10.8% |  |
| -1 | 16 | 4.6% |  |
| 0.9676 | 1 | 0.3% |  |
| 0.98122 | 1 | 0.3% |  |
| 0.38696 | 1 | 0.3% |  |
| 0.66667 | 1 | 0.3% |  |
| 0.06704 | 1 | 0.3% |  |
| 0.86528 | 1 | 0.3% |  |
| 0.8092 | 1 | 0.3% |  |
| 0.6568 | 1 | 0.3% |  |
| 0.98103 | 1 | 0.3% |  |
| 0.65926 | 1 | 0.3% |  |
| 0.72314 | 1 | 0.3% |  |
| 0.91966 | 1 | 0.3% |  |
| 0.98994 | 1 | 0.3% |  |
| 0.67333 | 1 | 0.3% |  |
| 0.46939 | 1 | 0.3% |  |
| 0.85243 | 1 | 0.3% |  |
| 0.46667 | 1 | 0.3% |  |
| 0.79078 | 1 | 0.3% |  |
| 0.59085 | 1 | 0.3% |  |
| 0.73061 | 1 | 0.3% |  |
| 0.63935 | 1 | 0.3% |  |
| 0.98579 | 1 | 0.3% |  |
| Other values (179) | 179 | 51.0% |  |

- Minimum 5 values
- Maximum 5 values

| Value | Count | Frequency (%) |  |
| --- | --- | --- | --- |
| -1 | 16 | 4.6% |  |
| -0.78824 | 1 | 0.3% |  |
| -0.7619 | 1 | 0.3% |  |
| -0.72973 | 1 | 0.3% |  |
| -0.50694 | 1 | 0.3% |  |
| -0.33746 | 1 | 0.3% |  |
| -0.33672 | 1 | 0.3% |  |
| -0.31745 | 1 | 0.3% |  |
| -0.27303 | 1 | 0.3% |  |
| -0.09924 | 1 | 0.3% |  |

| Value | Count | Frequency (%) |  |
| --- | --- | --- | --- |
| 1 | 96 | 27.4% |  |
| 0.99815 | 1 | 0.3% |  |
| 0.99793 | 1 | 0.3% |  |
| 0.99672 | 1 | 0.3% |  |
| 0.99363 | 1 | 0.3% |  |
| 0.99352 | 1 | 0.3% |  |
| 0.9915 | 1 | 0.3% |  |
| 0.98994 | 1 | 0.3% |  |
| 0.98919 | 1 | 0.3% |  |
| 0.98579 | 1 | 0.3% |  |

16  
Real number (ℝ)

`ZEROS`

|  |  |
| --- | --- |
| Distinct count | 254 |
| Unique (%) | 72.4% |
| Missing | 0 |
| Missing (%) | 0.0% |
| Infinite | 0 |
| Infinite (%) | 0.0% |

|  |  |
| --- | --- |
| Mean | 0.38194900284900285 |
| Minimum | -1.0 |
| Maximum | 1.0 |
| Zeros | 25 |
| Zeros (%) | 7.1% |
| Memory size | 2.9 KiB |

2020-08-25T01:26:53.335605image/svg+xmlMatplotlib v3.3.1, https://matplotlib.org/

Toggle details

- Statistics
- Histogram(s)
- Common values
- Extreme values

Quantile statistics

|  |  |
| --- | --- |
| Minimum | -1 |
| 5-th percentile | -0.89465 |
| Q1 | 0 |
| median | 0.59091 |
| Q3 | 0.935705 |
| 95-th percentile | 1 |
| Maximum | 1 |
| Range | 2 |
| Interquartile range (IQR) | 0.935705 |

Descriptive statistics

|  |  |
| --- | --- |
| Standard deviation | 0.6180203542 |
| Coefficient of variation (CV) | 1.618070343 |
| Kurtosis | -0.5301590085 |
| Mean | 0.3819490028 |
| Median Absolute Deviation (MAD) | 0.40909 |
| Skewness | -0.8215933853 |
| Sum | 134.0641 |
| Variance | 0.3819491582 |

- Histogram

2020-08-25T01:26:53.441708image/svg+xmlMatplotlib v3.3.1, https://matplotlib.org/ 

**Histogram with fixed size bins** (bins=10)

| Value | Count | Frequency (%) |  |
| --- | --- | --- | --- |
| 1 | 59 | 16.8% |  |
| 0 | 25 | 7.1% |  |
| -1 | 12 | 3.4% |  |
| -0.375 | 3 | 0.9% |  |
| 0.75 | 2 | 0.6% |  |
| 0.36585 | 2 | 0.6% |  |
| -0.75436 | 1 | 0.3% |  |
| 0.22813 | 1 | 0.3% |  |
| 0.42271 | 1 | 0.3% |  |
| 0.78272 | 1 | 0.3% |  |
| 0.76929 | 1 | 0.3% |  |
| 0.50455 | 1 | 0.3% |  |
| -0.7894 | 1 | 0.3% |  |
| -0.13117 | 1 | 0.3% |  |
| 0.91236 | 1 | 0.3% |  |
| 0.66775 | 1 | 0.3% |  |
| 0.67023 | 1 | 0.3% |  |
| 0.9948 | 1 | 0.3% |  |
| 0.66145 | 1 | 0.3% |  |
| 0.97173 | 1 | 0.3% |  |
| 0.92561 | 1 | 0.3% |  |
| 0.93262 | 1 | 0.3% |  |
| -0.03766 | 1 | 0.3% |  |
| 0.56156 | 1 | 0.3% |  |
| 0.63636 | 1 | 0.3% |  |
| Other values (229) | 229 | 65.2% |  |

- Minimum 5 values
- Maximum 5 values

| Value | Count | Frequency (%) |  |
| --- | --- | --- | --- |
| -1 | 12 | 3.4% |  |
| -0.98039 | 1 | 0.3% |  |
| -0.94315 | 1 | 0.3% |  |
| -0.93104 | 1 | 0.3% |  |
| -0.93001 | 1 | 0.3% |  |
| -0.92282 | 1 | 0.3% |  |
| -0.91456 | 1 | 0.3% |  |
| -0.87474 | 1 | 0.3% |  |
| -0.8615 | 1 | 0.3% |  |
| -0.85803 | 1 | 0.3% |  |

| Value | Count | Frequency (%) |  |
| --- | --- | --- | --- |
| 1 | 59 | 16.8% |  |
| 0.9963 | 1 | 0.3% |  |
| 0.99528 | 1 | 0.3% |  |
| 0.9948 | 1 | 0.3% |  |
| 0.99273 | 1 | 0.3% |  |
| 0.98881 | 1 | 0.3% |  |
| 0.98878 | 1 | 0.3% |  |
| 0.98497 | 1 | 0.3% |  |
| 0.98305 | 1 | 0.3% |  |
| 0.97869 | 1 | 0.3% |  |

17  
Real number (ℝ)

`ZEROS`

|  |  |
| --- | --- |
| Distinct count | 280 |
| Unique (%) | 79.8% |
| Missing | 0 |
| Missing (%) | 0.0% |
| Infinite | 0 |
| Infinite (%) | 0.0% |

|  |  |
| --- | --- |
| Mean | -0.0036168091168091113 |
| Minimum | -1.0 |
| Maximum | 1.0 |
| Zeros | 30 |
| Zeros (%) | 8.5% |
| Memory size | 2.9 KiB |

2020-08-25T01:26:53.564438image/svg+xmlMatplotlib v3.3.1, https://matplotlib.org/

Toggle details

- Statistics
- Histogram(s)
- Common values
- Extreme values

Quantile statistics

|  |  |
| --- | --- |
| Minimum | -1 |
| 5-th percentile | -1 |
| Q1 | -0.22569 |
| median | 0 |
| Q3 | 0.195285 |
| 95-th percentile | 0.99361 |
| Maximum | 1 |
| Range | 2 |
| Interquartile range (IQR) | 0.420975 |

Descriptive statistics

|  |  |
| --- | --- |
| Standard deviation | 0.4967619833 |
| Coefficient of variation (CV) | -137.348134 |
| Kurtosis | 0.008103993261 |
| Mean | -0.003616809117 |
| Median Absolute Deviation (MAD) | 0.21231 |
| Skewness | 0.005083118602 |
| Sum | -1.2695 |
| Variance | 0.246772468 |

- Histogram

2020-08-25T01:26:53.674443image/svg+xmlMatplotlib v3.3.1, https://matplotlib.org/ 

**Histogram with fixed size bins** (bins=10)

| Value | Count | Frequency (%) |  |
| --- | --- | --- | --- |
| 0 | 30 | 8.5% |  |
| -1 | 26 | 7.4% |  |
| 1 | 18 | 5.1% |  |
| -0.68627 | 1 | 0.3% |  |
| -0.03191 | 1 | 0.3% |  |
| -0.39695 | 1 | 0.3% |  |
| -0.57779 | 1 | 0.3% |  |
| -0.11905 | 1 | 0.3% |  |
| 0.65701 | 1 | 0.3% |  |
| 0.67861 | 1 | 0.3% |  |
| 0.98722 | 1 | 0.3% |  |
| 0.01852 | 1 | 0.3% |  |
| -0.07157 | 1 | 0.3% |  |
| -0.3933 | 1 | 0.3% |  |
| 0.19565 | 1 | 0.3% |  |
| 0.19328 | 1 | 0.3% |  |
| -0.03416 | 1 | 0.3% |  |
| 0.07135 | 1 | 0.3% |  |
| -0.07853 | 1 | 0.3% |  |
| 0.62385 | 1 | 0.3% |  |
| 0.03458 | 1 | 0.3% |  |
| -0.17863 | 1 | 0.3% |  |
| 0.83939 | 1 | 0.3% |  |
| 0.01943 | 1 | 0.3% |  |
| 0.12722 | 1 | 0.3% |  |
| Other values (255) | 255 | 72.6% |  |

- Minimum 5 values
- Maximum 5 values

| Value | Count | Frequency (%) |  |
| --- | --- | --- | --- |
| -1 | 26 | 7.4% |  |
| -0.83297 | 1 | 0.3% |  |
| -0.83007 | 1 | 0.3% |  |
| -0.81556 | 1 | 0.3% |  |
| -0.79603 | 1 | 0.3% |  |
| -0.77778 | 1 | 0.3% |  |
| -0.77206 | 1 | 0.3% |  |
| -0.71951 | 1 | 0.3% |  |
| -0.71725 | 1 | 0.3% |  |
| -0.70729 | 1 | 0.3% |  |

| Value | Count | Frequency (%) |  |
| --- | --- | --- | --- |
| 1 | 18 | 5.1% |  |
| 0.98722 | 1 | 0.3% |  |
| 0.95122 | 1 | 0.3% |  |
| 0.93429 | 1 | 0.3% |  |
| 0.91624 | 1 | 0.3% |  |
| 0.87396 | 1 | 0.3% |  |
| 0.85475 | 1 | 0.3% |  |
| 0.8521 | 1 | 0.3% |  |
| 0.83939 | 1 | 0.3% |  |
| 0.82813 | 1 | 0.3% |  |

5  
Real number (ℝ)

`ZEROS`

|  |  |
| --- | --- |
| Distinct count | 259 |
| Unique (%) | 73.8% |
| Missing | 0 |
| Missing (%) | 0.0% |
| Infinite | 0 |
| Infinite (%) | 0.0% |

|  |  |
| --- | --- |
| Mean | 0.11588900284900285 |
| Minimum | -1.0 |
| Maximum | 1.0 |
| Zeros | 46 |
| Zeros (%) | 13.1% |
| Memory size | 2.9 KiB |

2020-08-25T01:26:53.795350image/svg+xmlMatplotlib v3.3.1, https://matplotlib.org/

Toggle details

- Statistics
- Histogram(s)
- Common values
- Extreme values

Quantile statistics

|  |  |
| --- | --- |
| Minimum | -1 |
| 5-th percentile | -1 |
| Q1 | -0.024795 |
| median | 0.0228 |
| Q3 | 0.334655 |
| 95-th percentile | 1 |
| Maximum | 1 |
| Range | 2 |
| Interquartile range (IQR) | 0.35945 |

Descriptive statistics

|  |  |
| --- | --- |
| Standard deviation | 0.460810129 |
| Coefficient of variation (CV) | 3.976305928 |
| Kurtosis | 0.8558761612 |
| Mean | 0.1158890028 |
| Median Absolute Deviation (MAD) | 0.14424 |
| Skewness | -0.2794400302 |
| Sum | 40.67704 |
| Variance | 0.212345975 |

- Histogram

2020-08-25T01:26:53.908180image/svg+xmlMatplotlib v3.3.1, https://matplotlib.org/ 

**Histogram with fixed size bins** (bins=10)

| Value | Count | Frequency (%) |  |
| --- | --- | --- | --- |
| 0 | 46 | 13.1% |  |
| 1 | 26 | 7.4% |  |
| -1 | 23 | 6.6% |  |
| -0.05178 | 1 | 0.3% |  |
| 0.04598 | 1 | 0.3% |  |
| 0.07143 | 1 | 0.3% |  |
| -0.01989 | 1 | 0.3% |  |
| 0.25359 | 1 | 0.3% |  |
| -0.16964 | 1 | 0.3% |  |
| -0.02703 | 1 | 0.3% |  |
| -0.03142 | 1 | 0.3% |  |
| -0.02299 | 1 | 0.3% |  |
| 0.77655 | 1 | 0.3% |  |
| 0.69438 | 1 | 0.3% |  |
| 0.04861 | 1 | 0.3% |  |
| 0.16251 | 1 | 0.3% |  |
| 0.76432 | 1 | 0.3% |  |
| -0.10169 | 1 | 0.3% |  |
| 0.00843 | 1 | 0.3% |  |
| 0.07418 | 1 | 0.3% |  |
| 0.35543 | 1 | 0.3% |  |
| 0.33598 | 1 | 0.3% |  |
| -0.62766 | 1 | 0.3% |  |
| -0.14706 | 1 | 0.3% |  |
| 0.18281 | 1 | 0.3% |  |
| Other values (234) | 234 | 66.7% |  |

- Minimum 5 values
- Maximum 5 values

| Value | Count | Frequency (%) |  |
| --- | --- | --- | --- |
| -1 | 23 | 6.6% |  |
| -0.80553 | 1 | 0.3% |  |
| -0.78509 | 1 | 0.3% |  |
| -0.76087 | 1 | 0.3% |  |
| -0.73371 | 1 | 0.3% |  |
| -0.62766 | 1 | 0.3% |  |
| -0.45455 | 1 | 0.3% |  |
| -0.36156 | 1 | 0.3% |  |
| -0.33203 | 1 | 0.3% |  |
| -0.2963 | 1 | 0.3% |  |

| Value | Count | Frequency (%) |  |
| --- | --- | --- | --- |
| 1 | 26 | 7.4% |  |
| 0.90763 | 1 | 0.3% |  |
| 0.85388 | 1 | 0.3% |  |
| 0.82857 | 1 | 0.3% |  |
| 0.82161 | 1 | 0.3% |  |
| 0.82021 | 1 | 0.3% |  |
| 0.80521 | 1 | 0.3% |  |
| 0.78579 | 1 | 0.3% |  |
| 0.77655 | 1 | 0.3% |  |
| 0.76432 | 1 | 0.3% |  |

13  
Real number (ℝ)

`ZEROS`

|  |  |
| --- | --- |
| Distinct count | 266 |
| Unique (%) | 75.8% |
| Missing | 0 |
| Missing (%) | 0.0% |
| Infinite | 0 |
| Infinite (%) | 0.0% |

|  |  |
| --- | --- |
| Mean | 0.09341367521367522 |
| Minimum | -1.0 |
| Maximum | 1.0 |
| Zeros | 37 |
| Zeros (%) | 10.5% |
| Memory size | 2.9 KiB |

2020-08-25T01:26:54.032288image/svg+xmlMatplotlib v3.3.1, https://matplotlib.org/

Toggle details

- Statistics
- Histogram(s)
- Common values
- Extreme values

Quantile statistics

|  |  |
| --- | --- |
| Minimum | -1 |
| 5-th percentile | -1 |
| Q1 | -0.073725 |
| median | 0.03027 |
| Q3 | 0.37486 |
| 95-th percentile | 1 |
| Maximum | 1 |
| Range | 2 |
| Interquartile range (IQR) | 0.448585 |

Descriptive statistics

|  |  |
| --- | --- |
| Standard deviation | 0.4948726408 |
| Coefficient of variation (CV) | 5.297646621 |
| Kurtosis | 0.2063045796 |
| Mean | 0.09341367521 |
| Median Absolute Deviation (MAD) | 0.19107 |
| Skewness | -0.2194820172 |
| Sum | 32.7882 |
| Variance | 0.2448989306 |

- Histogram

2020-08-25T01:26:54.141416image/svg+xmlMatplotlib v3.3.1, https://matplotlib.org/ 

**Histogram with fixed size bins** (bins=10)

| Value | Count | Frequency (%) |  |
| --- | --- | --- | --- |
| 0 | 37 | 10.5% |  |
| -1 | 24 | 6.8% |  |
| 1 | 23 | 6.6% |  |
| -0.11111 | 3 | 0.9% |  |
| 0.11765 | 2 | 0.6% |  |
| -0.01436 | 2 | 0.6% |  |
| -0.27273 | 1 | 0.3% |  |
| 0.0506 | 1 | 0.3% |  |
| -0.49639 | 1 | 0.3% |  |
| 0.58842 | 1 | 0.3% |  |
| -0.37679 | 1 | 0.3% |  |
| 0.34328 | 1 | 0.3% |  |
| 0.9513 | 1 | 0.3% |  |
| 0.1421 | 1 | 0.3% |  |
| -0.31736 | 1 | 0.3% |  |
| 0.15152 | 1 | 0.3% |  |
| 0.33987 | 1 | 0.3% |  |
| 0.00915 | 1 | 0.3% |  |
| 0.10417 | 1 | 0.3% |  |
| -0.14247 | 1 | 0.3% |  |
| -0.12581 | 1 | 0.3% |  |
| 0.05489 | 1 | 0.3% |  |
| 0.83397 | 1 | 0.3% |  |
| -0.0897 | 1 | 0.3% |  |
| 0.29091 | 1 | 0.3% |  |
| Other values (241) | 241 | 68.7% |  |

- Minimum 5 values
- Maximum 5 values

| Value | Count | Frequency (%) |  |
| --- | --- | --- | --- |
| -1 | 24 | 6.8% |  |
| -0.89375 | 1 | 0.3% |  |
| -0.86402 | 1 | 0.3% |  |
| -0.85727 | 1 | 0.3% |  |
| -0.84848 | 1 | 0.3% |  |
| -0.8209 | 1 | 0.3% |  |
| -0.79313 | 1 | 0.3% |  |
| -0.69707 | 1 | 0.3% |  |
| -0.59212 | 1 | 0.3% |  |
| -0.59043 | 1 | 0.3% |  |

| Value | Count | Frequency (%) |  |
| --- | --- | --- | --- |
| 1 | 23 | 6.6% |  |
| 0.9513 | 1 | 0.3% |  |
| 0.93945 | 1 | 0.3% |  |
| 0.93778 | 1 | 0.3% |  |
| 0.89489 | 1 | 0.3% |  |
| 0.89335 | 1 | 0.3% |  |
| 0.892 | 1 | 0.3% |  |
| 0.88248 | 1 | 0.3% |  |
| 0.87493 | 1 | 0.3% |  |
| 0.86029 | 1 | 0.3% |  |

11  
Real number (ℝ)

`ZEROS`

|  |  |
| --- | --- |
| Distinct count | 269 |
| Unique (%) | 76.6% |
| Missing | 0 |
| Missing (%) | 0.0% |
| Infinite | 0 |
| Infinite (%) | 0.0% |

|  |  |
| --- | --- |
| Mean | 0.15504045584045587 |
| Minimum | -1.0 |
| Maximum | 1.0 |
| Zeros | 37 |
| Zeros (%) | 10.5% |
| Memory size | 2.9 KiB |

2020-08-25T01:26:54.272383image/svg+xmlMatplotlib v3.3.1, https://matplotlib.org/

Toggle details

- Statistics
- Histogram(s)
- Common values
- Extreme values

Quantile statistics

|  |  |
| --- | --- |
| Minimum | -1 |
| 5-th percentile | -0.974265 |
| Q1 | -0.065265 |
| median | 0.02825 |
| Q3 | 0.482375 |
| 95-th percentile | 1 |
| Maximum | 1 |
| Range | 2 |
| Interquartile range (IQR) | 0.54764 |

Descriptive statistics

|  |  |
| --- | --- |
| Standard deviation | 0.4948174492 |
| Coefficient of variation (CV) | 3.191537631 |
| Kurtosis | 0.0523178627 |
| Mean | 0.1550404558 |
| Median Absolute Deviation (MAD) | 0.20472 |
| Skewness | -0.08114890524 |
| Sum | 54.4192 |
| Variance | 0.244844308 |

- Histogram

2020-08-25T01:26:54.381778image/svg+xmlMatplotlib v3.3.1, https://matplotlib.org/ 

**Histogram with fixed size bins** (bins=10)

| Value | Count | Frequency (%) |  |
| --- | --- | --- | --- |
| 0 | 37 | 10.5% |  |
| 1 | 29 | 8.3% |  |
| -1 | 18 | 5.1% |  |
| -0.11111 | 2 | 0.6% |  |
| 0.73746 | 1 | 0.3% |  |
| -0.25712 | 1 | 0.3% |  |
| 0.79346 | 1 | 0.3% |  |
| -0.28909 | 1 | 0.3% |  |
| -0.3871 | 1 | 0.3% |  |
| 0.01419 | 1 | 0.3% |  |
| 0.50122 | 1 | 0.3% |  |
| 0.8323 | 1 | 0.3% |  |
| -0.42444 | 1 | 0.3% |  |
| -0.11073 | 1 | 0.3% |  |
| -0.24077 | 1 | 0.3% |  |
| 0.59942 | 1 | 0.3% |  |
| 0.33266 | 1 | 0.3% |  |
| -0.02809 | 1 | 0.3% |  |
| 0.02575 | 1 | 0.3% |  |
| -0.07496 | 1 | 0.3% |  |
| -0.21649 | 1 | 0.3% |  |
| -0.11734 | 1 | 0.3% |  |
| -0.17755 | 1 | 0.3% |  |
| 0.86121 | 1 | 0.3% |  |
| 0.07808 | 1 | 0.3% |  |
| Other values (244) | 244 | 69.5% |  |

- Minimum 5 values
- Maximum 5 values

| Value | Count | Frequency (%) |  |
| --- | --- | --- | --- |
| -1 | 18 | 5.1% |  |
| -0.94853 | 1 | 0.3% |  |
| -0.89098 | 1 | 0.3% |  |
| -0.67743 | 1 | 0.3% |  |
| -0.62723 | 1 | 0.3% |  |
| -0.49863 | 1 | 0.3% |  |
| -0.49057 | 1 | 0.3% |  |
| -0.47929 | 1 | 0.3% |  |
| -0.43569 | 1 | 0.3% |  |
| -0.42778 | 1 | 0.3% |  |

| Value | Count | Frequency (%) |  |
| --- | --- | --- | --- |
| 1 | 29 | 8.3% |  |
| 0.99557 | 1 | 0.3% |  |
| 0.96748 | 1 | 0.3% |  |
| 0.96301 | 1 | 0.3% |  |
| 0.96167 | 1 | 0.3% |  |
| 0.96161 | 1 | 0.3% |  |
| 0.96128 | 1 | 0.3% |  |
| 0.94749 | 1 | 0.3% |  |
| 0.92643 | 1 | 0.3% |  |
| 0.92453 | 1 | 0.3% |  |

1  
Boolean

`CONSTANT`  
`REJECTED`

|  |  |
| --- | --- |
| Distinct count | 1 |
| Unique (%) | 0.3% |
| Missing | 0 |
| Missing (%) | 0.0% |
| Memory size | 2.9 KiB |

|  |  |
| --- | --- |
| 0 | 351 |

Toggle details

- Frequency Table

| Value | Count | Frequency (%) |  |
| --- | --- | --- | --- |
| 0 | 351 | 100.0% |  |

2  
Real number (ℝ)

`ZEROS`

|  |  |
| --- | --- |
| Distinct count | 219 |
| Unique (%) | 62.4% |
| Missing | 0 |
| Missing (%) | 0.0% |
| Infinite | 0 |
| Infinite (%) | 0.0% |

|  |  |
| --- | --- |
| Mean | 0.6413418518518519 |
| Minimum | -1.0 |
| Maximum | 1.0 |
| Zeros | 25 |
| Zeros (%) | 7.1% |
| Memory size | 2.9 KiB |

2020-08-25T01:26:54.506012image/svg+xmlMatplotlib v3.3.1, https://matplotlib.org/

Toggle details

- Statistics
- Histogram(s)
- Common values
- Extreme values

Quantile statistics

|  |  |
| --- | --- |
| Minimum | -1 |
| 5-th percentile | -0.59233 |
| Q1 | 0.472135 |
| median | 0.87111 |
| Q3 | 1 |
| 95-th percentile | 1 |
| Maximum | 1 |
| Range | 2 |
| Interquartile range (IQR) | 0.527865 |

Descriptive statistics

|  |  |
| --- | --- |
| Standard deviation | 0.4977082025 |
| Coefficient of variation (CV) | 0.776041983 |
| Kurtosis | 3.086346703 |
| Mean | 0.6413418519 |
| Median Absolute Deviation (MAD) | 0.12889 |
| Skewness | -1.851541305 |
| Sum | 225.11099 |
| Variance | 0.2477134549 |

- Histogram

2020-08-25T01:26:54.621006image/svg+xmlMatplotlib v3.3.1, https://matplotlib.org/ 

**Histogram with fixed size bins** (bins=10)

| Value | Count | Frequency (%) |  |
| --- | --- | --- | --- |
| 1 | 95 | 27.1% |  |
| 0 | 25 | 7.1% |  |
| -1 | 15 | 4.3% |  |
| 0.94631 | 1 | 0.3% |  |
| 0.96355 | 1 | 0.3% |  |
| 0.85013 | 1 | 0.3% |  |
| -0.67935 | 1 | 0.3% |  |
| 0.65845 | 1 | 0.3% |  |
| 0.35346 | 1 | 0.3% |  |
| 0.92436 | 1 | 0.3% |  |
| 0.87032 | 1 | 0.3% |  |
| 0.84843 | 1 | 0.3% |  |
| 0.88853 | 1 | 0.3% |  |
| 0.21429 | 1 | 0.3% |  |
| 0.8941 | 1 | 0.3% |  |
| 0.25316 | 1 | 0.3% |  |
| 0.93669 | 1 | 0.3% |  |
| 0.87578 | 1 | 0.3% |  |
| -0.205 | 1 | 0.3% |  |
| 0.87772 | 1 | 0.3% |  |
| 0.03852 | 1 | 0.3% |  |
| 0.98182 | 1 | 0.3% |  |
| 0.45455 | 1 | 0.3% |  |
| 0.98002 | 1 | 0.3% |  |
| 0.05866 | 1 | 0.3% |  |
| Other values (194) | 194 | 55.3% |  |

- Minimum 5 values
- Maximum 5 values

| Value | Count | Frequency (%) |  |
| --- | --- | --- | --- |
| -1 | 15 | 4.3% |  |
| -0.67935 | 1 | 0.3% |  |
| -0.65625 | 1 | 0.3% |  |
| -0.64286 | 1 | 0.3% |  |
| -0.5418 | 1 | 0.3% |  |
| -0.26667 | 1 | 0.3% |  |
| -0.205 | 1 | 0.3% |  |
| -0.01864 | 1 | 0.3% |  |
| -0.00641 | 1 | 0.3% |  |
| 0 | 25 | 7.1% |  |

| Value | Count | Frequency (%) |  |
| --- | --- | --- | --- |
| 1 | 95 | 27.1% |  |
| 0.99701 | 1 | 0.3% |  |
| 0.99645 | 1 | 0.3% |  |
| 0.99539 | 1 | 0.3% |  |
| 0.99449 | 1 | 0.3% |  |
| 0.99025 | 1 | 0.3% |  |
| 0.98822 | 1 | 0.3% |  |
| 0.98455 | 1 | 0.3% |  |
| 0.98182 | 1 | 0.3% |  |
| 0.98166 | 1 | 0.3% |  |

target  
Boolean

|  |  |
| --- | --- |
| Distinct count | 2 |
| Unique (%) | 0.6% |
| Missing | 0 |
| Missing (%) | 0.0% |
| Memory size | 2.9 KiB |

|  |  |
| --- | --- |
| 1 | 225 |
| 0 | 126 |

Toggle details

- Frequency Table

| Value | Count | Frequency (%) |  |
| --- | --- | --- | --- |
| 1 | 225 | 64.1% |  |
| 0 | 126 | 35.9% |  |

# Interactions

15 19 27 26 8 24 21 12 32 9 4 16 17 5 13 11 2

15 19 27 26 8 24 21 12 32 9 4 16 17 5 13 11 2

2020-08-25T01:26:04.092914image/svg+xmlMatplotlib v3.3.1, https://matplotlib.org/

2020-08-25T01:26:04.249366image/svg+xmlMatplotlib v3.3.1, https://matplotlib.org/

2020-08-25T01:26:04.402130image/svg+xmlMatplotlib v3.3.1, https://matplotlib.org/

2020-08-25T01:26:04.756571image/svg+xmlMatplotlib v3.3.1, https://matplotlib.org/

2020-08-25T01:26:04.921834image/svg+xmlMatplotlib v3.3.1, https://matplotlib.org/

2020-08-25T01:26:05.092365image/svg+xmlMatplotlib v3.3.1, https://matplotlib.org/

2020-08-25T01:26:05.247776image/svg+xmlMatplotlib v3.3.1, https://matplotlib.org/

2020-08-25T01:26:05.402018image/svg+xmlMatplotlib v3.3.1, https://matplotlib.org/

2020-08-25T01:26:05.557957image/svg+xmlMatplotlib v3.3.1, https://matplotlib.org/

2020-08-25T01:26:05.732793image/svg+xmlMatplotlib v3.3.1, https://matplotlib.org/

2020-08-25T01:26:05.886390image/svg+xmlMatplotlib v3.3.1, https://matplotlib.org/

2020-08-25T01:26:06.041589image/svg+xmlMatplotlib v3.3.1, https://matplotlib.org/

2020-08-25T01:26:06.198677image/svg+xmlMatplotlib v3.3.1, https://matplotlib.org/

2020-08-25T01:26:06.356292image/svg+xmlMatplotlib v3.3.1, https://matplotlib.org/

2020-08-25T01:26:06.516382image/svg+xmlMatplotlib v3.3.1, https://matplotlib.org/

2020-08-25T01:26:06.676732image/svg+xmlMatplotlib v3.3.1, https://matplotlib.org/

2020-08-25T01:26:06.831850image/svg+xmlMatplotlib v3.3.1, https://matplotlib.org/

15 19 27 26 8 24 21 12 32 9 4 16 17 5 13 11 2

2020-08-25T01:26:06.983860image/svg+xmlMatplotlib v3.3.1, https://matplotlib.org/

2020-08-25T01:26:07.137230image/svg+xmlMatplotlib v3.3.1, https://matplotlib.org/

2020-08-25T01:26:07.296528image/svg+xmlMatplotlib v3.3.1, https://matplotlib.org/

2020-08-25T01:26:07.449665image/svg+xmlMatplotlib v3.3.1, https://matplotlib.org/

2020-08-25T01:26:07.603012image/svg+xmlMatplotlib v3.3.1, https://matplotlib.org/

2020-08-25T01:26:07.763270image/svg+xmlMatplotlib v3.3.1, https://matplotlib.org/

2020-08-25T01:26:07.922480image/svg+xmlMatplotlib v3.3.1, https://matplotlib.org/

2020-08-25T01:26:08.074181image/svg+xmlMatplotlib v3.3.1, https://matplotlib.org/

2020-08-25T01:26:08.232528image/svg+xmlMatplotlib v3.3.1, https://matplotlib.org/

2020-08-25T01:26:08.396953image/svg+xmlMatplotlib v3.3.1, https://matplotlib.org/

2020-08-25T01:26:08.552666image/svg+xmlMatplotlib v3.3.1, https://matplotlib.org/

2020-08-25T01:26:08.718147image/svg+xmlMatplotlib v3.3.1, https://matplotlib.org/

2020-08-25T01:26:08.899614image/svg+xmlMatplotlib v3.3.1, https://matplotlib.org/

2020-08-25T01:26:09.075881image/svg+xmlMatplotlib v3.3.1, https://matplotlib.org/

2020-08-25T01:26:09.454187image/svg+xmlMatplotlib v3.3.1, https://matplotlib.org/

2020-08-25T01:26:09.614590image/svg+xmlMatplotlib v3.3.1, https://matplotlib.org/

2020-08-25T01:26:09.771206image/svg+xmlMatplotlib v3.3.1, https://matplotlib.org/

15 19 27 26 8 24 21 12 32 9 4 16 17 5 13 11 2

2020-08-25T01:26:09.927907image/svg+xmlMatplotlib v3.3.1, https://matplotlib.org/

2020-08-25T01:26:10.084629image/svg+xmlMatplotlib v3.3.1, https://matplotlib.org/

2020-08-25T01:26:10.247963image/svg+xmlMatplotlib v3.3.1, https://matplotlib.org/

2020-08-25T01:26:10.413640image/svg+xmlMatplotlib v3.3.1, https://matplotlib.org/

2020-08-25T01:26:10.583105image/svg+xmlMatplotlib v3.3.1, https://matplotlib.org/

2020-08-25T01:26:10.747660image/svg+xmlMatplotlib v3.3.1, https://matplotlib.org/

2020-08-25T01:26:10.899652image/svg+xmlMatplotlib v3.3.1, https://matplotlib.org/

2020-08-25T01:26:11.051985image/svg+xmlMatplotlib v3.3.1, https://matplotlib.org/

2020-08-25T01:26:11.206275image/svg+xmlMatplotlib v3.3.1, https://matplotlib.org/

2020-08-25T01:26:11.361424image/svg+xmlMatplotlib v3.3.1, https://matplotlib.org/

2020-08-25T01:26:11.514832image/svg+xmlMatplotlib v3.3.1, https://matplotlib.org/

2020-08-25T01:26:11.665898image/svg+xmlMatplotlib v3.3.1, https://matplotlib.org/

2020-08-25T01:26:11.829999image/svg+xmlMatplotlib v3.3.1, https://matplotlib.org/

2020-08-25T01:26:12.003763image/svg+xmlMatplotlib v3.3.1, https://matplotlib.org/

2020-08-25T01:26:12.158236image/svg+xmlMatplotlib v3.3.1, https://matplotlib.org/

2020-08-25T01:26:12.320782image/svg+xmlMatplotlib v3.3.1, https://matplotlib.org/

2020-08-25T01:26:12.472391image/svg+xmlMatplotlib v3.3.1, https://matplotlib.org/

15 19 27 26 8 24 21 12 32 9 4 16 17 5 13 11 2

2020-08-25T01:26:12.625532image/svg+xmlMatplotlib v3.3.1, https://matplotlib.org/

2020-08-25T01:26:12.782559image/svg+xmlMatplotlib v3.3.1, https://matplotlib.org/

2020-08-25T01:26:12.935727image/svg+xmlMatplotlib v3.3.1, https://matplotlib.org/

2020-08-25T01:26:13.088875image/svg+xmlMatplotlib v3.3.1, https://matplotlib.org/

2020-08-25T01:26:13.242263image/svg+xmlMatplotlib v3.3.1, https://matplotlib.org/

2020-08-25T01:26:13.403320image/svg+xmlMatplotlib v3.3.1, https://matplotlib.org/

2020-08-25T01:26:13.558882image/svg+xmlMatplotlib v3.3.1, https://matplotlib.org/

2020-08-25T01:26:13.711416image/svg+xmlMatplotlib v3.3.1, https://matplotlib.org/

2020-08-25T01:26:14.072587image/svg+xmlMatplotlib v3.3.1, https://matplotlib.org/

2020-08-25T01:26:14.225274image/svg+xmlMatplotlib v3.3.1, https://matplotlib.org/

2020-08-25T01:26:14.379464image/svg+xmlMatplotlib v3.3.1, https://matplotlib.org/

2020-08-25T01:26:14.537499image/svg+xmlMatplotlib v3.3.1, https://matplotlib.org/

2020-08-25T01:26:14.689468image/svg+xmlMatplotlib v3.3.1, https://matplotlib.org/

2020-08-25T01:26:14.843367image/svg+xmlMatplotlib v3.3.1, https://matplotlib.org/

2020-08-25T01:26:14.995489image/svg+xmlMatplotlib v3.3.1, https://matplotlib.org/

2020-08-25T01:26:15.149287image/svg+xmlMatplotlib v3.3.1, https://matplotlib.org/

2020-08-25T01:26:15.302634image/svg+xmlMatplotlib v3.3.1, https://matplotlib.org/

15 19 27 26 8 24 21 12 32 9 4 16 17 5 13 11 2

2020-08-25T01:26:15.453922image/svg+xmlMatplotlib v3.3.1, https://matplotlib.org/

2020-08-25T01:26:15.604491image/svg+xmlMatplotlib v3.3.1, https://matplotlib.org/

2020-08-25T01:26:15.768173image/svg+xmlMatplotlib v3.3.1, https://matplotlib.org/

2020-08-25T01:26:15.929496image/svg+xmlMatplotlib v3.3.1, https://matplotlib.org/

2020-08-25T01:26:16.088432image/svg+xmlMatplotlib v3.3.1, https://matplotlib.org/

2020-08-25T01:26:16.243310image/svg+xmlMatplotlib v3.3.1, https://matplotlib.org/

2020-08-25T01:26:16.403516image/svg+xmlMatplotlib v3.3.1, https://matplotlib.org/

2020-08-25T01:26:16.556149image/svg+xmlMatplotlib v3.3.1, https://matplotlib.org/

2020-08-25T01:26:16.707933image/svg+xmlMatplotlib v3.3.1, https://matplotlib.org/

2020-08-25T01:26:16.861269image/svg+xmlMatplotlib v3.3.1, https://matplotlib.org/

2020-08-25T01:26:17.014348image/svg+xmlMatplotlib v3.3.1, https://matplotlib.org/

2020-08-25T01:26:17.170456image/svg+xmlMatplotlib v3.3.1, https://matplotlib.org/

2020-08-25T01:26:17.325416image/svg+xmlMatplotlib v3.3.1, https://matplotlib.org/

2020-08-25T01:26:17.479729image/svg+xmlMatplotlib v3.3.1, https://matplotlib.org/

2020-08-25T01:26:17.634079image/svg+xmlMatplotlib v3.3.1, https://matplotlib.org/

2020-08-25T01:26:17.792907image/svg+xmlMatplotlib v3.3.1, https://matplotlib.org/

2020-08-25T01:26:17.952387image/svg+xmlMatplotlib v3.3.1, https://matplotlib.org/

15 19 27 26 8 24 21 12 32 9 4 16 17 5 13 11 2

2020-08-25T01:26:18.119375image/svg+xmlMatplotlib v3.3.1, https://matplotlib.org/

2020-08-25T01:26:18.281531image/svg+xmlMatplotlib v3.3.1, https://matplotlib.org/

2020-08-25T01:26:18.636348image/svg+xmlMatplotlib v3.3.1, https://matplotlib.org/

2020-08-25T01:26:18.789168image/svg+xmlMatplotlib v3.3.1, https://matplotlib.org/

2020-08-25T01:26:18.956186image/svg+xmlMatplotlib v3.3.1, https://matplotlib.org/

2020-08-25T01:26:19.109700image/svg+xmlMatplotlib v3.3.1, https://matplotlib.org/

2020-08-25T01:26:19.263812image/svg+xmlMatplotlib v3.3.1, https://matplotlib.org/

2020-08-25T01:26:19.418959image/svg+xmlMatplotlib v3.3.1, https://matplotlib.org/

2020-08-25T01:26:19.572068image/svg+xmlMatplotlib v3.3.1, https://matplotlib.org/

2020-08-25T01:26:19.726446image/svg+xmlMatplotlib v3.3.1, https://matplotlib.org/

2020-08-25T01:26:19.876618image/svg+xmlMatplotlib v3.3.1, https://matplotlib.org/

2020-08-25T01:26:20.026485image/svg+xmlMatplotlib v3.3.1, https://matplotlib.org/

2020-08-25T01:26:20.175014image/svg+xmlMatplotlib v3.3.1, https://matplotlib.org/

2020-08-25T01:26:20.322711image/svg+xmlMatplotlib v3.3.1, https://matplotlib.org/

2020-08-25T01:26:20.473849image/svg+xmlMatplotlib v3.3.1, https://matplotlib.org/

2020-08-25T01:26:20.621990image/svg+xmlMatplotlib v3.3.1, https://matplotlib.org/

2020-08-25T01:26:20.775758image/svg+xmlMatplotlib v3.3.1, https://matplotlib.org/

15 19 27 26 8 24 21 12 32 9 4 16 17 5 13 11 2

2020-08-25T01:26:20.925560image/svg+xmlMatplotlib v3.3.1, https://matplotlib.org/

2020-08-25T01:26:21.074953image/svg+xmlMatplotlib v3.3.1, https://matplotlib.org/

2020-08-25T01:26:21.220918image/svg+xmlMatplotlib v3.3.1, https://matplotlib.org/

2020-08-25T01:26:21.373309image/svg+xmlMatplotlib v3.3.1, https://matplotlib.org/

2020-08-25T01:26:21.518464image/svg+xmlMatplotlib v3.3.1, https://matplotlib.org/

2020-08-25T01:26:21.667615image/svg+xmlMatplotlib v3.3.1, https://matplotlib.org/

2020-08-25T01:26:21.814112image/svg+xmlMatplotlib v3.3.1, https://matplotlib.org/

2020-08-25T01:26:21.970088image/svg+xmlMatplotlib v3.3.1, https://matplotlib.org/

2020-08-25T01:26:22.117286image/svg+xmlMatplotlib v3.3.1, https://matplotlib.org/

2020-08-25T01:26:22.263899image/svg+xmlMatplotlib v3.3.1, https://matplotlib.org/

2020-08-25T01:26:22.412756image/svg+xmlMatplotlib v3.3.1, https://matplotlib.org/

2020-08-25T01:26:22.568883image/svg+xmlMatplotlib v3.3.1, https://matplotlib.org/

2020-08-25T01:26:22.714839image/svg+xmlMatplotlib v3.3.1, https://matplotlib.org/

2020-08-25T01:26:23.041771image/svg+xmlMatplotlib v3.3.1, https://matplotlib.org/

2020-08-25T01:26:23.189116image/svg+xmlMatplotlib v3.3.1, https://matplotlib.org/

2020-08-25T01:26:23.336768image/svg+xmlMatplotlib v3.3.1, https://matplotlib.org/

2020-08-25T01:26:23.485935image/svg+xmlMatplotlib v3.3.1, https://matplotlib.org/

15 19 27 26 8 24 21 12 32 9 4 16 17 5 13 11 2

2020-08-25T01:26:23.631767image/svg+xmlMatplotlib v3.3.1, https://matplotlib.org/

2020-08-25T01:26:23.777828image/svg+xmlMatplotlib v3.3.1, https://matplotlib.org/

2020-08-25T01:26:23.950300image/svg+xmlMatplotlib v3.3.1, https://matplotlib.org/

2020-08-25T01:26:24.094653image/svg+xmlMatplotlib v3.3.1, https://matplotlib.org/

2020-08-25T01:26:24.244718image/svg+xmlMatplotlib v3.3.1, https://matplotlib.org/

2020-08-25T01:26:24.387456image/svg+xmlMatplotlib v3.3.1, https://matplotlib.org/

2020-08-25T01:26:24.534541image/svg+xmlMatplotlib v3.3.1, https://matplotlib.org/

2020-08-25T01:26:24.680854image/svg+xmlMatplotlib v3.3.1, https://matplotlib.org/

2020-08-25T01:26:24.832786image/svg+xmlMatplotlib v3.3.1, https://matplotlib.org/

2020-08-25T01:26:24.980465image/svg+xmlMatplotlib v3.3.1, https://matplotlib.org/

2020-08-25T01:26:25.141568image/svg+xmlMatplotlib v3.3.1, https://matplotlib.org/

2020-08-25T01:26:25.289729image/svg+xmlMatplotlib v3.3.1, https://matplotlib.org/

2020-08-25T01:26:25.436806image/svg+xmlMatplotlib v3.3.1, https://matplotlib.org/

2020-08-25T01:26:25.584366image/svg+xmlMatplotlib v3.3.1, https://matplotlib.org/

2020-08-25T01:26:25.729886image/svg+xmlMatplotlib v3.3.1, https://matplotlib.org/

2020-08-25T01:26:25.876577image/svg+xmlMatplotlib v3.3.1, https://matplotlib.org/

2020-08-25T01:26:26.026079image/svg+xmlMatplotlib v3.3.1, https://matplotlib.org/

15 19 27 26 8 24 21 12 32 9 4 16 17 5 13 11 2

2020-08-25T01:26:26.187538image/svg+xmlMatplotlib v3.3.1, https://matplotlib.org/

2020-08-25T01:26:26.334492image/svg+xmlMatplotlib v3.3.1, https://matplotlib.org/

2020-08-25T01:26:26.483372image/svg+xmlMatplotlib v3.3.1, https://matplotlib.org/

2020-08-25T01:26:26.628840image/svg+xmlMatplotlib v3.3.1, https://matplotlib.org/

2020-08-25T01:26:26.781820image/svg+xmlMatplotlib v3.3.1, https://matplotlib.org/

2020-08-25T01:26:26.929223image/svg+xmlMatplotlib v3.3.1, https://matplotlib.org/

2020-08-25T01:26:27.078516image/svg+xmlMatplotlib v3.3.1, https://matplotlib.org/

2020-08-25T01:26:27.411135image/svg+xmlMatplotlib v3.3.1, https://matplotlib.org/

2020-08-25T01:26:27.560125image/svg+xmlMatplotlib v3.3.1, https://matplotlib.org/

2020-08-25T01:26:27.706281image/svg+xmlMatplotlib v3.3.1, https://matplotlib.org/

2020-08-25T01:26:27.860568image/svg+xmlMatplotlib v3.3.1, https://matplotlib.org/

2020-08-25T01:26:28.011419image/svg+xmlMatplotlib v3.3.1, https://matplotlib.org/

2020-08-25T01:26:28.157502image/svg+xmlMatplotlib v3.3.1, https://matplotlib.org/

2020-08-25T01:26:28.304772image/svg+xmlMatplotlib v3.3.1, https://matplotlib.org/

2020-08-25T01:26:28.461320image/svg+xmlMatplotlib v3.3.1, https://matplotlib.org/

2020-08-25T01:26:28.616136image/svg+xmlMatplotlib v3.3.1, https://matplotlib.org/

2020-08-25T01:26:28.765368image/svg+xmlMatplotlib v3.3.1, https://matplotlib.org/

15 19 27 26 8 24 21 12 32 9 4 16 17 5 13 11 2

2020-08-25T01:26:28.916482image/svg+xmlMatplotlib v3.3.1, https://matplotlib.org/

2020-08-25T01:26:29.069005image/svg+xmlMatplotlib v3.3.1, https://matplotlib.org/

2020-08-25T01:26:29.219408image/svg+xmlMatplotlib v3.3.1, https://matplotlib.org/

2020-08-25T01:26:29.363297image/svg+xmlMatplotlib v3.3.1, https://matplotlib.org/

2020-08-25T01:26:29.512990image/svg+xmlMatplotlib v3.3.1, https://matplotlib.org/

2020-08-25T01:26:29.657172image/svg+xmlMatplotlib v3.3.1, https://matplotlib.org/

2020-08-25T01:26:29.804336image/svg+xmlMatplotlib v3.3.1, https://matplotlib.org/

2020-08-25T01:26:29.947965image/svg+xmlMatplotlib v3.3.1, https://matplotlib.org/

2020-08-25T01:26:30.094568image/svg+xmlMatplotlib v3.3.1, https://matplotlib.org/

2020-08-25T01:26:30.241811image/svg+xmlMatplotlib v3.3.1, https://matplotlib.org/

2020-08-25T01:26:30.383780image/svg+xmlMatplotlib v3.3.1, https://matplotlib.org/

2020-08-25T01:26:30.531038image/svg+xmlMatplotlib v3.3.1, https://matplotlib.org/

2020-08-25T01:26:30.678941image/svg+xmlMatplotlib v3.3.1, https://matplotlib.org/

2020-08-25T01:26:30.825219image/svg+xmlMatplotlib v3.3.1, https://matplotlib.org/

2020-08-25T01:26:30.967743image/svg+xmlMatplotlib v3.3.1, https://matplotlib.org/
