# Supplementary material for: TPOT-NN: augmenting tree-based automated machine learning with neural network estimators
Source: Genet Program Evolvable Mach. Author manuscript; Available in PMC 2025 Aug 6. (PMC12327408; doi:10.1007/s10710-021-09401-z)
Supplement: Supplementary Material 1 [file NIHMS2035489-supplement-Supplementary_Material_1.zip › New folder/mushroom.html]

mushroom 

Toggle navigationmushroom

- Overview
- Variables
- Interactions
- Correlations
- Missing values
- Sample
- Duplicate rows

# Overview

- Overview
- Reproduction
- Warnings 11

Dataset statistics

|  |  |
| --- | --- |
| Number of variables | 20 |
| Number of observations | 8124 |
| Missing cells | 0 |
| Missing cells (%) | 0.0% |
| Duplicate rows | 4528 |
| Duplicate rows (%) | 55.7% |
| Total size in memory | 1.2 MiB |
| Average record size in memory | 160.0 B |

Variable types

|  |  |
| --- | --- |
| NUM | 9 |
| BOOL | 6 |
| CAT | 5 |

Reproduction

|  |  |
| --- | --- |
| Analysis started | 2020-08-25 01:41:17.359208 |
| Analysis finished | 2020-08-25 01:41:32.272665 |
| Duration | 14.91 seconds |
| Version | pandas-profiling v2.8.0 |
| Command line | `pandas_profiling --config_file config.yaml [YOUR_FILE.csv]` |
| Download configuration | config.yaml |

Warnings

|  |  |
| --- | --- |
| `veil-type` has constant value "0" | Constant |
| Dataset has 4528 (55.7%) duplicate rows | Duplicates |
| `cap-shape` has 452 (5.6%) zeros | Zeros |
| `stalk-color-above-ring` has 448 (5.5%) zeros | Zeros |
| `gill-color` has 408 (5.0%) zeros | Zeros |
| `population` has 384 (4.7%) zeros | Zeros |
| `odor` has 400 (4.9%) zeros | Zeros |
| `ring-type` has 2776 (34.2%) zeros | Zeros |
| `cap-color` has 2284 (28.1%) zeros | Zeros |
| `habitat` has 2148 (26.4%) zeros | Zeros |
| `stalk-root` has 2480 (30.5%) zeros | Zeros |

# Variables

cap-shape  
Real number (ℝ≥0)

`ZEROS`

|  |  |
| --- | --- |
| Distinct count | 6 |
| Unique (%) | 0.1% |
| Missing | 0 |
| Missing (%) | 0.0% |
| Infinite | 0 |
| Infinite (%) | 0.0% |

|  |  |
| --- | --- |
| Mean | 2.4918759231905465 |
| Minimum | 0 |
| Maximum | 5 |
| Zeros | 452 |
| Zeros (%) | 5.6% |
| Memory size | 63.6 KiB |

2020-08-25T01:41:32.325117image/svg+xmlMatplotlib v3.3.1, https://matplotlib.org/

Toggle details

- Statistics
- Histogram(s)
- Common values
- Extreme values

Quantile statistics

|  |  |
| --- | --- |
| Minimum | 0 |
| 5-th percentile | 0 |
| Q1 | 2 |
| median | 2 |
| Q3 | 3 |
| 95-th percentile | 4 |
| Maximum | 5 |
| Range | 5 |
| Interquartile range (IQR) | 1 |

Descriptive statistics

|  |  |
| --- | --- |
| Standard deviation | 0.9012871786 |
| Coefficient of variation (CV) | 0.3616902311 |
| Kurtosis | 1.371745853 |
| Mean | 2.491875923 |
| Median Absolute Deviation (MAD) | 1 |
| Skewness | -0.6195585645 |
| Sum | 20244 |
| Variance | 0.8123185782 |

- Histogram

2020-08-25T01:41:32.432531image/svg+xmlMatplotlib v3.3.1, https://matplotlib.org/ 

**Histogram with fixed size bins** (bins=10)

| Value | Count | Frequency (%) |  |
| --- | --- | --- | --- |
| 2 | 3656 | 45.0% |  |
| 3 | 3152 | 38.8% |  |
| 4 | 828 | 10.2% |  |
| 0 | 452 | 5.6% |  |
| 5 | 32 | 0.4% |  |
| 1 | 4 | < 0.1% |  |

- Minimum 5 values
- Maximum 5 values

| Value | Count | Frequency (%) |  |
| --- | --- | --- | --- |
| 0 | 452 | 5.6% |  |
| 1 | 4 | < 0.1% |  |
| 2 | 3656 | 45.0% |  |
| 3 | 3152 | 38.8% |  |
| 4 | 828 | 10.2% |  |
| 5 | 32 | 0.4% |  |

| Value | Count | Frequency (%) |  |
| --- | --- | --- | --- |
| 5 | 32 | 0.4% |  |
| 4 | 828 | 10.2% |  |
| 3 | 3152 | 38.8% |  |
| 2 | 3656 | 45.0% |  |
| 1 | 4 | < 0.1% |  |
| 0 | 452 | 5.6% |  |

stalk-color-above-ring  
Real number (ℝ≥0)

`ZEROS`

|  |  |
| --- | --- |
| Distinct count | 9 |
| Unique (%) | 0.1% |
| Missing | 0 |
| Missing (%) | 0.0% |
| Infinite | 0 |
| Infinite (%) | 0.0% |

|  |  |
| --- | --- |
| Mean | 5.4465780403742 |
| Minimum | 0 |
| Maximum | 8 |
| Zeros | 448 |
| Zeros (%) | 5.5% |
| Memory size | 63.6 KiB |

2020-08-25T01:41:32.544074image/svg+xmlMatplotlib v3.3.1, https://matplotlib.org/

Toggle details

- Statistics
- Histogram(s)
- Common values
- Extreme values

Quantile statistics

|  |  |
| --- | --- |
| Minimum | 0 |
| 5-th percentile | 0 |
| Q1 | 5 |
| median | 7 |
| Q3 | 7 |
| 95-th percentile | 7 |
| Maximum | 8 |
| Range | 8 |
| Interquartile range (IQR) | 2 |

Descriptive statistics

|  |  |
| --- | --- |
| Standard deviation | 2.143900327 |
| Coefficient of variation (CV) | 0.3936233561 |
| Kurtosis | 0.5917901093 |
| Mean | 5.44657804 |
| Median Absolute Deviation (MAD) | 0 |
| Skewness | -1.301345538 |
| Sum | 44248 |
| Variance | 4.596308614 |

- Histogram

2020-08-25T01:41:32.659925image/svg+xmlMatplotlib v3.3.1, https://matplotlib.org/ 

**Histogram with fixed size bins** (bins=10)

| Value | Count | Frequency (%) |  |
| --- | --- | --- | --- |
| 7 | 4464 | 54.9% |  |
| 5 | 1872 | 23.0% |  |
| 3 | 576 | 7.1% |  |
| 0 | 448 | 5.5% |  |
| 1 | 432 | 5.3% |  |
| 4 | 192 | 2.4% |  |
| 6 | 96 | 1.2% |  |
| 2 | 36 | 0.4% |  |
| 8 | 8 | 0.1% |  |

- Minimum 5 values
- Maximum 5 values

| Value | Count | Frequency (%) |  |
| --- | --- | --- | --- |
| 0 | 448 | 5.5% |  |
| 1 | 432 | 5.3% |  |
| 2 | 36 | 0.4% |  |
| 3 | 576 | 7.1% |  |
| 4 | 192 | 2.4% |  |
| 5 | 1872 | 23.0% |  |
| 6 | 96 | 1.2% |  |
| 7 | 4464 | 54.9% |  |
| 8 | 8 | 0.1% |  |

| Value | Count | Frequency (%) |  |
| --- | --- | --- | --- |
| 8 | 8 | 0.1% |  |
| 7 | 4464 | 54.9% |  |
| 6 | 96 | 1.2% |  |
| 5 | 1872 | 23.0% |  |
| 4 | 192 | 2.4% |  |
| 3 | 576 | 7.1% |  |
| 2 | 36 | 0.4% |  |
| 1 | 432 | 5.3% |  |
| 0 | 448 | 5.5% |  |

gill-color  
Real number (ℝ≥0)

`ZEROS`

|  |  |
| --- | --- |
| Distinct count | 12 |
| Unique (%) | 0.1% |
| Missing | 0 |
| Missing (%) | 0.0% |
| Infinite | 0 |
| Infinite (%) | 0.0% |

|  |  |
| --- | --- |
| Mean | 4.729443623830625 |
| Minimum | 0 |
| Maximum | 11 |
| Zeros | 408 |
| Zeros (%) | 5.0% |
| Memory size | 63.6 KiB |

2020-08-25T01:41:32.778340image/svg+xmlMatplotlib v3.3.1, https://matplotlib.org/

Toggle details

- Statistics
- Histogram(s)
- Common values
- Extreme values

Quantile statistics

|  |  |
| --- | --- |
| Minimum | 0 |
| 5-th percentile | 0 |
| Q1 | 2 |
| median | 4 |
| Q3 | 7 |
| 95-th percentile | 10 |
| Maximum | 11 |
| Range | 11 |
| Interquartile range (IQR) | 5 |

Descriptive statistics

|  |  |
| --- | --- |
| Standard deviation | 3.342401907 |
| Coefficient of variation (CV) | 0.7067220107 |
| Kurtosis | -1.32490759 |
| Mean | 4.729443624 |
| Median Absolute Deviation (MAD) | 3 |
| Skewness | 0.3387940788 |
| Sum | 38422 |
| Variance | 11.17165051 |

- Histogram

2020-08-25T01:41:32.884343image/svg+xmlMatplotlib v3.3.1, https://matplotlib.org/ 

**Histogram with fixed size bins** (bins=10)

| Value | Count | Frequency (%) |  |
| --- | --- | --- | --- |
| 2 | 1728 | 21.3% |  |
| 7 | 1492 | 18.4% |  |
| 10 | 1202 | 14.8% |  |
| 1 | 1048 | 12.9% |  |
| 4 | 752 | 9.3% |  |
| 3 | 732 | 9.0% |  |
| 8 | 492 | 6.1% |  |
| 0 | 408 | 5.0% |  |
| 9 | 96 | 1.2% |  |
| 11 | 86 | 1.1% |  |
| 6 | 64 | 0.8% |  |
| 5 | 24 | 0.3% |  |

- Minimum 5 values
- Maximum 5 values

| Value | Count | Frequency (%) |  |
| --- | --- | --- | --- |
| 0 | 408 | 5.0% |  |
| 1 | 1048 | 12.9% |  |
| 2 | 1728 | 21.3% |  |
| 3 | 732 | 9.0% |  |
| 4 | 752 | 9.3% |  |
| 5 | 24 | 0.3% |  |
| 6 | 64 | 0.8% |  |
| 7 | 1492 | 18.4% |  |
| 8 | 492 | 6.1% |  |
| 9 | 96 | 1.2% |  |

| Value | Count | Frequency (%) |  |
| --- | --- | --- | --- |
| 11 | 86 | 1.1% |  |
| 10 | 1202 | 14.8% |  |
| 9 | 96 | 1.2% |  |
| 8 | 492 | 6.1% |  |
| 7 | 1492 | 18.4% |  |
| 6 | 64 | 0.8% |  |
| 5 | 24 | 0.3% |  |
| 4 | 752 | 9.3% |  |
| 3 | 732 | 9.0% |  |
| 2 | 1728 | 21.3% |  |

cap-surface  
Categorical

|  |  |
| --- | --- |
| Distinct count | 4 |
| Unique (%) | < 0.1% |
| Missing | 0 |
| Missing (%) | 0.0% |
| Memory size | 63.6 KiB |

|  |  |
| --- | --- |
| 2 | 3244 |
| 3 | 2556 |
| 0 | 2320 |
| 1 | 4 |

Toggle details

- Common Values
- Length
- Unicode

| Value | Count | Frequency (%) |  |
| --- | --- | --- | --- |
| 2 | 3244 | 39.9% |  |
| 3 | 2556 | 31.5% |  |
| 0 | 2320 | 28.6% |  |
| 1 | 4 | < 0.1% |  |

2020-08-25T01:41:33.060345image/svg+xmlMatplotlib v3.3.1, https://matplotlib.org/

Length

|  |  |
| --- | --- |
| Max length | 1 |
| Median length | 1 |
| Mean length | 1 |
| Min length | 1 |

- Overview
- Characters
- Categories
- Scripts
- Blocks

Overview of Unicode Properties

|  |  |
| --- | --- |
| Unique unicode characters | 4 |
| Unique unicode categories (?) | 1 |
| Unique unicode scripts (?) | 1 |
| Unique unicode blocks (?) | 1 |

The Unicode Standard assigns character properties to each code point, which can be used to analyse textual variables.

#### Most occurring characters

| Value | Count | Frequency (%) |  |
| --- | --- | --- | --- |
| 2 | 3244 | 39.9% |  |
| 3 | 2556 | 31.5% |  |
| 0 | 2320 | 28.6% |  |
| 1 | 4 | < 0.1% |  |

#### Most occurring categories

| Value | Count | Frequency (%) |  |
| --- | --- | --- | --- |
| Decimal Number | 8124 | 100.0% |  |

#### Most frequent Decimal Number characters

| Value | Count | Frequency (%) |  |
| --- | --- | --- | --- |
| 2 | 3244 | 39.9% |  |
| 3 | 2556 | 31.5% |  |
| 0 | 2320 | 28.6% |  |
| 1 | 4 | < 0.1% |  |

#### Most occurring scripts

| Value | Count | Frequency (%) |  |
| --- | --- | --- | --- |
| Common | 8124 | 100.0% |  |

#### Most frequent Common characters

| Value | Count | Frequency (%) |  |
| --- | --- | --- | --- |
| 2 | 3244 | 39.9% |  |
| 3 | 2556 | 31.5% |  |
| 0 | 2320 | 28.6% |  |
| 1 | 4 | < 0.1% |  |

#### Most occurring blocks

| Value | Count | Frequency (%) |  |
| --- | --- | --- | --- |
| ASCII | 8124 | 100.0% |  |

#### Most frequent ASCII characters

| Value | Count | Frequency (%) |  |
| --- | --- | --- | --- |
| 2 | 3244 | 39.9% |  |
| 3 | 2556 | 31.5% |  |
| 0 | 2320 | 28.6% |  |
| 1 | 4 | < 0.1% |  |

veil-type  
Boolean

`CONSTANT`  
`REJECTED`

|  |  |
| --- | --- |
| Distinct count | 1 |
| Unique (%) | < 0.1% |
| Missing | 0 |
| Missing (%) | 0.0% |
| Memory size | 63.6 KiB |

|  |  |
| --- | --- |
| 0 | 8124 |

Toggle details

- Frequency Table

| Value | Count | Frequency (%) |  |
| --- | --- | --- | --- |
| 0 | 8124 | 100.0% |  |

gill-attachment  
Boolean

|  |  |
| --- | --- |
| Distinct count | 2 |
| Unique (%) | < 0.1% |
| Missing | 0 |
| Missing (%) | 0.0% |
| Memory size | 63.6 KiB |

|  |  |
| --- | --- |
| 1 | 7914 |
| 0 | 210 |

Toggle details

- Frequency Table

| Value | Count | Frequency (%) |  |
| --- | --- | --- | --- |
| 1 | 7914 | 97.4% |  |
| 0 | 210 | 2.6% |  |

population  
Real number (ℝ≥0)

`ZEROS`

|  |  |
| --- | --- |
| Distinct count | 6 |
| Unique (%) | 0.1% |
| Missing | 0 |
| Missing (%) | 0.0% |
| Infinite | 0 |
| Infinite (%) | 0.0% |

|  |  |
| --- | --- |
| Mean | 3.6440177252584935 |
| Minimum | 0 |
| Maximum | 5 |
| Zeros | 384 |
| Zeros (%) | 4.7% |
| Memory size | 63.6 KiB |

2020-08-25T01:41:33.185771image/svg+xmlMatplotlib v3.3.1, https://matplotlib.org/

Toggle details

- Statistics
- Histogram(s)
- Common values
- Extreme values

Quantile statistics

|  |  |
| --- | --- |
| Minimum | 0 |
| 5-th percentile | 1 |
| Q1 | 3 |
| median | 4 |
| Q3 | 4 |
| 95-th percentile | 5 |
| Maximum | 5 |
| Range | 5 |
| Interquartile range (IQR) | 1 |

Descriptive statistics

|  |  |
| --- | --- |
| Standard deviation | 1.25208182 |
| Coefficient of variation (CV) | 0.3435992671 |
| Kurtosis | 1.676557918 |
| Mean | 3.644017725 |
| Median Absolute Deviation (MAD) | 1 |
| Skewness | -1.413095676 |
| Sum | 29604 |
| Variance | 1.567708884 |

- Histogram

2020-08-25T01:41:33.315385image/svg+xmlMatplotlib v3.3.1, https://matplotlib.org/ 

**Histogram with fixed size bins** (bins=10)

| Value | Count | Frequency (%) |  |
| --- | --- | --- | --- |
| 4 | 4040 | 49.7% |  |
| 5 | 1712 | 21.1% |  |
| 3 | 1248 | 15.4% |  |
| 2 | 400 | 4.9% |  |
| 0 | 384 | 4.7% |  |
| 1 | 340 | 4.2% |  |

- Minimum 5 values
- Maximum 5 values

| Value | Count | Frequency (%) |  |
| --- | --- | --- | --- |
| 0 | 384 | 4.7% |  |
| 1 | 340 | 4.2% |  |
| 2 | 400 | 4.9% |  |
| 3 | 1248 | 15.4% |  |
| 4 | 4040 | 49.7% |  |
| 5 | 1712 | 21.1% |  |

| Value | Count | Frequency (%) |  |
| --- | --- | --- | --- |
| 5 | 1712 | 21.1% |  |
| 4 | 4040 | 49.7% |  |
| 3 | 1248 | 15.4% |  |
| 2 | 400 | 4.9% |  |
| 1 | 340 | 4.2% |  |
| 0 | 384 | 4.7% |  |

stalk-surface-above-ring  
Categorical

|  |  |
| --- | --- |
| Distinct count | 4 |
| Unique (%) | < 0.1% |
| Missing | 0 |
| Missing (%) | 0.0% |
| Memory size | 63.6 KiB |

|  |  |
| --- | --- |
| 3 | 5176 |
| 2 | 2372 |
| 0 | 552 |
| 1 | 24 |

Toggle details

- Common Values
- Length
- Unicode

| Value | Count | Frequency (%) |  |
| --- | --- | --- | --- |
| 3 | 5176 | 63.7% |  |
| 2 | 2372 | 29.2% |  |
| 0 | 552 | 6.8% |  |
| 1 | 24 | 0.3% |  |

2020-08-25T01:41:33.494664image/svg+xmlMatplotlib v3.3.1, https://matplotlib.org/

Length

|  |  |
| --- | --- |
| Max length | 1 |
| Median length | 1 |
| Mean length | 1 |
| Min length | 1 |

- Overview
- Characters
- Categories
- Scripts
- Blocks

Overview of Unicode Properties

|  |  |
| --- | --- |
| Unique unicode characters | 4 |
| Unique unicode categories (?) | 1 |
| Unique unicode scripts (?) | 1 |
| Unique unicode blocks (?) | 1 |

The Unicode Standard assigns character properties to each code point, which can be used to analyse textual variables.

#### Most occurring characters

| Value | Count | Frequency (%) |  |
| --- | --- | --- | --- |
| 3 | 5176 | 63.7% |  |
| 2 | 2372 | 29.2% |  |
| 0 | 552 | 6.8% |  |
| 1 | 24 | 0.3% |  |

#### Most occurring categories

| Value | Count | Frequency (%) |  |
| --- | --- | --- | --- |
| Decimal Number | 8124 | 100.0% |  |

#### Most frequent Decimal Number characters

| Value | Count | Frequency (%) |  |
| --- | --- | --- | --- |
| 3 | 5176 | 63.7% |  |
| 2 | 2372 | 29.2% |  |
| 0 | 552 | 6.8% |  |
| 1 | 24 | 0.3% |  |

#### Most occurring scripts

| Value | Count | Frequency (%) |  |
| --- | --- | --- | --- |
| Common | 8124 | 100.0% |  |

#### Most frequent Common characters

| Value | Count | Frequency (%) |  |
| --- | --- | --- | --- |
| 3 | 5176 | 63.7% |  |
| 2 | 2372 | 29.2% |  |
| 0 | 552 | 6.8% |  |
| 1 | 24 | 0.3% |  |

#### Most occurring blocks

| Value | Count | Frequency (%) |  |
| --- | --- | --- | --- |
| ASCII | 8124 | 100.0% |  |

#### Most frequent ASCII characters

| Value | Count | Frequency (%) |  |
| --- | --- | --- | --- |
| 3 | 5176 | 63.7% |  |
| 2 | 2372 | 29.2% |  |
| 0 | 552 | 6.8% |  |
| 1 | 24 | 0.3% |  |

bruises?  
Boolean

|  |  |
| --- | --- |
| Distinct count | 2 |
| Unique (%) | < 0.1% |
| Missing | 0 |
| Missing (%) | 0.0% |
| Memory size | 63.6 KiB |

|  |  |
| --- | --- |
| 1 | 4748 |
| 0 | 3376 |

Toggle details

- Frequency Table

| Value | Count | Frequency (%) |  |
| --- | --- | --- | --- |
| 1 | 4748 | 58.4% |  |
| 0 | 3376 | 41.6% |  |

odor  
Real number (ℝ≥0)

`ZEROS`

|  |  |
| --- | --- |
| Distinct count | 9 |
| Unique (%) | 0.1% |
| Missing | 0 |
| Missing (%) | 0.0% |
| Infinite | 0 |
| Infinite (%) | 0.0% |

|  |  |
| --- | --- |
| Mean | 4.788281634662728 |
| Minimum | 0 |
| Maximum | 8 |
| Zeros | 400 |
| Zeros (%) | 4.9% |
| Memory size | 63.6 KiB |

2020-08-25T01:41:33.607694image/svg+xmlMatplotlib v3.3.1, https://matplotlib.org/

Toggle details

- Statistics
- Histogram(s)
- Common values
- Extreme values

Quantile statistics

|  |  |
| --- | --- |
| Minimum | 0 |
| 5-th percentile | 1 |
| Q1 | 4 |
| median | 6 |
| Q3 | 6 |
| 95-th percentile | 8 |
| Maximum | 8 |
| Range | 8 |
| Interquartile range (IQR) | 2 |

Descriptive statistics

|  |  |
| --- | --- |
| Standard deviation | 1.983678459 |
| Coefficient of variation (CV) | 0.4142777328 |
| Kurtosis | 0.07008745619 |
| Mean | 4.788281635 |
| Median Absolute Deviation (MAD) | 2 |
| Skewness | -0.726370513 |
| Sum | 38900 |
| Variance | 3.93498023 |

- Histogram

2020-08-25T01:41:33.728285image/svg+xmlMatplotlib v3.3.1, https://matplotlib.org/ 

**Histogram with fixed size bins** (bins=10)

| Value | Count | Frequency (%) |  |
| --- | --- | --- | --- |
| 6 | 3528 | 43.4% |  |
| 4 | 2160 | 26.6% |  |
| 3 | 576 | 7.1% |  |
| 8 | 576 | 7.1% |  |
| 1 | 400 | 4.9% |  |
| 0 | 400 | 4.9% |  |
| 7 | 256 | 3.2% |  |
| 2 | 192 | 2.4% |  |
| 5 | 36 | 0.4% |  |

- Minimum 5 values
- Maximum 5 values

| Value | Count | Frequency (%) |  |
| --- | --- | --- | --- |
| 0 | 400 | 4.9% |  |
| 1 | 400 | 4.9% |  |
| 2 | 192 | 2.4% |  |
| 3 | 576 | 7.1% |  |
| 4 | 2160 | 26.6% |  |
| 5 | 36 | 0.4% |  |
| 6 | 3528 | 43.4% |  |
| 7 | 256 | 3.2% |  |
| 8 | 576 | 7.1% |  |

| Value | Count | Frequency (%) |  |
| --- | --- | --- | --- |
| 8 | 576 | 7.1% |  |
| 7 | 256 | 3.2% |  |
| 6 | 3528 | 43.4% |  |
| 5 | 36 | 0.4% |  |
| 4 | 2160 | 26.6% |  |
| 3 | 576 | 7.1% |  |
| 2 | 192 | 2.4% |  |
| 1 | 400 | 4.9% |  |
| 0 | 400 | 4.9% |  |

ring-number  
Categorical

|  |  |
| --- | --- |
| Distinct count | 3 |
| Unique (%) | < 0.1% |
| Missing | 0 |
| Missing (%) | 0.0% |
| Memory size | 63.6 KiB |

|  |  |
| --- | --- |
| 1 | 7488 |
| 2 | 600 |
| 0 | 36 |

Toggle details

- Common Values
- Length
- Unicode

| Value | Count | Frequency (%) |  |
| --- | --- | --- | --- |
| 1 | 7488 | 92.2% |  |
| 2 | 600 | 7.4% |  |
| 0 | 36 | 0.4% |  |

2020-08-25T01:41:33.913423image/svg+xmlMatplotlib v3.3.1, https://matplotlib.org/

Length

|  |  |
| --- | --- |
| Max length | 1 |
| Median length | 1 |
| Mean length | 1 |
| Min length | 1 |

- Overview
- Characters
- Categories
- Scripts
- Blocks

Overview of Unicode Properties

|  |  |
| --- | --- |
| Unique unicode characters | 3 |
| Unique unicode categories (?) | 1 |
| Unique unicode scripts (?) | 1 |
| Unique unicode blocks (?) | 1 |

The Unicode Standard assigns character properties to each code point, which can be used to analyse textual variables.

#### Most occurring characters

| Value | Count | Frequency (%) |  |
| --- | --- | --- | --- |
| 1 | 7488 | 92.2% |  |
| 2 | 600 | 7.4% |  |
| 0 | 36 | 0.4% |  |

#### Most occurring categories

| Value | Count | Frequency (%) |  |
| --- | --- | --- | --- |
| Decimal Number | 8124 | 100.0% |  |

#### Most frequent Decimal Number characters

| Value | Count | Frequency (%) |  |
| --- | --- | --- | --- |
| 1 | 7488 | 92.2% |  |
| 2 | 600 | 7.4% |  |
| 0 | 36 | 0.4% |  |

#### Most occurring scripts

| Value | Count | Frequency (%) |  |
| --- | --- | --- | --- |
| Common | 8124 | 100.0% |  |

#### Most frequent Common characters

| Value | Count | Frequency (%) |  |
| --- | --- | --- | --- |
| 1 | 7488 | 92.2% |  |
| 2 | 600 | 7.4% |  |
| 0 | 36 | 0.4% |  |

#### Most occurring blocks

| Value | Count | Frequency (%) |  |
| --- | --- | --- | --- |
| ASCII | 8124 | 100.0% |  |

#### Most frequent ASCII characters

| Value | Count | Frequency (%) |  |
| --- | --- | --- | --- |
| 1 | 7488 | 92.2% |  |
| 2 | 600 | 7.4% |  |
| 0 | 36 | 0.4% |  |

stalk-surface-below-ring  
Categorical

|  |  |
| --- | --- |
| Distinct count | 4 |
| Unique (%) | < 0.1% |
| Missing | 0 |
| Missing (%) | 0.0% |
| Memory size | 63.6 KiB |

|  |  |
| --- | --- |
| 3 | 4936 |
| 2 | 2304 |
| 0 | 600 |
| 1 | 284 |

Toggle details

- Common Values
- Length
- Unicode

| Value | Count | Frequency (%) |  |
| --- | --- | --- | --- |
| 3 | 4936 | 60.8% |  |
| 2 | 2304 | 28.4% |  |
| 0 | 600 | 7.4% |  |
| 1 | 284 | 3.5% |  |

2020-08-25T01:41:34.086741image/svg+xmlMatplotlib v3.3.1, https://matplotlib.org/

Length

|  |  |
| --- | --- |
| Max length | 1 |
| Median length | 1 |
| Mean length | 1 |
| Min length | 1 |

- Overview
- Characters
- Categories
- Scripts
- Blocks

Overview of Unicode Properties

|  |  |
| --- | --- |
| Unique unicode characters | 4 |
| Unique unicode categories (?) | 1 |
| Unique unicode scripts (?) | 1 |
| Unique unicode blocks (?) | 1 |

The Unicode Standard assigns character properties to each code point, which can be used to analyse textual variables.

#### Most occurring characters

| Value | Count | Frequency (%) |  |
| --- | --- | --- | --- |
| 3 | 4936 | 60.8% |  |
| 2 | 2304 | 28.4% |  |
| 0 | 600 | 7.4% |  |
| 1 | 284 | 3.5% |  |

#### Most occurring categories

| Value | Count | Frequency (%) |  |
| --- | --- | --- | --- |
| Decimal Number | 8124 | 100.0% |  |

#### Most frequent Decimal Number characters

| Value | Count | Frequency (%) |  |
| --- | --- | --- | --- |
| 3 | 4936 | 60.8% |  |
| 2 | 2304 | 28.4% |  |
| 0 | 600 | 7.4% |  |
| 1 | 284 | 3.5% |  |

#### Most occurring scripts

| Value | Count | Frequency (%) |  |
| --- | --- | --- | --- |
| Common | 8124 | 100.0% |  |

#### Most frequent Common characters

| Value | Count | Frequency (%) |  |
| --- | --- | --- | --- |
| 3 | 4936 | 60.8% |  |
| 2 | 2304 | 28.4% |  |
| 0 | 600 | 7.4% |  |
| 1 | 284 | 3.5% |  |

#### Most occurring blocks

| Value | Count | Frequency (%) |  |
| --- | --- | --- | --- |
| ASCII | 8124 | 100.0% |  |

#### Most frequent ASCII characters

| Value | Count | Frequency (%) |  |
| --- | --- | --- | --- |
| 3 | 4936 | 60.8% |  |
| 2 | 2304 | 28.4% |  |
| 0 | 600 | 7.4% |  |
| 1 | 284 | 3.5% |  |

ring-type  
Real number (ℝ≥0)

`ZEROS`

|  |  |
| --- | --- |
| Distinct count | 5 |
| Unique (%) | 0.1% |
| Missing | 0 |
| Missing (%) | 0.0% |
| Infinite | 0 |
| Infinite (%) | 0.0% |

|  |  |
| --- | --- |
| Mean | 2.291974396848843 |
| Minimum | 0 |
| Maximum | 4 |
| Zeros | 2776 |
| Zeros (%) | 34.2% |
| Memory size | 63.6 KiB |

2020-08-25T01:41:34.202621image/svg+xmlMatplotlib v3.3.1, https://matplotlib.org/

Toggle details

- Statistics
- Histogram(s)
- Common values
- Extreme values

Quantile statistics

|  |  |
| --- | --- |
| Minimum | 0 |
| 5-th percentile | 0 |
| Q1 | 0 |
| median | 2 |
| Q3 | 4 |
| 95-th percentile | 4 |
| Maximum | 4 |
| Range | 4 |
| Interquartile range (IQR) | 4 |

Descriptive statistics

|  |  |
| --- | --- |
| Standard deviation | 1.801672002 |
| Coefficient of variation (CV) | 0.7860785899 |
| Kurtosis | -1.708767354 |
| Mean | 2.291974397 |
| Median Absolute Deviation (MAD) | 2 |
| Skewness | -0.2900182044 |
| Sum | 18620 |
| Variance | 3.246022003 |

- Histogram

2020-08-25T01:41:34.317686image/svg+xmlMatplotlib v3.3.1, https://matplotlib.org/ 

**Histogram with fixed size bins** (bins=10)

| Value | Count | Frequency (%) |  |
| --- | --- | --- | --- |
| 4 | 3968 | 48.8% |  |
| 0 | 2776 | 34.2% |  |
| 2 | 1296 | 16.0% |  |
| 1 | 48 | 0.6% |  |
| 3 | 36 | 0.4% |  |

- Minimum 5 values
- Maximum 5 values

| Value | Count | Frequency (%) |  |
| --- | --- | --- | --- |
| 0 | 2776 | 34.2% |  |
| 1 | 48 | 0.6% |  |
| 2 | 1296 | 16.0% |  |
| 3 | 36 | 0.4% |  |
| 4 | 3968 | 48.8% |  |

| Value | Count | Frequency (%) |  |
| --- | --- | --- | --- |
| 4 | 3968 | 48.8% |  |
| 3 | 36 | 0.4% |  |
| 2 | 1296 | 16.0% |  |
| 1 | 48 | 0.6% |  |
| 0 | 2776 | 34.2% |  |

veil-color  
Categorical

|  |  |
| --- | --- |
| Distinct count | 4 |
| Unique (%) | < 0.1% |
| Missing | 0 |
| Missing (%) | 0.0% |
| Memory size | 63.6 KiB |

|  |  |
| --- | --- |
| 2 | 7924 |
| 1 | 96 |
| 0 | 96 |
| 3 | 8 |

Toggle details

- Common Values
- Length
- Unicode

| Value | Count | Frequency (%) |  |
| --- | --- | --- | --- |
| 2 | 7924 | 97.5% |  |
| 1 | 96 | 1.2% |  |
| 0 | 96 | 1.2% |  |
| 3 | 8 | 0.1% |  |

2020-08-25T01:41:34.504522image/svg+xmlMatplotlib v3.3.1, https://matplotlib.org/

Length

|  |  |
| --- | --- |
| Max length | 1 |
| Median length | 1 |
| Mean length | 1 |
| Min length | 1 |

- Overview
- Characters
- Categories
- Scripts
- Blocks

Overview of Unicode Properties

|  |  |
| --- | --- |
| Unique unicode characters | 4 |
| Unique unicode categories (?) | 1 |
| Unique unicode scripts (?) | 1 |
| Unique unicode blocks (?) | 1 |

The Unicode Standard assigns character properties to each code point, which can be used to analyse textual variables.

#### Most occurring characters

| Value | Count | Frequency (%) |  |
| --- | --- | --- | --- |
| 2 | 7924 | 97.5% |  |
| 0 | 96 | 1.2% |  |
| 1 | 96 | 1.2% |  |
| 3 | 8 | 0.1% |  |

#### Most occurring categories

| Value | Count | Frequency (%) |  |
| --- | --- | --- | --- |
| Decimal Number | 8124 | 100.0% |  |

#### Most frequent Decimal Number characters

| Value | Count | Frequency (%) |  |
| --- | --- | --- | --- |
| 2 | 7924 | 97.5% |  |
| 0 | 96 | 1.2% |  |
| 1 | 96 | 1.2% |  |
| 3 | 8 | 0.1% |  |

#### Most occurring scripts

| Value | Count | Frequency (%) |  |
| --- | --- | --- | --- |
| Common | 8124 | 100.0% |  |

#### Most frequent Common characters

| Value | Count | Frequency (%) |  |
| --- | --- | --- | --- |
| 2 | 7924 | 97.5% |  |
| 0 | 96 | 1.2% |  |
| 1 | 96 | 1.2% |  |
| 3 | 8 | 0.1% |  |

#### Most occurring blocks

| Value | Count | Frequency (%) |  |
| --- | --- | --- | --- |
| ASCII | 8124 | 100.0% |  |

#### Most frequent ASCII characters

| Value | Count | Frequency (%) |  |
| --- | --- | --- | --- |
| 2 | 7924 | 97.5% |  |
| 0 | 96 | 1.2% |  |
| 1 | 96 | 1.2% |  |
| 3 | 8 | 0.1% |  |

cap-color  
Real number (ℝ≥0)

`ZEROS`

|  |  |
| --- | --- |
| Distinct count | 10 |
| Unique (%) | 0.1% |
| Missing | 0 |
| Missing (%) | 0.0% |
| Infinite | 0 |
| Infinite (%) | 0.0% |

|  |  |
| --- | --- |
| Mean | 4.323485967503693 |
| Minimum | 0 |
| Maximum | 9 |
| Zeros | 2284 |
| Zeros (%) | 28.1% |
| Memory size | 63.6 KiB |

2020-08-25T01:41:34.616155image/svg+xmlMatplotlib v3.3.1, https://matplotlib.org/

Toggle details

- Statistics
- Histogram(s)
- Common values
- Extreme values

Quantile statistics

|  |  |
| --- | --- |
| Minimum | 0 |
| 5-th percentile | 0 |
| Q1 | 0 |
| median | 3 |
| Q3 | 8 |
| 95-th percentile | 9 |
| Maximum | 9 |
| Range | 9 |
| Interquartile range (IQR) | 8 |

Descriptive statistics

|  |  |
| --- | --- |
| Standard deviation | 3.444390875 |
| Coefficient of variation (CV) | 0.7966698401 |
| Kurtosis | -1.595954042 |
| Mean | 4.323485968 |
| Median Absolute Deviation (MAD) | 3 |
| Skewness | -0.01618156112 |
| Sum | 35124 |
| Variance | 11.8638285 |

- Histogram

2020-08-25T01:41:34.719686image/svg+xmlMatplotlib v3.3.1, https://matplotlib.org/ 

**Histogram with fixed size bins** (bins=10)

| Value | Count | Frequency (%) |  |
| --- | --- | --- | --- |
| 0 | 2284 | 28.1% |  |
| 3 | 1840 | 22.6% |  |
| 7 | 1500 | 18.5% |  |
| 9 | 1072 | 13.2% |  |
| 8 | 1040 | 12.8% |  |
| 1 | 168 | 2.1% |  |
| 5 | 144 | 1.8% |  |
| 2 | 44 | 0.5% |  |
| 6 | 16 | 0.2% |  |
| 4 | 16 | 0.2% |  |

- Minimum 5 values
- Maximum 5 values

| Value | Count | Frequency (%) |  |
| --- | --- | --- | --- |
| 0 | 2284 | 28.1% |  |
| 1 | 168 | 2.1% |  |
| 2 | 44 | 0.5% |  |
| 3 | 1840 | 22.6% |  |
| 4 | 16 | 0.2% |  |
| 5 | 144 | 1.8% |  |
| 6 | 16 | 0.2% |  |
| 7 | 1500 | 18.5% |  |
| 8 | 1040 | 12.8% |  |
| 9 | 1072 | 13.2% |  |

| Value | Count | Frequency (%) |  |
| --- | --- | --- | --- |
| 9 | 1072 | 13.2% |  |
| 8 | 1040 | 12.8% |  |
| 7 | 1500 | 18.5% |  |
| 6 | 16 | 0.2% |  |
| 5 | 144 | 1.8% |  |
| 4 | 16 | 0.2% |  |
| 3 | 1840 | 22.6% |  |
| 2 | 44 | 0.5% |  |
| 1 | 168 | 2.1% |  |
| 0 | 2284 | 28.1% |  |

stalk-shape  
Boolean

|  |  |
| --- | --- |
| Distinct count | 2 |
| Unique (%) | < 0.1% |
| Missing | 0 |
| Missing (%) | 0.0% |
| Memory size | 63.6 KiB |

|  |  |
| --- | --- |
| 1 | 4608 |
| 0 | 3516 |

Toggle details

- Frequency Table

| Value | Count | Frequency (%) |  |
| --- | --- | --- | --- |
| 1 | 4608 | 56.7% |  |
| 0 | 3516 | 43.3% |  |

habitat  
Real number (ℝ≥0)

`ZEROS`

|  |  |
| --- | --- |
| Distinct count | 7 |
| Unique (%) | 0.1% |
| Missing | 0 |
| Missing (%) | 0.0% |
| Infinite | 0 |
| Infinite (%) | 0.0% |

|  |  |
| --- | --- |
| Mean | 3.2210733628754307 |
| Minimum | 0 |
| Maximum | 6 |
| Zeros | 2148 |
| Zeros (%) | 26.4% |
| Memory size | 63.6 KiB |

2020-08-25T01:41:35.011023image/svg+xmlMatplotlib v3.3.1, https://matplotlib.org/

Toggle details

- Statistics
- Histogram(s)
- Common values
- Extreme values

Quantile statistics

|  |  |
| --- | --- |
| Minimum | 0 |
| 5-th percentile | 0 |
| Q1 | 0 |
| median | 3 |
| Q3 | 6 |
| 95-th percentile | 6 |
| Maximum | 6 |
| Range | 6 |
| Interquartile range (IQR) | 6 |

Descriptive statistics

|  |  |
| --- | --- |
| Standard deviation | 2.530691874 |
| Coefficient of variation (CV) | 0.785667257 |
| Kurtosis | -1.673747886 |
| Mean | 3.221073363 |
| Median Absolute Deviation (MAD) | 3 |
| Skewness | -0.09599061819 |
| Sum | 26168 |
| Variance | 6.404401359 |

- Histogram

2020-08-25T01:41:35.121950image/svg+xmlMatplotlib v3.3.1, https://matplotlib.org/ 

**Histogram with fixed size bins** (bins=10)

| Value | Count | Frequency (%) |  |
| --- | --- | --- | --- |
| 6 | 3148 | 38.7% |  |
| 0 | 2148 | 26.4% |  |
| 3 | 1144 | 14.1% |  |
| 1 | 832 | 10.2% |  |
| 4 | 368 | 4.5% |  |
| 2 | 292 | 3.6% |  |
| 5 | 192 | 2.4% |  |

- Minimum 5 values
- Maximum 5 values

| Value | Count | Frequency (%) |  |
| --- | --- | --- | --- |
| 0 | 2148 | 26.4% |  |
| 1 | 832 | 10.2% |  |
| 2 | 292 | 3.6% |  |
| 3 | 1144 | 14.1% |  |
| 4 | 368 | 4.5% |  |
| 5 | 192 | 2.4% |  |
| 6 | 3148 | 38.7% |  |

| Value | Count | Frequency (%) |  |
| --- | --- | --- | --- |
| 6 | 3148 | 38.7% |  |
| 5 | 192 | 2.4% |  |
| 4 | 368 | 4.5% |  |
| 3 | 1144 | 14.1% |  |
| 2 | 292 | 3.6% |  |
| 1 | 832 | 10.2% |  |
| 0 | 2148 | 26.4% |  |

gill-size  
Boolean

|  |  |
| --- | --- |
| Distinct count | 2 |
| Unique (%) | < 0.1% |
| Missing | 0 |
| Missing (%) | 0.0% |
| Memory size | 63.6 KiB |

|  |  |
| --- | --- |
| 0 | 5612 |
| 1 | 2512 |

Toggle details

- Frequency Table

| Value | Count | Frequency (%) |  |
| --- | --- | --- | --- |
| 0 | 5612 | 69.1% |  |
| 1 | 2512 | 30.9% |  |

stalk-root  
Real number (ℝ≥0)

`ZEROS`

|  |  |
| --- | --- |
| Distinct count | 5 |
| Unique (%) | 0.1% |
| Missing | 0 |
| Missing (%) | 0.0% |
| Infinite | 0 |
| Infinite (%) | 0.0% |

|  |  |
| --- | --- |
| Mean | 1.1097981290004924 |
| Minimum | 0 |
| Maximum | 4 |
| Zeros | 2480 |
| Zeros (%) | 30.5% |
| Memory size | 63.6 KiB |

2020-08-25T01:41:35.236572image/svg+xmlMatplotlib v3.3.1, https://matplotlib.org/

Toggle details

- Statistics
- Histogram(s)
- Common values
- Extreme values

Quantile statistics

|  |  |
| --- | --- |
| Minimum | 0 |
| 5-th percentile | 0 |
| Q1 | 0 |
| median | 1 |
| Q3 | 1 |
| 95-th percentile | 3 |
| Maximum | 4 |
| Range | 4 |
| Interquartile range (IQR) | 1 |

Descriptive statistics

|  |  |
| --- | --- |
| Standard deviation | 1.061106068 |
| Coefficient of variation (CV) | 0.9561252991 |
| Kurtosis | 0.08976099331 |
| Mean | 1.109798129 |
| Median Absolute Deviation (MAD) | 1 |
| Skewness | 0.9478523612 |
| Sum | 9016 |
| Variance | 1.125946088 |

- Histogram

2020-08-25T01:41:35.349949image/svg+xmlMatplotlib v3.3.1, https://matplotlib.org/ 

**Histogram with fixed size bins** (bins=10)

| Value | Count | Frequency (%) |  |
| --- | --- | --- | --- |
| 1 | 3776 | 46.5% |  |
| 0 | 2480 | 30.5% |  |
| 3 | 1120 | 13.8% |  |
| 2 | 556 | 6.8% |  |
| 4 | 192 | 2.4% |  |

- Minimum 5 values
- Maximum 5 values

| Value | Count | Frequency (%) |  |
| --- | --- | --- | --- |
| 0 | 2480 | 30.5% |  |
| 1 | 3776 | 46.5% |  |
| 2 | 556 | 6.8% |  |
| 3 | 1120 | 13.8% |  |
| 4 | 192 | 2.4% |  |

| Value | Count | Frequency (%) |  |
| --- | --- | --- | --- |
| 4 | 192 | 2.4% |  |
| 3 | 1120 | 13.8% |  |
| 2 | 556 | 6.8% |  |
| 1 | 3776 | 46.5% |  |
| 0 | 2480 | 30.5% |  |

target  
Boolean

|  |  |
| --- | --- |
| Distinct count | 2 |
| Unique (%) | < 0.1% |
| Missing | 0 |
| Missing (%) | 0.0% |
| Memory size | 63.6 KiB |

|  |  |
| --- | --- |
| 0 | 4208 |
| 1 | 3916 |

Toggle details

- Frequency Table

| Value | Count | Frequency (%) |  |
| --- | --- | --- | --- |
| 0 | 4208 | 51.8% |  |
| 1 | 3916 | 48.2% |  |

# Interactions

- cap-shape
- stalk-color-above-ring
- gill-color
- population
- odor
- ring-type
- cap-color
- habitat
- stalk-root

- cap-shape
- stalk-color-above-ring
- gill-color
- population
- odor
- ring-type
- cap-color
- habitat
- stalk-root

2020-08-25T01:41:18.732626image/svg+xmlMatplotlib v3.3.1, https://matplotlib.org/

2020-08-25T01:41:18.867875image/svg+xmlMatplotlib v3.3.1, https://matplotlib.org/

2020-08-25T01:41:19.027836image/svg+xmlMatplotlib v3.3.1, https://matplotlib.org/

2020-08-25T01:41:19.165420image/svg+xmlMatplotlib v3.3.1, https://matplotlib.org/

2020-08-25T01:41:19.300133image/svg+xmlMatplotlib v3.3.1, https://matplotlib.org/

2020-08-25T01:41:19.445646image/svg+xmlMatplotlib v3.3.1, https://matplotlib.org/

2020-08-25T01:41:19.597286image/svg+xmlMatplotlib v3.3.1, https://matplotlib.org/

2020-08-25T01:41:19.741267image/svg+xmlMatplotlib v3.3.1, https://matplotlib.org/

2020-08-25T01:41:19.881032image/svg+xmlMatplotlib v3.3.1, https://matplotlib.org/

- cap-shape
- stalk-color-above-ring
- gill-color
- population
- odor
- ring-type
- cap-color
- habitat
- stalk-root

2020-08-25T01:41:20.030314image/svg+xmlMatplotlib v3.3.1, https://matplotlib.org/

2020-08-25T01:41:20.182523image/svg+xmlMatplotlib v3.3.1, https://matplotlib.org/

2020-08-25T01:41:20.345280image/svg+xmlMatplotlib v3.3.1, https://matplotlib.org/

2020-08-25T01:41:20.495949image/svg+xmlMatplotlib v3.3.1, https://matplotlib.org/

2020-08-25T01:41:20.646612image/svg+xmlMatplotlib v3.3.1, https://matplotlib.org/

2020-08-25T01:41:20.805600image/svg+xmlMatplotlib v3.3.1, https://matplotlib.org/

2020-08-25T01:41:20.969018image/svg+xmlMatplotlib v3.3.1, https://matplotlib.org/

2020-08-25T01:41:21.292727image/svg+xmlMatplotlib v3.3.1, https://matplotlib.org/

2020-08-25T01:41:21.448581image/svg+xmlMatplotlib v3.3.1, https://matplotlib.org/

- cap-shape
- stalk-color-above-ring
- gill-color
- population
- odor
- ring-type
- cap-color
- habitat
- stalk-root

2020-08-25T01:41:21.615740image/svg+xmlMatplotlib v3.3.1, https://matplotlib.org/

2020-08-25T01:41:21.750206image/svg+xmlMatplotlib v3.3.1, https://matplotlib.org/

2020-08-25T01:41:21.897818image/svg+xmlMatplotlib v3.3.1, https://matplotlib.org/

2020-08-25T01:41:22.039988image/svg+xmlMatplotlib v3.3.1, https://matplotlib.org/

2020-08-25T01:41:22.177739image/svg+xmlMatplotlib v3.3.1, https://matplotlib.org/

2020-08-25T01:41:22.322133image/svg+xmlMatplotlib v3.3.1, https://matplotlib.org/

2020-08-25T01:41:22.470347image/svg+xmlMatplotlib v3.3.1, https://matplotlib.org/

2020-08-25T01:41:22.603996image/svg+xmlMatplotlib v3.3.1, https://matplotlib.org/

2020-08-25T01:41:22.743954image/svg+xmlMatplotlib v3.3.1, https://matplotlib.org/

- cap-shape
- stalk-color-above-ring
- gill-color
- population
- odor
- ring-type
- cap-color
- habitat
- stalk-root

2020-08-25T01:41:22.894742image/svg+xmlMatplotlib v3.3.1, https://matplotlib.org/

2020-08-25T01:41:23.029819image/svg+xmlMatplotlib v3.3.1, https://matplotlib.org/

2020-08-25T01:41:23.188606image/svg+xmlMatplotlib v3.3.1, https://matplotlib.org/

2020-08-25T01:41:23.328473image/svg+xmlMatplotlib v3.3.1, https://matplotlib.org/

2020-08-25T01:41:23.465698image/svg+xmlMatplotlib v3.3.1, https://matplotlib.org/

2020-08-25T01:41:23.613042image/svg+xmlMatplotlib v3.3.1, https://matplotlib.org/

2020-08-25T01:41:23.762012image/svg+xmlMatplotlib v3.3.1, https://matplotlib.org/

2020-08-25T01:41:23.906037image/svg+xmlMatplotlib v3.3.1, https://matplotlib.org/

2020-08-25T01:41:24.045668image/svg+xmlMatplotlib v3.3.1, https://matplotlib.org/

- cap-shape
- stalk-color-above-ring
- gill-color
- population
- odor
- ring-type
- cap-color
- habitat
- stalk-root

2020-08-25T01:41:24.200142image/svg+xmlMatplotlib v3.3.1, https://matplotlib.org/

2020-08-25T01:41:24.348968image/svg+xmlMatplotlib v3.3.1, https://matplotlib.org/

2020-08-25T01:41:24.512018image/svg+xmlMatplotlib v3.3.1, https://matplotlib.org/

2020-08-25T01:41:24.660836image/svg+xmlMatplotlib v3.3.1, https://matplotlib.org/

2020-08-25T01:41:24.809488image/svg+xmlMatplotlib v3.3.1, https://matplotlib.org/

2020-08-25T01:41:24.966622image/svg+xmlMatplotlib v3.3.1, https://matplotlib.org/

2020-08-25T01:41:25.128654image/svg+xmlMatplotlib v3.3.1, https://matplotlib.org/

2020-08-25T01:41:25.271921image/svg+xmlMatplotlib v3.3.1, https://matplotlib.org/

2020-08-25T01:41:25.430999image/svg+xmlMatplotlib v3.3.1, https://matplotlib.org/

- cap-shape
- stalk-color-above-ring
- gill-color
- population
- odor
- ring-type
- cap-color
- habitat
- stalk-root

2020-08-25T01:41:25.592016image/svg+xmlMatplotlib v3.3.1, https://matplotlib.org/

2020-08-25T01:41:25.742192image/svg+xmlMatplotlib v3.3.1, https://matplotlib.org/

2020-08-25T01:41:26.102466image/svg+xmlMatplotlib v3.3.1, https://matplotlib.org/

2020-08-25T01:41:26.255007image/svg+xmlMatplotlib v3.3.1, https://matplotlib.org/

2020-08-25T01:41:26.406621image/svg+xmlMatplotlib v3.3.1, https://matplotlib.org/

2020-08-25T01:41:26.573022image/svg+xmlMatplotlib v3.3.1, https://matplotlib.org/

2020-08-25T01:41:26.741078image/svg+xmlMatplotlib v3.3.1, https://matplotlib.org/

2020-08-25T01:41:26.888725image/svg+xmlMatplotlib v3.3.1, https://matplotlib.org/

2020-08-25T01:41:27.042659image/svg+xmlMatplotlib v3.3.1, https://matplotlib.org/

- cap-shape
- stalk-color-above-ring
- gill-color
- population
- odor
- ring-type
- cap-color
- habitat
- stalk-root

2020-08-25T01:41:27.208785image/svg+xmlMatplotlib v3.3.1, https://matplotlib.org/

2020-08-25T01:41:27.336687image/svg+xmlMatplotlib v3.3.1, https://matplotlib.org/

2020-08-25T01:41:27.477927image/svg+xmlMatplotlib v3.3.1, https://matplotlib.org/

2020-08-25T01:41:27.606698image/svg+xmlMatplotlib v3.3.1, https://matplotlib.org/

2020-08-25T01:41:27.739995image/svg+xmlMatplotlib v3.3.1, https://matplotlib.org/

2020-08-25T01:41:27.878596image/svg+xmlMatplotlib v3.3.1, https://matplotlib.org/

2020-08-25T01:41:28.020481image/svg+xmlMatplotlib v3.3.1, https://matplotlib.org/

2020-08-25T01:41:28.148939image/svg+xmlMatplotlib v3.3.1, https://matplotlib.org/

2020-08-25T01:41:28.282087image/svg+xmlMatplotlib v3.3.1, https://matplotlib.org/

- cap-shape
- stalk-color-above-ring
- gill-color
- population
- odor
- ring-type
- cap-color
- habitat
- stalk-root

2020-08-25T01:41:28.428264image/svg+xmlMatplotlib v3.3.1, https://matplotlib.org/

2020-08-25T01:41:28.570873image/svg+xmlMatplotlib v3.3.1, https://matplotlib.org/

2020-08-25T01:41:28.739477image/svg+xmlMatplotlib v3.3.1, https://matplotlib.org/

2020-08-25T01:41:28.887257image/svg+xmlMatplotlib v3.3.1, https://matplotlib.org/

2020-08-25T01:41:29.031051image/svg+xmlMatplotlib v3.3.1, https://matplotlib.org/

2020-08-25T01:41:29.185511image/svg+xmlMatplotlib v3.3.1, https://matplotlib.org/

2020-08-25T01:41:29.337970image/svg+xmlMatplotlib v3.3.1, https://matplotlib.org/

2020-08-25T01:41:29.478003image/svg+xmlMatplotlib v3.3.1, https://matplotlib.org/

2020-08-25T01:41:29.620275image/svg+xmlMatplotlib v3.3.1, https://matplotlib.org/

- cap-shape
- stalk-color-above-ring
- gill-color
- population
- odor
- ring-type
- cap-color
- habitat
- stalk-root

2020-08-25T01:41:29.781598image/svg+xmlMatplotlib v3.3.1, https://matplotlib.org/

2020-08-25T01:41:29.932254image/svg+xmlMatplotlib v3.3.1, https://matplotlib.org/

2020-08-25T01:41:30.107904image/svg+xmlMatplotlib v3.3.1, https://matplotlib.org/

2020-08-25T01:41:30.269311image/svg+xmlMatplotlib v3.3.1, https://matplotlib.org/

2020-08-25T01:41:30.421297image/svg+xmlMatplotlib v3.3.1, https://matplotlib.org/

2020-08-25T01:41:30.580618image/svg+xmlMatplotlib v3.3.1, https://matplotlib.org/

2020-08-25T01:41:30.938373image/svg+xmlMatplotlib v3.3.1, https://matplotlib.org/

2020-08-25T01:41:31.085606image/svg+xmlMatplotlib v3.3.1, https://matplotlib.org/

2020-08-25T01:41:31.241278image/svg+xmlMatplotlib v3.3.1, https://matplotlib.org/

# Correlations

Toggle correlation descriptions

- Pearson's r
- Spearman's ρ
- Kendall's τ
- Phik (φk)
- Cramér's V (φc)

2020-08-25T01:41:35.517045image/svg+xmlMatplotlib v3.3.1, https://matplotlib.org/

### Pearson's r

The Pearson's correlation coefficient (*r*) is a measure of linear correlation between two variables. It's value lies between -1 and +1, -1 indicating total negative linear correlation, 0 indicating no linear correlation and 1 indicating total positive linear correlation. Furthermore, *r* is invariant under separate changes in location and scale of the two variables, implying that for a linear function the angle to the x-axis does not affect *r*.  
  
To calculate *r* for two variables *X* and *Y*, one divides the covariance of *X* and *Y* by the product of their standard deviations.

2020-08-25T01:41:35.869439image/svg+xmlMatplotlib v3.3.1, https://matplotlib.org/

### Spearman's ρ

The Spearman's rank correlation coefficient (*ρ*) is a measure of monotonic correlation between two variables, and is therefore better in catching nonlinear monotonic correlations than Pearson's *r*. It's value lies between -1 and +1, -1 indicating total negative monotonic correlation, 0 indicating no monotonic correlation and 1 indicating total positive monotonic correlation.  
  
To calculate *ρ* for two variables *X* and *Y*, one divides the covariance of the rank variables of *X* and *Y* by the product of their standard deviations.

2020-08-25T01:41:36.222016image/svg+xmlMatplotlib v3.3.1, https://matplotlib.org/

### Kendall's τ

Similarly to Spearman's rank correlation coefficient, the Kendall rank correlation coefficient (*τ*) measures ordinal association between two variables. It's value lies between -1 and +1, -1 indicating total negative correlation, 0 indicating no correlation and 1 indicating total positive correlation.   
  
To calculate *τ* for two variables *X* and *Y*, one determines the number of concordant and discordant pairs of observations. *τ* is given by the number of concordant pairs minus the discordant pairs divided by the total number of pairs.

2020-08-25T01:41:36.585599image/svg+xmlMatplotlib v3.3.1, https://matplotlib.org/

### Phik (φk)

Phik (φk) is a new and practical correlation coefficient that works consistently between categorical, ordinal and interval variables, captures non-linear dependency and reverts to the Pearson correlation coefficient in case of a bivariate normal input distribution. There is extensive documentation available here.

2020-08-25T01:41:36.882782image/svg+xmlMatplotlib v3.3.1, https://matplotlib.org/

### Cramér's V (φc)

Cramér's V is an association measure for nominal random variables. The coefficient ranges from 0 to 1, with 0 indicating independence and 1 indicating perfect association. The empirical estimators used for Cramér's V have been proved to be biased, even for large samples. We use a bias-corrected measure that has been proposed by Bergsma in 2013 that can be found here.

# Missing values

- Count
- Matrix

2020-08-25T01:41:31.559244image/svg+xmlMatplotlib v3.3.1, https://matplotlib.org/

2020-08-25T01:41:32.065468image/svg+xmlMatplotlib v3.3.1, https://matplotlib.org/

# Sample

## First rows

|  | cap-shape | stalk-color-above-ring | gill-color | cap-surface | veil-type | gill-attachment | population | stalk-surface-above-ring | bruises? | odor | ring-number | stalk-surface-below-ring | ring-type | veil-color | cap-color | stalk-shape | habitat | gill-size | stalk-root | target |
| --- | --- | --- | --- | --- | --- | --- | --- | --- | --- | --- | --- | --- | --- | --- | --- | --- | --- | --- | --- | --- |
| 0 | 2 | 3 | 8 | 0 | 0 | 1 | 4 | 3 | 0 | 6 | 1 | 3 | 4 | 2 | 7 | 1 | 6 | 0 | 1 | 0 |
| 1 | 0 | 7 | 4 | 3 | 0 | 1 | 2 | 3 | 0 | 0 | 1 | 3 | 4 | 2 | 9 | 0 | 0 | 0 | 2 | 0 |
| 2 | 2 | 7 | 0 | 3 | 0 | 1 | 3 | 0 | 1 | 6 | 1 | 3 | 0 | 2 | 8 | 1 | 0 | 0 | 3 | 0 |
| 3 | 2 | 0 | 4 | 2 | 0 | 1 | 4 | 2 | 1 | 4 | 1 | 2 | 2 | 2 | 3 | 0 | 3 | 0 | 1 | 1 |
| 4 | 3 | 7 | 8 | 0 | 0 | 1 | 5 | 3 | 0 | 6 | 1 | 3 | 4 | 2 | 0 | 1 | 6 | 0 | 1 | 0 |
| 5 | 2 | 7 | 0 | 2 | 0 | 1 | 4 | 3 | 0 | 7 | 1 | 3 | 4 | 2 | 8 | 0 | 4 | 1 | 3 | 1 |
| 6 | 4 | 7 | 2 | 3 | 0 | 1 | 4 | 3 | 1 | 8 | 1 | 3 | 0 | 2 | 0 | 1 | 1 | 1 | 0 | 1 |
| 7 | 3 | 6 | 10 | 2 | 0 | 1 | 1 | 3 | 0 | 6 | 2 | 3 | 0 | 2 | 5 | 0 | 5 | 0 | 0 | 0 |
| 8 | 2 | 0 | 4 | 0 | 0 | 1 | 5 | 2 | 1 | 4 | 1 | 2 | 2 | 2 | 9 | 0 | 3 | 0 | 1 | 1 |
| 9 | 3 | 5 | 4 | 0 | 0 | 1 | 4 | 2 | 1 | 4 | 1 | 2 | 2 | 2 | 3 | 0 | 0 | 0 | 1 | 1 |

## Last rows

|  | cap-shape | stalk-color-above-ring | gill-color | cap-surface | veil-type | gill-attachment | population | stalk-surface-above-ring | bruises? | odor | ring-number | stalk-surface-below-ring | ring-type | veil-color | cap-color | stalk-shape | habitat | gill-size | stalk-root | target |
| --- | --- | --- | --- | --- | --- | --- | --- | --- | --- | --- | --- | --- | --- | --- | --- | --- | --- | --- | --- | --- |
| 8114 | 2 | 7 | 1 | 0 | 0 | 1 | 0 | 0 | 1 | 6 | 1 | 0 | 0 | 2 | 3 | 1 | 0 | 0 | 3 | 0 |
| 8115 | 3 | 5 | 7 | 2 | 0 | 1 | 4 | 2 | 1 | 4 | 1 | 2 | 2 | 2 | 9 | 0 | 0 | 0 | 1 | 1 |
| 8116 | 2 | 7 | 1 | 2 | 0 | 1 | 5 | 3 | 0 | 1 | 1 | 1 | 4 | 2 | 0 | 0 | 3 | 0 | 4 | 0 |
| 8117 | 2 | 7 | 10 | 3 | 0 | 1 | 4 | 3 | 0 | 7 | 1 | 3 | 4 | 2 | 0 | 0 | 0 | 1 | 3 | 1 |
| 8118 | 4 | 7 | 2 | 3 | 0 | 1 | 4 | 2 | 1 | 8 | 1 | 3 | 0 | 2 | 0 | 1 | 6 | 1 | 0 | 1 |
| 8119 | 3 | 5 | 2 | 2 | 0 | 1 | 4 | 2 | 1 | 8 | 1 | 2 | 0 | 2 | 0 | 1 | 1 | 1 | 0 | 1 |
| 8120 | 3 | 7 | 0 | 2 | 0 | 1 | 3 | 3 | 0 | 7 | 1 | 3 | 4 | 2 | 8 | 0 | 4 | 1 | 3 | 1 |
| 8121 | 2 | 7 | 4 | 0 | 0 | 1 | 2 | 2 | 1 | 6 | 2 | 3 | 4 | 2 | 8 | 0 | 0 | 0 | 0 | 0 |
| 8122 | 2 | 7 | 4 | 3 | 0 | 1 | 3 | 3 | 1 | 6 | 2 | 2 | 4 | 2 | 8 | 0 | 0 | 0 | 0 | 0 |
| 8123 | 3 | 7 | 2 | 2 | 0 | 1 | 4 | 2 | 1 | 3 | 1 | 3 | 0 | 2 | 0 | 1 | 6 | 1 | 0 | 1 |

# Duplicate rows

## Most frequent

|  | cap-shape | stalk-color-above-ring | gill-color | cap-surface | veil-type | gill-attachment | population | stalk-surface-above-ring | bruises? | odor | ring-number | stalk-surface-below-ring | ring-type | veil-color | cap-color | stalk-shape | habitat | gill-size | stalk-root | target | count |
| --- | --- | --- | --- | --- | --- | --- | --- | --- | --- | --- | --- | --- | --- | --- | --- | --- | --- | --- | --- | --- | --- |
| 288 | 2 | 3 | 1 | 0 | 0 | 1 | 4 | 3 | 0 | 6 | 1 | 3 | 4 | 2 | 0 | 1 | 6 | 0 | 1 | 0 | 6 |
| 289 | 2 | 3 | 1 | 0 | 0 | 1 | 4 | 3 | 0 | 6 | 1 | 3 | 4 | 2 | 3 | 1 | 6 | 0 | 1 | 0 | 6 |
| 290 | 2 | 3 | 1 | 0 | 0 | 1 | 4 | 3 | 0 | 6 | 1 | 3 | 4 | 2 | 7 | 1 | 6 | 0 | 1 | 0 | 6 |
| 291 | 2 | 3 | 1 | 0 | 0 | 1 | 5 | 3 | 0 | 6 | 1 | 3 | 4 | 2 | 0 | 1 | 6 | 0 | 1 | 0 | 6 |
| 292 | 2 | 3 | 1 | 0 | 0 | 1 | 5 | 3 | 0 | 6 | 1 | 3 | 4 | 2 | 3 | 1 | 6 | 0 | 1 | 0 | 6 |
| 293 | 2 | 3 | 1 | 0 | 0 | 1 | 5 | 3 | 0 | 6 | 1 | 3 | 4 | 2 | 7 | 1 | 6 | 0 | 1 | 0 | 6 |
| 294 | 2 | 3 | 1 | 2 | 0 | 1 | 4 | 3 | 0 | 6 | 1 | 3 | 4 | 2 | 0 | 1 | 6 | 0 | 1 | 0 | 6 |
| 295 | 2 | 3 | 1 | 2 | 0 | 1 | 4 | 3 | 0 | 6 | 1 | 3 | 4 | 2 | 3 | 1 | 6 | 0 | 1 | 0 | 6 |
| 296 | 2 | 3 | 1 | 2 | 0 | 1 | 4 | 3 | 0 | 6 | 1 | 3 | 4 | 2 | 7 | 1 | 6 | 0 | 1 | 0 | 6 |
| 297 | 2 | 3 | 1 | 2 | 0 | 1 | 5 | 3 | 0 | 6 | 1 | 3 | 4 | 2 | 0 | 1 | 6 | 0 | 1 | 0 | 6 |

Report generated with pandas-profiling.

 
