# Supplementary material for: TPOT-NN: augmenting tree-based automated machine learning with neural network estimators
Source: Genet Program Evolvable Mach. Author manuscript; Available in PMC 2025 Aug 6. (PMC12327408; doi:10.1007/s10710-021-09401-z)
Supplement: Supplementary Material 1 [file NIHMS2035489-supplement-Supplementary_Material_1.zip › New folder/spambase.html]

spambase 

Toggle navigationspambase

- Overview
- Variables
- Interactions
- Correlations
- Missing values
- Sample
- Duplicate rows

# Overview

- Overview
- Reproduction
- Warnings 21

Dataset statistics

|  |  |
| --- | --- |
| Number of variables | 20 |
| Number of observations | 4601 |
| Missing cells | 0 |
| Missing cells (%) | 0.0% |
| Duplicate rows | 1369 |
| Duplicate rows (%) | 29.8% |
| Total size in memory | 719.0 KiB |
| Average record size in memory | 160.0 B |

Variable types

|  |  |
| --- | --- |
| NUM | 19 |
| BOOL | 1 |

Reproduction

|  |  |
| --- | --- |
| Analysis started | 2020-08-25 01:54:16.910714 |
| Analysis finished | 2020-08-25 01:55:09.280859 |
| Duration | 52.37 seconds |
| Version | pandas-profiling v2.8.0 |
| Command line | `pandas_profiling --config_file config.yaml [YOUR_FILE.csv]` |
| Download configuration | config.yaml |

Warnings

|  |  |
| --- | --- |
| Dataset has 1369 (29.8%) duplicate rows | Duplicates |
| `55` is highly skewed (γ1 = 30.76499258) | Skewed |
| `3` is highly skewed (γ1 = 26.22774447) | Skewed |
| `0` has 3548 (77.1%) zeros | Zeros |
| `5` has 3602 (78.3%) zeros | Zeros |
| `30` has 4308 (93.6%) zeros | Zeros |
| `13` has 4244 (92.2%) zeros | Zeros |
| `34` has 4116 (89.5%) zeros | Zeros |
| `27` has 4138 (89.9%) zeros | Zeros |
| `31` has 4396 (95.5%) zeros | Zeros |
| `45` has 4084 (88.8%) zeros | Zeros |
| `12` has 3749 (81.5%) zeros | Zeros |
| `47` has 4398 (95.6%) zeros | Zeros |
| `3` has 4554 (99.0%) zeros | Zeros |
| `43` has 4274 (92.9%) zeros | Zeros |
| `26` has 3821 (83.0%) zeros | Zeros |
| `8` has 3828 (83.2%) zeros | Zeros |
| `17` has 3563 (77.4%) zeros | Zeros |
| `6` has 3794 (82.5%) zeros | Zeros |
| `4` has 2853 (62.0%) zeros | Zeros |
| `40` has 4453 (96.8%) zeros | Zeros |

# Variables

0  
Real number (ℝ≥0)

`ZEROS`

|  |  |
| --- | --- |
| Distinct count | 142 |
| Unique (%) | 3.1% |
| Missing | 0 |
| Missing (%) | 0.0% |
| Infinite | 0 |
| Infinite (%) | 0.0% |

|  |  |
| --- | --- |
| Mean | 0.10455335796565964 |
| Minimum | 0.0 |
| Maximum | 4.54 |
| Zeros | 3548 |
| Zeros (%) | 77.1% |
| Memory size | 36.1 KiB |

2020-08-25T01:55:09.326935image/svg+xmlMatplotlib v3.3.1, https://matplotlib.org/

Toggle details

- Statistics
- Histogram(s)
- Common values
- Extreme values

Quantile statistics

|  |  |
| --- | --- |
| Minimum | 0 |
| 5-th percentile | 0 |
| Q1 | 0 |
| median | 0 |
| Q3 | 0 |
| 95-th percentile | 0.65 |
| Maximum | 4.54 |
| Range | 4.54 |
| Interquartile range (IQR) | 0 |

Descriptive statistics

|  |  |
| --- | --- |
| Standard deviation | 0.305357562 |
| Coefficient of variation (CV) | 2.920590672 |
| Kurtosis | 49.30506416 |
| Mean | 0.104553358 |
| Median Absolute Deviation (MAD) | 0 |
| Skewness | 5.675639164 |
| Sum | 481.05 |
| Variance | 0.09324324068 |

- Histogram

2020-08-25T01:55:09.429944image/svg+xmlMatplotlib v3.3.1, https://matplotlib.org/ 

**Histogram with fixed size bins** (bins=10)

| Value | Count | Frequency (%) |  |
| --- | --- | --- | --- |
| 0 | 3548 | 77.1% |  |
| 0.1 | 51 | 1.1% |  |
| 0.09 | 41 | 0.9% |  |
| 0.17 | 38 | 0.8% |  |
| 0.08 | 34 | 0.7% |  |
| 0.05 | 27 | 0.6% |  |
| 0.07 | 22 | 0.5% |  |
| 0.06 | 20 | 0.4% |  |
| 0.34 | 20 | 0.4% |  |
| 0.33 | 19 | 0.4% |  |
| 0.43 | 17 | 0.4% |  |
| 0.14 | 17 | 0.4% |  |
| 0.12 | 16 | 0.3% |  |
| 0.26 | 16 | 0.3% |  |
| 0.16 | 16 | 0.3% |  |
| 0.23 | 16 | 0.3% |  |
| 0.19 | 16 | 0.3% |  |
| 0.27 | 15 | 0.3% |  |
| 0.11 | 14 | 0.3% |  |
| 0.13 | 14 | 0.3% |  |
| 0.18 | 14 | 0.3% |  |
| 0.32 | 14 | 0.3% |  |
| 0.15 | 13 | 0.3% |  |
| 0.47 | 13 | 0.3% |  |
| 0.46 | 13 | 0.3% |  |
| Other values (117) | 557 | 12.1% |  |

- Minimum 5 values
- Maximum 5 values

| Value | Count | Frequency (%) |  |
| --- | --- | --- | --- |
| 0 | 3548 | 77.1% |  |
| 0.01 | 4 | 0.1% |  |
| 0.02 | 4 | 0.1% |  |
| 0.03 | 4 | 0.1% |  |
| 0.04 | 7 | 0.2% |  |
| 0.05 | 27 | 0.6% |  |
| 0.06 | 20 | 0.4% |  |
| 0.07 | 22 | 0.5% |  |
| 0.08 | 34 | 0.7% |  |
| 0.09 | 41 | 0.9% |  |

| Value | Count | Frequency (%) |  |
| --- | --- | --- | --- |
| 4.54 | 1 | < 0.1% |  |
| 4.34 | 1 | < 0.1% |  |
| 4 | 1 | < 0.1% |  |
| 3.94 | 1 | < 0.1% |  |
| 3.84 | 1 | < 0.1% |  |
| 3.03 | 1 | < 0.1% |  |
| 2.85 | 1 | < 0.1% |  |
| 2.77 | 2 | < 0.1% |  |
| 2.43 | 1 | < 0.1% |  |
| 2.35 | 1 | < 0.1% |  |

5  
Real number (ℝ≥0)

`ZEROS`

|  |  |
| --- | --- |
| Distinct count | 141 |
| Unique (%) | 3.1% |
| Missing | 0 |
| Missing (%) | 0.0% |
| Infinite | 0 |
| Infinite (%) | 0.0% |

|  |  |
| --- | --- |
| Mean | 0.09590089111062812 |
| Minimum | 0.0 |
| Maximum | 5.88 |
| Zeros | 3602 |
| Zeros (%) | 78.3% |
| Memory size | 36.1 KiB |

2020-08-25T01:55:09.545477image/svg+xmlMatplotlib v3.3.1, https://matplotlib.org/

Toggle details

- Statistics
- Histogram(s)
- Common values
- Extreme values

Quantile statistics

|  |  |
| --- | --- |
| Minimum | 0 |
| 5-th percentile | 0 |
| Q1 | 0 |
| median | 0 |
| Q3 | 0 |
| 95-th percentile | 0.63 |
| Maximum | 5.88 |
| Range | 5.88 |
| Interquartile range (IQR) | 0 |

Descriptive statistics

|  |  |
| --- | --- |
| Standard deviation | 0.273824083 |
| Coefficient of variation (CV) | 2.855281946 |
| Kurtosis | 68.445258 |
| Mean | 0.09590089111 |
| Median Absolute Deviation (MAD) | 0 |
| Skewness | 5.956952736 |
| Sum | 441.24 |
| Variance | 0.07497962844 |

- Histogram

2020-08-25T01:55:09.651445image/svg+xmlMatplotlib v3.3.1, https://matplotlib.org/ 

**Histogram with fixed size bins** (bins=10)

| Value | Count | Frequency (%) |  |
| --- | --- | --- | --- |
| 0 | 3602 | 78.3% |  |
| 0.09 | 33 | 0.7% |  |
| 0.1 | 32 | 0.7% |  |
| 0.19 | 25 | 0.5% |  |
| 0.03 | 25 | 0.5% |  |
| 0.08 | 25 | 0.5% |  |
| 0.16 | 24 | 0.5% |  |
| 0.11 | 23 | 0.5% |  |
| 0.32 | 21 | 0.5% |  |
| 0.25 | 20 | 0.4% |  |
| 0.13 | 20 | 0.4% |  |
| 0.36 | 19 | 0.4% |  |
| 0.05 | 19 | 0.4% |  |
| 0.17 | 19 | 0.4% |  |
| 0.2 | 18 | 0.4% |  |
| 0.65 | 17 | 0.4% |  |
| 0.23 | 17 | 0.4% |  |
| 0.38 | 17 | 0.4% |  |
| 0.22 | 17 | 0.4% |  |
| 0.8 | 16 | 0.3% |  |
| 0.26 | 16 | 0.3% |  |
| 0.12 | 16 | 0.3% |  |
| 0.64 | 15 | 0.3% |  |
| 0.27 | 15 | 0.3% |  |
| 0.34 | 14 | 0.3% |  |
| Other values (116) | 516 | 11.2% |  |

- Minimum 5 values
- Maximum 5 values

| Value | Count | Frequency (%) |  |
| --- | --- | --- | --- |
| 0 | 3602 | 78.3% |  |
| 0.01 | 1 | < 0.1% |  |
| 0.02 | 4 | 0.1% |  |
| 0.03 | 25 | 0.5% |  |
| 0.04 | 6 | 0.1% |  |
| 0.05 | 19 | 0.4% |  |
| 0.06 | 6 | 0.1% |  |
| 0.07 | 7 | 0.2% |  |
| 0.08 | 25 | 0.5% |  |
| 0.09 | 33 | 0.7% |  |

| Value | Count | Frequency (%) |  |
| --- | --- | --- | --- |
| 5.88 | 1 | < 0.1% |  |
| 3.57 | 1 | < 0.1% |  |
| 3.44 | 1 | < 0.1% |  |
| 2.94 | 1 | < 0.1% |  |
| 2.63 | 1 | < 0.1% |  |
| 2.54 | 1 | < 0.1% |  |
| 2.43 | 1 | < 0.1% |  |
| 2.3 | 2 | < 0.1% |  |
| 2.1 | 2 | < 0.1% |  |
| 1.88 | 3 | 0.1% |  |

30  
Real number (ℝ≥0)

`ZEROS`

|  |  |
| --- | --- |
| Distinct count | 128 |
| Unique (%) | 2.8% |
| Missing | 0 |
| Missing (%) | 0.0% |
| Infinite | 0 |
| Infinite (%) | 0.0% |

|  |  |
| --- | --- |
| Mean | 0.06475331449684851 |
| Minimum | 0.0 |
| Maximum | 12.5 |
| Zeros | 4308 |
| Zeros (%) | 93.6% |
| Memory size | 36.1 KiB |

2020-08-25T01:55:09.760057image/svg+xmlMatplotlib v3.3.1, https://matplotlib.org/

Toggle details

- Statistics
- Histogram(s)
- Common values
- Extreme values

Quantile statistics

|  |  |
| --- | --- |
| Minimum | 0 |
| 5-th percentile | 0 |
| Q1 | 0 |
| median | 0 |
| Q3 | 0 |
| 95-th percentile | 0.28 |
| Maximum | 12.5 |
| Range | 12.5 |
| Interquartile range (IQR) | 0 |

Descriptive statistics

|  |  |
| --- | --- |
| Standard deviation | 0.4033925009 |
| Coefficient of variation (CV) | 6.229681122 |
| Kurtosis | 254.2325086 |
| Mean | 0.0647533145 |
| Median Absolute Deviation (MAD) | 0 |
| Skewness | 12.66908113 |
| Sum | 297.93 |
| Variance | 0.1627255098 |

- Histogram

2020-08-25T01:55:10.039058image/svg+xmlMatplotlib v3.3.1, https://matplotlib.org/ 

**Histogram with fixed size bins** (bins=10)

| Value | Count | Frequency (%) |  |
| --- | --- | --- | --- |
| 0 | 4308 | 93.6% |  |
| 0.7 | 9 | 0.2% |  |
| 0.58 | 8 | 0.2% |  |
| 0.24 | 7 | 0.2% |  |
| 4.76 | 6 | 0.1% |  |
| 0.26 | 6 | 0.1% |  |
| 0.27 | 6 | 0.1% |  |
| 0.34 | 5 | 0.1% |  |
| 0.15 | 5 | 0.1% |  |
| 0.22 | 5 | 0.1% |  |
| 0.18 | 5 | 0.1% |  |
| 0.55 | 5 | 0.1% |  |
| 0.23 | 5 | 0.1% |  |
| 0.39 | 5 | 0.1% |  |
| 0.59 | 4 | 0.1% |  |
| 0.42 | 4 | 0.1% |  |
| 0.16 | 4 | 0.1% |  |
| 0.86 | 4 | 0.1% |  |
| 0.33 | 4 | 0.1% |  |
| 0.54 | 4 | 0.1% |  |
| 0.68 | 4 | 0.1% |  |
| 0.32 | 4 | 0.1% |  |
| 0.5 | 4 | 0.1% |  |
| 0.73 | 3 | 0.1% |  |
| 3.57 | 3 | 0.1% |  |
| Other values (103) | 174 | 3.8% |  |

- Minimum 5 values
- Maximum 5 values

| Value | Count | Frequency (%) |  |
| --- | --- | --- | --- |
| 0 | 4308 | 93.6% |  |
| 0.09 | 3 | 0.1% |  |
| 0.1 | 1 | < 0.1% |  |
| 0.12 | 1 | < 0.1% |  |
| 0.13 | 2 | < 0.1% |  |
| 0.14 | 1 | < 0.1% |  |
| 0.15 | 5 | 0.1% |  |
| 0.16 | 4 | 0.1% |  |
| 0.17 | 3 | 0.1% |  |
| 0.18 | 5 | 0.1% |  |

| Value | Count | Frequency (%) |  |
| --- | --- | --- | --- |
| 12.5 | 1 | < 0.1% |  |
| 4.76 | 6 | 0.1% |  |
| 4.54 | 2 | < 0.1% |  |
| 4.34 | 3 | 0.1% |  |
| 4.16 | 3 | 0.1% |  |
| 4 | 1 | < 0.1% |  |
| 3.84 | 1 | < 0.1% |  |
| 3.57 | 3 | 0.1% |  |
| 3.26 | 1 | < 0.1% |  |
| 3.12 | 1 | < 0.1% |  |

13  
Real number (ℝ≥0)

`ZEROS`

|  |  |
| --- | --- |
| Distinct count | 133 |
| Unique (%) | 2.9% |
| Missing | 0 |
| Missing (%) | 0.0% |
| Infinite | 0 |
| Infinite (%) | 0.0% |

|  |  |
| --- | --- |
| Mean | 0.05862638556835471 |
| Minimum | 0.0 |
| Maximum | 10.0 |
| Zeros | 4244 |
| Zeros (%) | 92.2% |
| Memory size | 36.1 KiB |

2020-08-25T01:55:10.145849image/svg+xmlMatplotlib v3.3.1, https://matplotlib.org/

Toggle details

- Statistics
- Histogram(s)
- Common values
- Extreme values

Quantile statistics

|  |  |
| --- | --- |
| Minimum | 0 |
| 5-th percentile | 0 |
| Q1 | 0 |
| median | 0 |
| Q3 | 0 |
| 95-th percentile | 0.2 |
| Maximum | 10 |
| Range | 10 |
| Interquartile range (IQR) | 0 |

Descriptive statistics

|  |  |
| --- | --- |
| Standard deviation | 0.3351838298 |
| Coefficient of variation (CV) | 5.717286278 |
| Kurtosis | 229.2012712 |
| Mean | 0.05862638557 |
| Median Absolute Deviation (MAD) | 0 |
| Skewness | 11.75464548 |
| Sum | 269.74 |
| Variance | 0.1123481997 |

- Histogram

2020-08-25T01:55:10.243628image/svg+xmlMatplotlib v3.3.1, https://matplotlib.org/ 

**Histogram with fixed size bins** (bins=10)

| Value | Count | Frequency (%) |  |
| --- | --- | --- | --- |
| 0 | 4244 | 92.2% |  |
| 0.36 | 19 | 0.4% |  |
| 0.05 | 16 | 0.3% |  |
| 0.08 | 16 | 0.3% |  |
| 0.17 | 13 | 0.3% |  |
| 0.07 | 10 | 0.2% |  |
| 1.19 | 9 | 0.2% |  |
| 0.19 | 9 | 0.2% |  |
| 0.06 | 9 | 0.2% |  |
| 0.11 | 9 | 0.2% |  |
| 0.1 | 7 | 0.2% |  |
| 0.09 | 7 | 0.2% |  |
| 0.16 | 7 | 0.2% |  |
| 0.58 | 6 | 0.1% |  |
| 0.6 | 6 | 0.1% |  |
| 0.87 | 6 | 0.1% |  |
| 1.69 | 5 | 0.1% |  |
| 0.2 | 5 | 0.1% |  |
| 0.23 | 5 | 0.1% |  |
| 1.27 | 5 | 0.1% |  |
| 1.23 | 5 | 0.1% |  |
| 1.26 | 5 | 0.1% |  |
| 0.37 | 4 | 0.1% |  |
| 2.06 | 4 | 0.1% |  |
| 0.38 | 4 | 0.1% |  |
| Other values (108) | 166 | 3.6% |  |

- Minimum 5 values
- Maximum 5 values

| Value | Count | Frequency (%) |  |
| --- | --- | --- | --- |
| 0 | 4244 | 92.2% |  |
| 0.01 | 1 | < 0.1% |  |
| 0.02 | 2 | < 0.1% |  |
| 0.03 | 2 | < 0.1% |  |
| 0.04 | 2 | < 0.1% |  |
| 0.05 | 16 | 0.3% |  |
| 0.06 | 9 | 0.2% |  |
| 0.07 | 10 | 0.2% |  |
| 0.08 | 16 | 0.3% |  |
| 0.09 | 7 | 0.2% |  |

| Value | Count | Frequency (%) |  |
| --- | --- | --- | --- |
| 10 | 1 | < 0.1% |  |
| 5.55 | 1 | < 0.1% |  |
| 5.12 | 1 | < 0.1% |  |
| 4.76 | 1 | < 0.1% |  |
| 4.34 | 1 | < 0.1% |  |
| 3.84 | 1 | < 0.1% |  |
| 3.44 | 1 | < 0.1% |  |
| 3.12 | 1 | < 0.1% |  |
| 2.94 | 3 | 0.1% |  |
| 2.85 | 1 | < 0.1% |  |

34  
Real number (ℝ≥0)

`ZEROS`

|  |  |
| --- | --- |
| Distinct count | 177 |
| Unique (%) | 3.8% |
| Missing | 0 |
| Missing (%) | 0.0% |
| Infinite | 0 |
| Infinite (%) | 0.0% |

|  |  |
| --- | --- |
| Mean | 0.10541186698543796 |
| Minimum | 0.0 |
| Maximum | 20.0 |
| Zeros | 4116 |
| Zeros (%) | 89.5% |
| Memory size | 36.1 KiB |

2020-08-25T01:55:10.346933image/svg+xmlMatplotlib v3.3.1, https://matplotlib.org/

Toggle details

- Statistics
- Histogram(s)
- Common values
- Extreme values

Quantile statistics

|  |  |
| --- | --- |
| Minimum | 0 |
| 5-th percentile | 0 |
| Q1 | 0 |
| median | 0 |
| Q3 | 0 |
| 95-th percentile | 0.62 |
| Maximum | 20 |
| Range | 20 |
| Interquartile range (IQR) | 0 |

Descriptive statistics

|  |  |
| --- | --- |
| Standard deviation | 0.5322598753 |
| Coefficient of variation (CV) | 5.049335436 |
| Kurtosis | 449.3742708 |
| Mean | 0.105411867 |
| Median Absolute Deviation (MAD) | 0 |
| Skewness | 15.23081146 |
| Sum | 485 |
| Variance | 0.2833005749 |

- Histogram

2020-08-25T01:55:10.449559image/svg+xmlMatplotlib v3.3.1, https://matplotlib.org/ 

**Histogram with fixed size bins** (bins=10)

| Value | Count | Frequency (%) |  |
| --- | --- | --- | --- |
| 0 | 4116 | 89.5% |  |
| 0.1 | 14 | 0.3% |  |
| 0.33 | 11 | 0.2% |  |
| 0.24 | 11 | 0.2% |  |
| 0.58 | 9 | 0.2% |  |
| 0.5 | 8 | 0.2% |  |
| 0.19 | 7 | 0.2% |  |
| 0.29 | 7 | 0.2% |  |
| 0.34 | 7 | 0.2% |  |
| 0.26 | 7 | 0.2% |  |
| 0.22 | 7 | 0.2% |  |
| 0.31 | 6 | 0.1% |  |
| 0.23 | 6 | 0.1% |  |
| 0.44 | 6 | 0.1% |  |
| 4.76 | 6 | 0.1% |  |
| 0.86 | 6 | 0.1% |  |
| 0.08 | 6 | 0.1% |  |
| 0.39 | 6 | 0.1% |  |
| 0.67 | 5 | 0.1% |  |
| 0.54 | 5 | 0.1% |  |
| 0.43 | 5 | 0.1% |  |
| 0.42 | 5 | 0.1% |  |
| 0.28 | 5 | 0.1% |  |
| 1.72 | 5 | 0.1% |  |
| 0.15 | 5 | 0.1% |  |
| Other values (152) | 320 | 7.0% |  |

- Minimum 5 values
- Maximum 5 values

| Value | Count | Frequency (%) |  |
| --- | --- | --- | --- |
| 0 | 4116 | 89.5% |  |
| 0.01 | 1 | < 0.1% |  |
| 0.03 | 2 | < 0.1% |  |
| 0.04 | 1 | < 0.1% |  |
| 0.06 | 3 | 0.1% |  |
| 0.07 | 2 | < 0.1% |  |
| 0.08 | 6 | 0.1% |  |
| 0.09 | 3 | 0.1% |  |
| 0.1 | 14 | 0.3% |  |
| 0.11 | 1 | < 0.1% |  |

| Value | Count | Frequency (%) |  |
| --- | --- | --- | --- |
| 20 | 1 | < 0.1% |  |
| 5.88 | 1 | < 0.1% |  |
| 4.76 | 6 | 0.1% |  |
| 4.65 | 1 | < 0.1% |  |
| 4.54 | 1 | < 0.1% |  |
| 4.44 | 1 | < 0.1% |  |
| 4.34 | 3 | 0.1% |  |
| 4.16 | 3 | 0.1% |  |
| 4.11 | 1 | < 0.1% |  |
| 4 | 1 | < 0.1% |  |

55  
Real number (ℝ≥0)

`SKEWED`

|  |  |
| --- | --- |
| Distinct count | 271 |
| Unique (%) | 5.9% |
| Missing | 0 |
| Missing (%) | 0.0% |
| Infinite | 0 |
| Infinite (%) | 0.0% |

|  |  |
| --- | --- |
| Mean | 52.17278852423386 |
| Minimum | 1 |
| Maximum | 9989 |
| Zeros | 0 |
| Zeros (%) | 0.0% |
| Memory size | 36.1 KiB |

2020-08-25T01:55:10.568154image/svg+xmlMatplotlib v3.3.1, https://matplotlib.org/

Toggle details

- Statistics
- Histogram(s)
- Common values
- Extreme values

Quantile statistics

|  |  |
| --- | --- |
| Minimum | 1 |
| 5-th percentile | 1 |
| Q1 | 6 |
| median | 15 |
| Q3 | 43 |
| 95-th percentile | 181 |
| Maximum | 9989 |
| Range | 9988 |
| Interquartile range (IQR) | 37 |

Descriptive statistics

|  |  |
| --- | --- |
| Standard deviation | 194.8913095 |
| Coefficient of variation (CV) | 3.735497278 |
| Kurtosis | 1480.64205 |
| Mean | 52.17278852 |
| Median Absolute Deviation (MAD) | 11 |
| Skewness | 30.76499258 |
| Sum | 240047 |
| Variance | 37982.62253 |

- Histogram

2020-08-25T01:55:10.667121image/svg+xmlMatplotlib v3.3.1, https://matplotlib.org/ 

**Histogram with fixed size bins** (bins=10)

| Value | Count | Frequency (%) |  |
| --- | --- | --- | --- |
| 1 | 349 | 7.6% |  |
| 5 | 247 | 5.4% |  |
| 11 | 228 | 5.0% |  |
| 4 | 221 | 4.8% |  |
| 12 | 219 | 4.8% |  |
| 3 | 165 | 3.6% |  |
| 7 | 134 | 2.9% |  |
| 15 | 133 | 2.9% |  |
| 13 | 132 | 2.9% |  |
| 10 | 128 | 2.8% |  |
| 6 | 122 | 2.7% |  |
| 8 | 109 | 2.4% |  |
| 2 | 97 | 2.1% |  |
| 9 | 78 | 1.7% |  |
| 17 | 75 | 1.6% |  |
| 19 | 70 | 1.5% |  |
| 22 | 66 | 1.4% |  |
| 28 | 63 | 1.4% |  |
| 16 | 61 | 1.3% |  |
| 18 | 61 | 1.3% |  |
| 14 | 60 | 1.3% |  |
| 21 | 56 | 1.2% |  |
| 47 | 53 | 1.2% |  |
| 25 | 52 | 1.1% |  |
| 20 | 44 | 1.0% |  |
| Other values (246) | 1578 | 34.3% |  |

- Minimum 5 values
- Maximum 5 values

| Value | Count | Frequency (%) |  |
| --- | --- | --- | --- |
| 1 | 349 | 7.6% |  |
| 2 | 97 | 2.1% |  |
| 3 | 165 | 3.6% |  |
| 4 | 221 | 4.8% |  |
| 5 | 247 | 5.4% |  |
| 6 | 122 | 2.7% |  |
| 7 | 134 | 2.9% |  |
| 8 | 109 | 2.4% |  |
| 9 | 78 | 1.7% |  |
| 10 | 128 | 2.8% |  |

| Value | Count | Frequency (%) |  |
| --- | --- | --- | --- |
| 9989 | 1 | < 0.1% |  |
| 2204 | 1 | < 0.1% |  |
| 2042 | 1 | < 0.1% |  |
| 1505 | 1 | < 0.1% |  |
| 1488 | 1 | < 0.1% |  |
| 1333 | 1 | < 0.1% |  |
| 1327 | 1 | < 0.1% |  |
| 1325 | 2 | < 0.1% |  |
| 1177 | 3 | 0.1% |  |
| 1171 | 2 | < 0.1% |  |

27  
Real number (ℝ≥0)

`ZEROS`

|  |  |
| --- | --- |
| Distinct count | 200 |
| Unique (%) | 4.3% |
| Missing | 0 |
| Missing (%) | 0.0% |
| Infinite | 0 |
| Infinite (%) | 0.0% |

|  |  |
| --- | --- |
| Mean | 0.12484459900021737 |
| Minimum | 0.0 |
| Maximum | 9.09 |
| Zeros | 4138 |
| Zeros (%) | 89.9% |
| Memory size | 36.1 KiB |

2020-08-25T01:55:10.768364image/svg+xmlMatplotlib v3.3.1, https://matplotlib.org/

Toggle details

- Statistics
- Histogram(s)
- Common values
- Extreme values

Quantile statistics

|  |  |
| --- | --- |
| Minimum | 0 |
| 5-th percentile | 0 |
| Q1 | 0 |
| median | 0 |
| Q3 | 0 |
| 95-th percentile | 0.8 |
| Maximum | 9.09 |
| Range | 9.09 |
| Interquartile range (IQR) | 0 |

Descriptive statistics

|  |  |
| --- | --- |
| Standard deviation | 0.538576044 |
| Coefficient of variation (CV) | 4.313971516 |
| Kurtosis | 58.37302181 |
| Mean | 0.124844599 |
| Median Absolute Deviation (MAD) | 0 |
| Skewness | 6.606533933 |
| Sum | 574.41 |
| Variance | 0.2900641552 |

- Histogram

2020-08-25T01:55:10.873431image/svg+xmlMatplotlib v3.3.1, https://matplotlib.org/ 

**Histogram with fixed size bins** (bins=10)

| Value | Count | Frequency (%) |  |
| --- | --- | --- | --- |
| 0 | 4138 | 89.9% |  |
| 0.24 | 7 | 0.2% |  |
| 0.68 | 7 | 0.2% |  |
| 4.76 | 7 | 0.2% |  |
| 2.04 | 7 | 0.2% |  |
| 0.66 | 6 | 0.1% |  |
| 0.63 | 6 | 0.1% |  |
| 0.15 | 6 | 0.1% |  |
| 0.54 | 6 | 0.1% |  |
| 0.58 | 6 | 0.1% |  |
| 0.39 | 5 | 0.1% |  |
| 0.88 | 5 | 0.1% |  |
| 0.1 | 5 | 0.1% |  |
| 0.29 | 5 | 0.1% |  |
| 4.34 | 5 | 0.1% |  |
| 0.64 | 5 | 0.1% |  |
| 0.5 | 5 | 0.1% |  |
| 0.59 | 5 | 0.1% |  |
| 2.32 | 4 | 0.1% |  |
| 0.67 | 4 | 0.1% |  |
| 0.49 | 4 | 0.1% |  |
| 0.85 | 4 | 0.1% |  |
| 0.55 | 4 | 0.1% |  |
| 0.74 | 4 | 0.1% |  |
| 0.17 | 4 | 0.1% |  |
| Other values (175) | 337 | 7.3% |  |

- Minimum 5 values
- Maximum 5 values

| Value | Count | Frequency (%) |  |
| --- | --- | --- | --- |
| 0 | 4138 | 89.9% |  |
| 0.02 | 2 | < 0.1% |  |
| 0.03 | 1 | < 0.1% |  |
| 0.04 | 3 | 0.1% |  |
| 0.05 | 3 | 0.1% |  |
| 0.06 | 3 | 0.1% |  |
| 0.08 | 3 | 0.1% |  |
| 0.09 | 2 | < 0.1% |  |
| 0.1 | 5 | 0.1% |  |
| 0.11 | 1 | < 0.1% |  |

| Value | Count | Frequency (%) |  |
| --- | --- | --- | --- |
| 9.09 | 1 | < 0.1% |  |
| 8.33 | 1 | < 0.1% |  |
| 5.88 | 1 | < 0.1% |  |
| 4.76 | 7 | 0.2% |  |
| 4.65 | 1 | < 0.1% |  |
| 4.54 | 1 | < 0.1% |  |
| 4.44 | 1 | < 0.1% |  |
| 4.34 | 5 | 0.1% |  |
| 4.25 | 1 | < 0.1% |  |
| 4.16 | 3 | 0.1% |  |

31  
Real number (ℝ≥0)

`ZEROS`

|  |  |
| --- | --- |
| Distinct count | 106 |
| Unique (%) | 2.3% |
| Missing | 0 |
| Missing (%) | 0.0% |
| Infinite | 0 |
| Infinite (%) | 0.0% |

|  |  |
| --- | --- |
| Mean | 0.047048467724407746 |
| Minimum | 0.0 |
| Maximum | 4.76 |
| Zeros | 4396 |
| Zeros (%) | 95.5% |
| Memory size | 36.1 KiB |

2020-08-25T01:55:10.974413image/svg+xmlMatplotlib v3.3.1, https://matplotlib.org/

Toggle details

- Statistics
- Histogram(s)
- Common values
- Extreme values

Quantile statistics

|  |  |
| --- | --- |
| Minimum | 0 |
| 5-th percentile | 0 |
| Q1 | 0 |
| median | 0 |
| Q3 | 0 |
| 95-th percentile | 0 |
| Maximum | 4.76 |
| Range | 4.76 |
| Interquartile range (IQR) | 0 |

Descriptive statistics

|  |  |
| --- | --- |
| Standard deviation | 0.3285588817 |
| Coefficient of variation (CV) | 6.983413012 |
| Kurtosis | 127.3765293 |
| Mean | 0.04704846772 |
| Median Absolute Deviation (MAD) | 0 |
| Skewness | 10.54918417 |
| Sum | 216.47 |
| Variance | 0.1079509387 |

- Histogram

2020-08-25T01:55:11.070698image/svg+xmlMatplotlib v3.3.1, https://matplotlib.org/ 

**Histogram with fixed size bins** (bins=10)

| Value | Count | Frequency (%) |  |
| --- | --- | --- | --- |
| 0 | 4396 | 95.5% |  |
| 0.58 | 7 | 0.2% |  |
| 4.76 | 6 | 0.1% |  |
| 0.68 | 5 | 0.1% |  |
| 0.39 | 5 | 0.1% |  |
| 0.63 | 4 | 0.1% |  |
| 0.24 | 4 | 0.1% |  |
| 0.55 | 4 | 0.1% |  |
| 0.35 | 4 | 0.1% |  |
| 0.17 | 4 | 0.1% |  |
| 0.15 | 4 | 0.1% |  |
| 0.76 | 4 | 0.1% |  |
| 1.01 | 3 | 0.1% |  |
| 0.73 | 3 | 0.1% |  |
| 0.64 | 3 | 0.1% |  |
| 0.86 | 3 | 0.1% |  |
| 0.28 | 3 | 0.1% |  |
| 0.47 | 3 | 0.1% |  |
| 0.33 | 3 | 0.1% |  |
| 0.51 | 3 | 0.1% |  |
| 4.16 | 3 | 0.1% |  |
| 0.87 | 3 | 0.1% |  |
| 4.34 | 3 | 0.1% |  |
| 0.27 | 3 | 0.1% |  |
| 0.66 | 3 | 0.1% |  |
| Other values (81) | 115 | 2.5% |  |

- Minimum 5 values
- Maximum 5 values

| Value | Count | Frequency (%) |  |
| --- | --- | --- | --- |
| 0 | 4396 | 95.5% |  |
| 0.09 | 2 | < 0.1% |  |
| 0.1 | 1 | < 0.1% |  |
| 0.13 | 1 | < 0.1% |  |
| 0.15 | 4 | 0.1% |  |
| 0.16 | 1 | < 0.1% |  |
| 0.17 | 4 | 0.1% |  |
| 0.19 | 2 | < 0.1% |  |
| 0.2 | 2 | < 0.1% |  |
| 0.22 | 2 | < 0.1% |  |

| Value | Count | Frequency (%) |  |
| --- | --- | --- | --- |
| 4.76 | 6 | 0.1% |  |
| 4.7 | 1 | < 0.1% |  |
| 4.54 | 1 | < 0.1% |  |
| 4.34 | 3 | 0.1% |  |
| 4.16 | 3 | 0.1% |  |
| 4 | 1 | < 0.1% |  |
| 3.84 | 1 | < 0.1% |  |
| 3.57 | 1 | < 0.1% |  |
| 3.17 | 1 | < 0.1% |  |
| 3.12 | 1 | < 0.1% |  |

45  
Real number (ℝ≥0)

`ZEROS`

|  |  |
| --- | --- |
| Distinct count | 227 |
| Unique (%) | 4.9% |
| Missing | 0 |
| Missing (%) | 0.0% |
| Infinite | 0 |
| Infinite (%) | 0.0% |

|  |  |
| --- | --- |
| Mean | 0.17982395131493156 |
| Minimum | 0.0 |
| Maximum | 22.05 |
| Zeros | 4084 |
| Zeros (%) | 88.8% |
| Memory size | 36.1 KiB |

2020-08-25T01:55:11.172156image/svg+xmlMatplotlib v3.3.1, https://matplotlib.org/

Toggle details

- Statistics
- Histogram(s)
- Common values
- Extreme values

Quantile statistics

|  |  |
| --- | --- |
| Minimum | 0 |
| 5-th percentile | 0 |
| Q1 | 0 |
| median | 0 |
| Q3 | 0 |
| 95-th percentile | 1.02 |
| Maximum | 22.05 |
| Range | 22.05 |
| Interquartile range (IQR) | 0 |

Descriptive statistics

|  |  |
| --- | --- |
| Standard deviation | 0.9111190632 |
| Coefficient of variation (CV) | 5.066728078 |
| Kurtosis | 150.8998167 |
| Mean | 0.1798239513 |
| Median Absolute Deviation (MAD) | 0 |
| Skewness | 10.12266272 |
| Sum | 827.37 |
| Variance | 0.8301379473 |

- Histogram

2020-08-25T01:55:11.267577image/svg+xmlMatplotlib v3.3.1, https://matplotlib.org/ 

**Histogram with fixed size bins** (bins=10)

| Value | Count | Frequency (%) |  |
| --- | --- | --- | --- |
| 0 | 4084 | 88.8% |  |
| 0.08 | 18 | 0.4% |  |
| 0.1 | 13 | 0.3% |  |
| 0.09 | 10 | 0.2% |  |
| 0.27 | 10 | 0.2% |  |
| 0.16 | 8 | 0.2% |  |
| 0.34 | 8 | 0.2% |  |
| 0.33 | 7 | 0.2% |  |
| 0.28 | 6 | 0.1% |  |
| 0.8 | 6 | 0.1% |  |
| 5 | 5 | 0.1% |  |
| 1.2 | 5 | 0.1% |  |
| 0.37 | 5 | 0.1% |  |
| 1.58 | 5 | 0.1% |  |
| 0.51 | 5 | 0.1% |  |
| 0.25 | 5 | 0.1% |  |
| 2.32 | 5 | 0.1% |  |
| 0.44 | 5 | 0.1% |  |
| 1.85 | 4 | 0.1% |  |
| 0.89 | 4 | 0.1% |  |
| 0.61 | 4 | 0.1% |  |
| 7.14 | 4 | 0.1% |  |
| 0.29 | 4 | 0.1% |  |
| 0.48 | 4 | 0.1% |  |
| 0.46 | 4 | 0.1% |  |
| Other values (202) | 363 | 7.9% |  |

- Minimum 5 values
- Maximum 5 values

| Value | Count | Frequency (%) |  |
| --- | --- | --- | --- |
| 0 | 4084 | 88.8% |  |
| 0.02 | 1 | < 0.1% |  |
| 0.03 | 1 | < 0.1% |  |
| 0.04 | 2 | < 0.1% |  |
| 0.06 | 3 | 0.1% |  |
| 0.07 | 1 | < 0.1% |  |
| 0.08 | 18 | 0.4% |  |
| 0.09 | 10 | 0.2% |  |
| 0.1 | 13 | 0.3% |  |
| 0.11 | 3 | 0.1% |  |

| Value | Count | Frequency (%) |  |
| --- | --- | --- | --- |
| 22.05 | 1 | < 0.1% |  |
| 16.7 | 1 | < 0.1% |  |
| 15.35 | 1 | < 0.1% |  |
| 13.37 | 1 | < 0.1% |  |
| 10 | 2 | < 0.1% |  |
| 9.52 | 1 | < 0.1% |  |
| 9.09 | 3 | 0.1% |  |
| 8.49 | 1 | < 0.1% |  |
| 7.88 | 1 | < 0.1% |  |
| 7.69 | 3 | 0.1% |  |

12  
Real number (ℝ≥0)

`ZEROS`

|  |  |
| --- | --- |
| Distinct count | 158 |
| Unique (%) | 3.4% |
| Missing | 0 |
| Missing (%) | 0.0% |
| Infinite | 0 |
| Infinite (%) | 0.0% |

|  |  |
| --- | --- |
| Mean | 0.09392958052597261 |
| Minimum | 0.0 |
| Maximum | 5.55 |
| Zeros | 3749 |
| Zeros (%) | 81.5% |
| Memory size | 36.1 KiB |

2020-08-25T01:55:11.369760image/svg+xmlMatplotlib v3.3.1, https://matplotlib.org/

Toggle details

- Statistics
- Histogram(s)
- Common values
- Extreme values

Quantile statistics

|  |  |
| --- | --- |
| Minimum | 0 |
| 5-th percentile | 0 |
| Q1 | 0 |
| median | 0 |
| Q3 | 0 |
| 95-th percentile | 0.61 |
| Maximum | 5.55 |
| Range | 5.55 |
| Interquartile range (IQR) | 0 |

Descriptive statistics

|  |  |
| --- | --- |
| Standard deviation | 0.3010358036 |
| Coefficient of variation (CV) | 3.204909485 |
| Kurtosis | 84.94182188 |
| Mean | 0.09392958053 |
| Median Absolute Deviation (MAD) | 0 |
| Skewness | 6.955548227 |
| Sum | 432.17 |
| Variance | 0.09062255504 |

- Histogram

2020-08-25T01:55:11.468129image/svg+xmlMatplotlib v3.3.1, https://matplotlib.org/ 

**Histogram with fixed size bins** (bins=10)

| Value | Count | Frequency (%) |  |
| --- | --- | --- | --- |
| 0 | 3749 | 81.5% |  |
| 0.17 | 43 | 0.9% |  |
| 0.19 | 30 | 0.7% |  |
| 0.3 | 29 | 0.6% |  |
| 0.32 | 26 | 0.6% |  |
| 0.27 | 20 | 0.4% |  |
| 0.12 | 18 | 0.4% |  |
| 0.25 | 17 | 0.4% |  |
| 0.29 | 16 | 0.3% |  |
| 0.2 | 16 | 0.3% |  |
| 0.65 | 15 | 0.3% |  |
| 0.08 | 15 | 0.3% |  |
| 0.38 | 15 | 0.3% |  |
| 0.14 | 15 | 0.3% |  |
| 0.31 | 14 | 0.3% |  |
| 0.11 | 14 | 0.3% |  |
| 0.16 | 13 | 0.3% |  |
| 0.06 | 12 | 0.3% |  |
| 0.09 | 12 | 0.3% |  |
| 0.22 | 12 | 0.3% |  |
| 0.28 | 12 | 0.3% |  |
| 0.54 | 12 | 0.3% |  |
| 0.37 | 12 | 0.3% |  |
| 0.56 | 11 | 0.2% |  |
| 0.15 | 11 | 0.2% |  |
| Other values (133) | 442 | 9.6% |  |

- Minimum 5 values
- Maximum 5 values

| Value | Count | Frequency (%) |  |
| --- | --- | --- | --- |
| 0 | 3749 | 81.5% |  |
| 0.01 | 1 | < 0.1% |  |
| 0.02 | 3 | 0.1% |  |
| 0.03 | 5 | 0.1% |  |
| 0.04 | 4 | 0.1% |  |
| 0.05 | 5 | 0.1% |  |
| 0.06 | 12 | 0.3% |  |
| 0.07 | 6 | 0.1% |  |
| 0.08 | 15 | 0.3% |  |
| 0.09 | 12 | 0.3% |  |

| Value | Count | Frequency (%) |  |
| --- | --- | --- | --- |
| 5.55 | 3 | 0.1% |  |
| 2.94 | 1 | < 0.1% |  |
| 2.71 | 1 | < 0.1% |  |
| 2.63 | 1 | < 0.1% |  |
| 2.58 | 1 | < 0.1% |  |
| 2.56 | 1 | < 0.1% |  |
| 2.5 | 1 | < 0.1% |  |
| 2.46 | 1 | < 0.1% |  |
| 2.38 | 1 | < 0.1% |  |
| 2.22 | 1 | < 0.1% |  |

47  
Real number (ℝ≥0)

`ZEROS`

|  |  |
| --- | --- |
| Distinct count | 106 |
| Unique (%) | 2.3% |
| Missing | 0 |
| Missing (%) | 0.0% |
| Infinite | 0 |
| Infinite (%) | 0.0% |

|  |  |
| --- | --- |
| Mean | 0.03186915887850467 |
| Minimum | 0.0 |
| Maximum | 10.0 |
| Zeros | 4398 |
| Zeros (%) | 95.6% |
| Memory size | 36.1 KiB |

2020-08-25T01:55:11.575016image/svg+xmlMatplotlib v3.3.1, https://matplotlib.org/

Toggle details

- Statistics
- Histogram(s)
- Common values
- Extreme values

Quantile statistics

|  |  |
| --- | --- |
| Minimum | 0 |
| 5-th percentile | 0 |
| Q1 | 0 |
| median | 0 |
| Q3 | 0 |
| 95-th percentile | 0 |
| Maximum | 10 |
| Range | 10 |
| Interquartile range (IQR) | 0 |

Descriptive statistics

|  |  |
| --- | --- |
| Standard deviation | 0.2857346463 |
| Coefficient of variation (CV) | 8.965867201 |
| Kurtosis | 537.4930074 |
| Mean | 0.03186915888 |
| Median Absolute Deviation (MAD) | 0 |
| Skewness | 19.72044578 |
| Sum | 146.63 |
| Variance | 0.08164428809 |

- Histogram

2020-08-25T01:55:11.670989image/svg+xmlMatplotlib v3.3.1, https://matplotlib.org/ 

**Histogram with fixed size bins** (bins=10)

| Value | Count | Frequency (%) |  |
| --- | --- | --- | --- |
| 0 | 4398 | 95.6% |  |
| 0.13 | 10 | 0.2% |  |
| 0.24 | 7 | 0.2% |  |
| 0.2 | 7 | 0.2% |  |
| 0.15 | 6 | 0.1% |  |
| 0.19 | 6 | 0.1% |  |
| 0.1 | 6 | 0.1% |  |
| 0.28 | 5 | 0.1% |  |
| 0.14 | 5 | 0.1% |  |
| 0.04 | 4 | 0.1% |  |
| 0.08 | 4 | 0.1% |  |
| 0.33 | 4 | 0.1% |  |
| 0.11 | 4 | 0.1% |  |
| 0.34 | 3 | 0.1% |  |
| 0.44 | 3 | 0.1% |  |
| 0.35 | 3 | 0.1% |  |
| 0.09 | 3 | 0.1% |  |
| 0.12 | 3 | 0.1% |  |
| 0.05 | 3 | 0.1% |  |
| 0.02 | 3 | 0.1% |  |
| 0.41 | 3 | 0.1% |  |
| 0.3 | 2 | < 0.1% |  |
| 0.54 | 2 | < 0.1% |  |
| 0.26 | 2 | < 0.1% |  |
| 0.53 | 2 | < 0.1% |  |
| Other values (81) | 103 | 2.2% |  |

- Minimum 5 values
- Maximum 5 values

| Value | Count | Frequency (%) |  |
| --- | --- | --- | --- |
| 0 | 4398 | 95.6% |  |
| 0.02 | 3 | 0.1% |  |
| 0.03 | 1 | < 0.1% |  |
| 0.04 | 4 | 0.1% |  |
| 0.05 | 3 | 0.1% |  |
| 0.06 | 1 | < 0.1% |  |
| 0.07 | 2 | < 0.1% |  |
| 0.08 | 4 | 0.1% |  |
| 0.09 | 3 | 0.1% |  |
| 0.1 | 6 | 0.1% |  |

| Value | Count | Frequency (%) |  |
| --- | --- | --- | --- |
| 10 | 1 | < 0.1% |  |
| 8.33 | 1 | < 0.1% |  |
| 5 | 1 | < 0.1% |  |
| 4.76 | 1 | < 0.1% |  |
| 3.7 | 1 | < 0.1% |  |
| 3.33 | 2 | < 0.1% |  |
| 2.81 | 1 | < 0.1% |  |
| 2.56 | 2 | < 0.1% |  |
| 2.5 | 1 | < 0.1% |  |
| 2.45 | 1 | < 0.1% |  |

3  
Real number (ℝ≥0)

`SKEWED`  
`ZEROS`

|  |  |
| --- | --- |
| Distinct count | 43 |
| Unique (%) | 0.9% |
| Missing | 0 |
| Missing (%) | 0.0% |
| Infinite | 0 |
| Infinite (%) | 0.0% |

|  |  |
| --- | --- |
| Mean | 0.06542490762877635 |
| Minimum | 0.0 |
| Maximum | 42.81 |
| Zeros | 4554 |
| Zeros (%) | 99.0% |
| Memory size | 36.1 KiB |

2020-08-25T01:55:11.774554image/svg+xmlMatplotlib v3.3.1, https://matplotlib.org/

Toggle details

- Statistics
- Histogram(s)
- Common values
- Extreme values

Quantile statistics

|  |  |
| --- | --- |
| Minimum | 0 |
| 5-th percentile | 0 |
| Q1 | 0 |
| median | 0 |
| Q3 | 0 |
| 95-th percentile | 0 |
| Maximum | 42.81 |
| Range | 42.81 |
| Interquartile range (IQR) | 0 |

Descriptive statistics

|  |  |
| --- | --- |
| Standard deviation | 1.39515137 |
| Coefficient of variation (CV) | 21.32446833 |
| Kurtosis | 726.4515381 |
| Mean | 0.06542490763 |
| Median Absolute Deviation (MAD) | 0 |
| Skewness | 26.22774447 |
| Sum | 301.02 |
| Variance | 1.946447347 |

- Histogram

2020-08-25T01:55:11.867632image/svg+xmlMatplotlib v3.3.1, https://matplotlib.org/ 

**Histogram with fixed size bins** (bins=10)

| Value | Count | Frequency (%) |  |
| --- | --- | --- | --- |
| 0 | 4554 | 99.0% |  |
| 35.46 | 2 | < 0.1% |  |
| 0.58 | 2 | < 0.1% |  |
| 0.21 | 2 | < 0.1% |  |
| 0.42 | 2 | < 0.1% |  |
| 0.17 | 2 | < 0.1% |  |
| 0.52 | 1 | < 0.1% |  |
| 0.11 | 1 | < 0.1% |  |
| 1.26 | 1 | < 0.1% |  |
| 0.04 | 1 | < 0.1% |  |
| 0.06 | 1 | < 0.1% |  |
| 19.73 | 1 | < 0.1% |  |
| 9.16 | 1 | < 0.1% |  |
| 0.15 | 1 | < 0.1% |  |
| 0.1 | 1 | < 0.1% |  |
| 0.49 | 1 | < 0.1% |  |
| 0.95 | 1 | < 0.1% |  |
| 0.19 | 1 | < 0.1% |  |
| 1.29 | 1 | < 0.1% |  |
| 0.44 | 1 | < 0.1% |  |
| 7.07 | 1 | < 0.1% |  |
| 7.18 | 1 | < 0.1% |  |
| 0.81 | 1 | < 0.1% |  |
| 1.35 | 1 | < 0.1% |  |
| 0.91 | 1 | < 0.1% |  |
| Other values (18) | 18 | 0.4% |  |

- Minimum 5 values
- Maximum 5 values

| Value | Count | Frequency (%) |  |
| --- | --- | --- | --- |
| 0 | 4554 | 99.0% |  |
| 0.04 | 1 | < 0.1% |  |
| 0.06 | 1 | < 0.1% |  |
| 0.1 | 1 | < 0.1% |  |
| 0.11 | 1 | < 0.1% |  |
| 0.13 | 1 | < 0.1% |  |
| 0.14 | 1 | < 0.1% |  |
| 0.15 | 1 | < 0.1% |  |
| 0.16 | 1 | < 0.1% |  |
| 0.17 | 2 | < 0.1% |  |

| Value | Count | Frequency (%) |  |
| --- | --- | --- | --- |
| 42.81 | 1 | < 0.1% |  |
| 42.73 | 1 | < 0.1% |  |
| 40.13 | 1 | < 0.1% |  |
| 35.46 | 2 | < 0.1% |  |
| 19.73 | 1 | < 0.1% |  |
| 19.16 | 1 | < 0.1% |  |
| 13.63 | 1 | < 0.1% |  |
| 9.16 | 1 | < 0.1% |  |
| 7.18 | 1 | < 0.1% |  |
| 7.07 | 1 | < 0.1% |  |

43  
Real number (ℝ≥0)

`ZEROS`

|  |  |
| --- | --- |
| Distinct count | 160 |
| Unique (%) | 3.5% |
| Missing | 0 |
| Missing (%) | 0.0% |
| Infinite | 0 |
| Infinite (%) | 0.0% |

|  |  |
| --- | --- |
| Mean | 0.0791958269941317 |
| Minimum | 0.0 |
| Maximum | 20.0 |
| Zeros | 4274 |
| Zeros (%) | 92.9% |
| Memory size | 36.1 KiB |

2020-08-25T01:55:11.969898image/svg+xmlMatplotlib v3.3.1, https://matplotlib.org/

Toggle details

- Statistics
- Histogram(s)
- Common values
- Extreme values

Quantile statistics

|  |  |
| --- | --- |
| Minimum | 0 |
| 5-th percentile | 0 |
| Q1 | 0 |
| median | 0 |
| Q3 | 0 |
| 95-th percentile | 0.24 |
| Maximum | 20 |
| Range | 20 |
| Interquartile range (IQR) | 0 |

Descriptive statistics

|  |  |
| --- | --- |
| Standard deviation | 0.6219755735 |
| Coefficient of variation (CV) | 7.853640742 |
| Kurtosis | 479.8309068 |
| Mean | 0.07919582699 |
| Median Absolute Deviation (MAD) | 0 |
| Skewness | 18.7715155 |
| Sum | 364.38 |
| Variance | 0.386853614 |

- Histogram

2020-08-25T01:55:12.076832image/svg+xmlMatplotlib v3.3.1, https://matplotlib.org/ 

**Histogram with fixed size bins** (bins=10)

| Value | Count | Frequency (%) |  |
| --- | --- | --- | --- |
| 0 | 4274 | 92.9% |  |
| 0.08 | 13 | 0.3% |  |
| 0.06 | 12 | 0.3% |  |
| 0.05 | 10 | 0.2% |  |
| 0.28 | 10 | 0.2% |  |
| 0.1 | 9 | 0.2% |  |
| 0.33 | 8 | 0.2% |  |
| 0.16 | 8 | 0.2% |  |
| 0.02 | 7 | 0.2% |  |
| 0.8 | 6 | 0.1% |  |
| 0.26 | 6 | 0.1% |  |
| 0.54 | 5 | 0.1% |  |
| 0.58 | 4 | 0.1% |  |
| 0.14 | 4 | 0.1% |  |
| 0.09 | 4 | 0.1% |  |
| 0.03 | 4 | 0.1% |  |
| 0.29 | 3 | 0.1% |  |
| 0.44 | 3 | 0.1% |  |
| 4.54 | 3 | 0.1% |  |
| 1.26 | 3 | 0.1% |  |
| 0.22 | 3 | 0.1% |  |
| 0.37 | 3 | 0.1% |  |
| 0.23 | 3 | 0.1% |  |
| 0.84 | 3 | 0.1% |  |
| 0.64 | 3 | 0.1% |  |
| Other values (135) | 190 | 4.1% |  |

- Minimum 5 values
- Maximum 5 values

| Value | Count | Frequency (%) |  |
| --- | --- | --- | --- |
| 0 | 4274 | 92.9% |  |
| 0.01 | 1 | < 0.1% |  |
| 0.02 | 7 | 0.2% |  |
| 0.03 | 4 | 0.1% |  |
| 0.04 | 2 | < 0.1% |  |
| 0.05 | 10 | 0.2% |  |
| 0.06 | 12 | 0.3% |  |
| 0.07 | 1 | < 0.1% |  |
| 0.08 | 13 | 0.3% |  |
| 0.09 | 4 | 0.1% |  |

| Value | Count | Frequency (%) |  |
| --- | --- | --- | --- |
| 20 | 1 | < 0.1% |  |
| 16.66 | 2 | < 0.1% |  |
| 10 | 1 | < 0.1% |  |
| 8.1 | 1 | < 0.1% |  |
| 6.34 | 1 | < 0.1% |  |
| 6.25 | 1 | < 0.1% |  |
| 5 | 2 | < 0.1% |  |
| 4.76 | 1 | < 0.1% |  |
| 4.57 | 1 | < 0.1% |  |
| 4.54 | 3 | 0.1% |  |

26  
Real number (ℝ≥0)

`ZEROS`

|  |  |
| --- | --- |
| Distinct count | 240 |
| Unique (%) | 5.2% |
| Missing | 0 |
| Missing (%) | 0.0% |
| Infinite | 0 |
| Infinite (%) | 0.0% |

|  |  |
| --- | --- |
| Mean | 0.767304933710063 |
| Minimum | 0.0 |
| Maximum | 33.33 |
| Zeros | 3821 |
| Zeros (%) | 83.0% |
| Memory size | 36.1 KiB |

2020-08-25T01:55:12.191788image/svg+xmlMatplotlib v3.3.1, https://matplotlib.org/

Toggle details

- Statistics
- Histogram(s)
- Common values
- Extreme values

Quantile statistics

|  |  |
| --- | --- |
| Minimum | 0 |
| 5-th percentile | 0 |
| Q1 | 0 |
| median | 0 |
| Q3 | 0 |
| 95-th percentile | 2.73 |
| Maximum | 33.33 |
| Range | 33.33 |
| Interquartile range (IQR) | 0 |

Descriptive statistics

|  |  |
| --- | --- |
| Standard deviation | 3.367291802 |
| Coefficient of variation (CV) | 4.388466247 |
| Kurtosis | 34.20447596 |
| Mean | 0.7673049337 |
| Median Absolute Deviation (MAD) | 0 |
| Skewness | 5.744493294 |
| Sum | 3530.37 |
| Variance | 11.33865408 |

- Histogram

2020-08-25T01:55:12.298054image/svg+xmlMatplotlib v3.3.1, https://matplotlib.org/ 

**Histogram with fixed size bins** (bins=10)

| Value | Count | Frequency (%) |  |
| --- | --- | --- | --- |
| 0 | 3821 | 83.0% |  |
| 20 | 80 | 1.7% |  |
| 25 | 16 | 0.3% |  |
| 0.05 | 15 | 0.3% |  |
| 0.7 | 13 | 0.3% |  |
| 0.08 | 10 | 0.2% |  |
| 16.66 | 10 | 0.2% |  |
| 2 | 9 | 0.2% |  |
| 4.76 | 9 | 0.2% |  |
| 0.68 | 7 | 0.2% |  |
| 4.34 | 7 | 0.2% |  |
| 1.17 | 7 | 0.2% |  |
| 2.63 | 7 | 0.2% |  |
| 3.03 | 7 | 0.2% |  |
| 0.11 | 7 | 0.2% |  |
| 4.16 | 7 | 0.2% |  |
| 0.88 | 6 | 0.1% |  |
| 1.4 | 6 | 0.1% |  |
| 1.63 | 6 | 0.1% |  |
| 1.36 | 6 | 0.1% |  |
| 1.2 | 6 | 0.1% |  |
| 14.28 | 6 | 0.1% |  |
| 2.56 | 6 | 0.1% |  |
| 1.16 | 6 | 0.1% |  |
| 1.19 | 6 | 0.1% |  |
| Other values (215) | 515 | 11.2% |  |

- Minimum 5 values
- Maximum 5 values

| Value | Count | Frequency (%) |  |
| --- | --- | --- | --- |
| 0 | 3821 | 83.0% |  |
| 0.01 | 3 | 0.1% |  |
| 0.02 | 3 | 0.1% |  |
| 0.03 | 3 | 0.1% |  |
| 0.04 | 3 | 0.1% |  |
| 0.05 | 15 | 0.3% |  |
| 0.06 | 5 | 0.1% |  |
| 0.07 | 5 | 0.1% |  |
| 0.08 | 10 | 0.2% |  |
| 0.09 | 4 | 0.1% |  |

| Value | Count | Frequency (%) |  |
| --- | --- | --- | --- |
| 33.33 | 4 | 0.1% |  |
| 25 | 16 | 0.3% |  |
| 20 | 80 | 1.7% |  |
| 16.66 | 10 | 0.2% |  |
| 14.28 | 6 | 0.1% |  |
| 13.33 | 1 | < 0.1% |  |
| 12.5 | 2 | < 0.1% |  |
| 11.11 | 4 | 0.1% |  |
| 10 | 1 | < 0.1% |  |
| 9.09 | 4 | 0.1% |  |

8  
Real number (ℝ≥0)

`ZEROS`

|  |  |
| --- | --- |
| Distinct count | 144 |
| Unique (%) | 3.1% |
| Missing | 0 |
| Missing (%) | 0.0% |
| Infinite | 0 |
| Infinite (%) | 0.0% |

|  |  |
| --- | --- |
| Mean | 0.09006737665724841 |
| Minimum | 0.0 |
| Maximum | 5.26 |
| Zeros | 3828 |
| Zeros (%) | 83.2% |
| Memory size | 36.1 KiB |

2020-08-25T01:55:12.406175image/svg+xmlMatplotlib v3.3.1, https://matplotlib.org/

Toggle details

- Statistics
- Histogram(s)
- Common values
- Extreme values

Quantile statistics

|  |  |
| --- | --- |
| Minimum | 0 |
| 5-th percentile | 0 |
| Q1 | 0 |
| median | 0 |
| Q3 | 0 |
| 95-th percentile | 0.65 |
| Maximum | 5.26 |
| Range | 5.26 |
| Interquartile range (IQR) | 0 |

Descriptive statistics

|  |  |
| --- | --- |
| Standard deviation | 0.2786158642 |
| Coefficient of variation (CV) | 3.093416002 |
| Kurtosis | 46.94025552 |
| Mean | 0.09006737666 |
| Median Absolute Deviation (MAD) | 0 |
| Skewness | 5.226066951 |
| Sum | 414.4 |
| Variance | 0.07762679981 |

- Histogram

2020-08-25T01:55:12.519267image/svg+xmlMatplotlib v3.3.1, https://matplotlib.org/ 

**Histogram with fixed size bins** (bins=10)

| Value | Count | Frequency (%) |  |
| --- | --- | --- | --- |
| 0 | 3828 | 83.2% |  |
| 0.09 | 26 | 0.6% |  |
| 0.08 | 24 | 0.5% |  |
| 0.8 | 18 | 0.4% |  |
| 0.1 | 17 | 0.4% |  |
| 0.23 | 16 | 0.3% |  |
| 0.16 | 16 | 0.3% |  |
| 0.24 | 15 | 0.3% |  |
| 0.05 | 15 | 0.3% |  |
| 0.2 | 14 | 0.3% |  |
| 0.06 | 14 | 0.3% |  |
| 0.44 | 13 | 0.3% |  |
| 0.59 | 13 | 0.3% |  |
| 0.4 | 13 | 0.3% |  |
| 0.32 | 13 | 0.3% |  |
| 0.54 | 13 | 0.3% |  |
| 0.28 | 12 | 0.3% |  |
| 0.58 | 12 | 0.3% |  |
| 0.29 | 12 | 0.3% |  |
| 0.27 | 12 | 0.3% |  |
| 0.31 | 11 | 0.2% |  |
| 0.66 | 11 | 0.2% |  |
| 0.25 | 11 | 0.2% |  |
| 0.13 | 10 | 0.2% |  |
| 0.35 | 10 | 0.2% |  |
| Other values (119) | 432 | 9.4% |  |

- Minimum 5 values
- Maximum 5 values

| Value | Count | Frequency (%) |  |
| --- | --- | --- | --- |
| 0 | 3828 | 83.2% |  |
| 0.01 | 1 | < 0.1% |  |
| 0.02 | 2 | < 0.1% |  |
| 0.03 | 4 | 0.1% |  |
| 0.04 | 4 | 0.1% |  |
| 0.05 | 15 | 0.3% |  |
| 0.06 | 14 | 0.3% |  |
| 0.07 | 7 | 0.2% |  |
| 0.08 | 24 | 0.5% |  |
| 0.09 | 26 | 0.6% |  |

| Value | Count | Frequency (%) |  |
| --- | --- | --- | --- |
| 5.26 | 1 | < 0.1% |  |
| 3.33 | 1 | < 0.1% |  |
| 3.23 | 1 | < 0.1% |  |
| 2.59 | 1 | < 0.1% |  |
| 2.5 | 1 | < 0.1% |  |
| 2.48 | 1 | < 0.1% |  |
| 2.38 | 1 | < 0.1% |  |
| 2.35 | 1 | < 0.1% |  |
| 2.29 | 1 | < 0.1% |  |
| 2.12 | 1 | < 0.1% |  |

17  
Real number (ℝ≥0)

`ZEROS`

|  |  |
| --- | --- |
| Distinct count | 229 |
| Unique (%) | 5.0% |
| Missing | 0 |
| Missing (%) | 0.0% |
| Infinite | 0 |
| Infinite (%) | 0.0% |

|  |  |
| --- | --- |
| Mean | 0.18474462073462292 |
| Minimum | 0.0 |
| Maximum | 9.09 |
| Zeros | 3563 |
| Zeros (%) | 77.4% |
| Memory size | 36.1 KiB |

2020-08-25T01:55:12.633208image/svg+xmlMatplotlib v3.3.1, https://matplotlib.org/

Toggle details

- Statistics
- Histogram(s)
- Common values
- Extreme values

Quantile statistics

|  |  |
| --- | --- |
| Minimum | 0 |
| 5-th percentile | 0 |
| Q1 | 0 |
| median | 0 |
| Q3 | 0 |
| 95-th percentile | 1.21 |
| Maximum | 9.09 |
| Range | 9.09 |
| Interquartile range (IQR) | 0 |

Descriptive statistics

|  |  |
| --- | --- |
| Standard deviation | 0.531122422 |
| Coefficient of variation (CV) | 2.874900605 |
| Kurtosis | 47.96167444 |
| Mean | 0.1847446207 |
| Median Absolute Deviation (MAD) | 0 |
| Skewness | 5.413753723 |
| Sum | 850.01 |
| Variance | 0.2820910272 |

- Histogram

2020-08-25T01:55:12.742347image/svg+xmlMatplotlib v3.3.1, https://matplotlib.org/ 

**Histogram with fixed size bins** (bins=10)

| Value | Count | Frequency (%) |  |
| --- | --- | --- | --- |
| 0 | 3563 | 77.4% |  |
| 0.08 | 20 | 0.4% |  |
| 0.05 | 20 | 0.4% |  |
| 1.11 | 20 | 0.4% |  |
| 0.32 | 18 | 0.4% |  |
| 0.44 | 17 | 0.4% |  |
| 0.06 | 17 | 0.4% |  |
| 0.33 | 16 | 0.3% |  |
| 0.12 | 15 | 0.3% |  |
| 0.19 | 14 | 0.3% |  |
| 0.38 | 13 | 0.3% |  |
| 0.55 | 13 | 0.3% |  |
| 0.03 | 13 | 0.3% |  |
| 0.9 | 12 | 0.3% |  |
| 0.36 | 12 | 0.3% |  |
| 0.07 | 12 | 0.3% |  |
| 0.29 | 12 | 0.3% |  |
| 0.27 | 11 | 0.2% |  |
| 0.46 | 11 | 0.2% |  |
| 0.34 | 11 | 0.2% |  |
| 0.39 | 11 | 0.2% |  |
| 0.58 | 11 | 0.2% |  |
| 0.4 | 11 | 0.2% |  |
| 0.11 | 10 | 0.2% |  |
| 0.37 | 10 | 0.2% |  |
| Other values (204) | 708 | 15.4% |  |

- Minimum 5 values
- Maximum 5 values

| Value | Count | Frequency (%) |  |
| --- | --- | --- | --- |
| 0 | 3563 | 77.4% |  |
| 0.01 | 4 | 0.1% |  |
| 0.02 | 7 | 0.2% |  |
| 0.03 | 13 | 0.3% |  |
| 0.04 | 4 | 0.1% |  |
| 0.05 | 20 | 0.4% |  |
| 0.06 | 17 | 0.4% |  |
| 0.07 | 12 | 0.3% |  |
| 0.08 | 20 | 0.4% |  |
| 0.09 | 2 | < 0.1% |  |

| Value | Count | Frequency (%) |  |
| --- | --- | --- | --- |
| 9.09 | 1 | < 0.1% |  |
| 7.69 | 1 | < 0.1% |  |
| 6.66 | 1 | < 0.1% |  |
| 5.33 | 1 | < 0.1% |  |
| 5.26 | 1 | < 0.1% |  |
| 5.06 | 1 | < 0.1% |  |
| 4.87 | 1 | < 0.1% |  |
| 4.83 | 1 | < 0.1% |  |
| 4.51 | 1 | < 0.1% |  |
| 4.44 | 1 | < 0.1% |  |

6  
Real number (ℝ≥0)

`ZEROS`

|  |  |
| --- | --- |
| Distinct count | 173 |
| Unique (%) | 3.8% |
| Missing | 0 |
| Missing (%) | 0.0% |
| Infinite | 0 |
| Infinite (%) | 0.0% |

|  |  |
| --- | --- |
| Mean | 0.11420778091719191 |
| Minimum | 0.0 |
| Maximum | 7.27 |
| Zeros | 3794 |
| Zeros (%) | 82.5% |
| Memory size | 36.1 KiB |

2020-08-25T01:55:12.846341image/svg+xmlMatplotlib v3.3.1, https://matplotlib.org/

Toggle details

- Statistics
- Histogram(s)
- Common values
- Extreme values

Quantile statistics

|  |  |
| --- | --- |
| Minimum | 0 |
| 5-th percentile | 0 |
| Q1 | 0 |
| median | 0 |
| Q3 | 0 |
| 95-th percentile | 0.74 |
| Maximum | 7.27 |
| Range | 7.27 |
| Interquartile range (IQR) | 0 |

Descriptive statistics

|  |  |
| --- | --- |
| Standard deviation | 0.3914413548 |
| Coefficient of variation (CV) | 3.42744909 |
| Kurtosis | 75.41343865 |
| Mean | 0.1142077809 |
| Median Absolute Deviation (MAD) | 0 |
| Skewness | 6.765580469 |
| Sum | 525.47 |
| Variance | 0.1532263342 |

- Histogram

2020-08-25T01:55:13.136960image/svg+xmlMatplotlib v3.3.1, https://matplotlib.org/ 

**Histogram with fixed size bins** (bins=10)

| Value | Count | Frequency (%) |  |
| --- | --- | --- | --- |
| 0 | 3794 | 82.5% |  |
| 0.08 | 30 | 0.7% |  |
| 0.05 | 21 | 0.5% |  |
| 0.5 | 19 | 0.4% |  |
| 0.32 | 19 | 0.4% |  |
| 0.19 | 18 | 0.4% |  |
| 0.25 | 16 | 0.3% |  |
| 0.4 | 14 | 0.3% |  |
| 0.16 | 14 | 0.3% |  |
| 0.1 | 14 | 0.3% |  |
| 0.2 | 13 | 0.3% |  |
| 0.06 | 12 | 0.3% |  |
| 0.33 | 12 | 0.3% |  |
| 0.31 | 12 | 0.3% |  |
| 0.17 | 12 | 0.3% |  |
| 0.68 | 11 | 0.2% |  |
| 0.03 | 11 | 0.2% |  |
| 0.23 | 11 | 0.2% |  |
| 0.38 | 11 | 0.2% |  |
| 0.49 | 11 | 0.2% |  |
| 0.14 | 11 | 0.2% |  |
| 0.09 | 10 | 0.2% |  |
| 0.15 | 10 | 0.2% |  |
| 0.9 | 9 | 0.2% |  |
| 0.64 | 9 | 0.2% |  |
| Other values (148) | 477 | 10.4% |  |

- Minimum 5 values
- Maximum 5 values

| Value | Count | Frequency (%) |  |
| --- | --- | --- | --- |
| 0 | 3794 | 82.5% |  |
| 0.02 | 4 | 0.1% |  |
| 0.03 | 11 | 0.2% |  |
| 0.04 | 8 | 0.2% |  |
| 0.05 | 21 | 0.5% |  |
| 0.06 | 12 | 0.3% |  |
| 0.07 | 7 | 0.2% |  |
| 0.08 | 30 | 0.7% |  |
| 0.09 | 10 | 0.2% |  |
| 0.1 | 14 | 0.3% |  |

| Value | Count | Frequency (%) |  |
| --- | --- | --- | --- |
| 7.27 | 2 | < 0.1% |  |
| 5.4 | 1 | < 0.1% |  |
| 4.54 | 1 | < 0.1% |  |
| 4.08 | 1 | < 0.1% |  |
| 4 | 1 | < 0.1% |  |
| 3.27 | 1 | < 0.1% |  |
| 3.12 | 2 | < 0.1% |  |
| 3.07 | 1 | < 0.1% |  |
| 2.98 | 1 | < 0.1% |  |
| 2.94 | 2 | < 0.1% |  |

4  
Real number (ℝ≥0)

`ZEROS`

|  |  |
| --- | --- |
| Distinct count | 255 |
| Unique (%) | 5.5% |
| Missing | 0 |
| Missing (%) | 0.0% |
| Infinite | 0 |
| Infinite (%) | 0.0% |

|  |  |
| --- | --- |
| Mean | 0.312223429689198 |
| Minimum | 0.0 |
| Maximum | 10.0 |
| Zeros | 2853 |
| Zeros (%) | 62.0% |
| Memory size | 36.1 KiB |

2020-08-25T01:55:13.252497image/svg+xmlMatplotlib v3.3.1, https://matplotlib.org/

Toggle details

- Statistics
- Histogram(s)
- Common values
- Extreme values

Quantile statistics

|  |  |
| --- | --- |
| Minimum | 0 |
| 5-th percentile | 0 |
| Q1 | 0 |
| median | 0 |
| Q3 | 0.38 |
| 95-th percentile | 1.49 |
| Maximum | 10 |
| Range | 10 |
| Interquartile range (IQR) | 0.38 |

Descriptive statistics

|  |  |
| --- | --- |
| Standard deviation | 0.6725127693 |
| Coefficient of variation (CV) | 2.153947159 |
| Kurtosis | 37.94116889 |
| Mean | 0.3122234297 |
| Median Absolute Deviation (MAD) | 0 |
| Skewness | 4.747126114 |
| Sum | 1436.54 |
| Variance | 0.4522734249 |

- Histogram

2020-08-25T01:55:13.354497image/svg+xmlMatplotlib v3.3.1, https://matplotlib.org/ 

**Histogram with fixed size bins** (bins=10)

| Value | Count | Frequency (%) |  |
| --- | --- | --- | --- |
| 0 | 2853 | 62.0% |  |
| 0.36 | 28 | 0.6% |  |
| 0.32 | 26 | 0.6% |  |
| 0.19 | 24 | 0.5% |  |
| 0.8 | 24 | 0.5% |  |
| 0.26 | 22 | 0.5% |  |
| 0.29 | 22 | 0.5% |  |
| 0.14 | 22 | 0.5% |  |
| 0.23 | 21 | 0.5% |  |
| 0.68 | 21 | 0.5% |  |
| 0.53 | 21 | 0.5% |  |
| 0.64 | 21 | 0.5% |  |
| 0.45 | 21 | 0.5% |  |
| 0.34 | 21 | 0.5% |  |
| 0.4 | 20 | 0.4% |  |
| 0.13 | 20 | 0.4% |  |
| 0.65 | 20 | 0.4% |  |
| 0.38 | 19 | 0.4% |  |
| 0.33 | 19 | 0.4% |  |
| 0.27 | 19 | 0.4% |  |
| 0.09 | 19 | 0.4% |  |
| 0.25 | 19 | 0.4% |  |
| 0.43 | 19 | 0.4% |  |
| 0.12 | 19 | 0.4% |  |
| 0.28 | 18 | 0.4% |  |
| Other values (230) | 1243 | 27.0% |  |

- Minimum 5 values
- Maximum 5 values

| Value | Count | Frequency (%) |  |
| --- | --- | --- | --- |
| 0 | 2853 | 62.0% |  |
| 0.02 | 5 | 0.1% |  |
| 0.03 | 1 | < 0.1% |  |
| 0.04 | 12 | 0.3% |  |
| 0.05 | 15 | 0.3% |  |
| 0.06 | 2 | < 0.1% |  |
| 0.07 | 9 | 0.2% |  |
| 0.08 | 15 | 0.3% |  |
| 0.09 | 19 | 0.4% |  |
| 0.1 | 17 | 0.4% |  |

| Value | Count | Frequency (%) |  |
| --- | --- | --- | --- |
| 10 | 1 | < 0.1% |  |
| 9.09 | 1 | < 0.1% |  |
| 8.33 | 1 | < 0.1% |  |
| 7.69 | 1 | < 0.1% |  |
| 7.14 | 1 | < 0.1% |  |
| 6.25 | 4 | 0.1% |  |
| 5.55 | 1 | < 0.1% |  |
| 5.26 | 1 | < 0.1% |  |
| 5 | 2 | < 0.1% |  |
| 4.76 | 3 | 0.1% |  |

40  
Real number (ℝ≥0)

`ZEROS`

|  |  |
| --- | --- |
| Distinct count | 108 |
| Unique (%) | 2.3% |
| Missing | 0 |
| Missing (%) | 0.0% |
| Infinite | 0 |
| Infinite (%) | 0.0% |

|  |  |
| --- | --- |
| Mean | 0.043666594218648117 |
| Minimum | 0.0 |
| Maximum | 7.14 |
| Zeros | 4453 |
| Zeros (%) | 96.8% |
| Memory size | 36.1 KiB |

2020-08-25T01:55:13.464301image/svg+xmlMatplotlib v3.3.1, https://matplotlib.org/

Toggle details

- Statistics
- Histogram(s)
- Common values
- Extreme values

Quantile statistics

|  |  |
| --- | --- |
| Minimum | 0 |
| 5-th percentile | 0 |
| Q1 | 0 |
| median | 0 |
| Q3 | 0 |
| 95-th percentile | 0 |
| Maximum | 7.14 |
| Range | 7.14 |
| Interquartile range (IQR) | 0 |

Descriptive statistics

|  |  |
| --- | --- |
| Standard deviation | 0.3612047007 |
| Coefficient of variation (CV) | 8.271877098 |
| Kurtosis | 193.6192939 |
| Mean | 0.04366659422 |
| Median Absolute Deviation (MAD) | 0 |
| Skewness | 12.58790037 |
| Sum | 200.91 |
| Variance | 0.1304688358 |

- Histogram

2020-08-25T01:55:13.572837image/svg+xmlMatplotlib v3.3.1, https://matplotlib.org/ 

**Histogram with fixed size bins** (bins=10)

| Value | Count | Frequency (%) |  |
| --- | --- | --- | --- |
| 0 | 4453 | 96.8% |  |
| 0.31 | 5 | 0.1% |  |
| 7.14 | 4 | 0.1% |  |
| 4.75 | 3 | 0.1% |  |
| 0.1 | 3 | 0.1% |  |
| 0.34 | 3 | 0.1% |  |
| 1.44 | 3 | 0.1% |  |
| 0.25 | 3 | 0.1% |  |
| 0.68 | 2 | < 0.1% |  |
| 2.56 | 2 | < 0.1% |  |
| 1.92 | 2 | < 0.1% |  |
| 0.89 | 2 | < 0.1% |  |
| 0.46 | 2 | < 0.1% |  |
| 0.36 | 2 | < 0.1% |  |
| 1.16 | 2 | < 0.1% |  |
| 2 | 2 | < 0.1% |  |
| 0.08 | 2 | < 0.1% |  |
| 0.06 | 2 | < 0.1% |  |
| 0.28 | 2 | < 0.1% |  |
| 1.06 | 2 | < 0.1% |  |
| 0.65 | 2 | < 0.1% |  |
| 0.13 | 2 | < 0.1% |  |
| 0.51 | 2 | < 0.1% |  |
| 0.16 | 2 | < 0.1% |  |
| 0.86 | 2 | < 0.1% |  |
| Other values (83) | 90 | 2.0% |  |

- Minimum 5 values
- Maximum 5 values

| Value | Count | Frequency (%) |  |
| --- | --- | --- | --- |
| 0 | 4453 | 96.8% |  |
| 0.02 | 1 | < 0.1% |  |
| 0.03 | 1 | < 0.1% |  |
| 0.04 | 1 | < 0.1% |  |
| 0.06 | 2 | < 0.1% |  |
| 0.07 | 1 | < 0.1% |  |
| 0.08 | 2 | < 0.1% |  |
| 0.1 | 3 | 0.1% |  |
| 0.13 | 2 | < 0.1% |  |
| 0.16 | 2 | < 0.1% |  |

| Value | Count | Frequency (%) |  |
| --- | --- | --- | --- |
| 7.14 | 4 | 0.1% |  |
| 5.88 | 1 | < 0.1% |  |
| 5 | 1 | < 0.1% |  |
| 4.76 | 1 | < 0.1% |  |
| 4.75 | 3 | 0.1% |  |
| 4.28 | 1 | < 0.1% |  |
| 3.26 | 1 | < 0.1% |  |
| 3.25 | 1 | < 0.1% |  |
| 3.22 | 1 | < 0.1% |  |
| 3.12 | 1 | < 0.1% |  |

target  
Boolean

|  |  |
| --- | --- |
| Distinct count | 2 |
| Unique (%) | < 0.1% |
| Missing | 0 |
| Missing (%) | 0.0% |
| Memory size | 36.1 KiB |

|  |  |
| --- | --- |
| 0 | 2788 |
| 1 | 1813 |

Toggle details

- Frequency Table

| Value | Count | Frequency (%) |  |
| --- | --- | --- | --- |
| 0 | 2788 | 60.6% |  |
| 1 | 1813 | 39.4% |  |

# Interactions

0 5 30 13 34 55 27 31 45 12 47 3 43 26 8 17 6 4 40

0 5 30 13 34 55 27 31 45 12 47 3 43 26 8 17 6 4 40

2020-08-25T01:54:17.987323image/svg+xmlMatplotlib v3.3.1, https://matplotlib.org/

2020-08-25T01:54:18.110352image/svg+xmlMatplotlib v3.3.1, https://matplotlib.org/

2020-08-25T01:54:18.238197image/svg+xmlMatplotlib v3.3.1, https://matplotlib.org/

2020-08-25T01:54:18.361217image/svg+xmlMatplotlib v3.3.1, https://matplotlib.org/

2020-08-25T01:54:18.481810image/svg+xmlMatplotlib v3.3.1, https://matplotlib.org/

2020-08-25T01:54:18.618963image/svg+xmlMatplotlib v3.3.1, https://matplotlib.org/

2020-08-25T01:54:18.749367image/svg+xmlMatplotlib v3.3.1, https://matplotlib.org/

2020-08-25T01:54:18.872147image/svg+xmlMatplotlib v3.3.1, https://matplotlib.org/

2020-08-25T01:54:18.994981image/svg+xmlMatplotlib v3.3.1, https://matplotlib.org/

2020-08-25T01:54:19.116857image/svg+xmlMatplotlib v3.3.1, https://matplotlib.org/

2020-08-25T01:54:19.238867image/svg+xmlMatplotlib v3.3.1, https://matplotlib.org/

2020-08-25T01:54:19.365866image/svg+xmlMatplotlib v3.3.1, https://matplotlib.org/

2020-08-25T01:54:19.485395image/svg+xmlMatplotlib v3.3.1, https://matplotlib.org/

2020-08-25T01:54:19.623747image/svg+xmlMatplotlib v3.3.1, https://matplotlib.org/

2020-08-25T01:54:19.752040image/svg+xmlMatplotlib v3.3.1, https://matplotlib.org/

2020-08-25T01:54:19.873125image/svg+xmlMatplotlib v3.3.1, https://matplotlib.org/

2020-08-25T01:54:19.992087image/svg+xmlMatplotlib v3.3.1, https://matplotlib.org/

2020-08-25T01:54:20.123984image/svg+xmlMatplotlib v3.3.1, https://matplotlib.org/

2020-08-25T01:54:20.433341image/svg+xmlMatplotlib v3.3.1, https://matplotlib.org/

0 5 30 13 34 55 27 31 45 12 47 3 43 26 8 17 6 4 40

2020-08-25T01:54:20.562481image/svg+xmlMatplotlib v3.3.1, https://matplotlib.org/

2020-08-25T01:54:20.692522image/svg+xmlMatplotlib v3.3.1, https://matplotlib.org/

2020-08-25T01:54:20.827058image/svg+xmlMatplotlib v3.3.1, https://matplotlib.org/

2020-08-25T01:54:20.966333image/svg+xmlMatplotlib v3.3.1, https://matplotlib.org/

2020-08-25T01:54:21.105646image/svg+xmlMatplotlib v3.3.1, https://matplotlib.org/

2020-08-25T01:54:21.249923image/svg+xmlMatplotlib v3.3.1, https://matplotlib.org/

2020-08-25T01:54:21.380390image/svg+xmlMatplotlib v3.3.1, https://matplotlib.org/

2020-08-25T01:54:21.506541image/svg+xmlMatplotlib v3.3.1, https://matplotlib.org/

2020-08-25T01:54:21.636397image/svg+xmlMatplotlib v3.3.1, https://matplotlib.org/

2020-08-25T01:54:21.772480image/svg+xmlMatplotlib v3.3.1, https://matplotlib.org/

2020-08-25T01:54:21.903605image/svg+xmlMatplotlib v3.3.1, https://matplotlib.org/

2020-08-25T01:54:22.034989image/svg+xmlMatplotlib v3.3.1, https://matplotlib.org/

2020-08-25T01:54:22.166013image/svg+xmlMatplotlib v3.3.1, https://matplotlib.org/

2020-08-25T01:54:22.310445image/svg+xmlMatplotlib v3.3.1, https://matplotlib.org/

2020-08-25T01:54:22.445237image/svg+xmlMatplotlib v3.3.1, https://matplotlib.org/

2020-08-25T01:54:22.574247image/svg+xmlMatplotlib v3.3.1, https://matplotlib.org/

2020-08-25T01:54:22.707675image/svg+xmlMatplotlib v3.3.1, https://matplotlib.org/

2020-08-25T01:54:22.846743image/svg+xmlMatplotlib v3.3.1, https://matplotlib.org/

2020-08-25T01:54:22.982305image/svg+xmlMatplotlib v3.3.1, https://matplotlib.org/

0 5 30 13 34 55 27 31 45 12 47 3 43 26 8 17 6 4 40

2020-08-25T01:54:23.122788image/svg+xmlMatplotlib v3.3.1, https://matplotlib.org/

2020-08-25T01:54:23.258642image/svg+xmlMatplotlib v3.3.1, https://matplotlib.org/

2020-08-25T01:54:23.392811image/svg+xmlMatplotlib v3.3.1, https://matplotlib.org/

2020-08-25T01:54:23.527849image/svg+xmlMatplotlib v3.3.1, https://matplotlib.org/

2020-08-25T01:54:23.661803image/svg+xmlMatplotlib v3.3.1, https://matplotlib.org/

2020-08-25T01:54:23.806568image/svg+xmlMatplotlib v3.3.1, https://matplotlib.org/

2020-08-25T01:54:23.941858image/svg+xmlMatplotlib v3.3.1, https://matplotlib.org/

2020-08-25T01:54:24.072072image/svg+xmlMatplotlib v3.3.1, https://matplotlib.org/

2020-08-25T01:54:24.204160image/svg+xmlMatplotlib v3.3.1, https://matplotlib.org/

2020-08-25T01:54:24.342085image/svg+xmlMatplotlib v3.3.1, https://matplotlib.org/

2020-08-25T01:54:24.474737image/svg+xmlMatplotlib v3.3.1, https://matplotlib.org/

2020-08-25T01:54:24.604478image/svg+xmlMatplotlib v3.3.1, https://matplotlib.org/

2020-08-25T01:54:24.937359image/svg+xmlMatplotlib v3.3.1, https://matplotlib.org/

2020-08-25T01:54:25.084758image/svg+xmlMatplotlib v3.3.1, https://matplotlib.org/

2020-08-25T01:54:25.226176image/svg+xmlMatplotlib v3.3.1, https://matplotlib.org/

2020-08-25T01:54:25.361396image/svg+xmlMatplotlib v3.3.1, https://matplotlib.org/

2020-08-25T01:54:25.495557image/svg+xmlMatplotlib v3.3.1, https://matplotlib.org/

2020-08-25T01:54:25.636247image/svg+xmlMatplotlib v3.3.1, https://matplotlib.org/

2020-08-25T01:54:25.770318image/svg+xmlMatplotlib v3.3.1, https://matplotlib.org/

0 5 30 13 34 55 27 31 45 12 47 3 43 26 8 17 6 4 40

2020-08-25T01:54:25.909065image/svg+xmlMatplotlib v3.3.1, https://matplotlib.org/

2020-08-25T01:54:26.034068image/svg+xmlMatplotlib v3.3.1, https://matplotlib.org/

2020-08-25T01:54:26.164726image/svg+xmlMatplotlib v3.3.1, https://matplotlib.org/

2020-08-25T01:54:26.303439image/svg+xmlMatplotlib v3.3.1, https://matplotlib.org/

2020-08-25T01:54:26.430839image/svg+xmlMatplotlib v3.3.1, https://matplotlib.org/

2020-08-25T01:54:26.572967image/svg+xmlMatplotlib v3.3.1, https://matplotlib.org/

2020-08-25T01:54:26.703650image/svg+xmlMatplotlib v3.3.1, https://matplotlib.org/

2020-08-25T01:54:26.826771image/svg+xmlMatplotlib v3.3.1, https://matplotlib.org/

2020-08-25T01:54:26.956197image/svg+xmlMatplotlib v3.3.1, https://matplotlib.org/

2020-08-25T01:54:27.086555image/svg+xmlMatplotlib v3.3.1, https://matplotlib.org/

2020-08-25T01:54:27.213445image/svg+xmlMatplotlib v3.3.1, https://matplotlib.org/

2020-08-25T01:54:27.338944image/svg+xmlMatplotlib v3.3.1, https://matplotlib.org/

2020-08-25T01:54:27.462852image/svg+xmlMatplotlib v3.3.1, https://matplotlib.org/

2020-08-25T01:54:27.602362image/svg+xmlMatplotlib v3.3.1, https://matplotlib.org/

2020-08-25T01:54:27.736335image/svg+xmlMatplotlib v3.3.1, https://matplotlib.org/

2020-08-25T01:54:27.879157image/svg+xmlMatplotlib v3.3.1, https://matplotlib.org/

2020-08-25T01:54:28.002151image/svg+xmlMatplotlib v3.3.1, https://matplotlib.org/

2020-08-25T01:54:28.138421image/svg+xmlMatplotlib v3.3.1, https://matplotlib.org/

2020-08-25T01:54:28.268908image/svg+xmlMatplotlib v3.3.1, https://matplotlib.org/

0 5 30 13 34 55 27 31 45 12 47 3 43 26 8 17 6 4 40

2020-08-25T01:54:28.402124image/svg+xmlMatplotlib v3.3.1, https://matplotlib.org/

2020-08-25T01:54:28.546232image/svg+xmlMatplotlib v3.3.1, https://matplotlib.org/

2020-08-25T01:54:28.698838image/svg+xmlMatplotlib v3.3.1, https://matplotlib.org/

2020-08-25T01:54:28.845335image/svg+xmlMatplotlib v3.3.1, https://matplotlib.org/

2020-08-25T01:54:28.996068image/svg+xmlMatplotlib v3.3.1, https://matplotlib.org/

2020-08-25T01:54:29.152322image/svg+xmlMatplotlib v3.3.1, https://matplotlib.org/

2020-08-25T01:54:29.486434image/svg+xmlMatplotlib v3.3.1, https://matplotlib.org/

2020-08-25T01:54:29.628955image/svg+xmlMatplotlib v3.3.1, https://matplotlib.org/

2020-08-25T01:54:29.772388image/svg+xmlMatplotlib v3.3.1, https://matplotlib.org/

2020-08-25T01:54:29.913507image/svg+xmlMatplotlib v3.3.1, https://matplotlib.org/

2020-08-25T01:54:30.057279image/svg+xmlMatplotlib v3.3.1, https://matplotlib.org/

2020-08-25T01:54:30.202507image/svg+xmlMatplotlib v3.3.1, https://matplotlib.org/

2020-08-25T01:54:30.346279image/svg+xmlMatplotlib v3.3.1, https://matplotlib.org/

2020-08-25T01:54:30.501618image/svg+xmlMatplotlib v3.3.1, https://matplotlib.org/

2020-08-25T01:54:30.647812image/svg+xmlMatplotlib v3.3.1, https://matplotlib.org/

2020-08-25T01:54:30.795737image/svg+xmlMatplotlib v3.3.1, https://matplotlib.org/

2020-08-25T01:54:30.937745image/svg+xmlMatplotlib v3.3.1, https://matplotlib.org/

2020-08-25T01:54:31.089444image/svg+xmlMatplotlib v3.3.1, https://matplotlib.org/

2020-08-25T01:54:31.235005image/svg+xmlMatplotlib v3.3.1, https://matplotlib.org/

0 5 30 13 34 55 27 31 45 12 47 3 43 26 8 17 6 4 40

2020-08-25T01:54:31.387022image/svg+xmlMatplotlib v3.3.1, https://matplotlib.org/

2020-08-25T01:54:31.518277image/svg+xmlMatplotlib v3.3.1, https://matplotlib.org/

2020-08-25T01:54:31.656073image/svg+xmlMatplotlib v3.3.1, https://matplotlib.org/

2020-08-25T01:54:31.789898image/svg+xmlMatplotlib v3.3.1, https://matplotlib.org/

2020-08-25T01:54:31.917571image/svg+xmlMatplotlib v3.3.1, https://matplotlib.org/

2020-08-25T01:54:32.058854image/svg+xmlMatplotlib v3.3.1, https://matplotlib.org/

2020-08-25T01:54:32.186416image/svg+xmlMatplotlib v3.3.1, https://matplotlib.org/

2020-08-25T01:54:32.316639image/svg+xmlMatplotlib v3.3.1, https://matplotlib.org/

2020-08-25T01:54:32.444915image/svg+xmlMatplotlib v3.3.1, https://matplotlib.org/

2020-08-25T01:54:32.575420image/svg+xmlMatplotlib v3.3.1, https://matplotlib.org/

2020-08-25T01:54:32.706071image/svg+xmlMatplotlib v3.3.1, https://matplotlib.org/

2020-08-25T01:54:32.846238image/svg+xmlMatplotlib v3.3.1, https://matplotlib.org/

2020-08-25T01:54:32.970502image/svg+xmlMatplotlib v3.3.1, https://matplotlib.org/

2020-08-25T01:54:33.112470image/svg+xmlMatplotlib v3.3.1, https://matplotlib.org/

2020-08-25T01:54:33.252137image/svg+xmlMatplotlib v3.3.1, https://matplotlib.org/

2020-08-25T01:54:33.386739image/svg+xmlMatplotlib v3.3.1, https://matplotlib.org/

2020-08-25T01:54:33.514988image/svg+xmlMatplotlib v3.3.1, https://matplotlib.org/

2020-08-25T01:54:33.654312image/svg+xmlMatplotlib v3.3.1, https://matplotlib.org/

2020-08-25T01:54:33.780518image/svg+xmlMatplotlib v3.3.1, https://matplotlib.org/

0 5 30 13 34 55 27 31 45 12 47 3 43 26 8 17 6 4 40

2020-08-25T01:54:34.106747image/svg+xmlMatplotlib v3.3.1, https://matplotlib.org/

2020-08-25T01:54:34.230011image/svg+xmlMatplotlib v3.3.1, https://matplotlib.org/

2020-08-25T01:54:34.357505image/svg+xmlMatplotlib v3.3.1, https://matplotlib.org/

2020-08-25T01:54:34.485290image/svg+xmlMatplotlib v3.3.1, https://matplotlib.org/

2020-08-25T01:54:34.605089image/svg+xmlMatplotlib v3.3.1, https://matplotlib.org/

2020-08-25T01:54:34.740574image/svg+xmlMatplotlib v3.3.1, https://matplotlib.org/

2020-08-25T01:54:34.867085image/svg+xmlMatplotlib v3.3.1, https://matplotlib.org/

2020-08-25T01:54:34.986435image/svg+xmlMatplotlib v3.3.1, https://matplotlib.org/

2020-08-25T01:54:35.109270image/svg+xmlMatplotlib v3.3.1, https://matplotlib.org/

2020-08-25T01:54:35.241815image/svg+xmlMatplotlib v3.3.1, https://matplotlib.org/

2020-08-25T01:54:35.368730image/svg+xmlMatplotlib v3.3.1, https://matplotlib.org/

2020-08-25T01:54:35.492412image/svg+xmlMatplotlib v3.3.1, https://matplotlib.org/

2020-08-25T01:54:35.609008image/svg+xmlMatplotlib v3.3.1, https://matplotlib.org/

2020-08-25T01:54:35.741803image/svg+xmlMatplotlib v3.3.1, https://matplotlib.org/

2020-08-25T01:54:35.869454image/svg+xmlMatplotlib v3.3.1, https://matplotlib.org/

2020-08-25T01:54:35.992707image/svg+xmlMatplotlib v3.3.1, https://matplotlib.org/

2020-08-25T01:54:36.112139image/svg+xmlMatplotlib v3.3.1, https://matplotlib.org/

2020-08-25T01:54:36.241169image/svg+xmlMatplotlib v3.3.1, https://matplotlib.org/

2020-08-25T01:54:36.367468image/svg+xmlMatplotlib v3.3.1, https://matplotlib.org/

0 5 30 13 34 55 27 31 45 12 47 3 43 26 8 17 6 4 40

2020-08-25T01:54:36.498827image/svg+xmlMatplotlib v3.3.1, https://matplotlib.org/

2020-08-25T01:54:36.619741image/svg+xmlMatplotlib v3.3.1, https://matplotlib.org/

2020-08-25T01:54:36.747413image/svg+xmlMatplotlib v3.3.1, https://matplotlib.org/

2020-08-25T01:54:36.886409image/svg+xmlMatplotlib v3.3.1, https://matplotlib.org/

2020-08-25T01:54:37.015671image/svg+xmlMatplotlib v3.3.1, https://matplotlib.org/

2020-08-25T01:54:37.152436image/svg+xmlMatplotlib v3.3.1, https://matplotlib.org/

2020-08-25T01:54:37.279595image/svg+xmlMatplotlib v3.3.1, https://matplotlib.org/

2020-08-25T01:54:37.406322image/svg+xmlMatplotlib v3.3.1, https://matplotlib.org/

2020-08-25T01:54:37.528119image/svg+xmlMatplotlib v3.3.1, https://matplotlib.org/

2020-08-25T01:54:37.648265image/svg+xmlMatplotlib v3.3.1, https://matplotlib.org/

2020-08-25T01:54:37.770891image/svg+xmlMatplotlib v3.3.1, https://matplotlib.org/

2020-08-25T01:54:37.904453image/svg+xmlMatplotlib v3.3.1, https://matplotlib.org/

2020-08-25T01:54:38.033095image/svg+xmlMatplotlib v3.3.1, https://matplotlib.org/

2020-08-25T01:54:38.171113image/svg+xmlMatplotlib v3.3.1, https://matplotlib.org/

2020-08-25T01:54:38.483745image/svg+xmlMatplotlib v3.3.1, https://matplotlib.org/

2020-08-25T01:54:38.621563image/svg+xmlMatplotlib v3.3.1, https://matplotlib.org/

2020-08-25T01:54:38.743815image/svg+xmlMatplotlib v3.3.1, https://matplotlib.org/

2020-08-25T01:54:38.877411image/svg+xmlMatplotlib v3.3.1, https://matplotlib.org/

2020-08-25T01:54:39.002334image/svg+xmlMatplotlib v3.3.1, https://matplotlib.org/

0 5 30 13 34 55 27 31 45 12 47 3 43 26 8 17 6 4 40

2020-08-25T01:54:39.134667image/svg+xmlMatplotlib v3.3.1, https://matplotlib.org/

2020-08-25T01:54:39.256313image/svg+xmlMatplotlib v3.3.1, https://matplotlib.org/

2020-08-25T01:54:39.385531image/svg+xmlMatplotlib v3.3.1, https://matplotlib.org/

2020-08-25T01:54:39.517088image/svg+xmlMatplotlib v3.3.1, https://matplotlib.org/

2020-08-25T01:54:39.640132image/svg+xmlMatplotlib v3.3.1, https://matplotlib.org/

2020-08-25T01:54:39.777424image/svg+xmlMatplotlib v3.3.1, https://matplotlib.org/

2020-08-25T01:54:39.910762image/svg+xmlMatplotlib v3.3.1, https://matplotlib.org/

2020-08-25T01:54:40.030319image/svg+xmlMatplotlib v3.3.1, https://matplotlib.org/

2020-08-25T01:54:40.151574image/svg+xmlMatplotlib v3.3.1, https://matplotlib.org/

2020-08-25T01:54:40.274279image/svg+xmlMatplotlib v3.3.1, https://matplotlib.org/

2020-08-25T01:54:40.401988image/svg+xmlMatplotlib v3.3.1, https://matplotlib.org/

2020-08-25T01:54:40.525004image/svg+xmlMatplotlib v3.3.1, https://matplotlib.org/

2020-08-25T01:54:40.650600image/svg+xmlMatplotlib v3.3.1, https://matplotlib.org/

2020-08-25T01:54:40.786458image/svg+xmlMatplotlib v3.3.1, https://matplotlib.org/

2020-08-25T01:54:40.915976image/svg+xmlMatplotlib v3.3.1, https://matplotlib.org/

2020-08-25T01:54:41.039712image/svg+xmlMatplotlib v3.3.1, https://matplotlib.org/

2020-08-25T01:54:41.161943image/svg+xmlMatplotlib v3.3.1, https://matplotlib.org/

2020-08-25T01:54:41.298860image/svg+xmlMatplotlib v3.3.1, https://matplotlib.org/

2020-08-25T01:54:41.432178image/svg+xmlMatplotlib v3.3.1, https://matplotlib.org/

0 5 30 13 34 55 27 31 45 12 47 3 43 26 8 17 6 4 40

2020-08-25T01:54:41.562754image/svg+xmlMatplotlib v3.3.1, https://matplotlib.org/

2020-08-25T01:54:41.688119image/svg+xmlMatplotlib v3.3.1, https://matplotlib.org/

2020-08-25T01:54:41.821689image/svg+xmlMatplotlib v3.3.1, https://matplotlib.org/

2020-08-25T01:54:41.957220image/svg+xmlMatplotlib v3.3.1, https://matplotlib.org/

2020-08-25T01:54:42.086215image/svg+xmlMatplotlib v3.3.1, https://matplotlib.org/

2020-08-25T01:54:42.228338image/svg+xmlMatplotlib v3.3.1, https://matplotlib.org/

2020-08-25T01:54:42.359036image/svg+xmlMatplotlib v3.3.1, https://matplotlib.org/

2020-08-25T01:54:42.491127image/svg+xmlMatplotlib v3.3.1, https://matplotlib.org/

2020-08-25T01:54:42.617035image/svg+xmlMatplotlib v3.3.1, https://matplotlib.org/

2020-08-25T01:54:42.931060image/svg+xmlMatplotlib v3.3.1, https://matplotlib.org/

2020-08-25T01:54:43.062454image/svg+xmlMatplotlib v3.3.1, https://matplotlib.org/

2020-08-25T01:54:43.191529image/svg+xmlMatplotlib v3.3.1, https://matplotlib.org/
